# Immune-related serious adverse events with immune checkpoint inhibitors: Systematic review and network meta-analysis

**DOI:** 10.1007/s00228-024-03647-z

**Published:** 2024-02-19

**Authors:** Clara Oliveira, Beatrice Mainoli, Gonçalo S. Duarte, Tiago Machado, Rita G. Tinoco, Miguel Esperança-Martins, Joaquim J. Ferreira, João Costa

**Affiliations:** 1 Laboratório de Farmacologia Clínica e Terapêutica, Faculdade de Medicina, Universidade de Lisboa, Avenida Professor Egas Moniz, 1649-028 Lisbon, Portugal; 2 Instituto de Medicina Molecular João Lobo Antunes, Faculdade de Medicina, Universidade de Lisboa, Lisbon, Portugal; 3 Clinical Research Unit, Research Center of IPO Porto (CI-IPOP)/RISE@CI-IPOP (Health Research Network), Portuguese Oncology Institute of Porto (IPO Porto)/Porto Comprehensive Cancer Center (Porto.CCC), Porto, Portugal; 4 Clinical Pharmacology Department, Hospital da Luz Lisboa, Lisbon, Portugal; 5 Departamento Médico Grunenthal, SA Lisbon, Portugal; 6 Department of Medical Oncology, Centro Hospitalar Universitário Lisboa Norte, Hospital Santa Maria, Lisbon, Portugal; 7 7CNS–Campus Neurológico Sénior, Torres Vedras, Portugal; 8 Centro de Estudos de Medicina Baseada na Evidência, Faculdade de Medicina, Universidade de Lisboa, Lisbon, Portugal

**Keywords:** Systematic review, Network meta-analysis, Clinical trials, Immune check-point inhibitors, Cancer, Safety

## Abstract

**Purpose:**

Immune checkpoint inhibitors (ICIs) have revolutionized cancer treatment, though uncertainty exists regarding their immune-related safety. The objective of this study was to assess the comparative safety profile (odds ratio) of ICIs and estimate the absolute rate of immune-related serious adverse events (irSAEs) in cancer patients undergoing treatment with ICIs.

**Methods:**

We searched for randomized trials till February 2021, including all ICIs for all cancers. Primary outcome was overall irSAEs, and secondary outcomes were pneumonitis, colitis, hepatitis, hypophysitis, myocarditis, nephritis, and pancreatitis. We conducted Bayesian network meta-analyses, estimated absolute rates and ranked treatments according to the surface under the cumulative ranking curve (SUCRA).

**Results:**

We included 96 trials (52,811 participants, median age 62 years). Risk of bias was high in most trials. Most cancers were non-small cell lung cancer (28 trials) and melanoma (15 trials). The worst-ranked ICI was ipilimumab (SUCRA 14%; event rate 848/10,000 patients) while the best-ranked ICI was atezolizumab (SUCRA 82%; event rate 119/10,000 patients).

**Conclusion:**

Each ICI showed a unique safety profile, with certain events more frequently observed with specific ICIs, which should be considered when managing cancer patients.

**Supplementary Information:**

The online version contains supplementary material available at 10.1007/s00228-024-03647-z.

## Introduction

Immune checkpoint inhibitors (ICIs) have revolutionized the treatment of a vast array of cancers, both in a curative and palliative context [1, 2]. Their current use has focused on anti-CTLA-4 antibodies (ipilimumab, tremelimumab), anti-PD-1 agents (pembrolizumab, nivolumab, cemiplimab), and anti-PD-L1 agents (atezolizumab, avelumab, durvalumab). By blocking intrinsic immunity downregulators, ICIs increase antitumor immunity [3] increasing T cell activation and proliferation, reducing Treg functions, and possibly boosting humoral autoimmunity [4].

Although commonalities exist between the toxicity profiles of different ICIs, there are important differences in the frequency and presentation of specific immune-related adverse events (irAEs) [5]. These events occur via T cell activation and cross-reactivity between antitumor T cells and similar antigens on healthy cells, increased cytokine secretion, autoantibody expansion, or direct binding of monoclonal antibodies to normal tissues [4, 6, 7]. The clinical relevance of irAEs is heterogeneous. Some do not motivate any change in patient care, while others may lead to treatment suspension, disability, hospitalization, or death.

A previous systematic review and network meta-analysis by Xu and colleagues [8] estimated the comparative risk of several irAEs. Based on the 36 phase II/III trials included in this review, the authors estimated a pooled incidence ranging between 54 and 76% for all irAEs, without a differentiation based on their clinical importance, but rather on the severity of the events.

In this network meta-analysis, we chose to focus on adverse events with a major impact in patient care, namely, serious adverse events. We aimed to estimate the frequency of immune-related serious adverse events (irSAEs) as a whole and specifically regarding selected irSAEs: pneumonitis, myocarditis, colitis, nephritis, pancreatitis, hepatitis, and hypophysitis. These specific adverse events were chosen based on their severity and impact on morbidity, mortality, and treatment discontinuation.

## Methods

### Eligibility criteria

We included randomized trials conducted in adult (i.e., > 17 years) with any type of cancer treated with any type of ICI. We compared ICIs with the following: (1) other ICIs, (2) conventional therapy (chemotherapy, targeted therapy, or their combination), (3) placebo or no intervention, or (4) combinations of the previous interventions. We only included trials published in English. We imposed no restrictions on the number of centers, regional area, or year of publication.

### Search and selection

We searched MEDLINE, Embase, CENTRAL, and ClinicalTrials.gov, from inception to February 2021. The full search strategy is provided in the Supplementary Material. Two reviewers independently screened the search results. Disagreements were resolved by consensus. The reasons for exclusion were recorded at the full-text screening stage.

### Data extraction and bias assessment

Reviewers independently extracted study data. Disagreements were resolved by adjudication. Risk of bias was independently evaluated using the Cochrane risk of bias tool [9]. Disagreements were resolved by consensus. The overall risk of bias for each trial was divided as high or low risk [9].

Additionally, we used R (version 4.1.0) to retrieve publicly available information on trial characteristics and data on safety results from the Access to Aggregate Content of ClinicalTrials.gov database [10]. This allowed us to validate the manually extracted data, and enabled automatic updates to the result data anytime updates were made in the ClinicalTrials.gov database.

### Outcomes

The primary outcome was the sum of serious pneumonitis, myocarditis, colitis, nephritis, pancreatitis, hepatitis, and hypophysitis, which we named overall irSAEs. Our secondary outcomes were serious pneumonitis, myocarditis, colitis, nephritis, pancreatitis, hepatitis, and hypophysitis. All were analyzed as binary outcomes (i.e., had or did not have an event) and reported as an odds ratio (OR) and associated 95% credible intervals (CrI). ORs lower than one indicate a lower event risk. We adopted the regulatory definition of serious adverse event (SAE) used for reporting in clinical trials. This refers to any untoward medical occurrence that results in death, is life-threatening, requires or prolongs hospitalization, results in persistent or significant disability or congenital anomaly, or other events that require intervention to prevent one of the other outcomes [11].

### Data analysis

Detailed statistical methods are provided in the Appendix B.1 of the Supplementary Material. The network meta-analyses were performed with a Bayesian hierarchical model using R (version 4.0.5). All outcomes were analyzed using log ORs, binomial likelihood, and cloglog link.

To rank the treatments, we used the surface under the cumulative ranking curve (SUCRA) [12]. Higher SUCRA values indicate that a higher probability of that intervention is associated with a lower risk of developing an event. We synthetized results by comparing the effect of each intervention with conventional therapy. Additionally, we assessed the absolute rate of each outcome per 10,000 patients [13]. For all outcomes, analyses were conducted at the level of individual interventions and treatment modalities.

Reporting is according to PRISMA guidelines [14].

## Results

### General results

We included 96 trials with a combined 52,811 participants, published between 2011 and 2021. Trial references and characteristics are summarized in the Tables B.1 and B.2 of the Supplementary Material. The median trial sample size was 559 (interquartile range (IQR) 75 to 763). The median participant age was 62.1 years (IQR 60.3 to 74.3), and 37.0% of the overall trial participants were female (19,540 of 52,811). Thirty-four trials (35.4%) used a double-blind design. Nine trials were unpublished (9.4%).

The number of trials and participants per type of cancer is summarized in Table B.2.3 of the Supplementary Material. Notably, 28 trials (29.2%) were conducted in patients with non-small cell lung cancer (17,014 combined participants; 32.2%), and 15 trials (15.6%) were conducted in patients with melanoma (8008 combined participants; 15.2%).

The studied interventions of the included trials are detailed in Table B.2.2 of the Supplementary Material. Notably, 18 trials (18.8%) assessed pembrolizumab alone (5609 combined participants; 10.6%), and 18 trials (18.8%) assessed nivolumab alone (4116 combined participants; 7.8%). When the interventions were analyzed as treatment modalities, the most frequently studied were anti-PD-1/anti-PD-L1 inhibitors alone and anti-PD-1/anti-PD-L1 inhibitors plus conventional therapy, with 25,106 (47.5%) and 11,924 (22.6%) combined participants, in 54 and 25 trials, respectively.

### Risk of bias

Regarding bias assessment, 79 trials (82.3%) had a high overall risk of bias. Across trials, there were specific domains contributing to the high risk of bias; however, the most frequent issue relates to performance bias. The risk of bias assessments for the individual trials is available in the Table B.3 of the Supplementary Material.

### Model properties

For all outcomes, at both the level of individual interventions and treatment modalities, only the random-effect models had similar total residual deviances, when compared with the total number of data points, indicating an adequate fit of the results. Therefore, the results presented throughout pertain exclusively to the random-effect models. Table B.4 of the Supplementary Material details on the model fit for each outcome. Pairwise meta-analysis globally showed low levels of heterogeneity across most outcomes (Fig. B.4 of the Supplementary Material). Regarding heterogeneity, for all outcomes, the between-study SD was deemed to be acceptable. The nodesplit models do not suggest inconsistency. Comparisons including placebo/no intervention showed inconsistency (Fig. B.5 of the Supplementary Material). Regarding meta-regressions based on overall survival, progression-free survival, bias, and sex distribution, we found no significant effect (Table B.5 of the Supplementary Material). We did not find evidence of publication bias in any outcome apart from colitis (*p* value = 0.02; Table B.6 of the Supplementary Material). The full league tables for all outcomes, estimated absolute event rates, and SUCRA values are provided in the Supplementary Material.

### Overall irSAEs

**Fig. 1:**
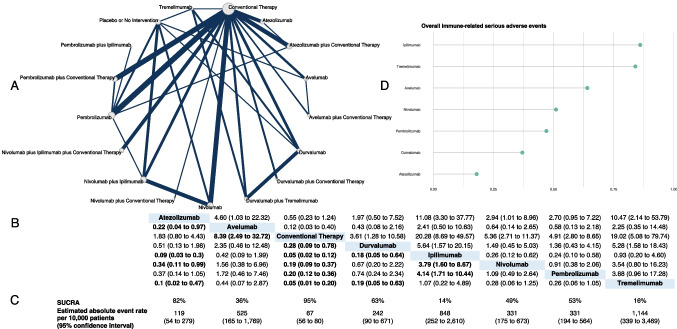
Network meta-analysis of overall immune-related serious adverse events. **A** Network plot showing comparisons in overall immune-related serious adverse events between nodes (gray circles), each representing an intervention. The size of each node is proportional to the total number of participants assigned to the intervention. The width of each connecting line is proportional to the number of studies performing head-to-head comparisons between the two nodes. **B** League table showing the comparative safety profile of each intervention in terms of this outcome. Values in each cell refer to odds ratios and corresponding 95% credible intervals. The interventions are ordered alphabetically. Significant results are in bold. **C** Estimated absolute event rates for each intervention, expressed as rate per 10,000 patients, with corresponding 95% confidence intervals and SUCRA, expressed as a percentage, with higher values indicating a higher certainty that an intervention is superior in terms of the risk of this outcome. **D** Lollipop plot expressing 1-SUCRA values, where greater values indicate a greater likelihood of overall immune-related serious adverse events

Overall, 96 trials (49,941 participants), with 13 multi-arm trials, were pooled for this outcome, consisting of 19 interventions (Fig. 1A). Pembrolizumab (18 trials, 5609 participants) and nivolumab (18 trials, 4116 participants) were the most frequently investigated ICIs. We found evidence of statistical heterogeneity exclusively in the comparison of anti-CTLA-4 versus anti-PD1/anti-PD-L1 (*I*^2^ = 90%; Fig. B.4.1 of the Supplementary Material).

The best-ranked treatment modality was anti-PD-1/PD-L1 plus conventional therapy (SUCRA 61%), while the worst ranked was anti-CTLA-4 (SUCRA 7%). Anti-CTLA-4 was associated with significantly increased odds of irSAEs compared with conventional therapy (OR 24.41, 95% CrI 11.11 to 58.04), anti-PD-1/PD-L1 plus conventional therapy (OR 6.23, 95% CrI 2.58 to 15.77), and anti-PD-1/anti-PD-L1 (OR 4.67, 95% CrI 2.32 to 9.64).

The best-ranked single ICI was atezolizumab (SUCRA 82%), while the worst ranked was ipilimumab (SUCRA 14%). The league plot can be found in Fig. 1B. Ipilimumab was associated with an estimated absolute event rate of 848 irSAEs per 10,000 patients (95% CI 252 to 2610; Fig. 1C). Figure 1D shows a lollipop plot with the treatment rank values (1-SUCRA), where greater values indicate a greater risk of overall irSAEs.

### Pneumonitis

Overall, 82 trials (47,634 participants) were pooled for this outcome, consisting of 19 interventions (Fig. B.2.1.A of the Supplementary Material). Regarding heterogeneity, we found low values of *I*^2^ for all comparisons (Fig. B.4.2 of the Supplementary Material).

The best-ranked treatment modality was anti-CTLA-4 plus conventional therapy (SUCRA 76%), while the worst ranked was ICI combination (SUCRA 3%). ICI combination was associated with significantly increased odds of pneumonitis compared with conventional therapy (OR 11.66, 95% CrI 5.71 to 26.25), anti-CTLA-4 plus conventional therapy (OR 8.91, 95% CrI 2.11 to 38.68), anti-PD-1/PD-L1 plus conventional therapy (OR 4.01, 95% CrI 1.71 to 9.93), and anti-PD-1/anti-PD-L1 (OR 2.44, 95% CrI 1.29 to 4.71). The league plot can be found in Fig. B.2.1.B of the Supplementary Material.

The best-ranked single ICI was atezolizumab (SUCRA 73%), while the worst ranked was ipilimumab (SUCRA 25%). Ipilimumab was associated with an estimated absolute event rate of 187 serious pneumonitis episodes per 10,000 patients (95% CI 62 to 608; Fig. B.2.1.C of the Supplementary Material).

### Colitis

Overall, 81 trials (46,058 participants) were pooled for this outcome, consisting of 18 interventions (Fig. B.2.2.A of the Supplementary Material). We found evidence of statistical heterogeneity exclusively in the comparison of anti-CTLA-4 versus anti-PD1/anti-PD-L1 (*I*^2^ = 67%; Fig. B.4.4 of the Supplementary Material).

The best-ranked treatment modality was anti-PD-1/PD-L1 (SUCRA 61%), while the worst ranked was ICI combination (SUCRA 1%). ICI combination was associated with significantly increased odds of colitis compared with conventional therapy (OR 32.06, 95% CrI 15.95 to 73.32), anti-PD-1/PD-L1 plus conventional therapy (OR 6.56, 95% CrI 2.81 to 16.16), and anti-PD-1 or anti-PD-L1 (OR 7.85, 95% CrI 4.67 to 14.49). The league plot can be found in Fig. B.2.2.B of the Supplementary Material.

The best-ranked single ICI was nivolumab (SUCRA 80%), while the worst ranked was tremelimumab (SUCRA 11%). Tremelimumab was associated with an estimated absolute event rate of 552 serious colitis episodes per 10,000 patients (95% CI 117 to 2689; Fig. B.2.2.C of the Supplementary Material).

### Hepatitis

Overall, 62 trials (36,303 participants) were pooled for this outcome, consisting of 18 interventions (Fig. B.2.3.A of the Supplementary Material). Regarding heterogeneity, we found low values of *I*^2^ for all comparisons (Fig. B.4.7 of the Supplementary Material).

The best-ranked treatment modality was anti-PD-1/PD-L1 plus conventional therapy (SUCRA 58%), while the worst ranked was anti-CTLA-4 plus conventional therapy (SUCRA 5%). Anti-CTLA-4 plus conventional therapy was associated with significantly increased odds of hepatitis compared with conventional therapy (OR 200.17, 95% CrI 6.61 to 297,489), anti-PD-1/PD-L1 plus conventional therapy (OR 48.32, 95% CrI 1.34 to 71,719), and anti-PD-1 or anti-PD-L1 (OR 36.83, 95% CrI 1.05 to 56,704.7). The league plot can be found in Fig. B.2.3.B of the Supplementary Material.

The best-ranked single ICI was atezolizumab (SUCRA 84%), while the worst ranked was ipilimumab (SUCRA 34%). Ipilimumab was associated with an estimated absolute event rate of 50 serious hepatitis episodes per 10,000 patients (95% CI 13 to 189; Fig. B.2.3.C of the Supplementary Material).

### Hypophysitis

Overall, 50 trials (30,868 participants) were pooled for this outcome, consisting of 17 individual interventions (Fig. B.2.4.A of the Supplementary Material). Regarding heterogeneity, we found low values of *I*^2^ for all comparisons (Fig. B.4.8 of the Supplementary Material).

The best-ranked treatment modality was anti-CTLA-4 plus conventional therapy (SUCRA 64%), while the worst ranked was anti-CTLA-4 (SUCRA 9%). Anti-CTLA-4 was associated with statistically significant increased odds of hypophysitis compared with conventional therapy (OR 60.96, 95% CrI 15.88 to 374.52), anti-CTLA-4 plus conventional therapy (OR 15.49, 95% CrI 1.96 to 142.73), and anti-PD-1/PD-L1 (OR 4.81, 95% CrI 2.31 to 11.59). The full league plot indicating relative treatment effects can be found in Fig. B.2.4.B of the Supplementary Material.

At the level of individual interventions, the best-ranked single ICI was tremelimumab (SUCRA 85%), while the worst ranked was ipilimumab (SUCRA 21%). Ipilimumab was associated with an estimated absolute event rate of 422 serious hypophysitis episodes per 10,000 patients (95% CI 124 to 1,565; Fig. B.2.4.C of the Supplementary Material).

### Myocarditis, nephritis, and pancreatitis

We found considerable fewer trials reporting myocarditis (21 trials, 12,936 participants), nephritis (38 trials, 26,616 participants), and pancreatitis (40 trials, 26,990 participants). Below we present the main findings for each outcome regarding individual interventions, though detailed analyses can be found in the Supplementary Material.

For myocarditis, the best-ranked single ICI was pembrolizumab (SUCRA 86%), while the worst ranked was atezolizumab (SUCRA 17%). Atezolizumab was associated with an estimated absolute event rate of 1127 serious myocarditis episodes per 10,000 patients (95% CI 20 to 9917).

For nephritis, the best-ranked single ICI was nivolumab (SUCRA 67%), while the worst-ranked single ICI was atezolizumab (SUCRA 16%). Atezolizumab was associated with an estimated absolute event rate of 267 serious nephritis episodes per 10,000 patients (95% CI 11 to 7532).

For pancreatitis, the best-ranked single ICI was durvalumab (SUCRA 58%), while the worst-ranked single ICI was avelumab (SUCRA 9%). Avelumab was associated with an estimated absolute event rate of 204 serious pancreatitis episodes per 10,000 patients (95% CI 9 to 7571).

### Individual safety profiles

**Fig. 2:**
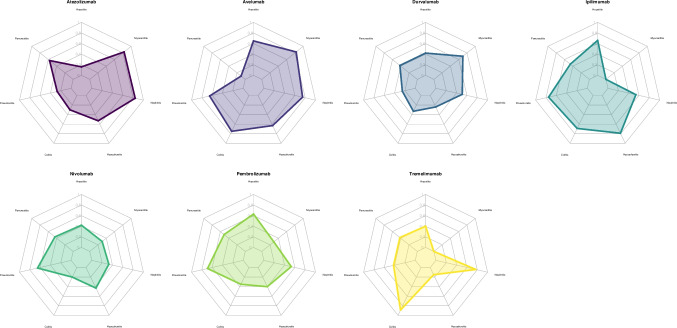
Spider plots. Spider plots of each single ICI in relation to the treatment rank (1-SUCRA) of each immune-related serious adverse event. Greater values indicate a greater likelihood of immune-related serious adverse events

Figure 2 shows the individual safety profiles for each ICI in terms of each outcome. No serious myocarditis events were reported in any ipilimumab or tremelimumab trial, despite being shown in Fig. 2.

## Discussion

We conducted a network meta-analysis of 96 trials with a combined 52,811 participants. To our knowledge, this is the largest network meta-analysis to date on ICI safety. We included all available ICI trials to assess their safety profile regarding immune-related serious adverse events. We analyzed seven irSAEs based on their clinical importance, namely, pneumonitis, colitis, hepatitis, hypophysitis, myocarditis, nephritis, and pancreatitis, all of which have a major role in the management of cancer patients, either by directly increasing morbidity and mortality [15] or by motivating treatment suspension. This is further underlined as this review assesses serious adverse events, not events as a whole. By pooling events across cancer types, this review essentially assumes a tumor-agnostic approach, which is in line with current thinking in relation to ICIs.

Based on a network meta-analysis conducted by Xu and colleagues [8] and the most recent ESMO Immuno-Oncology Handbook [16], both published in 2018, there is evidence that the risk of irAEs is greater with ICI combinations. Xu et al. analyzed the overall safety profile of different ICIs, concluding that the ranks are, from best to worst, atezolizumab, nivolumab, pembrolizumab, ipilimumab, and tremelimumab. Regarding the risk of irSAEs, our study had similar results, but it suggests a new rank, from best to worst: atezolizumab, durvalumab, pembrolizumab, nivolumab, avelumab, tremelimumab, and, finally, ipilimumab. Even though our results showed that atezolizumab has the lowest risk of overall irSAEs, each treatment has a unique safety profile.

We found that the absolute frequency of overall irSAEs is between 1/100 and 1/10, which can be interpreted as common [17]. Regarding the specific irSAEs analyzed, their frequency varied between uncommon and common. The worst-ranked single ICI, ipilimumab, had a notably high event rate for overall irSAEs that can be interpreted as between common and very common (potentially > 1/10). Regarding other single ICIs, both nivolumab and pembrolizumab have comparable event rates that can be interpreted as common (between 1/100 and 1/10), notably lower than ipilimumab.

Conventional therapy was uniformly the best-ranked treatment modality. However, when analyzing the rate of overall irSAEs in the conventional therapy group, we estimated an absolute frequency of 67 events per 10,000 patients. Although this event rate can be classified as uncommon, it is greater than expected since immune-related toxicity is not expected with non-ICIs. This may be attributable to the baseline rate of these events, though it may also reveal imprecision in categorizing events as immune-related within clinical trials.

Consistent with the distinct actions of immune checkpoints, the risk of certain immune-related events differs depending on the targeted pathway [4]. CTLA-4 inhibition non-specifically expands T cell clones, reduces Treg proliferation, and stimulates B cell activity, leading to persistence of high-frequency peripheral blood clonotypes. In contrast, PD-1/PD-L1 inhibition promotes a more focused oligoclonal expansion of T cell clones at the tumor site [4, 7]. Thus, CTLA-4 blockade might induce a greater magnitude of T cell proliferation than PD-1/PD-L1 blockade [4, 6, 7]. In this review, anti-CTLA-4 agents and ICI combinations were the worst-ranked treatment modalities across all outcomes, which corroborates our mechanistic understanding of these events.

Previous evidence has suggested that the treatment class most associated with hypophysitis and colitis is anti-CTLA-4 [4]. Our results were consistent with this understanding for serious hypophysitis. However, unexpectedly, tremelimumab, an anti-CTLA-4, was the best-ranked intervention regarding hypophysitis. Regarding serious colitis, we found that ICI combination was the worst-ranked treatment modality and anti-CTLA-4 agents were the second-worst ranked treatment modality.

The anti-PD-L1 treatment class is often associated with pneumonitis [4]. However, contrary to what was previously believed, for serious pneumonitis, we found a larger risk among patients treated with anti-CTLA-4 agents and ICI combinations, than with those treated with anti-PD-L1. Moreover, ipilimumab was the worst-ranked single intervention.

Myocarditis is thought to be more associated with ICI combinations [16], which is consistent with our results. However, for serious myocarditis, we found that atezolizumab was the worst-ranked treatment modality. For serious nephritis, our study adds new evidence by reinforcing anti-CTLA-4 as the worst treatment class and atezolizumab as the worst single ICI. For serious hepatitis, the worst-ranked individual interventions were ICI combinations with ipilimumab and ipilimumab alone. This is somewhat contrary to previous evidence, which suggested that there is a smaller risk of hepatitis with ipilimumab [16].

Overall, our findings demonstrate that each individual ICI has a specific safety profile which should be considered in patient care. For example, atezolizumab, the ICI with the lowest risk of overall irSAEs, has a low risk of pneumonitis, hepatitis, and colitis, despite having a notably high risk of myocarditis and nephritis. Similarly, avelumab was found to be one of the more toxic ICIs, despite having a very low risk of pancreatitis. Understanding the individuality of each ICI is of paramount importance.

### Limitations

We combined all available evidence independently of the cancer type, grouping different clinical situations, from cancer stages to different treatment duration and different populations. Overall, however, the statistical heterogeneity across analyses was low, and we did not find evidence of inconsistency. This choice is validated by evidence that the types of irAEs do not seem to be specific to the type of cancer [18], and therefore, it is pertinent to look at each ICI individual risk profile, independently of cancer type.

We analyzed only seven irSAEs. This is a twofold limitation. Firstly, we considered only serious adverse events and not the more frequent non-serious adverse events. Secondly, other events could have been chosen. Our choices were clinically based, since these events increase morbidity and mortality [15] and may motivate treatment discontinuations.

Lastly, when a patient experiences more than one irSAE, calculating estimates for total adverse events by simply summing them up may lead to an overestimation of the overall number of participants affects by irSAEs. However, given the rarity of the specific adverse events, we believe that the potential for overestimation is minimal and does not substantially impact the overall findings and conclusions of our results.

## Conclusions

ICIs remain relatively novel agents, and immune-related events continue to pose diagnostic and therapeutic challenges for clinicians. In the future, the evidence will continue to inform our understanding of ICI safety.

This review expands the knowledge on each ICI’s safety profile, thus providing evidence that might clarify which immune-related serious events to expect from each individual treatment. By making these events easier to predict, their management might therefore be improved.

### Supplementary Information

Below is the link to the electronic supplementary material.

Supplementary file1 (DOCX 6076 KB)
