# Correction to: Immune‑related serious adverse events with immune checkpoint inhibitors: systematic review and network meta‑analysis

**DOI:** 10.1007/s00228-024-03732-3

**Published:** 2024-07-24

**Authors:** Clara Oliveira, Beatrice Mainoli, Gonçalo S. Duarte, Tiago Machado, Rita G. Tinoco, Miguel Esperança‑Martins, Joaquim J. Ferreira, João Costa

**Affiliations:** 1 Laboratório de Farmacologia Clínica e Terapêutica, Faculdade de Medicina, Universidade de Lisboa, Avenida Professor Egas Moniz, 1649‑028 Lisbon, Portugal; 2 Instituto de Medicina Molecular João Lobo Antunes, Faculdade de Medicina, Universidade de Lisboa, Lisbon, Portugal; 3 Clinical Research Unit, Research Center of IPO Porto (CI-IPOP)/RISE@CI-IPOP (Health Research Network), Portuguese Oncology Institute of Porto (IPO Porto)/Porto Comprehensive Cancer Center (Porto.CCC), Porto, Portugal; 4 Clinical Pharmacology Department, Hospital da Luz Lisboa, Lisbon, Portugal; 5 Departamento Médico Grunenthal, Lisbon, SA Portugal; 6 Department of Medical Oncology, Centro Hospitalar Universitário Lisboa Norte, Hospital Santa Maria, Lisbon, Portugal; 7 CNS–Campus Neurológico Sénior, Torres Vedras, Portugal; 8 Centro de Estudos de Medicina Baseada na Evidência, Faculdade de Medicina, Universidade de Lisboa, Lisbon, Portugal

**Correction to: European Journal of Clinical Pharmacology (2024) 80:677–684** 10.1007/s00228-024-03647-z

The image of Fig. a contains unconverted text characters.
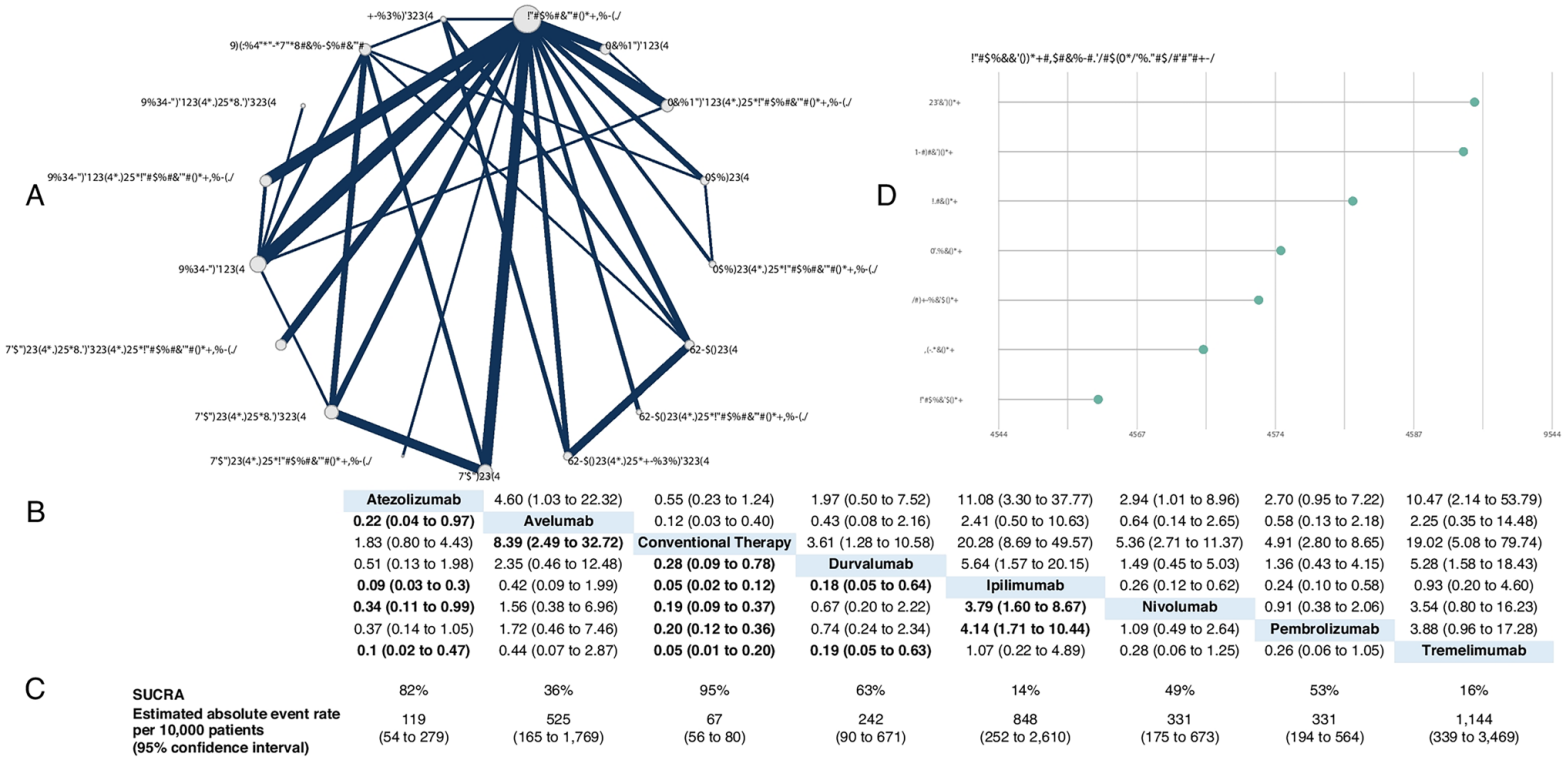


The correct image of Fig. b is shown below:
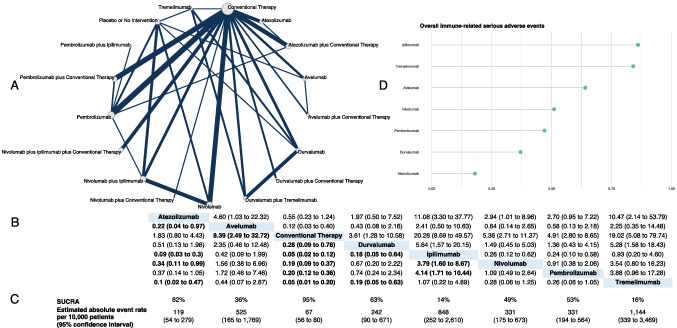


The original article has been corrected.

