# Supplementary material for: Immune-related serious adverse events with immune checkpoint inhibitors: Systematic review and network meta-analysis
Source: Eur J Clin Pharmacol. 2024 Feb 19;80(5):677–84. doi: 10.1007/s00228-024-03647-z (PMC11001692; doi:10.1007/s00228-024-03647-z)
Supplement: Supplementary file 1 — Supplementary file1 (DOCX 6076 KB) [file 228_2024_3647_MOESM1_ESM.docx]

**Supplementary Material**

*Immune-related serious adverse events with immune checkpoint inhibitors: systematic review and network meta-analysis.*

Clara Oliveira, Beatrice Mainoli, Gonçalo S Duarte, Rita G Tinoco, Tiago Machado, Miguel E Martins, Joaquim J Ferreira, João Costa.

**Corresponding author:** João Costa, MD, PhD

Laboratório de Farmacologia Clínica e Terapêutica, Faculdade de Medicina da Universidade de Lisboa, Av. Prof. Egas Moniz, Lisboa, 1649-028, Portugal.

**E-mail:** jcosta.fml@gmail.com
**Phone number:** (+351) 21 797 34 53; **Fax number:** (+351) 21 781 96 88.

Table of contents

[Appendix A.1. Search strategy 1](#_Toc156746904)

[Appendix B.1. Statistical methods 1](#_Toc156746905)

[Figure B.1. Flowchart of study selection 1](#_Toc156746906)

[Appendix B.2. List of excluded trials 1](#_Toc156746907)

[Table B.1. Included trials 1](#_Toc156746908)

[Table B.2. Characteristics of included trials 1](#_Toc156746909)

[Table B.2.1. Clinical and methodological characteristics of included trials. 1](#_Toc156746910)

[Table B.2.2. Characterization of drug interventions. 1](#_Toc156746911)

[Table B.2.3. Patients per cancer type, trial characteristics. 1](#_Toc156746912)

[Table B.3. Risk of bias assessment 1](#_Toc156746913)

[Figure B.2. Network plots based on individual treatments 1](#_Toc156746914)

[Figure B.2.1. Network plot for individual treatments, pneumonitis 1](#_Toc156746915)

[Figure B.2.2. Network plot for individual treatments, colitis 1](#_Toc156746916)

[Figure B.2.3. Network plot for individual treatments, hepatitis 1](#_Toc156746917)

[Figure B.2.4. Network plot for individual treatments, hypophysitis 1](#_Toc156746918)

[Figure B.3. Network plots based on treatment modalities 1](#_Toc156746919)

[Figure B.3.1. Network plot for treatment modalities, overall immune-related serious adverse events. 1](#_Toc156746920)

[Figure B.3.2. Network plot for treatment modalities, pneumonitis. 1](#_Toc156746921)

[Figure B.3.3. Network plot, treatment modalities, myocarditis. 1](#_Toc156746922)

[Figure B.3.4. Network plot, treatment modalities, colitis. 1](#_Toc156746923)

[Figure B.3.5. Network plot, treatment modalities, nephritis 1](#_Toc156746924)

[Figure B.3.6. Network plot, treatment modalities, pancreatitis 1](#_Toc156746925)

[Figure B.3.7. Network plot, treatment modalities, hepatitis. 1](#_Toc156746926)

[Figure B.3.8. Network plot, treatment modalities, hypophysitis. 1](#_Toc156746927)

[Table B.4. Model fit for each outcome 1](#_Toc156746928)

[Table B.4.1. Network meta-analysis models, individual interventions, overall immune-related serious adverse events. 1](#_Toc156746929)

[Table B.4.2. Network meta-analysis models, individual interventions, pneumonitis. 1](#_Toc156746930)

[Table B.4.3. Network meta-analysis models, individual interventions, myocarditis. 1](#_Toc156746931)

[Table B.4.4. Network meta-analysis models, individual interventions, colitis. 1](#_Toc156746932)

[Table B.4.5. Network meta-analysis models, individual interventions, nephritis. 1](#_Toc156746933)

[Table B.4.6. Network meta-analysis models, individual interventions, pancreatitis. 1](#_Toc156746934)

[Table B.4.7. Network meta-analysis models, individual interventions, hepatitis. 1](#_Toc156746935)

[Table B.4.8. Network meta-analysis models, individual interventions, hypophysitis. 1](#_Toc156746936)

[Figure B.4. Pairwise meta-analyses 1](#_Toc156746937)

[Figure B.4.1. Pairwise meta-analysis, overall immune-related serious adverse events 1](#_Toc156746938)

[Figure B.4.2. Pairwise meta-analysis, pneumonitis. 1](#_Toc156746939)

[Figure B.4.3. Pairwise meta-analyses, myocarditis. 1](#_Toc156746940)

[Figure B.4.4. Pairwise meta-analyses, colitis. 1](#_Toc156746941)

[Figure B.4.5. Pairwise meta-analyses, nephritis. 1](#_Toc156746942)

[Figure B.4.6. Pairwise meta-analyses, pancreatitis. 1](#_Toc156746943)

[Figure B.4.7. Pairwise meta-analyses, hepatitis. 1](#_Toc156746944)

[Figure B.4.8. Pairwise meta-analyses, hypophysitis. 1](#_Toc156746945)

[Figure B.5. Nodesplit figures 1](#_Toc156746946)

[Figure B.5.1. Nodesplit figure, overall immune-related serious adverse events 1](#_Toc156746947)

[Figure B.5.2. Nodesplit figure, pneumonitis. 1](#_Toc156746948)

[Figure B.5.3. Nodesplit figure, myocarditis. 1](#_Toc156746949)

[Figure B.5.4. Nodesplit figure, colitis. 1](#_Toc156746950)

[Figure B.5.5. Nodesplit figure, nephritis. 1](#_Toc156746951)

[Figure B.5.6. Nodesplit figure, pancreatitis. 1](#_Toc156746952)

[Figure B.5.7. Nodesplit figure, hepatitis. 1](#_Toc156746953)

[Figure B.5.8. Nodesplit figure, hypophysitis. 1](#_Toc156746954)

[Table B.5. Meta regression models and model fit according to treatment modalities 1](#_Toc156746955)

[Table B.5.1. Network meta-analysis regression models, treatment modalities, overall immune-related serious adverse events. 1](#_Toc156746956)

[Table B.5.2. Network meta-analysis regression models, treatment modalities, pneumonitis. 1](#_Toc156746957)

[Table B.5.3. Network meta-analysis models, treatment modalities, myocarditis. 1](#_Toc156746958)

[Table B.5.4. Network meta-analysis regression models, treatment modalities, colitis. 1](#_Toc156746959)

[Table B.5.5. Network meta-analysis models, treatment modalities, nephritis. 1](#_Toc156746960)

[Table B.5.6. Network meta-analysis models, treatment modalities, pancreatitis. 1](#_Toc156746961)

[Table B.5.7. Network meta-analysis regression models, treatment modalities, hepatitis. 1](#_Toc156746962)

[Table B.5.8. Network meta-analysis regression models, treatment modalities, hypophysitis. 1](#_Toc156746963)

[Table B.6. Assessment of publication bias 1](#_Toc156746964)

[Table B.7. Network meta-analysis results 1](#_Toc156746965)

[Table B.7.1. League table, overall immune-related serious adverse events. 1](#_Toc156746966)

[Table B.7.2. Estimated absolute event rate, overall immune-related serious adverse events. 1](#_Toc156746967)

[Table B.7.3. League table, pneumonitis. 1](#_Toc156746968)

[Table B.7.4. Estimated absolute event rate, pneumonitis. 1](#_Toc156746969)

[Table B.7.5. League table, myocarditis. 1](#_Toc156746970)

[Table B.7.6. Anticipated absolute event rate, myocarditis. 1](#_Toc156746971)

[Table B.7.8. League table, colitis. 1](#_Toc156746972)

[Table B.7.9. Anticipated absolute event rate, colitis. 1](#_Toc156746973)

[Table B.7.10. League table, nephritis. 1](#_Toc156746974)

[Table B.7.11. Estimated absolute event rate, nephritis. 1](#_Toc156746975)

[Table B.7.12. League table, pancreatitis. 1](#_Toc156746976)

[Table B.7.13. Estimated absolute event rate, pancreatitis. 1](#_Toc156746977)

[Table B.7.14. League table, hepatitis. 1](#_Toc156746978)

[Table B.7.15. Estimated absolute event rate, hepatitis. 1](#_Toc156746979)

[Table B.7.16. League table, hypophysitis. 1](#_Toc156746980)

[Table B.7.17. Estimated absolute event rate, hypophysitis. 1](#_Toc156746981)

[Table B.8. Individual interventions ranks (SUCRA) 1](#_Toc156746982)

[Table B.8.1. Ranks, individual interventions, overall immune-related serious adverse events. 1](#_Toc156746983)

[Table B.8.2. Ranks, individual interventions, pneumonitis. 1](#_Toc156746984)

[Table B.8.3. Ranks, individual interventions, myocarditis. 1](#_Toc156746985)

[Table B.8.4. Ranks, individual interventions, colitis. 1](#_Toc156746986)

[Table B.8.5. Ranks, individual interventions, nephritis. 1](#_Toc156746987)

[Table B.8.6. Ranks, individual interventions, pancreatitis. 1](#_Toc156746988)

[Table B.8.7. Ranks, individual interventions, hepatitis. 1](#_Toc156746989)

[Table B.8.8. Ranks, individual interventions, hypophysitis. 1](#_Toc156746990)

## Appendix A.1. Search strategy

**OVID Search strategy**

1. Ipilimumab.ab,kf,ti.

2. Tremelimumab.ab,kf,ti.

3. Pidilizumab.ti,kf,ti.

4. Yervoy.ab,kf,ti.

5. Opdivo.ab,kf,ti.

6. Nivolumab.ab,kf,ti.

7. Pembrolizumab.ab,kf,ti.

8. Keytruda.ab,kf,ti.

9. Tecentriq.ab,kf,ti.

10. Atezolizumab.ab,kf,ti.

11. Durvalumab.ab,kf,ti.

12. Imfinzi.ab,kf,ti.

13. Avelumab.ab,kf,ti.

14. Bavencio.ab,kf,ti.

15. Cemiplimab.ab,kf,ti.

16. Libtayo.ab,kf,ti.

17. Jemperli.ab,kf,ti.

18. Dostarlimab.ab,kf,ti.

19. or/1-18

20. (cancer* or neoplas* or tumor* or tumour* or carcinoma* or metasta* or leukemi* or leukaemi* or lymphoma*or myeloma* or sarcoma* or melanoma*).ab,kf,ti.

21. (randomized controlled trial or controlled clinical trial).pt. or randomized.ab. or placebo.ab. or clinical trials as topic.sh. or randomly.ab. or trial.ti.

22. exp animals/ not humans.sh.

23. 21 not 22

24. 19 and 20 and 23

25. remove duplicates from 24

**ClinicalTrials.gov Search Strategy**

AREA[ResultsFirstSubmitDate] NOT MISSING AND AREA[StudyType] EXPAND[Term] COVER[FullMatch] "Interventional" AND AREA[InterventionSearch] ( Ipilimumab OR Tremelimumab OR Pidilizumab OR Nivolumab OR Pembrolizumab OR Atezolizumab OR Durvalumab OR Avelumab OR Cemiplimab OR Dostarlimab )

## Appendix B.1. Statistical methods

All outcomes were dichotomous, and therefore we analyzed these data based on the number of events and the number of participants at risk in the intervention arms. We used these data to calculate the odds ratio (OR), where an OR lower than one corresponds to a safety benefit associated with the control group.

**Network meta-analysis**

We conducted NMAs of clinical trials to compare all interventions, namely ICIs, conventional therapy, placebo, and their combinations, in patients with any type of cancer. We used a Markov Chain Monte Carlo method in R using the *gemtc* package.(1) We analyzed data using log ORs and used a binomial likelihood and cloglog link.

**Prior distributions**

For all models, vague prior distributions were used for all trial baselines and for relative treatment or class effects (normal (0,100^2^)). For random treatment effects models, a minimally informative uniform prior distribution was used for the between-study heterogeneity parameter. For exchangeable-class models, a uniform (0, 5) prior distribution was used for the within-class standard deviation.(2)

Where the number of studies per comparison is small (usually less than 5), empirically informative prior distributions for the heterogeneity parameter are recommended.(3,4) Therefore, we conducted these analyses using the empirically estimated meta-epidemiological distributions log normal (-3.23, 1.79).(3,4)

**Model fit and choice**

We chose a model and considering it as the primary analysis for NMA using the following strategy:

1. Begin with consistency models (with random and fixed treatment effects). If both fit well (i.e., posterior mean of residual deviance is close to the number of data points), choose the model with the lowest deviance information criterion (DIC) (if the difference is less than 3, choose the fixed effect model) and stop. In this review we did not proceed past this point.
2. If the fixed treatment effect-fixed class model does not fit well, try the fixed treatment effect-random class model – assess fit, compare to models in the first step here, and choose the model with the lowest DIC.
3. If neither of the models in the first or second step fit well, try also random treatment effects with random class model. Choose a final model based on DIC but interpret with caution if model fit is poor.
4. Compare results of random class models to the equivalent treatment level model (i.e., no class), if networks are connected.

**Unit of analysis issues**

Our unit of analysis is study-level data, preferability from intention-to-treat analyses. Participants were used as the unit of analysis to eliminate the risk of multiple participants counting (i.e., number of participants with at least one event).

**Dealing with missing data**

Where data were missing, we used the available information (e.g., standard error, 95% confidence interval, or exact P value) to algebraically recover the missing data.(5)

**Assessment of heterogeneity**

**Pairwise meta-analysis**

We used I^2^ to measure heterogeneity between trials within comparisons.(6)

**Assessment of transitivity**

We summarized the key clinical and methodological characteristics that could potentially modify the treatment effects across trials. We tabulated and presented them to compare the characteristics and identify possible sources of clinical and methodological heterogeneity.

**Assessment of inconsistency and statistical consistency**

We assessed consistency by comparing the model fit and between-trial heterogeneity from NMA models versus those from an unrelated mean effects (inconsistency) model.(7) This was used as an omnibus test for inconsistency. We further created nodesplit models for each outcome.

**Assessment of reporting biases**

**Pairwise meta-analysis**

We assessed the possibility of reporting bias through visual inspection of funnel plot asymmetry, and Peter’s test, provided that 10 or more studies per outcome were available.(8)

**Network meta-analysis**

We aimed to minimize reporting bias from unpublished trials or selective outcome reporting by using a broad search strategy, and by checking references of included trials and relevant systematic reviews. For each outcome, we estimated and presented the proportion of trials that contributed to the NMA.

**Data synthesis**

We performed statistical analysis using R (version 4.0.5).

**Pairwise meta‐analysis**

We pooled data in situations where two or more trials provided data for the same comparison by applying the Sidik-Jonkman method.(9,10) We conducted data synthesis using a random‐effects model by default, independently of the presence or lack of considerable statistical heterogeneity, owing to the variety of disease subtypes that we intended to analyze.

**Network meta-analysis**

For each outcome, we constructed a network diagram to display the treatment comparisons for which direct evidence was available. We used this network diagram to examine the symmetry and geometry of the data, with node sizes corresponding to the number of participants receiving an intervention and connection sizes corresponding to the number of trials within a given comparison. When performing a network meta-analysis, we relied on the assumptions of transitivity (i.e., if drug B is superior to drug A, and drug C is superior to drug B, it is assumed that drug C is superior to drug A).(2)

We performed all analyses using 100,000 iterations after a burn-in of 10,000. To assess model convergence, we assessed Gelman-Rubin-Brooks plots and the potential scale reduction factors. In case of convergence, the potential scale reduction factor should gradually shrink down to zero with increasing numbers of interactions and should at least be below 1.05 in the end.(11) To rank the treatments for each outcome, we used the surface under the cumulative ranking curve (SUCRA).(12)

We synthetized our results by comparing the effect of each intervention with conventional therapy. Additionally, we assessed the absolute rate of each outcome, as rates per 10,000 patients. These estimates were calculated in accordance with the GRADE methodology.(13)

**Additional analyses**

We conducted meta-regressions for outcomes with at least 40 trials to test for the impact of possible confounders. We tested the impact of arm-level overall survival, arm-level progression-free survival, overall trial-level risk of bias, and arm-level percentage of female participants.

## Figure B.1. Flowchart of study selection

**Identification of studies via databases and registers**

Records removed *before screening*:

Duplicate records removed

(n = 16)

Records identified from:

Databases (n = 1803)

Registers (n = 521)

**Identification**

Records screened

(n = 2308)

Records excluded

(n = 1749)

Reports sought for retrieval

(n = 559)

Reports not retrieved

(n = 16)

**Screening**

Reports excluded:

Population (n = 7)

Intervention (n = 10)

Comparator (n = 125)

Study design (n = 79)

Duplicate (n = 7)

No results (n=30)

No events of interest (n = 15)

Reports assessed for eligibility

(n = 543)

Studies included in review

(n = 96)

Reports of included studies

(n = 270)

**Included**

## Appendix B.2. List of excluded trials

**Population**

- A Randomized, Double-Blind, Placebo-Controlled Study of a Single Dose of Pembrolizumab in HIV-Infected Patients (Clinical Trial Registration No. NCT03367754). clinicaltrials.gov. Retrieved 13 December 2021, from https://clinicaltrials.gov/ct2/show/NCT03367754
- Reck, M., Mok, T. S. K., Nishio, M., Jotte, R. M., Cappuzzo, F., Orlandi, F., Stroyakovskiy, D., Nogami, N., Rodríguez-Abreu, D., Moro-Sibilot, D., Thomas, C. A., Barlesi, F., Finley, G., Lee, A., Coleman, S., Deng, Y., Kowanetz, M., Shankar, G., Lin, W., … Socinski, M. A. (2019). Atezolizumab plus bevacizumab and chemotherapy in non-small-cell lung cancer (IMpower150): Key subgroup analyses of patients with EGFR mutations or baseline liver metastases in a randomised, open-label phase 3 trial. The Lancet Respiratory Medicine, 7(5), 387–401. https://doi.org/10.1016/S2213-2600(19)30084-0
- Reilley, M. J., Bailey, A., Subbiah, V., Janku, F., Naing, A., Falchook, G., Karp, D., Piha-Paul, S., Tsimberidou, A., Fu, S., Lim, J., Bean, S., Bass, A., Montez, S., Vence, L., Sharma, P., Allison, J., Meric-Bernstam, F., & Hong, D. S. (2017). Phase I clinical trial of combination imatinib and ipilimumab in patients with advanced malignancies. Journal for ImmunoTherapy of Cancer, 5(1), 35. https://doi.org/10.1186/s40425-017-0238-1
- Schadendorf, D., Dummer, R., Hauschild, A., Robert, C., Hamid, O., Daud, A., van den Eertwegh, A., Cranmer, L., O’Day, S., Puzanov, I., Schachter, J., Blank, C., Salama, A., Loquai, C., Mehnert, J. M., Hille, D., Ebbinghaus, S., Kang, S. P., Zhou, W., & Ribas, A. (2016). Health-related quality of life in the randomised KEYNOTE-002 study of pembrolizumab versus chemotherapy in patients with ipilimumab-refractory melanoma. European Journal of Cancer, 67, 46–54. https://doi.org/10.1016/j.ejca.2016.07.018

**Intervention**

- A Pilot Study to Test the Feasibility and Immunologic Impact of Sipuleucel-T (Provenge) Administered With or Without Anti-PD-1 mAb (CT-011) and Low Dose Cyclophosphamide in Men With Advanced Castrate-Resistant Prostate Cancer (Clinical Trial Registration No. NCT01420965). clinicaltrials.gov. Retrieved 13 December 2021, from https://clinicaltrials.gov/ct2/show/NCT01420965
- Berman, D., Parker, S. M., Siegel, J., Chasalow, S. D., Weber, J., Galbraith, S., Targan, S. R., & Wang, H. L. (2010). Blockade of cytotoxic T-lymphocyte antigen-4 by ipilimumab results in dysregulation of gastrointestinal immunity in patients with advanced melanoma. Cancer Immunity, 10, 11.
- Pakkala, S., Higgins, K., Chen, Z., Sica, G., Steuer, C., Zhang, C., Zhang, G., Wang, S., Hossain, M. S., Nazha, B., Beardslee, T., Khuri, F. R., Curran, W., Lonial, S., Waller, E. K., Ramalingam, S., & Owonikoko, T. K. (2020). Durvalumab and tremelimumab with or without stereotactic body radiation therapy in relapsed small cell lung cancer: A randomized phase II study. Journal for ImmunoTherapy of Cancer, 8(2), e001302. https://doi.org/10.1136/jitc-2020-001302
- Schoenfeld, J. D., Hanna, G. J., Jo, V. Y., Rawal, B., Chen, Y.-H., Catalano, P. S., Lako, A., Ciantra, Z., Weirather, J. L., Criscitiello, S., Luoma, A., Chau, N., Lorch, J., Kass, J. I., Annino, D., Goguen, L., Desai, A., Ross, B., Shah, H. J., … Haddad, R. I. (2020). Neoadjuvant Nivolumab or Nivolumab Plus Ipilimumab in Untreated Oral Cavity Squamous Cell Carcinoma: A Phase 2 Open-Label Randomized Clinical Trial. JAMA Oncology, 6(10), 1563. https://doi.org/10.1001/jamaoncol.2020.2955
- Thibault, C., Elaidi, R., Vano, Y.-A., Rouabah, M., Braychenko, E., Helali, I., Audenet, F., & Oudard, S. (2020). Open-label phase II to evaluate the efficacy of NEoadjuvant dose-dense MVAC In cOmbination with durvalumab and tremelimumab in muscle-invasive urothelial carcinoma: NEMIO. Bulletin Du Cancer, 107(5), eS8–eS15. https://doi.org/10.1016/S0007-4551(20)30281-2
- Tintelnot, J., Goekkurt, E., Binder, M., Thuss-Patience, P., Lorenzen, S., Knorrenschild, J. R., Kretzschmar, A., Ettrich, T., Lindig, U., Jacobasch, L., Pink, D., Al-Batran, S.-E., Hinke, A., Hegewisch-Becker, S., Nilsson, S., Bokemeyer, C., & Stein, A. (2020). Ipilimumab or FOLFOX with Nivolumab and Trastuzumab in previously untreated HER2-positive locally advanced or metastatic EsophagoGastric Adenocarcinoma—The randomized phase 2 INTEGA trial (AIO STO 0217). BMC Cancer, 20(1), 503. https://doi.org/10.1186/s12885-020-06958-3
- Zhou, Q., Chen, M., Wu, G., Chang, J.-H., Jiang, O., Cui, J.-W., Han, G., Lin, Q., Fang, J., Chen, G.-Y., & Wu, Y.-L. (2020). GEMSTONE-301: A phase III clinical trial of CS1001 as consolidation therapy in patients with locally advanced/ unresectable (stage III) non-small cell lung cancer (NSCLC) who did not have disease progression after prior concurrent/sequential chemoradiotherapy. Translational Lung Cancer Research, 9(5), 2008–2015. https://doi.org/10.21037/tlcr-20-608

**Comparator**

- A Phase IIa, Open-Label, Multi-Center, Multi-Cohort, Immune-Modulated Study of Selected Small Molecules (Gefitinib, AZD9291, or Selumetinib + Docetaxel) or a 1st Immune-Mediated Therapy (IMT; Tremelimumab) With a Sequential Switch to a 2nd IMT (MEDI4736) in Patients With Locally Advanced or Metastatic Non-Small-Cell Lung Cancer (Stage IIIB-IV) (Clinical Trial Registration No. NCT02179671). clinicaltrials.gov. Retrieved 13 December 2021, from https://clinicaltrials.gov/ct2/show/NCT02179671
- Adra, N., Einhorn, L. H., Althouse, S. K., Ammakkanavar, N. R., Musapatika, D., Albany, C., Vaughn, D., & Hanna, N. H. (2018). Phase II trial of pembrolizumab in patients with platinum refractory germ-cell tumors: A Hoosier Cancer Research Network Study GU14-206. Annals of Oncology, 29(1), 209–214. https://doi.org/10.1093/annonc/mdx680
- Armand, P., Engert, A., Younes, A., Fanale, M., Santoro, A., Zinzani, P. L., Timmerman, J. M., Collins, G. P., Ramchandren, R., Cohen, J. B., De Boer, J. P., Kuruvilla, J., Savage, K. J., Trneny, M., Shipp, M. A., Kato, K., Sumbul, A., Farsaci, B., & Ansell, S. M. (2018). Nivolumab for Relapsed/Refractory Classic Hodgkin Lymphoma After Failure of Autologous Hematopoietic Cell Transplantation: Extended Follow-Up of the Multicohort Single-Arm Phase II CheckMate 205 Trial. Journal of Clinical Oncology, 36(14), 1428–1439. https://doi.org/10.1200/JCO.2017.76.0793
- Balar, A. V., Galsky, M. D., Rosenberg, J. E., Powles, T., Petrylak, D. P., Bellmunt, J., Loriot, Y., Necchi, A., Hoffman-Censits, J., Perez-Gracia, J. L., Dawson, N. A., van der Heijden, M. S., Dreicer, R., Srinivas, S., Retz, M. M., Joseph, R. W., Drakaki, A., Vaishampayan, U. N., Sridhar, S. S., … IMvigor210 Study Group. (2017). Atezolizumab as first-line treatment in cisplatin-ineligible patients with locally advanced and metastatic urothelial carcinoma: A single-arm, multicentre, phase 2 trial. Lancet (London, England), 389(10064), 67–76. https://doi.org/10.1016/S0140-6736(16)32455-2
- Bauml, J. M., Mick, R., Ciunci, C., Aggarwal, C., Davis, C., Evans, T., Deshpande, C., Miller, L., Patel, P., Alley, E., Knepley, C., Mutale, F., Cohen, R. B., & Langer, C. J. (2019). Pembrolizumab After Completion of Locally Ablative Therapy for Oligometastatic Non–Small Cell Lung Cancer: A Phase 2 Trial. JAMA Oncology, 5(9), 1283. https://doi.org/10.1001/jamaoncol.2019.1449
- Bochner, B. H. (2017). Re: Atezolizumab in Patients with Locally Advanced and Metastatic Urothelial Carcinoma who have Progressed Following Treatment with Platinum-based Chemotherapy: A Single-arm, Multicenter, Phase 2 Trial. European Urology, 71(2), 299–300. https://doi.org/10.1016/j.eururo.2016.10.012
- Brufsky, A., Kim, S. B., Zvirbule, Ž., Eniu, A., Mebis, J., Sohn, J. H., Wongchenko, M., Chohan, S., Amin, R., Yan, Y., McNally, V., Miles, D., & Loi, S. (2021). A phase II randomized trial of cobimetinib plus chemotherapy, with or without atezolizumab, as first-line treatment for patients with locally advanced or metastatic triple-negative breast cancer (COLET): Primary analysis. Annals of Oncology, 32(5), 652–660. https://doi.org/10.1016/j.annonc.2021.01.065
- Chatterjee, M., Turner, D. C., Felip, E., Lena, H., Cappuzzo, F., Horn, L., Garon, E. B., Hui, R., Arkenau, H.-T., Gubens, M. A., Hellmann, M. D., Dong, D., Li, C., Mayawala, K., Freshwater, T., Ahamadi, M., Stone, J., Lubiniecki, G. M., Zhang, J., … Fløtten, Ø. (2016). Systematic evaluation of pembrolizumab dosing in patients with advanced non-small-cell lung cancer. Annals of Oncology, 27(7), 1291–1298. https://doi.org/10.1093/annonc/mdw174
- Cho, J., Kim, H. S., Ku, B. M., Choi, Y.-L., Cristescu, R., Han, J., Sun, J.-M., Lee, S.-H., Ahn, J. S., Park, K., & Ahn, M.-J. (2019). Pembrolizumab for Patients With Refractory or Relapsed Thymic Epithelial Tumor: An Open-Label Phase II Trial. Journal of Clinical Oncology, 37(24), 2162–2170. https://doi.org/10.1200/JCO.2017.77.3184
- Chung, H. C., Arkenau, H.-T., Lee, J., Rha, S. Y., Oh, D.-Y., Wyrwicz, L., Kang, Y.-K., Lee, K.-W., Infante, J. R., Lee, S. S., Kemeny, M., Keilholz, U., Melichar, B., Mita, A., Plummer, R., Smith, D., Gelb, A. B., Xiong, H., Hong, J., … Safran, H. (2019). Avelumab (anti–PD-L1) as first-line switch-maintenance or second-line therapy in patients with advanced gastric or gastroesophageal junction cancer: Phase 1b results from the JAVELIN Solid Tumor trial. Journal for ImmunoTherapy of Cancer, 7(1), 30. https://doi.org/10.1186/s40425-019-0508-1
- Cloughesy, T. F., Mochizuki, A. Y., Orpilla, J. R., Hugo, W., Lee, A. H., Davidson, T. B., Wang, A. C., Ellingson, B. M., Rytlewski, J. A., Sanders, C. M., Kawaguchi, E. S., Du, L., Li, G., Yong, W. H., Gaffey, S. C., Cohen, A. L., Mellinghoff, I. K., Lee, E. Q., Reardon, D. A., … Prins, R. M. (2019). Neoadjuvant anti-PD-1 immunotherapy promotes a survival benefit with intratumoral and systemic immune responses in recurrent glioblastoma. Nature Medicine, 25(3), 477–486. https://doi.org/10.1038/s41591-018-0337-7
- Colevas, A. D., Bahleda, R., Braiteh, F., Balmanoukian, A., Brana, I., Chau, N. G., Sarkar, I., Molinero, L., Grossman, W., Kabbinavar, F., Fassò, M., O’Hear, C., & Powderly, J. (2018). Safety and clinical activity of atezolizumab in head and neck cancer: Results from a phase I trial. Annals of Oncology, 29(11), 2247–2253. https://doi.org/10.1093/annonc/mdy411
- Di Giacomo, A. M., Ascierto, P. A., Pilla, L., Santinami, M., Ferrucci, P. F., Giannarelli, D., Marasco, A., Rivoltini, L., Simeone, E., Nicoletti, S. V., Fonsatti, E., Annesi, D., Queirolo, P., Testori, A., Ridolfi, R., Parmiani, G., & Maio, M. (2012). Ipilimumab and fotemustine in patients with advanced melanoma (NIBIT-M1): An open-label, single-arm phase 2 trial. The Lancet Oncology, 13(9), 879–886. https://doi.org/10.1016/S1470-2045(12)70324-8
- Disis, M. L., Taylor, M. H., Kelly, K., Beck, J. T., Gordon, M., Moore, K. M., Patel, M. R., Chaves, J., Park, H., Mita, A. C., Hamilton, E. P., Annunziata, C. M., Grote, H. J., von Heydebreck, A., Grewal, J., Chand, V., & Gulley, J. L. (2019). Efficacy and Safety of Avelumab for Patients With Recurrent or Refractory Ovarian Cancer: Phase 1b Results From the JAVELIN Solid Tumor Trial. JAMA Oncology, 5(3), 393. https://doi.org/10.1001/jamaoncol.2018.6258
- Doi, T., Iwasa, S., Muro, K., Satoh, T., Hironaka, S., Esaki, T., Nishina, T., Hara, H., Machida, N., Komatsu, Y., Shimada, Y., Otsu, S., Shimizu, S., & Watanabe, M. (2019). Phase 1 trial of avelumab (anti-PD-L1) in Japanese patients with advanced solid tumors, including dose expansion in patients with gastric or gastroesophageal junction cancer: The JAVELIN Solid Tumor JPN trial. Gastric Cancer, 22(4), 817–827. https://doi.org/10.1007/s10120-018-0903-1
- Eichhorn, F., Klotz, L. V., Bischoff, H., Thomas, M., Lasitschka, F., Winter, H., Hoffmann, H., & Eichhorn, M. E. (2019). Neoadjuvant anti-programmed Death-1 immunotherapy by Pembrolizumab in resectable nodal positive stage II/IIIa non-small-cell lung cancer (NSCLC): The NEOMUN trial. BMC Cancer, 19(1), 413. https://doi.org/10.1186/s12885-019-5624-2
- Frenel, J.-S., Le Tourneau, C., O’Neil, B., Ott, P. A., Piha-Paul, S. A., Gomez-Roca, C., van Brummelen, E. M. J., Rugo, H. S., Thomas, S., Saraf, S., Rangwala, R., & Varga, A. (2017). Safety and Efficacy of Pembrolizumab in Advanced, Programmed Death Ligand 1–Positive Cervical Cancer: Results From the Phase Ib KEYNOTE-028 Trial. Journal of Clinical Oncology, 35(36), 4035–4041. https://doi.org/10.1200/JCO.2017.74.5471
- Fuchs, C. S., Doi, T., Jang, R. W., Muro, K., Satoh, T., Machado, M., Sun, W., Jalal, S. I., Shah, M. A., Metges, J.-P., Garrido, M., Golan, T., Mandala, M., Wainberg, Z. A., Catenacci, D. V., Ohtsu, A., Shitara, K., Geva, R., Bleeker, J., … Yoon, H. H. (2018). Safety and Efficacy of Pembrolizumab Monotherapy in Patients With Previously Treated Advanced Gastric and Gastroesophageal Junction Cancer: Phase 2 Clinical KEYNOTE-059 Trial. JAMA Oncology, 4(5), e180013. https://doi.org/10.1001/jamaoncol.2018.0013
- Fujimoto, D., Yomota, M., Sekine, A., Morita, M., Morimoto, T., Hosomi, Y., Ogura, T., Tomioka, H., & Tomii, K. (2019). Nivolumab for advanced non-small cell lung cancer patients with mild idiopathic interstitial pneumonia: A multicenter, open-label single-arm phase II trial. Lung Cancer, 134, 274–278. https://doi.org/10.1016/j.lungcan.2019.06.001
- Fumet, J.-D., Isambert, N., Hervieu, A., Zanetta, S., Guion, J.-F., Hennequin, A., Rederstorff, E., Bertaut, A., & Ghiringhelli, F. (2018). Phase Ib/II trial evaluating the safety, tolerability and immunological activity of durvalumab (MEDI4736) (anti-PD-L1) plus tremelimumab (anti-CTLA-4) combined with FOLFOX in patients with metastatic colorectal cancer. ESMO Open, 3(4), e000375. https://doi.org/10.1136/esmoopen-2018-000375
- Gangadhar, T. C., Hwu, W.-J., Postow, M. A., Hamid, O., Daud, A., Dronca, R., Joseph, R., O’Day, S. J., Hodi, F. S., Pavlick, A. C., Kluger, H., Oxborough, R. P., Yang, A., Gazdoiu, M., Kush, D. A., Ebbinghaus, S., & Salama, A. K. S. (2017). Efficacy and Safety of Pembrolizumab in Patients Enrolled in KEYNOTE-030 in the United States: An Expanded Access Program. Journal of Immunotherapy, 40(9), 334–340. https://doi.org/10.1097/CJI.0000000000000186
- George, S., Motzer, R. J., Hammers, H. J., Redman, B. G., Kuzel, T. M., Tykodi, S. S., Plimack, E. R., Jiang, J., Waxman, I. M., & Rini, B. I. (2016). Safety and Efficacy of Nivolumab in Patients With Metastatic Renal Cell Carcinoma Treated Beyond Progression: A Subgroup Analysis of a Randomized Clinical Trial. JAMA Oncology, 2(9), 1179. https://doi.org/10.1001/jamaoncol.2016.0775
- Gettinger, S. N., Horn, L., Gandhi, L., Spigel, D. R., Antonia, S. J., Rizvi, N. A., Powderly, J. D., Heist, R. S., Carvajal, R. D., Jackman, D. M., Sequist, L. V., Smith, D. C., Leming, P., Carbone, D. P., Pinder-Schenck, M. C., Topalian, S. L., Hodi, F. S., Sosman, J. A., Sznol, M., … Brahmer, J. R. (2015). Overall Survival and Long-Term Safety of Nivolumab (Anti–Programmed Death 1 Antibody, BMS-936558, ONO-4538) in Patients With Previously Treated Advanced Non–Small-Cell Lung Cancer. Journal of Clinical Oncology, 33(18), 2004–2012. https://doi.org/10.1200/JCO.2014.58.3708
- Haag, G. M., Zoernig, I., Hassel, J. C., Halama, N., Dick, J., Lang, N., Podola, L., Funk, J., Ziegelmeier, C., Juenger, S., Bucur, M., Umansky, L., Falk, C. S., Freitag, A., Karapanagiotou-Schenkel, I., Beckhove, P., Enk, A., & Jaeger, D. (2018). Phase II trial of ipilimumab in melanoma patients with preexisting humoural immune response to NY-ESO-1. European Journal of Cancer, 90, 122–129. https://doi.org/10.1016/j.ejca.2017.12.001
- Hassan, R., Thomas, A., Nemunaitis, J. J., Patel, M. R., Bennouna, J., Chen, F. L., Delord, J.-P., Dowlati, A., Kochuparambil, S. T., Taylor, M. H., Powderly, J. D., Vaishampayan, U. N., Verschraegen, C., Grote, H. J., von Heydebreck, A., Chin, K., & Gulley, J. L. (2019). Efficacy and Safety of Avelumab Treatment in Patients With Advanced Unresectable Mesothelioma: Phase 1b Results From the JAVELIN Solid Tumor Trial. JAMA Oncology, 5(3), 351. https://doi.org/10.1001/jamaoncol.2018.5428
- Hodi, F. S., Lee, S., McDermott, D. F., Rao, U. N., Butterfield, L. H., Tarhini, A. A., Leming, P., Puzanov, I., Shin, D., & Kirkwood, J. M. (2014). Ipilimumab Plus Sargramostim vs Ipilimumab Alone for Treatment of Metastatic Melanoma: A Randomized Clinical Trial. JAMA, 312(17), 1744. https://doi.org/10.1001/jama.2014.13943
- Kaufman, H. L., Russell, J., Hamid, O., Bhatia, S., Terheyden, P., D’Angelo, S. P., Shih, K. C., Lebbé, C., Linette, G. P., Milella, M., Brownell, I., Lewis, K. D., Lorch, J. H., Chin, K., Mahnke, L., von Heydebreck, A., Cuillerot, J.-M., & Nghiem, P. (2016). Avelumab in patients with chemotherapy-refractory metastatic Merkel cell carcinoma: A multicentre, single-group, open-label, phase 2 trial. The Lancet Oncology, 17(10), 1374–1385. https://doi.org/10.1016/S1470-2045(16)30364-3
- Kaufman, H. L., Russell, J. S., Hamid, O., Bhatia, S., Terheyden, P., D’Angelo, S. P., Shih, K. C., Lebbé, C., Milella, M., Brownell, I., Lewis, K. D., Lorch, J. H., von Heydebreck, A., Hennessy, M., & Nghiem, P. (2018). Updated efficacy of avelumab in patients with previously treated metastatic Merkel cell carcinoma after ≥1 year of follow-up: JAVELIN Merkel 200, a phase 2 clinical trial. Journal for ImmunoTherapy of Cancer, 6(1), 7. https://doi.org/10.1186/s40425-017-0310-x
- Keilholz, U., Mehnert, J. M., Bauer, S., Bourgeois, H., Patel, M. R., Gravenor, D., Nemunaitis, J. J., Taylor, M. H., Wyrwicz, L., Lee, K.-W., Kasturi, V., Chin, K., von Heydebreck, A., & Gulley, J. L. (2019). Avelumab in patients with previously treated metastatic melanoma: Phase 1b results from the JAVELIN Solid Tumor trial. Journal for ImmunoTherapy of Cancer, 7(1), 12. https://doi.org/10.1186/s40425-018-0459-y
- Kluger, H. M., Chiang, V., Mahajan, A., Zito, C. R., Sznol, M., Tran, T., Weiss, S. A., Cohen, J. V., Yu, J., Hegde, U., Perrotti, E., Anderson, G., Ralabate, A., Kluger, Y., Wei, W., Goldberg, S. B., & Jilaveanu, L. B. (2019). Long-Term Survival of Patients With Melanoma With Active Brain Metastases Treated With Pembrolizumab on a Phase II Trial. Journal of Clinical Oncology, 37(1), 52–60. https://doi.org/10.1200/JCO.18.00204
- Lawrence, D. P., Hamid, O., McDermott, D. F., Puzanov, I., Sznol, M., Clark, J., Logan, T., Hodi, F. S., Heller, K. N., & Margolin, K. A. (2010). Phase II trial of ipilimumab monotherapy in melanoma patients with brain metastases. Journal of Clinical Oncology, 28(15_suppl), 8523–8523. https://doi.org/10.1200/jco.2010.28.15_suppl.8523
- Le, D. T., Lutz, E., Uram, J. N., Sugar, E. A., Onners, B., Solt, S., Zheng, L., Diaz, L. A., Donehower, R. C., Jaffee, E. M., & Laheru, D. A. (2013). Evaluation of Ipilimumab in Combination With Allogeneic Pancreatic Tumor Cells Transfected With a GM-CSF Gene in Previously Treated Pancreatic Cancer. Journal of Immunotherapy, 36(7), 382–389. https://doi.org/10.1097/CJI.0b013e31829fb7a2
- Leighl, N. B., Hellmann, M. D., Hui, R., Carcereny, E., Felip, E., Ahn, M.-J., Eder, J. P., Balmanoukian, A. S., Aggarwal, C., Horn, L., Patnaik, A., Gubens, M., Ramalingam, S. S., Lubiniecki, G. M., Zhang, J., Piperdi, B., & Garon, E. B. (2019). Pembrolizumab in patients with advanced non-small-cell lung cancer (KEYNOTE-001): 3-year results from an open-label, phase 1 study. The Lancet Respiratory Medicine, 7(4), 347–357. https://doi.org/10.1016/S2213-2600(18)30500-9
- Loi, S., Giobbie-Hurder, A., Gombos, A., Bachelot, T., Hui, R., Curigliano, G., Campone, M., Biganzoli, L., Bonnefoi, H., Jerusalem, G., Bartsch, R., Rabaglio-Poretti, M., Kammler, R., Maibach, R., Smyth, M. J., Di Leo, A., Colleoni, M., Viale, G., Regan, M. M., … Schneier, P. (2019). Pembrolizumab plus trastuzumab in trastuzumab-resistant, advanced, HER2-positive breast cancer (PANACEA): A single-arm, multicentre, phase 1b–2 trial. The Lancet Oncology, 20(3), 371–382. https://doi.org/10.1016/S1470-2045(18)30812-X
- Luke, J. J., Donahue, H., Nishino, M., Giobbie-Hurder, A., Davis, M., Bailey, N., Ott, P. A., & Hodi, F. S. (2015). Single Institution Experience of Ipilimumab 3 mg/kg with Sargramostim (GM-CSF) in Metastatic Melanoma. Cancer Immunology Research, 3(9), 986–991. https://doi.org/10.1158/2326-6066.CIR-15-0066
- Margolin, K., Ernstoff, M. S., Hamid, O., Lawrence, D., McDermott, D., Puzanov, I., Wolchok, J. D., Clark, J. I., Sznol, M., Logan, T. F., Richards, J., Michener, T., Balogh, A., Heller, K. N., & Hodi, F. S. (2012). Ipilimumab in patients with melanoma and brain metastases: An open-label, phase 2 trial. The Lancet Oncology, 13(5), 459–465. https://doi.org/10.1016/S1470-2045(12)70090-6
- Massarelli, E., William, W., Johnson, F., Kies, M., Ferrarotto, R., Guo, M., Feng, L., Lee, J. J., Tran, H., Kim, Y. U., Haymaker, C., Bernatchez, C., Curran, M., Zecchini Barrese, T., Rodriguez Canales, J., Wistuba, I., Li, L., Wang, J., van der Burg, S. H., … Glisson, B. (2019). Combining Immune Checkpoint Blockade and Tumor-Specific Vaccine for Patients With Incurable Human Papillomavirus 16–Related Cancer: A Phase 2 Clinical Trial. JAMA Oncology, 5(1), 67. https://doi.org/10.1001/jamaoncol.2018.4051
- McDermott, D. F., Drake, C. G., Sznol, M., Choueiri, T. K., Powderly, J. D., Smith, D. C., Brahmer, J. R., Carvajal, R. D., Hammers, H. J., Puzanov, I., Hodi, F. S., Kluger, H. M., Topalian, S. L., Pardoll, D. M., Wigginton, J. M., Kollia, G. D., Gupta, A., McDonald, D., Sankar, V., … Atkins, M. B. (2015). Survival, Durable Response, and Long-Term Safety in Patients With Previously Treated Advanced Renal Cell Carcinoma Receiving Nivolumab. Journal of Clinical Oncology, 33(18), 2013–2020. https://doi.org/10.1200/JCO.2014.58.1041
- Mitchell, T. C., Hamid, O., Smith, D. C., Bauer, T. M., Wasser, J. S., Olszanski, A. J., Luke, J. J., Balmanoukian, A. S., Schmidt, E. V., Zhao, Y., Gong, X., Maleski, J., Leopold, L., & Gajewski, T. F. (2018). Epacadostat Plus Pembrolizumab in Patients With Advanced Solid Tumors: Phase I Results From a Multicenter, Open-Label Phase I/II Trial (ECHO-202/KEYNOTE-037). Journal of Clinical Oncology, 36(32), 3223–3230. https://doi.org/10.1200/JCO.2018.78.9602
- Motzer, R. J., Rini, B. I., McDermott, D. F., Redman, B. G., Kuzel, T. M., Harrison, M. R., Vaishampayan, U. N., Drabkin, H. A., George, S., Logan, T. F., Margolin, K. A., Plimack, E. R., Lambert, A. M., Waxman, I. M., & Hammers, H. J. (2015). Nivolumab for Metastatic Renal Cell Carcinoma: Results of a Randomized Phase II Trial. Journal of Clinical Oncology, 33(13), 1430–1437. https://doi.org/10.1200/JCO.2014.59.0703
- Muro, K., Chung, H. C., Shankaran, V., Geva, R., Catenacci, D., Gupta, S., Eder, J. P., Golan, T., Le, D. T., Burtness, B., McRee, A. J., Lin, C.-C., Pathiraja, K., Lunceford, J., Emancipator, K., Juco, J., Koshiji, M., & Bang, Y.-J. (2016). Pembrolizumab for patients with PD-L1-positive advanced gastric cancer (KEYNOTE-012): A multicentre, open-label, phase 1b trial. The Lancet Oncology, 17(6), 717–726. https://doi.org/10.1016/S1470-2045(16)00175-3
- Naumann, R. W., Hollebecque, A., Meyer, T., Devlin, M.-J., Oaknin, A., Kerger, J., López-Picazo, J. M., Machiels, J.-P., Delord, J.-P., Evans, T. R. J., Boni, V., Calvo, E., Topalian, S. L., Chen, T., Soumaoro, I., Li, B., Gu, J., Zwirtes, R., & Moore, K. N. (2019). Safety and Efficacy of Nivolumab Monotherapy in Recurrent or Metastatic Cervical, Vaginal, or Vulvar Carcinoma: Results From the Phase I/II CheckMate 358 Trial. Journal of Clinical Oncology, 37(31), 2825–2834. https://doi.org/10.1200/JCO.19.00739
- Ornstein, M. C., Wood, L. S., Hobbs, B. P., Allman, K. D., Martin, A., Bevan, M., Gilligan, T. D., Garcia, J. A., & Rini, B. I. (2019). A phase II trial of intermittent nivolumab in patients with metastatic renal cell carcinoma (mRCC) who have received prior anti-angiogenic therapy. Journal for ImmunoTherapy of Cancer, 7(1), 127. https://doi.org/10.1186/s40425-019-0615-z
- Ottensmeier, C. : W., R. :. Haanen, J. B. :. Robert, C. :. Schadendorf, D. :. Lutzky, J. :. Peschel, C. :. Messina, M. :. Ibrahim, R. :. O’Day, S. (2010). Ipilimumab produces durable objective responses in patients with previously treated, advanced melanoma: Results from a phase III trial. Annals of Oncology, 21(SUPPL. 8). https://doi.org/10.1093/annonc/mdq535
- Overman, M. J., McDermott, R., Leach, J. L., Lonardi, S., Lenz, H.-J., Morse, M. A., Desai, J., Hill, A., Axelson, M., Moss, R. A., Goldberg, M. V., Cao, Z. A., Ledeine, J.-M., Maglinte, G. A., Kopetz, S., & André, T. (2017). Nivolumab in patients with metastatic DNA mismatch repair-deficient or microsatellite instability-high colorectal cancer (CheckMate 142): An open-label, multicentre, phase 2 study. The Lancet Oncology, 18(9), 1182–1191. https://doi.org/10.1016/S1470-2045(17)30422-9
- Patel, M. R., Ellerton, J., Infante, J. R., Agrawal, M., Gordon, M., Aljumaily, R., Britten, C. D., Dirix, L., Lee, K.-W., Taylor, M., Schöffski, P., Wang, D., Ravaud, A., Gelb, A. B., Xiong, J., Rosen, G., Gulley, J. L., & Apolo, A. B. (2018). Avelumab in metastatic urothelial carcinoma after platinum failure (JAVELIN Solid Tumor): Pooled results from two expansion cohorts of an open-label, phase 1 trial. The Lancet Oncology, 19(1), 51–64. https://doi.org/10.1016/S1470-2045(17)30900-2
- Pignon, J.-C., Jegede, O., Shukla, S. A., Braun, D. A., Horak, C. E., Wind-Rotolo, M., Ishii, Y., Catalano, P. J., Grosha, J., Flaifel, A., Novak, J. S., Mahoney, K. M., Freeman, G. J., Sharpe, A. H., Hodi, F. S., Motzer, R. J., Choueiri, T. K., Wu, C. J., Atkins, M. B., … Signoretti, S. (2019). irRECIST for the Evaluation of Candidate Biomarkers of Response to Nivolumab in Metastatic Clear Cell Renal Cell Carcinoma: Analysis of a Phase II Prospective Clinical Trial. Clinical Cancer Research, 25(7), 2174–2184. https://doi.org/10.1158/1078-0432.CCR-18-3206
- Powles, T., Kockx, M., Rodriguez-Vida, A., Duran, I., Crabb, S. J., Van Der Heijden, M. S., Szabados, B., Pous, A. F., Gravis, G., Herranz, U. A., Protheroe, A., Ravaud, A., Maillet, D., Mendez, M. J., Suarez, C., Linch, M., Prendergast, A., van Dam, P.-J., Stanoeva, D., … Castellano, D. (2019). Clinical efficacy and biomarker analysis of neoadjuvant atezolizumab in operable urothelial carcinoma in the ABACUS trial. Nature Medicine, 25(11), 1706–1714. https://doi.org/10.1038/s41591-019-0628-7
- Rexer, H., Steiner, Th., & Grünwald, V. (2017). Erstlinientherapie beim fortgeschrittenen Nierenzellkarzinom: Eine randomisierte Phase-II-Studie zur Untersuchung einer frühzeitigen Therapieumstellung von Tyrosinkinaseinhibitoren auf Nivolumab im Vergleich zu einer fortgesetzten Tyrosinkinaseinhibitortherapie bei Patienten mit fortgeschrittenem oder metastasiertem Nierenzellkarzinom und stabiler Erkrankung nach 3‑monatiger Behandlung (NIVOSWITCH) – AN 38/15 der AUO. Der Urologe, 56(4), 509–511. https://doi.org/10.1007/s00120-017-0343-2
- Rini, B. I., Plimack, E. R., Stus, V., Gafanov, R., Hawkins, R., Nosov, D., Pouliot, F., Alekseev, B., Soulières, D., Melichar, B., Vynnychenko, I., Kryzhanivska, A., Bondarenko, I., Azevedo, S. J., Borchiellini, D., Szczylik, C., Markus, M., McDermott, R. S., Bedke, J., … Powles, T. (2019). Pembrolizumab plus Axitinib versus Sunitinib for Advanced Renal-Cell Carcinoma. New England Journal of Medicine, 380(12), 1116–1127. https://doi.org/10.1056/NEJMoa1816714
- Rini, B. I., Powles, T., Atkins, M. B., Escudier, B., McDermott, D. F., Suarez, C., Bracarda, S., Stadler, W. M., Donskov, F., Lee, J. L., Hawkins, R., Ravaud, A., Alekseev, B., Staehler, M., Uemura, M., De Giorgi, U., Mellado, B., Porta, C., Melichar, B., … Motzer, R. J. (2019). Atezolizumab plus bevacizumab versus sunitinib in patients with previously untreated metastatic renal cell carcinoma (IMmotion151): A multicentre, open-label, phase 3, randomised controlled trial. The Lancet, 393(10189), 2404–2415. https://doi.org/10.1016/S0140-6736(19)30723-8
- Rizvi, N. A., Mazières, J., Planchard, D., Stinchcombe, T. E., Dy, G. K., Antonia, S. J., Horn, L., Lena, H., Minenza, E., Mennecier, B., Otterson, G. A., Campos, L. T., Gandara, D. R., Levy, B. P., Nair, S. G., Zalcman, G., Wolf, J., Souquet, P.-J., Baldini, E., … Ramalingam, S. S. (2015). Activity and safety of nivolumab, an anti-PD-1 immune checkpoint inhibitor, for patients with advanced, refractory squamous non-small-cell lung cancer (CheckMate 063): A phase 2, single-arm trial. The Lancet Oncology, 16(3), 257–265. https://doi.org/10.1016/S1470-2045(15)70054-9
- Robert, C., Ribas, A., Wolchok, J. D., Hodi, F. S., Hamid, O., Kefford, R., Weber, J. S., Joshua, A. M., Hwu, W.-J., Gangadhar, T. C., Patnaik, A., Dronca, R., Zarour, H., Joseph, R. W., Boasberg, P., Chmielowski, B., Mateus, C., Postow, M. A., Gergich, K., … Daud, A. (2014). Anti-programmed-death-receptor-1 treatment with pembrolizumab in ipilimumab-refractory advanced melanoma: A randomised dose-comparison cohort of a phase 1 trial. The Lancet, 384(9948), 1109–1117. https://doi.org/10.1016/S0140-6736(14)60958-2
- Ross, A. E., Hurley, P. J., Tran, P. T., Rowe, S. P., Benzon, B., Neal, T. O., Chapman, C., Harb, R., Milman, Y., Trock, B. J., Drake, C. G., & Antonarakis, E. S. (2020). A pilot trial of pembrolizumab plus prostatic cryotherapy for men with newly diagnosed oligometastatic hormone-sensitive prostate cancer. Prostate Cancer and Prostatic Diseases, 23(1), 184–193. https://doi.org/10.1038/s41391-019-0176-8
- Royal, R. E., Levy, C., Turner, K., Mathur, A., Hughes, M., Kammula, U. S., Sherry, R. M., Topalian, S. L., Yang, J. C., Lowy, I., & Rosenberg, S. A. (2010). Phase 2 Trial of Single Agent Ipilimumab (Anti-CTLA-4) for Locally Advanced or Metastatic Pancreatic Adenocarcinoma. Journal of Immunotherapy, 33(8), 828–833. https://doi.org/10.1097/CJI.0b013e3181eec14c
- Rozeman, E. A., Menzies, A. M., van Akkooi, A. C. J., Adhikari, C., Bierman, C., van de Wiel, B. A., Scolyer, R. A., Krijgsman, O., Sikorska, K., Eriksson, H., Broeks, A., van Thienen, J. V., Guminski, A. D., Acosta, A. T., ter Meulen, S., Koenen, A. M., Bosch, L. J. W., Shannon, K., Pronk, L. M., … Blank, C. U. (2019). Identification of the optimal combination dosing schedule of neoadjuvant ipilimumab plus nivolumab in macroscopic stage III melanoma (OpACIN-neo): A multicentre, phase 2, randomised, controlled trial. The Lancet Oncology, 20(7), 948–960. https://doi.org/10.1016/S1470-2045(19)30151-2
- Seiwert, T. Y., Burtness, B., Mehra, R., Weiss, J., Berger, R., Eder, J. P., Heath, K., McClanahan, T., Lunceford, J., Gause, C., Cheng, J. D., & Chow, L. Q. (2016). Safety and clinical activity of pembrolizumab for treatment of recurrent or metastatic squamous cell carcinoma of the head and neck (KEYNOTE-012): An open-label, multicentre, phase 1b trial. The Lancet Oncology, 17(7), 956–965. https://doi.org/10.1016/S1470-2045(16)30066-3
- Shahabi, V., Whitney, G., Hamid, O., Schmidt, H., Chasalow, S. D., Alaparthy, S., & Jackson, J. R. (2012). Assessment of association between BRAF-V600E mutation status in melanomas and clinical response to ipilimumab. Cancer Immunology, Immunotherapy, 61(5), 733–737. https://doi.org/10.1007/s00262-012-1227-3
- Sharma, P., Callahan, M. K., Bono, P., Kim, J., Spiliopoulou, P., Calvo, E., Pillai, R. N., Ott, P. A., de Braud, F., Morse, M., Le, D. T., Jaeger, D., Chan, E., Harbison, C., Lin, C.-S., Tschaika, M., Azrilevich, A., & Rosenberg, J. E. (2016). Nivolumab monotherapy in recurrent metastatic urothelial carcinoma (CheckMate 032): A multicentre, open-label, two-stage, multi-arm, phase 1/2 trial. The Lancet Oncology, 17(11), 1590–1598. https://doi.org/10.1016/S1470-2045(16)30496-X
- Sharma, P., Retz, M., Siefker-Radtke, A., Baron, A., Necchi, A., Bedke, J., Plimack, E. R., Vaena, D., Grimm, M.-O., Bracarda, S., Arranz, J. Á., Pal, S., Ohyama, C., Saci, A., Qu, X., Lambert, A., Krishnan, S., Azrilevich, A., & Galsky, M. D. (2017). Nivolumab in metastatic urothelial carcinoma after platinum therapy (CheckMate 275): A multicentre, single-arm, phase 2 trial. The Lancet Oncology, 18(3), 312–322. https://doi.org/10.1016/S1470-2045(17)30065-7
- Shoushtari, A. N., Friedman, C. F., Navid-Azarbaijani, P., Postow, M. A., Callahan, M. K., Momtaz, P., Panageas, K. S., Wolchok, J. D., & Chapman, P. B. (2018). Measuring Toxic Effects and Time to Treatment Failure for Nivolumab Plus Ipilimumab in Melanoma. JAMA Oncology, 4(1), 98. https://doi.org/10.1001/jamaoncol.2017.2391
- Small, E. J., Tchekmedyian, N. S., Rini, B. I., Fong, L., Lowy, I., & Allison, J. P. (2007). A Pilot Trial of CTLA-4 Blockade with Human Anti-CTLA-4 in Patients with Hormone-Refractory Prostate Cancer. Clinical Cancer Research, 13(6), 1810–1815. https://doi.org/10.1158/1078-0432.CCR-06-2318
- Sonpavde, G., Maughan, B. L., Wei, X. X., McGregor, B. A., Kilbridge, K. L., Lee, R. J., Yu, E., Schweizer, M. T., Montgomery, R. B., Cheng, H. H., Hsieh, A. C., Birhiray, R. E., Gabrail, N. Y., Nemunaitis, J. J., Rezazadeh, A., Van Veldhuizen, P. J., Vogelzang, N. J., Heery, C. R., & Grivas, P. (2019). A phase II, multicenter, single-arm trial of CV301 plus atezolizumab (Atezo) in locally advanced (unresectable) or metastatic urothelial cancer (UC). Journal of Clinical Oncology, 37(7_suppl), TPS494–TPS494. https://doi.org/10.1200/JCO.2019.37.7_suppl.TPS494
- Spigel, D., Jotte, R., Nemunaitis, J., Shum, M., Schneider, J., Goldschmidt, J., Eisenstein, J., Berz, D., Seneviratne, L., Socoteanu, M., Bhanderi, V., Konduri, K., Xia, M., Wang, H., Hozak, R. R., Gueorguieva, I., Ferry, D., Gandhi, L., Chao, B. H., & Rybkin, I. (2021). Randomized Phase 2 Studies of Checkpoint Inhibitors Alone or in Combination With Pegilodecakin in Patients With Metastatic NSCLC (CYPRESS 1 and CYPRESS 2). Journal of Thoracic Oncology, 16(2), 327–333. https://doi.org/10.1016/j.jtho.2020.10.001
- Sundahl, N., De Wolf, K., Rottey, S., Decaestecker, K., De Maeseneer, D., Meireson, A., Goetghebeur, E., Fonteyne, V., Verbeke, S., De Visschere, P., Reynders, D., Van Gele, M., Brochez, L., & Ost, P. (2017). A phase I/II trial of fixed-dose stereotactic body radiotherapy with sequential or concurrent pembrolizumab in metastatic urothelial carcinoma: Evaluation of safety and clinical and immunologic response. Journal of Translational Medicine, 15(1), 150. https://doi.org/10.1186/s12967-017-1251-3
- Tamura, K., Hasegawa, K., Katsumata, N., Matsumoto, K., Mukai, H., Takahashi, S., Nomura, H., & Minami, H. (2019). Efficacy and safety of nivolumab in Japanese patients with uterine cervical cancer, uterine corpus cancer, or soft tissue sarcoma: Multicenter, open‐label phase 2 trial. Cancer Science, 110(9), 2894–2904. https://doi.org/10.1111/cas.14148
- Tarhini, A. A., Lee, S. J., Hodi, F. S., Rao, U. N. M., Cohen, G. I., Hamid, O., Hutchins, L. F., Sosman, J. A., Kluger, H. M., Eroglu, Z., Koon, H. B., Lawrence, D. P., Kendra, K. L., Minor, D. R., Lee, C. B., Albertini, M. R., Flaherty, L. E., Petrella, T. M., Streicher, H., … Kirkwood, J. M. (2020). Phase III Study of Adjuvant Ipilimumab (3 or 10 mg/kg) Versus High-Dose Interferon Alfa-2b for Resected High-Risk Melanoma: North American Intergroup E1609. Journal of Clinical Oncology, 38(6), 567–575. https://doi.org/10.1200/JCO.19.01381
- Tawbi, H. A., Burgess, M., Bolejack, V., Van Tine, B. A., Schuetze, S. M., Hu, J., D’Angelo, S., Attia, S., Riedel, R. F., Priebat, D. A., Movva, S., Davis, L. E., Okuno, S. H., Reed, D. R., Crowley, J., Butterfield, L. H., Salazar, R., Rodriguez-Canales, J., Lazar, A. J., … Patel, S. (2017). Pembrolizumab in advanced soft-tissue sarcoma and bone sarcoma (SARC028): A multicentre, two-cohort, single-arm, open-label, phase 2 trial. The Lancet Oncology, 18(11), 1493–1501. https://doi.org/10.1016/S1470-2045(17)30624-1
- Thoma, C. (2017). No activity of pembrolizumab in phase II trial. Nature Reviews Urology, 14(12), 700–700. https://doi.org/10.1038/nrurol.2017.194
- Tollefson, M., Karnes, R. J., Thompson, R. H., Granberg, C., Hillman, D., Breau, R., Allison, J., Kwon, E., & Blute, M. (2010). 668 A RANDOMIZED PHASE II STUDY OF IPILIMUMAB WITH ANDROGEN ABLATION COMPARED WITH ANDROGEN ABLATION ALONE IN PATIENTS WITH ADVANCED PROSTATE CANCER. Journal of Urology, 183(4S). https://doi.org/10.1016/j.juro.2010.02.1055
- Toulmonde, M., Penel, N., Adam, J., Chevreau, C., Blay, J.-Y., Le Cesne, A., Bompas, E., Piperno-Neumann, S., Cousin, S., Grellety, T., Ryckewaert, T., Bessede, A., Ghiringhelli, F., Pulido, M., & Italiano, A. (2018). Use of PD-1 Targeting, Macrophage Infiltration, and IDO Pathway Activation in Sarcomas: A Phase 2 Clinical Trial. JAMA Oncology, 4(1), 93. https://doi.org/10.1001/jamaoncol.2017.1617
- Vaishampayan, U., Schöffski, P., Ravaud, A., Borel, C., Peguero, J., Chaves, J., Morris, J. C., Kotecki, N., Smakal, M., Zhou, D., Guenther, S., Bajars, M., & Gulley, J. L. (2019). Avelumab monotherapy as first-line or second-line treatment in patients with metastatic renal cell carcinoma: Phase Ib results from the JAVELIN Solid Tumor trial. Journal for ImmunoTherapy of Cancer, 7(1), 275. https://doi.org/10.1186/s40425-019-0746-2
- Weber, J. S. : B., D. :. Siegel, J. :. Minor, D. :. Maraveyas, A. :. Hamid, O. (2008). Clinical activity of ipilimumab in patients with advanced melanoma and brain metastases. Annals of Oncology, 19(S8). https://doi.org/10.1093/annonc/mdn516
- Weber, J., Thompson, J. A., Hamid, O., Minor, D., Amin, A., Ron, I., Ridolfi, R., Assi, H., Maraveyas, A., Berman, D., Siegel, J., & O’Day, S. J. (2009). A Randomized, Double-Blind, Placebo-Controlled, Phase II Study Comparing the Tolerability and Efficacy of Ipilimumab Administered with or without Prophylactic Budesonide in Patients with Unresectable Stage III or IV Melanoma. Clinical Cancer Research, 15(17), 5591–5598. https://doi.org/10.1158/1078-0432.CCR-09-1024

**Study Design**

- A Multi-arm Phase I Safety Study of Nivolumab in Combination With Gemcitabine/Cisplatin, Pemetrexed/Cisplatin, Carboplatin/Paclitaxel, Bevacizumab Maintenance, Erlotinib, Ipilimumab or as Monotherapy in Subjects With Stage IIIB/IV Non-small Cell Lung Cancer (NSCLC) (Clinical Trial Registration No. NCT01454102). clinicaltrials.gov. Retrieved 13 December 2021, from https://clinicaltrials.gov/ct2/show/NCT01454102
- A Phase IIa, Open-Label, Multi-Center, Multi-Cohort, Immune-Modulated Study of Selected Small Molecules (Gefitinib, AZD9291, or Selumetinib + Docetaxel) or a 1st Immune-Mediated Therapy (IMT; Tremelimumab) With a Sequential Switch to a 2nd IMT (MEDI4736) in Patients With Locally Advanced or Metastatic Non-Small-Cell Lung Cancer (Stage IIIB-IV) (Clinical Trial Registration No. NCT02179671). Retrieved 13 December 2021, from https://clinicaltrials.gov/ct2/show/NCT02179671
- A Phase III Randomized Open-Label Study of Single Agent Pembrolizumab vs Physicians’ Choice of Single Agent Docetaxel, Paclitaxel, or Irinotecan in Subjects With Advanced/Metastatic Adenocarcinoma and Squamous Cell Carcinoma of the Esophagus That Have Progressed After First-Line Standard Therapy (KEYNOTE-181) (Clinical Trial Registration No. NCT03933449). clinicaltrials.gov. Retrieved 13 December 2021, from https://clinicaltrials.gov/ct2/show/NCT03933449
- A Randomized, Open Label, Phase III Study of Overall Survival Comparing Pembrolizumab (MK-3475) Versus Platinum Based Chemotherapy in Treatment Naïve Subjects With PD-L1 Positive Advanced or Metastatic Non-Small Cell Lung Cancer (Keynote 042) (Clinical Trial Registration No. NCT03850444). clinicaltrials.gov. Retrieved 13 December 2021, from https://clinicaltrials.gov/ct2/show/NCT03850444
- Adams, S., Diamond, J. R., Hamilton, E., Pohlmann, P. R., Tolaney, S. M., Chang, C.-W., Zhang, W., Iizuka, K., Foster, P. G., Molinero, L., Funke, R., & Powderly, J. (2019). Atezolizumab Plus nab-Paclitaxel in the Treatment of Metastatic Triple-Negative Breast Cancer With 2-Year Survival Follow-up: A Phase 1b Clinical Trial. JAMA Oncology, 5(3), 334. https://doi.org/10.1001/jamaoncol.2018.5152
- Antonia, S. J., López-Martin, J. A., Bendell, J., Ott, P. A., Taylor, M., Eder, J. P., Jäger, D., Pietanza, M. C., Le, D. T., de Braud, F., Morse, M. A., Ascierto, P. A., Horn, L., Amin, A., Pillai, R. N., Evans, J., Chau, I., Bono, P., Atmaca, A., … Calvo, E. (2016). Nivolumab alone and nivolumab plus ipilimumab in recurrent small-cell lung cancer (CheckMate 032): A multicentre, open-label, phase 1/2 trial. The Lancet Oncology, 17(7), 883–895. https://doi.org/10.1016/S1470-2045(16)30098-5
- Ascierto, P. A., Simeone, E., Sileni, V. C., Vecchio, M. D., Marchetti, P., Cappellini, G. C. A., Ridolfi, R., de Rosa, F., Cognetti, F., Ferraresi, V., Testori, A., Queirolo, P., Bernengo, M. G., Guida, M., Galli, L., Mandalà, M., Cimminiello, C., Rinaldi, G., Carnevale-Schianca, F., & Maio, M. (2014). Sequential Treatment with Ipilimumab and BRAF Inhibitors in Patients With Metastatic Melanoma: Data From the Italian Cohort of the Ipilimumab Expanded Access Program. Cancer Investigation, 32(4), 144–149. https://doi.org/10.3109/07357907.2014.885984
- Atkins, M. B., Plimack, E. R., Puzanov, I., Fishman, M. N., McDermott, D. F., Cho, D. C., Vaishampayan, U., George, S., Tarazi, J. C., Duggan, W., Perini, R., Thakur, M., Fernandez, K. C., & Choueiri, T. K. (2021). Axitinib plus pembrolizumab in patients with advanced renal-cell carcinoma: Long-term efficacy and safety from a phase Ib trial. European Journal of Cancer (Oxford, England: 1990), 145, 1–10. https://doi.org/10.1016/j.ejca.2020.12.009
- Bahig, H., Aubin, F., Stagg, J., Gologan, O., Ballivy, O., Bissada, E., Nguyen-Tan, F.-P., Soulières, D., Guertin, L., Filion, E., Christopoulos, A., Lambert, L., Tehfe, M., Ayad, T., Charpentier, D., Jamal, R., & Wong, P. (2019). Phase I/II trial of Durvalumab plus Tremelimumab and stereotactic body radiotherapy for metastatic head and neck carcinoma. BMC Cancer, 19(1), 68. https://doi.org/10.1186/s12885-019-5266-4
- Boasberg, P., Hamid, O., & O’Day, S. (2010). Ipilimumab: Unleashing the Power of the Immune System Through CTLA-4 Blockade. Seminars in Oncology, 37(5), 440–449. https://doi.org/10.1053/j.seminoncol.2010.09.004
- Carneiro, B. A., Konda, B., Costa, R. B., Costa, R. L. B., Sagar, V., Gursel, D. B., Kirschner, L. S., Chae, Y. K., Abdulkadir, S. A., Rademaker, A., Mahalingam, D., Shah, M. H., & Giles, F. J. (2019). Nivolumab in Metastatic Adrenocortical Carcinoma: Results of a Phase 2 Trial. The Journal of Clinical Endocrinology & Metabolism, 104(12), 6193–6200. https://doi.org/10.1210/jc.2019-00600
- Carthon, B. C., Wolchok, J. D., Yuan, J., Kamat, A., Ng Tang, D. S., Sun, J., Ku, G., Troncoso, P., Logothetis, C. J., Allison, J. P., & Sharma, P. (2010). Preoperative CTLA-4 Blockade: Tolerability and Immune Monitoring in the Setting of a Presurgical Clinical Trial. Clinical Cancer Research, 16(10), 2861–2871. https://doi.org/10.1158/1078-0432.CCR-10-0569
- Chang, A. L. S., Tran, D. C., Cannon, J. G. D., Li, S., Jeng, M., Patel, R., Van der Bokke, L., Pague, A., Brotherton, R., Rieger, K. E., Satpathy, A. T., Yost, K. E., Reddy, S., Sarin, K., & Colevas, A. D. (2019). Pembrolizumab for advanced basal cell carcinoma: An investigator-initiated, proof-of-concept study. Journal of the American Academy of Dermatology, 80(2), 564–566. https://doi.org/10.1016/j.jaad.2018.08.017
- Chia, S., Bedard, P. L., Hilton, J., Amir, E., Gelmon, K., Goodwin, R., Villa, D., Cabanero, M., Tu, D., Tsao, M., & Seymour, L. (2019). A Phase Ib Trial of Durvalumab in Combination with Trastuzumab in HER2-Positive Metastatic Breast Cancer (CCTG IND.229). The Oncologist, 24(11), 1439–1445. https://doi.org/10.1634/theoncologist.2019-0321
- Di Giacomo, A. M., Danielli, R., Guidoboni, M., Calabrò, L., Carlucci, D., Miracco, C., Volterrani, L., Mazzei, M. A., Biagioli, M., Altomonte, M., & Maio, M. (2009). Therapeutic efficacy of ipilimumab, an anti-CTLA-4 monoclonal antibody, in patients with metastatic melanoma unresponsive to prior systemic treatments: Clinical and immunological evidence from three patient cases. Cancer Immunology, Immunotherapy, 58(8), 1297–1306. https://doi.org/10.1007/s00262-008-0642-y
- Disselhorst, M. J., Quispel-Janssen, J., Lalezari, F., Monkhorst, K., de Vries, J. F., van der Noort, V., Harms, E., Burgers, S., & Baas, P. (2019). Ipilimumab and nivolumab in the treatment of recurrent malignant pleural mesothelioma (INITIATE): Results of a prospective, single-arm, phase 2 trial. The Lancet Respiratory Medicine, 7(3), 260–270. https://doi.org/10.1016/S2213-2600(18)30420-X
- Eggermont, A. M. M., Testori, A., Maio, M., & Robert, C. (2010). Anti–CTLA-4 Antibody Adjuvant Therapy in Melanoma. Seminars in Oncology, 37(5), 455–459. https://doi.org/10.1053/j.seminoncol.2010.09.009
- El-Khoueiry, A. B., Sangro, B., Yau, T., Crocenzi, T. S., Kudo, M., Hsu, C., Kim, T.-Y., Choo, S.-P., Trojan, J., Welling, T. H., Meyer, T., Kang, Y.-K., Yeo, W., Chopra, A., Anderson, J., dela Cruz, C., Lang, L., Neely, J., Tang, H., … Melero, I. (2017). Nivolumab in patients with advanced hepatocellular carcinoma (CheckMate 040): An open-label, non-comparative, phase 1/2 dose escalation and expansion trial. The Lancet, 389(10088), 2492–2502. https://doi.org/10.1016/S0140-6736(17)31046-2
- Felip, E., Ardizzoni, A., Ciuleanu, T., Cobo, M., Laktionov, K., Szilasi, M., Califano, R., Carcereny, E., Griffiths, R., Paz-Ares, L., Duchnowska, R., Garcia, M. A., Isla, D., Jassem, J., Appel, W., Milanowski, J., Van Meerbeeck, J. P., Wolf, J., Li, A., … Popat, S. (2020). CheckMate 171: A phase 2 trial of nivolumab in patients with previously treated advanced squamous non-small cell lung cancer, including ECOG PS 2 and elderly populations. European Journal of Cancer, 127, 160–172. https://doi.org/10.1016/j.ejca.2019.11.019
- Feng, Y., Wang, X., Bajaj, G., Agrawal, S., Bello, A., Lestini, B., Finckenstein, F. G., Park, J.-S., & Roy, A. (2017). Nivolumab Exposure–Response Analyses of Efficacy and Safety in Previously Treated Squamous or Nonsquamous Non–Small Cell Lung Cancer. Clinical Cancer Research, 23(18), 5394–5405. https://doi.org/10.1158/1078-0432.CCR-16-2842
- Fukuoka, S., Hara, H., Takahashi, N., Kojima, T., Kawazoe, A., Asayama, M., Yoshii, T., Kotani, D., Tamura, H., Mikamoto, Y., Hirano, N., Wakabayashi, M., Nomura, S., Sato, A., Kuwata, T., Togashi, Y., Nishikawa, H., & Shitara, K. (2020). Regorafenib Plus Nivolumab in Patients With Advanced Gastric or Colorectal Cancer: An Open-Label, Dose-Escalation, and Dose-Expansion Phase Ib Trial (REGONIVO, EPOC1603). Journal of Clinical Oncology, 38(18), 2053–2061. https://doi.org/10.1200/JCO.19.03296
- Galsky, M. D., Mortazavi, A., Milowsky, M. I., George, S., Gupta, S., Fleming, M. T., Dang, L. H., Geynisman, D. M., Walling, R., Alter, R. S., Kassar, M., Wang, J., Gupta, S., Davis, N., Picus, J., Philips, G., Quinn, D. I., Haines, G. K., Hahn, N. M., … Pal, S. K. (2020). Randomized Double-Blind Phase II Study of Maintenance Pembrolizumab Versus Placebo After First-Line Chemotherapy in Patients With Metastatic Urothelial Cancer. Journal of Clinical Oncology, 38(16), 1797–1806. https://doi.org/10.1200/JCO.19.03091
- Galsky, M. D., Saci, A., Szabo, P. M., Han, G. C., Grossfeld, G., Collette, S., Siefker-Radtke, A., Necchi, A., & Sharma, P. (2020). Nivolumab in Patients with Advanced Platinum-resistant Urothelial Carcinoma: Efficacy, Safety, and Biomarker Analyses with Extended Follow-up from CheckMate 275. Clinical Cancer Research, 26(19), 5120–5128. https://doi.org/10.1158/1078-0432.CCR-19-4162
- Habra, M. A., Stephen, B., Campbell, M., Hess, K., Tapia, C., Xu, M., Rodon Ahnert, J., Jimenez, C., Lee, J. E., Perrier, N. D., Boraddus, R. R., Pant, S., Subbiah, V., Hong, D. S., Zarifa, A., Fu, S., Karp, D. D., Meric-Bernstam, F., & Naing, A. (2019). Phase II clinical trial of pembrolizumab efficacy and safety in advanced adrenocortical carcinoma. Journal for ImmunoTherapy of Cancer, 7(1), 253. https://doi.org/10.1186/s40425-019-0722-x
- Harshman, L. C., Drake, C. G., Haas, N. B., Manola, J., Puligandla, M., Signoretti, S., Cella, D., Gupta, R. T., Bhatt, R., Van Allen, E., Lara, P., Choueiri, T. K., Kapoor, A., Heng, D. Y. C., Shuch, B., Jewett, M., George, D., Michaelson, D., Carducci, M. A., … Allaf, M. (2017). Transforming the Perioperative Treatment Paradigm in Non-Metastatic RCC—A Possible Path Forward. Kidney Cancer, 1(1), 31–40. https://doi.org/10.3233/KCA-170010
- Heinhuis, K. M., Carlino, M., Joerger, M., Di Nicola, M., Meniawy, T., Rottey, S., Moreno, V., Gazzah, A., Delord, J.-P., Paz-Ares, L., Britschgi, C., Schilder, R. J., O’Byrne, K., Curigliano, G., Romano, E., Patah, P., Wang, R., Liu, Y., Bajaj, G., & Siu, L. L. (2020). Safety, Tolerability, and Potential Clinical Activity of a Glucocorticoid-Induced TNF Receptor–Related Protein Agonist Alone or in Combination With Nivolumab for Patients With Advanced Solid Tumors: A Phase 1/2a Dose-Escalation and Cohort-Expansion Clinical Trial. JAMA Oncology, 6(1), 100. https://doi.org/10.1001/jamaoncol.2019.3848
- Heinzl, S. (2019). JAVELIN Renal 101 trial: Avelumab plus axitinib in renal cell carcinoma. Arzneimitteltherapie, 37(1–2), 27–28.
- Hiniker, S. M., Reddy, S. A., Maecker, H. T., Subrahmanyam, P. B., Rosenberg-Hasson, Y., Swetter, S. M., Saha, S., Shura, L., & Knox, S. J. (2016). A Prospective Clinical Trial Combining Radiation Therapy With Systemic Immunotherapy in Metastatic Melanoma. International Journal of Radiation Oncology*Biology*Physics, 96(3), 578–588. https://doi.org/10.1016/j.ijrobp.2016.07.005
- Horn, L., Spigel, D. R., Vokes, E. E., Holgado, E., Ready, N., Steins, M., Poddubskaya, E., Borghaei, H., Felip, E., Paz-Ares, L., Pluzanski, A., Reckamp, K. L., Burgio, M. A., Kohlhäeufl, M., Waterhouse, D., Barlesi, F., Antonia, S., Arrieta, O., Fayette, J., … Eberhardt, W. E. E. (2017). Nivolumab Versus Docetaxel in Previously Treated Patients With Advanced Non–Small-Cell Lung Cancer: Two-Year Outcomes From Two Randomized, Open-Label, Phase III Trials (CheckMate 017 and CheckMate 057). Journal of Clinical Oncology, 35(35), 3924–3933. https://doi.org/10.1200/JCO.2017.74.3062
- Huang, M., Pietanza, M. C., Samkari, A., Pellissier, J., Burke, T., Chandwani, S., Kong, F., & Pickard, A. S. (2019). Q-TWiST Analysis to Assess Benefit–Risk of Pembrolizumab in Patients with PD-L1–Positive Advanced or Metastatic Non-small Cell Lung Cancer. PharmacoEconomics, 37(1), 105–116. https://doi.org/10.1007/s40273-018-0752-0
- Inokuchi, J., & Eto, M. (2019). Profile of pembrolizumab in the treatment of patients with unresectable or metastatic urothelial carcinoma. Cancer Management and Research, Volume 11, 4519–4528. https://doi.org/10.2147/CMAR.S167708
- Katsuya, Y., Horinouchi, H., Seto, T., Umemura, S., Hosomi, Y., Satouchi, M., Nishio, M., Kozuki, T., Hida, T., Sukigara, T., Nakamura, K., Kuchiba, A., & Ohe, Y. (2019). Single-arm, multicentre, phase II trial of nivolumab for unresectable or recurrent thymic carcinoma: PRIMER study. European Journal of Cancer, 113, 78–86. https://doi.org/10.1016/j.ejca.2019.03.012
- Kazandjian, D., Khozin, S., Blumenthal, G., Zhang, L., Tang, S., Libeg, M., Kluetz, P., Sridhara, R., Keegan, P., & Pazdur, R. (2016). Benefit-Risk Summary of Nivolumab for Patients With Metastatic Squamous Cell Lung Cancer After Platinum-Based Chemotherapy: A Report From the US Food and Drug Administration. JAMA Oncology, 2(1), 118. https://doi.org/10.1001/jamaoncol.2015.3934
- Kazandjian, D., Suzman, D. L., Blumenthal, G., Mushti, S., He, K., Libeg, M., Keegan, P., & Pazdur, R. (2016). FDA Approval Summary: Nivolumab for the Treatment of Metastatic Non-Small Cell Lung Cancer With Progression On or After Platinum-Based Chemotherapy. The Oncologist, 21(5), 634–642. https://doi.org/10.1634/theoncologist.2015-0507
- Konstantinopoulos, P. A., Waggoner, S., Vidal, G. A., Mita, M., Moroney, J. W., Holloway, R., Van Le, L., Sachdev, J. C., Chapman-Davis, E., Colon-Otero, G., Penson, R. T., Matulonis, U. A., Kim, Y. B., Moore, K. N., Swisher, E. M., Färkkilä, A., D’Andrea, A., Stringer-Reasor, E., Wang, J., … Munster, P. (2019). Single-Arm Phases 1 and 2 Trial of Niraparib in Combination With Pembrolizumab in Patients With Recurrent Platinum-Resistant Ovarian Carcinoma. JAMA Oncology, 5(8), 1141. https://doi.org/10.1001/jamaoncol.2019.1048
- Kudo, T., Hamamoto, Y., Kato, K., Ura, T., Kojima, T., Tsushima, T., Hironaka, S., Hara, H., Satoh, T., Iwasa, S., Muro, K., Yasui, H., Minashi, K., Yamaguchi, K., Ohtsu, A., Doki, Y., & Kitagawa, Y. (2017). Nivolumab treatment for oesophageal squamous-cell carcinoma: An open-label, multicentre, phase 2 trial. The Lancet Oncology, 18(5), 631–639. https://doi.org/10.1016/S1470-2045(17)30181-X
- Larkin, J., Lao, C. D., Urba, W. J., McDermott, D. F., Horak, C., Jiang, J., & Wolchok, J. D. (2015). Efficacy and Safety of Nivolumab in Patients With BRAF V600 Mutant and BRAF Wild-Type Advanced Melanoma: A Pooled Analysis of 4 Clinical Trials. JAMA Oncology, 1(4), 433. https://doi.org/10.1001/jamaoncol.2015.1184
- Liu, S. V., Camidge, D. R., Gettinger, S. N., Giaccone, G., Heist, R. S., Hodi, F. S., Ready, N. E., Zhang, W., Wallin, J., Funke, R., Waterkamp, D., Foster, P., Iizuka, K., & Powderly, J. (2018). Long-term survival follow-up of atezolizumab in combination with platinum-based doublet chemotherapy in patients with advanced non–small-cell lung cancer. European Journal of Cancer, 101, 114–122. https://doi.org/10.1016/j.ejca.2018.06.033
- Lundgren, K. T., Farina, M. S., & Bellmunt, J. (2017). Pembrolizumab in the treatment of advanced urothelial cancer. Future Oncology, 13(30), 2745–2758. https://doi.org/10.2217/fon-2017-0284
- Lynch, T. J., Bondarenko, I. N., Luft, A., Serwatowski, P., Barlesi, F., Chacko, R. T., Sebastian, M., Siegel, J., Cuillerot, J., & Reck, M. (2010). Phase II trial of ipilimumab (IPI) and paclitaxel/carboplatin (P/C) in first-line stage IIIb/IV non-small cell lung cancer (NSCLC). Journal of Clinical Oncology, 28(15_suppl), 7531–7531. https://doi.org/10.1200/jco.2010.28.15_suppl.7531
- Lynch, T. J. : N., J. :. Bondarenko, I. :. Luft, A. :. Serwatowski, P. :. Barlesi, F. :. Chacko, R. :. Sebastian, M. :. Cuillerot, J. :. Reck, M. (2010). Overall survival and progression free survival results for a randomized phase 2 trial of ipilimumab (IPI) and paclitaxel/carboplatin (P/C) in first-line stage IIIb/IV non-small cell lung cancer (NSCLC). Journal of Thoracic Oncology, 5(12 SUPPL. 7), S515. https://doi.org/10.1097/JTO.0b013e3182004f15
- Maio, M., Lebbé, C., Sileni, V. C., Siegel, J., Hoos, A., Humphrey, R., O’Day, S., Wolchok, J., Weber, J., & Harmankaya, K. (2009). 9307 Long-term survival in advanced melanoma patients treated with ipilimumab at 10 mg/kg: Ongoing analyses from completed Phase II trials. European Journal of Cancer Supplements, 7(2), 578. https://doi.org/10.1016/S1359-6349(09)71951-2
- Makker, V., Rasco, D., Vogelzang, N. J., Brose, M. S., Cohn, A. L., Mier, J., Di Simone, C., Hyman, D. M., Stepan, D. E., Dutcus, C. E., Schmidt, E. V., Guo, M., Sachdev, P., Shumaker, R., Aghajanian, C., & Taylor, M. (2019). Lenvatinib plus pembrolizumab in patients with advanced endometrial cancer: An interim analysis of a multicentre, open-label, single-arm, phase 2 trial. The Lancet Oncology, 20(5), 711–718. https://doi.org/10.1016/S1470-2045(19)30020-8
- Margolin, K. (2012). Ipilimumab in a Phase II trial of melanoma patients with brain metastases. OncoImmunology, 1(7), 1197–1199. https://doi.org/10.4161/onci.20687
- Meindl-Beinker, N. M., Betge, J., Gutting, T., Burgermeister, E., Belle, S., Zhan, T., Schulte, N., Maenz, M., Ebert, M. P., & Haertel, N. (2019). A multicenter open-label phase II trial to evaluate nivolumab and ipilimumab for 2nd line therapy in elderly patients with advanced esophageal squamous cell cancer (RAMONA). BMC Cancer, 19(1), 231. https://doi.org/10.1186/s12885-019-5446-2
- Morgensztern, D., & Herbst, R. S. (2016). Nivolumab and Pembrolizumab for Non–Small Cell Lung Cancer. Clinical Cancer Research, 22(15), 3713–3717. https://doi.org/10.1158/1078-0432.CCR-15-2998
- Mori, T. (2011). [Ipilimumab, a new molecular targetted therapy of malignant neoplastic disease]. Gan to Kagaku Ryoho. Cancer & Chemotherapy, 38(1), 31–35.
- Naing, A., Wong, D. J., Infante, J. R., Korn, W. M., Aljumaily, R., Papadopoulos, K. P., Autio, K. A., Pant, S., Bauer, T. M., Drakaki, A., Daver, N. G., Hung, A., Ratti, N., McCauley, S., Van Vlasselaer, P., Verma, R., Ferry, D., Oft, M., Diab, A., … Tannir, N. M. (2019). Pegilodecakin combined with pembrolizumab or nivolumab for patients with advanced solid tumours (IVY): A multicentre, multicohort, open-label, phase 1b trial. The Lancet Oncology, 20(11), 1544–1555. https://doi.org/10.1016/S1470-2045(19)30514-5
- Nosaki, K., Saka, H., Hosomi, Y., Baas, P., de Castro, G., Reck, M., Wu, Y.-L., Brahmer, J. R., Felip, E., Sawada, T., Noguchi, K., Han, S. R., Piperdi, B., Kush, D. A., & Lopes, G. (2019). Safety and efficacy of pembrolizumab monotherapy in elderly patients with PD-L1–positive advanced non–small-cell lung cancer: Pooled analysis from the KEYNOTE-010, KEYNOTE-024, and KEYNOTE-042 studies. Lung Cancer, 135, 188–195. https://doi.org/10.1016/j.lungcan.2019.07.004
- Oxnard, G. R., Yang, J. C.-H., Yu, H., Kim, S.-W., Saka, H., Horn, L., Goto, K., Ohe, Y., Mann, H., Thress, K. S., Frigault, M. M., Vishwanathan, K., Ghiorghiu, D., Ramalingam, S. S., & Ahn, M.-J. (2020). TATTON: A multi-arm, phase Ib trial of osimertinib combined with selumetinib, savolitinib, or durvalumab in EGFR-mutant lung cancer. Annals of Oncology, 31(4), 507–516. https://doi.org/10.1016/j.annonc.2020.01.013
- Rounds, A., & Kolesar, J. (2015). Nivolumab for second-line treatment of metastatic squamous non-small-cell lung cancer. American Journal of Health-System Pharmacy, 72(21), 1851–1855. https://doi.org/10.2146/ajhp150235
- Scholz, M., Yep, S., Chancey, M., Kelly, C., Chau, K., Turner, J., Lam, R., & Drake, C. (2017). Phase I clinical trial of sipuleucel-T combined with escalating doses of ipilimumab in progressive metastatic castrate-resistant prostate cancer. ImmunoTargets and Therapy, Volume 6, 11–16. https://doi.org/10.2147/ITT.S122497
- Shao, N., Wan, F., Zhu, Y., & Ye, D. (2019). Conditional Survival in Patients with Advanced Renal Cell Carcinoma Treated with Nivolumab. Medical Science Monitor, 25, 6518–6522. https://doi.org/10.12659/MSM.916984
- Singla, N. (2020). Re: Brian I. Rini, Thomas Powles, Michael B. Atkins, et al. Atezolizumab plus Bevacizumab Versus Sunitinib in Patients with Previously Untreated Metastatic Renal Cell Carcinoma (IMmotion151): A Multicentre, Open-label, Phase 3, Randomised Controlled Trial. Lancet 2019;393:2404–15. European Urology, 77(6), e168–e169. https://doi.org/10.1016/j.eururo.2019.09.015
- Thumar, J., & Kluger, H. (2010). Ipilimumab: A Promising Immunotherapy for Melanoma. Oncology (Williston Park, N.Y.), 24, 1280–1288.
- Tolaney, S. M., Barroso-Sousa, R., Keenan, T., Li, T., Trippa, L., Vaz-Luis, I., Wulf, G., Spring, L., Sinclair, N. F., Andrews, C., Pittenger, J., Richardson, E. T., Dillon, D., Lin, N. U., Overmoyer, B., Partridge, A. H., Van Allen, E., Mittendorf, E. A., Winer, E. P., & Krop, I. E. (2020). Effect of Eribulin With or Without Pembrolizumab on Progression-Free Survival for Patients With Hormone Receptor–Positive, ERBB2 -Negative Metastatic Breast Cancer: A Randomized Clinical Trial. JAMA Oncology, 6(10), 1598. https://doi.org/10.1001/jamaoncol.2020.3524
- Trump, D. (2016). Commentary on: ‘Ipilimumab versus placebo after radiotherapy in patients with metastatic castration-resistant prostate cancer that had progressed after docetaxel chemotherapy (CA184-043): A multicentre, randomised, double-blind, phase 3 trial.’ Kwon ED, Drake CG, Scher HI, Fizazi K, Bossi A, van den Eertwegh AJ, Krainer M, Houede N, Santos R, Mahammedi H, Ng S, Maio M, Franke FA, Sundar S, Agarwal N, Bergman AM, Ciuleanu TE, Korbenfeld E, Sengeløv L, Hansen S, Logothetis C, Beer TM, McHenry MB, Gagnier P, Liu D, Gerritsen WR, CA184-043 Investigators. Departments of Urology and Immunology and Mayo Clinic Comprehensive Cancer Center, Mayo Clinic, Rochester, MN, USA, Electronic address: kwon.eugene@mayo.edu; Johns Hopkins Sidney Kimmel Comprehensive Cancer Center and Brady Urological Institute, Baltimore, MD, USA; Memorial Sloan Kettering Cancer Center and Weill Cornell Medical College, New York, NY, USA; Institut Gustave Roussy, University of Paris-Sud, Villejuif, France; Institut Gustave Roussy, Villejuif, France; VU University Medical Centre, Amsterdam, Netherlands; Vienna General Hospital, Medical University Vienna, Vienna, Austria; Institut Bergonié, Bordeaux, France; CHU Caremeau, Nimes, France; Centro Médico Austral, Buenos Aires, Argentina; Centre Jean Perrin, Clermont-Ferrand, France; St John of God Hospital, Subiaco, WA, Australia; University Hospital of Siena, Istituto Toscano Tumori, Siena, Italy; Hospital de Caridade de Ijuí, Ijuí, Brazil; Nottingham University Hospital, Nottingham, UK; Huntsman Cancer Institute, University of Utah, Salt Lake City, UT, USA; Netherlands Cancer Institute and Antoni van Leeuwenhoek Hospital, Amsterdam, Netherlands; Institute of Oncology Ion Chiricuta and University of Medicine and Pharmacy Iuliu Hatieganu, Cluj-Napoca, Romania; Hospital Británico de Buenos Aires, Buenos Aires, Argentina; Herlev Hospital, Herlev, Denmark; Odense University Hospital, Odense, Denmark; University of Texas MD Anderson Cancer Center, Houston,. Urologic Oncology, 34(5), 249–250. https://doi.org/10.1016/j.urolonc.2015.03.013
- Vokes, E. E., Ready, N., Felip, E., Horn, L., Burgio, M. A., Antonia, S. J., Arén Frontera, O., Gettinger, S., Holgado, E., Spigel, D., Waterhouse, D., Domine, M., Garassino, M., Chow, L. Q. M., Blumenschein, G., Barlesi, F., Coudert, B., Gainor, J., Arrieta, O., … Crinò, L. (2018). Nivolumab versus docetaxel in previously treated advanced non-small-cell lung cancer (CheckMate 017 and CheckMate 057): 3-year update and outcomes in patients with liver metastases. Annals of Oncology, 29(4), 959–965. https://doi.org/10.1093/annonc/mdy041
- Weber, J. S., Amin, A., Minor, D., Siegel, J., Berman, D., & O’Day, S. J. (2011). Safety and clinical activity of ipilimumab in melanoma patients with brain metastases: Retrospective analysis of data from a phase 2 trial. Melanoma Research, 21(6), 530–534. https://doi.org/10.1097/CMR.0b013e32834d3d88
- Wolchok, J. D., Weber, J. S., Maio, M., Neyns, B., Harmankaya, K., Chin, K., Cykowski, L., de Pril, V., Humphrey, R., & Lebbé, C. (2013). Four-year survival rates for patients with metastatic melanoma who received ipilimumab in phase II clinical trials. Annals of Oncology, 24(8), 2174–2180. https://doi.org/10.1093/annonc/mdt161
- Zamarin, D., Walderich, S., Holland, A., Zhou, Q., Iasonos, A. E., Torrisi, J. M., Merghoub, T., Chesebrough, L. F., Mcdonnell, A. S., Gallagher, J. M., Li, Y., Hollmann, T. J., Grisham, R. N., Erskine, C. L., Block, M. S., Knutson, K. L., O’Cearbhaill, R. E., Aghajanian, C., & Konner, J. A. (2020). Safety, immunogenicity, and clinical efficacy of durvalumab in combination with folate receptor alpha vaccine TPIV200 in patients with advanced ovarian cancer: A phase II trial. Journal for ImmunoTherapy of Cancer, 8(1), e000829. https://doi.org/10.1136/jitc-2020-000829
- Zheng, H., Mineishi, S., Claxton, D., Zhu, J., Zhao, C., Jia, B., Ehmann, W. C., Rybka, W. B., Naik, S., Songdej, N., Drabick, J. J., & Hohl, R. J. (2021). A phase I clinical trial of avelumab in combination with decitabine as first line treatment of unfit patients with acute myeloid leukemia. American Journal of Hematology, 96(2). https://doi.org/10.1002/ajh.26043

**Protocol**

- Antoniotti, C., Borelli, B., Rossini, D., Pietrantonio, F., Morano, F., Salvatore, L., Lonardi, S., Marmorino, F., Tamberi, S., Corallo, S., Tortora, G., Bergamo, F., Brunella, D. S., Boccaccino, A., Grassi, E., Racca, P., Tamburini, E., Aprile, G., Moretto, R., … Cremolini, C. (2020). AtezoTRIBE: A randomised phase II study of FOLFOXIRI plus bevacizumab alone or in combination with atezolizumab as initial therapy for patients with unresectable metastatic colorectal cancer. BMC Cancer, 20(1), 683. https://doi.org/10.1186/s12885-020-07169-6
- Bex, A., van Thienen, J. V., Schrier, M., Graafland, N., Kuusk, T., Hendricksen, K., Lagerveld, B., Zondervan, P., van Moorselaar, J. A., Blank, C., Wilgenhof, S., & Haanen, J. (2019). A Phase II, single-arm trial of neoadjuvant axitinib plus avelumab in patients with localized renal cell carcinoma who are at high risk of relapse after nephrectomy (NEOAVAX). Future Oncology, 15(19), 2203–2209. https://doi.org/10.2217/fon-2019-0111
- Cortés, J., André, F., Gonçalves, A., Kümmel, S., Martín, M., Schmid, P., Schuetz, F., Swain, S. M., Easton, V., Pollex, E., Deurloo, R., & Dent, R. (2019). IMpassion132 Phase III trial: Atezolizumab and chemotherapy in early relapsing metastatic triple-negative breast cancer. Future Oncology, 15(17), 1951–1961. https://doi.org/10.2217/fon-2019-0059
- Fennell, D. A., Kirkpatrick, E., Cozens, K., Nye, M., Lester, J., Hanna, G., Steele, N., Szlosarek, P., Danson, S., Lord, J., Ottensmeier, C., Barnes, D., Hill, S., Kalevras, M., Maishman, T., & Griffiths, G. (2018). CONFIRM: A double-blind, placebo-controlled phase III clinical trial investigating the effect of nivolumab in patients with relapsed mesothelioma: study protocol for a randomised controlled trial. Trials, 19(1), 233. https://doi.org/10.1186/s13063-018-2602-y
- Fujimoto, N., Aoe, K., Kozuki, T., Oze, I., Kato, K., Kishimoto, T., & Hotta, K. (2018). A Phase II Trial of First-Line Combination Chemotherapy With Cisplatin, Pemetrexed, and Nivolumab for Unresectable Malignant Pleural Mesothelioma: A Study Protocol. Clinical Lung Cancer, 19(5), e705–e707. https://doi.org/10.1016/j.cllc.2018.05.001
- Hack, S. P., Spahn, J., Chen, M., Cheng, A.-L., Kaseb, A., Kudo, M., Lee, H. C., Yopp, A., Chow, P., & Qin, S. (2020). IMbrave 050: A Phase III trial of atezolizumab plus bevacizumab in high-risk hepatocellular carcinoma after curative resection or ablation. Future Oncology, 16(15), 975–989. https://doi.org/10.2217/fon-2020-0162
- Kamat, A. M., Shore, N., Hahn, N., Alanee, S., Nishiyama, H., Shariat, S., Nam, K., Kapadia, E., Frenkl, T., & Steinberg, G. (2020). KEYNOTE-676: Phase III study of BCG and pembrolizumab for persistent/recurrent high-risk NMIBC. Future Oncology, 16(10), 507–516. https://doi.org/10.2217/fon-2019-0817
- Kato, K., Shah, M. A., Enzinger, P., Bennouna, J., Shen, L., Adenis, A., Sun, J.-M., Cho, B. C., Özgüroğlu, M., Kojima, T., Kostorov, V., Hierro, C., Zhu, Y., McLean, L. A., Shah, S., & Doi, T. (2019). KEYNOTE-590: Phase III study of first-line chemotherapy with or without pembrolizumab for advanced esophageal cancer. Future Oncology (London, England), 15(10), 1057–1066. https://doi.org/10.2217/fon-2018-0609
- Kim, S., Buecher, B., André, T., Jary, M., Bidard, F.-C., Ghiringhelli, F., François, É., Taieb, J., Smith, D., de la Fouchardière, C., Desramé, J., Samalin, E., Parzy, A., Baba-Hamed, N., Bouché, O., Tougeron, D., Dahan, L., El Hajbi, F., Jacquin, M., … Borg, C. (2020). Atezolizumab plus modified docetaxel-cisplatin-5-fluorouracil (mDCF) regimen versus mDCF in patients with metastatic or unresectable locally advanced recurrent anal squamous cell carcinoma: A randomized, non-comparative phase II SCARCE GERCOR trial. BMC Cancer, 20(1), 352. https://doi.org/10.1186/s12885-020-06841-1
- Kyte, J. A., Andresen, N. K., Russnes, H. G., Fretland, S. Ø., Falk, R. S., Lingjærde, O. C., & Naume, B. (2020). ICON: A randomized phase IIb study evaluating immunogenic chemotherapy combined with ipilimumab and nivolumab in patients with metastatic hormone receptor positive breast cancer. Journal of Translational Medicine, 18(1), 269. https://doi.org/10.1186/s12967-020-02421-w
- Kyte, J. A., Røssevold, A., Falk, R. S., & Naume, B. (2020). ALICE: A randomized placebo-controlled phase II study evaluating atezolizumab combined with immunogenic chemotherapy in patients with metastatic triple-negative breast cancer. Journal of Translational Medicine, 18(1), 252. https://doi.org/10.1186/s12967-020-02424-7
- Lau, D., Kalaitzaki, E., Church, D. N., Pandha, H., Tomlinson, I., Annels, N., Gerlinger, M., Sclafani, F., Smith, G., Begum, R., Crux, R., Gillbanks, A., Wordsworth, S., Chau, I., Starling, N., Cunningham, D., & Dhillon, T. (2020). Rationale and design of the POLEM trial: Avelumab plus fluoropyrimidine-based chemotherapy as adjuvant treatment for stage III mismatch repair deficient or POLE exonuclease domain mutant colon cancer: a phase III randomised study. ESMO Open, 5(1), e000638. https://doi.org/10.1136/esmoopen-2019-000638
- Lee, J.-Y., Yi, J. Y., Kim, H.-S., Lim, J., Kim, S., Nam, B. H., Kim, H. S., Kim, J. W., Choi, C. H., Kim, B.-G., & KGOG investigators. (2019). An umbrella study of biomarker-driven targeted therapy in patients with platinum-resistant recurrent ovarian cancer: A Korean Gynecologic Oncology Group study (KGOG 3045), AMBITION. Japanese Journal of Clinical Oncology, 49(8), 789–792. https://doi.org/10.1093/jjco/hyz085
- Luke, J. J., Ascierto, P. A., Carlino, M. S., Gershenwald, J. E., Grob, J.-J., Hauschild, A., Kirkwood, J. M., Long, G. V., Mohr, P., Robert, C., Ross, M., Scolyer, R. A., Yoon, C. H., Poklepovic, A., Rutkowski, P., Anderson, J. R., Ahsan, S., Ibrahim, N., & M Eggermont, A. M. (2020). KEYNOTE-716: Phase III study of adjuvant pembrolizumab versus placebo in resected high-risk stage II melanoma. Future Oncology, 16(3), 4429–4438. https://doi.org/10.2217/fon-2019-0666
- Martin, D., Balermpas, P., Gollrad, J., Weiß, C., Valentini, C., Stuschke, M., Schäfer, H., Henkenberens, C., Debus, J., Krug, D., Kuhnt, T., Brunner, T., Bostel, T., Engenhart-Cabillic, R., Nestle, U., Combs, S. E., Belka, C., Hautmann, M., Hildebrandt, G., … Fokas, E. (2020). RADIANCE – Radiochemotherapy with or without Durvalumab in the treatment of anal squamous cell carcinoma: A randomized multicenter phase II trial. Clinical and Translational Radiation Oncology, 23, 43–49. https://doi.org/10.1016/j.ctro.2020.04.010
- Mayadev, J., Nunes, A. T., Li, M., Marcovitz, M., Lanasa, M. C., & Monk, B. J. (2020). CALLA: Efficacy and safety of concurrent and adjuvant durvalumab with chemoradiotherapy versus chemoradiotherapy alone in women with locally advanced cervical cancer: a phase III, randomized, double-blind, multicenter study. International Journal of Gynecologic Cancer, 30(7), 1065–1070. https://doi.org/10.1136/ijgc-2019-001135
- Ngoi, N. Y., Heong, V., Ow, S., Chay, W. Y., Kim, H. S., Choi, C. H., Goss, G., Goh, J. C., Tai, B. C., Lim, D. G., Kaliaperumal, N., Au, V. B., Connolly, J. E., Kim, J.-W., Friedlander, M., Kim, K., & Tan, D. S. (2020). A multicenter phase II randomized trial of durvalumab (MEDI-4736) versus physician’s choice chemotherapy in recurrent ovarian clear cell adenocarcinoma (MOCCA). International Journal of Gynecological Cancer: Official Journal of the International Gynecological Cancer Society, 30(8), 1239–1242. https://doi.org/10.1136/ijgc-2020-001604
- Pascual, T., Cejalvo, J. M., Oliveira, M., Vidal, M., Vega, E., Ganau, S., Julve, A., Zamora, E., Miranda, I., Delgado, A., Bermejo, B., la Cruz-Merino, L. de, Juan, M., Ferrero-Cafiero, J. M., Canes, J., Gonzalez, X., Villagrasa, P., & Prat, A. (2020). SOLTI-1503 PROMETEO TRIAL: Combination of talimogene laherparepvec with atezolizumab in early breast cancer. Future Oncology, 16(24), 1801–1813. https://doi.org/10.2217/fon-2020-0246
- Rexer, H., & Doehn, C. (2016). [First-line treatment for advanced renal cell carcinoma: A phase 3, open-label, randomized study of Atezolizumab (Anti-PD-L1-Antibody) in combination with Bevacizumab versus Sunitinib in patients with untreated advanced renal cell carcinoma (‘IMmotion’) - AN 37/15 der AUO]. Der Urologe. Ausg. A, 55(9), 1242–1243. https://doi.org/10.1007/s00120-016-0188-0
- Shah, M. A., Bennouna, J., Doi, T., Shen, L., Kato, K., Adenis, A., Mamon, H. J., Moehler, M., Fu, X., Cho, B. C., Bordia, S., Bhagia, P., Shih, C.-S., Desai, A., & Enzinger, P. (2021). KEYNOTE-975 study design: A Phase III study of definitive chemoradiotherapy plus pembrolizumab in patients with esophageal carcinoma. Future Oncology, 17(10), 1143–1153. https://doi.org/10.2217/fon-2020-0969
- Smyth, E., Knödler, M., Giraut, A., Mauer, M., Nilsson, M., Van Grieken, N., Wagner, A. D., Moehler, M., & Lordick, F. (2020). VESTIGE: Adjuvant Immunotherapy in Patients With Resected Esophageal, Gastroesophageal Junction and Gastric Cancer Following Preoperative Chemotherapy With High Risk for Recurrence (N+ and/or R1): An Open Label Randomized Controlled Phase-2-Study. Frontiers in Oncology, 9, 1320. https://doi.org/10.3389/fonc.2019.01320
- Solomon, B. J., Zhou, C. C., Drilon, A., Park, K., Wolf, J., Elamin, Y., Davis, H. M., Soldatenkova, V., Sashegyi, A., Lin, A. B., Lin, B. K., F Loong, H. H., Novello, S., Arriola, E., Pérol, M., Goto, K., & Santini, F. C. (2021). Phase III study of selpercatinib versus chemotherapy ± pembrolizumab in untreated RET positive non-small-cell lung cancer. Future Oncology, 17(7), 763–773. https://doi.org/10.2217/fon-2020-0935
- Sonpavde, G., Necchi, A., Gupta, S., Steinberg, G. D., Gschwend, J. E., Van Der Heijden, M. S., Garzon, N., Ibrahim, M., Raybold, B., Liaw, D., Rutstein, M., & Galsky, M. D. (2020). ENERGIZE: A Phase III study of neoadjuvant chemotherapy alone or with nivolumab with/without linrodostat mesylate for muscle-invasive bladder cancer. Future Oncology, 16(2), 4359–4368. https://doi.org/10.2217/fon-2019-0611
- Wu, A. A., Bever, K. M., Ho, W. J., Fertig, E. J., Niu, N., Zheng, L., Parkinson, R. M., Durham, J. N., Onners, B., Ferguson, A. K., Wilt, C., Ko, A. H., Wang-Gillam, A., Laheru, D. A., Anders, R. A., Thompson, E. D., Sugar, E. A., Jaffee, E. M., & Le, D. T. (2020). A Phase II Study of Allogeneic GM-CSF–Transfected Pancreatic Tumor Vaccine (GVAX) with Ipilimumab as Maintenance Treatment for Metastatic Pancreatic Cancer. Clinical Cancer Research, 26(19), 5129–5139. https://doi.org/10.1158/1078-0432.CCR-20-1025
- Zech, H. B., Moeckelmann, N., Boettcher, A., Muenscher, A., Binder, M., Vettorazzi, E., Bokemeyer, C., Schafhausen, P., Betz, C. S., & Busch, C.-J. (2020a). Phase III study of nivolumab alone or combined with ipilimumab as immunotherapy versus standard of care in resectable head and neck squamous cell carcinoma. Future Oncology, 16(36), 3035–3043. https://doi.org/10.2217/fon-2020-0595
- Zech, H. B., Moeckelmann, N., Boettcher, A., Muenscher, A., Binder, M., Vettorazzi, E., Bokemeyer, C., Schafhausen, P., Betz, C. S., & Busch, C.-J. (2020b). Phase III study of nivolumab alone or combined with ipilimumab as immunotherapy versus standard of care in resectable head and neck squamous cell carcinoma. Future Oncology (London, England), 16(36), 3035–3043. https://doi.org/10.2217/fon-2020-0595

**No results**

- A Multicentre Phase II Randomised Trial of Durvalumab (MEDI4736) Versus Physician’s Choice Chemotherapy in Recurrent Ovarian Clear Cell Adenocarcinomas (MOCCA) (Clinical Trial Registration No. NCT03405454). clinicaltrials.gov. Retrieved 13 December 2021, from https://clinicaltrials.gov/ct2/show/NCT03405454
- A Multicentre Randomised Phase III Trial Comparing Pembrolizumab Versus Standard Chemotherapy for Advanced Pre-treated Malignant Pleural Mesothelioma (Clinical Trial Registration No. NCT02991482). clinicaltrials.gov. Retrieved 13 December 2021, from https://clinicaltrials.gov/ct2/show/NCT02991482
- A Phase 2, Randomized, Open-Label Study of Nivolumab Combined With Ipilimumab Versus Standard of Care in Subjects With Previously Untreated and Advanced (Unresectable or Metastatic) Non-clear Cell Renal Cell Carcinoma (nccRCC) (Clinical Trial Registration No. NCT03075423). clinicaltrials.gov. Retrieved 13 December 2021, from https://clinicaltrials.gov/ct2/show/NCT03075423
- A Phase 3, Randomized, Global Trial of Nivolumab and Epacadostat With Platinum Doublet Chemotherapy Versus Platinum Doublet Chemotherapy in First-line Treatment of Stage IV or Recurrent Non-Small Cell Lung Cancer (NSCLC) (Clinical Trial Registration No. NCT03348904). clinicaltrials.gov. Retrieved 13 December 2021, from https://clinicaltrials.gov/ct2/show/NCT03348904
- A Phase II Immunotherapeutic Trial: Combination Androgen Ablative Therapy and CTLA-4 Blockade as a Treatment for Advanced Prostate Cancer (Clinical Trial Registration No. NCT00170157). clinicaltrials.gov. Retrieved 13 December 2021, from https://clinicaltrials.gov/ct2/show/NCT00170157
- A Phase II, Randomised Study of Nivolumab as Consolidation Therapy in Patients With Locally Advanced, Unresectable Non-Small Cell Lung Cancer (Stage III) Who Have Not Progressed Following Neoadjuvant Chemotherapy Plus Nivolumab and Definitive Concurrent Chemoradiation Therapy (Clinical Trial Registration No. NCT04085250). clinicaltrials.gov. Retrieved 13 December 2021, from https://clinicaltrials.gov/ct2/show/NCT04085250
- A Phase II Randomized, Double-Blind, Placebo-Controlled Study Evaluating Nintedanib Versus Placebo as Prophylaxis Against Radiation Pneumonitis in Patients With Unresectable NSCLC Undergoing Chemoradiation Therapy (Clinical Trial Registration No. NCT02452463). clinicaltrials.gov. Retrieved 13 December 2021, from https://clinicaltrials.gov/ct2/show/NCT02452463
- A Prospective Multicenter Open-label, Randomized Phase II Study of Pembrolizumab in Combination With Neoadjuvant EC-Paclitaxel Regimen in HER2-negative Inflammatory Breast Cancer. (Clinical Trial Registration No. NCT03515798). clinicaltrials.gov. Retrieved 13 December 2021, from https://clinicaltrials.gov/ct2/show/NCT03515798
- A Prospective Randomized and Phase II Trial for Metastatic Melanoma Using Adoptive Cell Therapy With Tumor-Infiltrating Lymphocytes Plus IL-2 Either Alone or Following the Administration of Pembrolizumab (Clinical Trial Registration No. NCT02621021). clinicaltrials.gov. Retrieved 13 December 2021, from https://clinicaltrials.gov/ct2/show/NCT02621021
- A Randomised Open-label Phase II Trial of Consolidation With Nivolumab and Ipilimumab in Limited-stage SCLC After Chemo-radiotherapy (Clinical Trial Registration No. NCT02046733). clinicaltrials.gov. Retrieved 13 December 2021, from https://clinicaltrials.gov/ct2/show/NCT02046733
- A Randomized, Open-Label, Phase 3 Study to Evaluate Efficacy and Safety of Pembrolizumab (MK-3475) Plus Epacadostat vs Standard of Care (Sunitinib or Pazopanib) as First-Line Treatment for Locally Advanced or Metastatic Renal Cell Carcinoma (mRCC) (KEYNOTE-679/ECHO-302) (Clinical Trial Registration No. NCT03260894). clinicaltrials.gov. Retrieved 13 December 2021, from https://clinicaltrials.gov/ct2/show/NCT03260894
- A Randomized Phase II Study Evaluating Pembrolizumab vs Topotecan in the Second-Line Treatment of Patients With Small Cell Lung Cancer (Clinical Trial Registration No. NCT02963090). clinicaltrials.gov. Retrieved 13 December 2021, from https://clinicaltrials.gov/ct2/show/NCT02963090
- A Randomized Phase II Study of Pembrolizumab, an Anti-Programmed Cell Death (PD)-1 Antibody, in Combination With Carboplatin Compared to Carboplatin Alone in Breast Cancer Patients With Chest Wall Disease (Clinical Trial Registration No. NCT03095352). clinicaltrials.gov. Retrieved 13 December 2021, from https://clinicaltrials.gov/ct2/show/NCT03095352
- A Randomized Phase II Trial of Standard of Care Alone or in Combination With Ad-CEA Vaccine and Avelumab in Patients With Previously Untreated Metastatic or Unresectable Colorectal Cancer (Clinical Trial Registration No. NCT03050814). clinicaltrials.gov. Retrieved 13 December 2021, from https://clinicaltrials.gov/ct2/show/NCT03050814
- A Randomized, Phase III Study of Fotemustine Versus the Combination of Fotemustine and Ipilimumab or the Combination of Ipilimumab and Nivolumab in Patients With Metastatic Melanoma With Brain Metastasis (Clinical Trial Registration No. NCT02460068). clinicaltrials.gov. Retrieved 13 December 2021, from https://clinicaltrials.gov/ct2/show/NCT02460068
- Adjuvant Nivolumab or Ipilimumab + Nivolumab Determined By Pathological Response To A Single Dose Of Neoadjvuant Nivolumab (Clinical Trial Registration No. NCT04013854). clinicaltrials.gov. Retrieved 13 December 2021, from https://clinicaltrials.gov/ct2/show/NCT04013854
- Boku, N., Ryu, M.-H., Kato, K., Chung, H. C., Minashi, K., Lee, K.-W., Cho, H., Kang, W. K., Komatsu, Y., Tsuda, M., Yamaguchi, K., Hara, H., Fumita, S., Azuma, M., Chen, L.-T., & Kang, Y.-K. (2019). Safety and efficacy of nivolumab in combination with S-1/capecitabine plus oxaliplatin in patients with previously untreated, unresectable, advanced, or recurrent gastric/gastroesophageal junction cancer: Interim results of a randomized, phase II trial (ATTRACTION-4). Annals of Oncology, 30(2), 250–258. https://doi.org/10.1093/annonc/mdy540
- Grau, J. F., Farinas-Madrid, L., & Oaknin, A. (2020). A randomized phase III trial of platinum chemotherapy plus paclitaxel with bevacizumab and atezolizumab versus platinum chemotherapy plus paclitaxel and bevacizumab in metastatic (stage IVB), persistent, or recurrent carcinoma of the cervix: The BEATcc study (ENGOT-Cx10/GEICO 68-C/JGOG1084/GOG-3030). International Journal of Gynecologic Cancer, 30(1), 139–143. https://doi.org/10.1136/ijgc-2019-000880
- METIMMOX: Colorectal Cancer METastasis—Shaping Anti-tumor IMMunity by OXaliplatin (Clinical Trial Registration No. NCT03388190). clinicaltrials.gov. Retrieved 13 December 2021, from https://clinicaltrials.gov/ct2/show/NCT03388190
- Moore, K. N., & Pignata, S. (2019). Trials in progress: IMagyn050/GOG 3015/ENGOT-OV39. A Phase III, multicenter, randomized study of atezolizumab versus placebo administered in combination with paclitaxel, carboplatin, and bevacizumab to patients with newly-diagnosed stage III or stage IV ovarian, fallopian tube, or primary peritoneal cancer. International Journal of Gynecologic Cancer, 29(2), 430–433. https://doi.org/10.1136/ijgc-2018-000071
- Motzer, R. J., Penkov, K., Haanen, J., Rini, B., Albiges, L., Campbell, M. T., Venugopal, B., Kollmannsberger, C., Negrier, S., Uemura, M., Lee, J. L., Vasiliev, A., Miller, W. H., Gurney, H., Schmidinger, M., Larkin, J., Atkins, M. B., Bedke, J., Alekseev, B., … Choueiri, T. K. (2019). Avelumab plus Axitinib versus Sunitinib for Advanced Renal-Cell Carcinoma. New England Journal of Medicine, 380(12), 1103–1115. https://doi.org/10.1056/NEJMoa1816047
- Necchi, A., Giannatempo, P., Raggi, D., Mariani, L., Colecchia, M., Farè, E., Monopoli, F., Calareso, G., Ali, S. M., Ross, J. S., Chung, J. H., & Salvioni, R. (2019). An Open-label Randomized Phase 2 study of Durvalumab Alone or in Combination with Tremelimumab in Patients with Advanced Germ Cell Tumors (APACHE): Results from the First Planned Interim Analysis. European Urology, 75(1), 201–203. https://doi.org/10.1016/j.eururo.2018.09.010
- Open-label, Multicenter, Phase 1b/2 Clinical Study to Evaluate the Safety and Efficacy of CD40 Agonistic Monoclonal Antibody (APX005M) Administered Together With Gemcitabine and Nab-Paclitaxel With or Without PD-1 Blocking Antibody (Nivolumab) in Patients With Previously Untreated Metastatic Pancreatic Adenocarcinoma (Clinical Trial Registration No. NCT03214250). clinicaltrials.gov. Retrieved 13 December 2021, from https://clinicaltrials.gov/ct2/show/NCT03214250
- Phase II Multicentre, Randomized, Open-label Study to Evaluate the Safety and Efficacy of Avelumab With Gemcitabine/Carboplatin Versus Gemcitabine/Carboplatin Alone in Patients With Unresectable or Metastatic Urothelial Carcinoma (UC) Who Have Not Received Prior Systemic Therapy and Who Are Ineligible to Receive Cisplatin-based Therapy. (Clinical Trial Registration No. NCT03390595). clinicaltrials.gov. Retrieved 13 December 2021, from https://clinicaltrials.gov/ct2/show/NCT03390595
- Randomized Phase II Trial Evaluating the Optimal Sequencing of PD-1 Inhibition With Pembrolizumab (MK-3475) and Standard Platinum-based Chemotherapy in Patients With Chemotherapy Naive Stage IV Non-small Cell Lung Cancer (Clinical Trial Registration No. NCT02591615). clinicaltrials.gov. Retrieved 13 December 2021, from https://clinicaltrials.gov/ct2/show/NCT02591615
- Villasboas, J. c., Reeder, C. b., Tun, H. w., Bartlett, N. l., Sharon, E., LaPlant, B., Adjei, A., & Ansell, S. m. (2019). The Dial Study (dual Immunomodulation in Aggressive Lymphoma): Randomized Phase 2 Trial of Varlilumab Plus Nivolumab in Relapsed/Refractory Aggressive B-Cell Lymphomas. Hematological Oncology, 37(S2), 70–71. https://doi.org/10.1002/hon.38_2629

**No adverse events of interest**

- A Phase 2 Proof-of-Concept Study of ACP-196 Alone and in Combination With Pembrolizumab in Subjects With Recurrent Ovarian Cancer (Clinical Trial Registration No. NCT02537444). clinicaltrials.gov. Retrieved 13 December 2021, from https://clinicaltrials.gov/ct2/show/NCT02537444
- A Phase 2b Study of Immune Checkpoint Inhibition With or Without Dorgenmeltucel-L (HyperAcute Melanoma) Immunotherapy for Stage IV Melanoma Patients (Clinical Trial Registration No. NCT02054520). clinicaltrials.gov. Retrieved 13 December 2021, from https://clinicaltrials.gov/ct2/show/NCT02054520
- A Phase 3 Randomized, Open-Label Clinical Study to Evaluate the Efficacy and Safety of Pembrolizumab Plus Epacadostat, Pembrolizumab Monotherapy, and the EXTREME Regimen as First Line Treatment for Recurrent or Metastatic Head and Neck Squamous Cell Carcinoma (KEYNOTE-669/ECHO-304) (Clinical Trial Registration No. NCT03358472). clinicaltrials.gov. Retrieved 13 December 2021, from https://clinicaltrials.gov/ct2/show/NCT03358472
- A Randomized, Open-Label, Two-arm, Comparative Study in Chinese Subjects With Chemotherapy Naïve Stage IV Melanoma Receiving Ipilimumab (3 mg/kg) vs. Dacarbazine (Clinical Trial Registration No. NCT02545075). clinicaltrials.gov. Retrieved 13 December 2021, from https://clinicaltrials.gov/ct2/show/NCT02545075
- Ascierto, P. A., Ferrucci, P. F., Fisher, R., Del Vecchio, M., Atkinson, V., Schmidt, H., Schachter, J., Queirolo, P., Long, G. V., Di Giacomo, A. M., Svane, I. M., Lotem, M., Bar-Sela, G., Couture, F., Mookerjee, B., Ghori, R., Ibrahim, N., Moreno, B. H., & Ribas, A. (2019). Dabrafenib, trametinib and pembrolizumab or placebo in BRAF-mutant melanoma. Nature Medicine, 25(6), 941–946. https://doi.org/10.1038/s41591-019-0448-9
- D’Angelo, S. P., Mahoney, M. R., Van Tine, B. A., Atkins, J., Milhem, M. M., Jahagirdar, B. N., Antonescu, C. R., Horvath, E., Tap, W. D., Schwartz, G. K., & Streicher, H. (2018). Nivolumab with or without ipilimumab treatment for metastatic sarcoma (Alliance A091401): Two open-label, non-comparative, randomised, phase 2 trials. The Lancet Oncology, 19(3), 416–426. https://doi.org/10.1016/S1470-2045(18)30006-8
- Ferrarotto, R., Bell, D., Rubin, M. L., Hutcheson, K. A., Johnson, J. M., Goepfert, R. P., Phan, J., Elamin, Y. Y., Torman, D. K., Warneke, C. L., Hessel, A. C., Garden, A. S., Myers, J. N., Johnson, F. M., Lee, J. J., Sikora, A. G., Gillison, M. L., Glisson, B. S., & Gross, N. D. (2020). Impact of Neoadjuvant Durvalumab with or without Tremelimumab on CD8 + Tumor Lymphocyte Density, Safety, and Efficacy in Patients with Oropharynx Cancer: CIAO Trial Results. Clinical Cancer Research, 26(13), 3211–3219. https://doi.org/10.1158/1078-0432.CCR-19-3977
- Gerber, D. E., Urbanic, J. J., Langer, C., Hu, C., Chang, I.-F., Lu, B., Movsas, B., Jeraj, R., Curran, W. J., & Bradley, J. D. (2017). Treatment Design and Rationale for a Randomized Trial of Cisplatin and Etoposide Plus Thoracic Radiotherapy Followed by Nivolumab or Placebo for Locally Advanced Non–Small-Cell Lung Cancer (RTOG 3505). Clinical Lung Cancer, 18(3), 333–339. https://doi.org/10.1016/j.cllc.2016.10.009
- Hellmann, M. D., Ciuleanu, T.-E., Pluzanski, A., Lee, J. S., Otterson, G. A., Audigier-Valette, C., Minenza, E., Linardou, H., Burgers, S., Salman, P., Borghaei, H., Ramalingam, S. S., Brahmer, J., Reck, M., O’Byrne, K. J., Geese, W. J., Green, G., Chang, H., Szustakowski, J., … Paz-Ares, L. (2018). Nivolumab plus Ipilimumab in Lung Cancer with a High Tumor Mutational Burden. New England Journal of Medicine, 378(22), 2093–2104. https://doi.org/10.1056/NEJMoa1801946
- Kato, K., Cho, B. C., Takahashi, M., Okada, M., Lin, C.-Y., Chin, K., Kadowaki, S., Ahn, M.-J., Hamamoto, Y., Doki, Y., Yen, C.-C., Kubota, Y., Kim, S.-B., Hsu, C.-H., Holtved, E., Xynos, I., Kodani, M., & Kitagawa, Y. (2019). Nivolumab versus chemotherapy in patients with advanced oesophageal squamous cell carcinoma refractory or intolerant to previous chemotherapy (ATTRACTION-3): A multicentre, randomised, open-label, phase 3 trial. The Lancet Oncology, 20(11), 1506–1517. https://doi.org/10.1016/S1470-2045(19)30626-6
- Pujol, J.-L., Greillier, L., Audigier-Valette, C., Moro-Sibilot, D., Uwer, L., Hureaux, J., Guisier, F., Carmier, D., Madelaine, J., Otto, J., Gounant, V., Merle, P., Mourlanette, P., Molinier, O., Renault, A., Rabeau, A., Antoine, M., Denis, M. G., Bommart, S., … Souquet, P.-J. (2019). A Randomized Non-Comparative Phase II Study of Anti-Programmed Cell Death-Ligand 1 Atezolizumab or Chemotherapy as Second-Line Therapy in Patients With Small Cell Lung Cancer: Results From the IFCT-1603 Trial. Journal of Thoracic Oncology, 14(5), 903–913. https://doi.org/10.1016/j.jtho.2019.01.008
- Randomized Phase II Study of AB (Nab-Paclitaxel [Abraxane?], Bevacizumab) Versus Ipilimumab for Therapy of Unresectable Stage IV Metastatic Malignant Melanoma (Clinical Trial Registration No. NCT02158520). clinicaltrials.gov. Retrieved 13 December 2021, from https://clinicaltrials.gov/ct2/show/NCT02158520
- Randomized Phase II Trial of Single Agent Chemotherapy Plus Nivolumab or Single Agent Chemotherapy Alone in Patients With Advanced Squamous or Non-squamous NSCLC With Primary Resistance to Prior PD-1 or PDL-1 Inhibitor (Clinical Trial Registration No. NCT03041181). clinicaltrials.gov. Retrieved 13 December 2021, from https://clinicaltrials.gov/ct2/show/NCT03041181
- Schmid, P., Cortes, J., Pusztai, L., McArthur, H., Kümmel, S., Bergh, J., Denkert, C., Park, Y. H., Hui, R., Harbeck, N., Takahashi, M., Foukakis, T., Fasching, P. A., Cardoso, F., Untch, M., Jia, L., Karantza, V., Zhao, J., Aktan, G., … O’Shaughnessy, J. (2020). Pembrolizumab for Early Triple-Negative Breast Cancer. New England Journal of Medicine, 382(9), 810–821. https://doi.org/10.1056/NEJMoa1910549
- Yang, J. C.-H., Shepherd, F. A., Kim, D.-W., Lee, G.-W., Lee, J. S., Chang, G.-C., Lee, S. S., Wei, Y.-F., Lee, Y. G., Laus, G., Collins, B., Pisetzky, F., & Horn, L. (2019). Osimertinib Plus Durvalumab versus Osimertinib Monotherapy in EGFR T790M–Positive NSCLC following Previous EGFR TKI Therapy: CAURAL Brief Report. Journal of Thoracic Oncology, 14(5), 933–939. https://doi.org/10.1016/j.jtho.2019.02.001

## Table B.1. Included trials

Table B.1. List of included trials.

| **Clinical trial** | **Register number** | **References** |
| --- | --- | --- |
| ABC | NCT02374242 | Long, G. V., Atkinson, V., Lo, S., Sandhu, S., Guminski, A. D., Brown, M. P., Wilmott, J. S., Edwards, J., Gonzalez, M., Scolyer, R. A., Menzies, A. M., & McArthur, G. A. (2018). Combination nivolumab and ipilimumab or nivolumab alone in melanoma brain metastases: A multicentre randomised phase 2 study. The Lancet Oncology, 19(5), 672–681. https://doi.org/10.1016/S1470-2045(18)30139-6 |
| ARTIC-Substudy-A | NCT02352948 | Planchard, D., Reinmuth, N., Orlov, S., Fischer, J. R., Sugawara, S., Mandziuk, S., Marquez-Medina, D., Novello, S., Takeda, Y., Soo, R., Park, K., McCleod, M., Geater, S. L., Powell, M., May, R., Scheuring, U., Stockman, P., & Kowalski, D. (2020). ARCTIC: Durvalumab with or without tremelimumab as third-line or later treatment of metastatic non-small-cell lung cancer. Annals of Oncology: Official Journal of the European Society for Medical Oncology, 31(5), 609–618. https://doi.org/10.1016/j.annonc.2020.02.006 |
| ARTIC-Substudy-B | NCT02352948 | Planchard, D., Reinmuth, N., Orlov, S., Fischer, J. R., Sugawara, S., Mandziuk, S., Marquez-Medina, D., Novello, S., Takeda, Y., Soo, R., Park, K., McCleod, M., Geater, S. L., Powell, M., May, R., Scheuring, U., Stockman, P., & Kowalski, D. (2020). ARCTIC: Durvalumab with or without tremelimumab as third-line or later treatment of metastatic non-small-cell lung cancer. Annals of Oncology: Official Journal of the European Society for Medical Oncology, 31(5), 609–618. https://doi.org/10.1016/j.annonc.2020.02.006 |
| ATTRACTION‑2 | NCT02267343 | Kang, Y.-K., Boku, N., Satoh, T., Ryu, M.-H., Chao, Y., Kato, K., Chung, H. C., Chen, J.-S., Muro, K., Kang, W. K., Yeh, K.-H., Yoshikawa, T., Oh, S. C., Bai, L.-Y., Tamura, T., Lee, K.-W., Hamamoto, Y., Kim, J. G., Chin, K., … Chen, L.-T. (2017). Nivolumab in patients with advanced gastric or gastro-oesophageal junction cancer refractory to, or intolerant of, at least two previous chemotherapy regimens (ONO-4538-12, ATTRACTION-2): A randomised, double-blind, placebo-controlled, phase 3 trial. The Lancet, 390(10111), 2461–2471. https://doi.org/10.1016/S0140-6736(17)31827-5 |
| CA184-041 | NCT00527735 | Lynch, T. J., Bondarenko, I., Luft, A., Serwatowski, P., Barlesi, F., Chacko, R., Sebastian, M., Neal, J., Lu, H., Cuillerot, J.-M., & Reck, M. (2012). Ipilimumab in Combination With Paclitaxel and Carboplatin As First-Line Treatment in Stage IIIB/IV Non–Small-Cell Lung Cancer: Results From a Randomized, Double-Blind, Multicenter Phase II Study. Journal of Clinical Oncology, 30(17), 2046–2054. https://doi.org/10.1200/JCO.2011.38.4032 |
| CA184-043 | NCT00861614 | Kwon, E. D., Drake, C. G., Scher, H. I., Fizazi, K., Bossi, A., van den Eertwegh, A. J. M., Krainer, M., Houede, N., Santos, R., Mahammedi, H., Ng, S., Maio, M., Franke, F. A., Sundar, S., Agarwal, N., Bergman, A. M., Ciuleanu, T. E., Korbenfeld, E., Sengeløv, L., … Gerritsen, W. R. (2014). Ipilimumab versus placebo after radiotherapy in patients with metastatic castration-resistant prostate cancer that had progressed after docetaxel chemotherapy (CA184-043): A multicentre, randomised, double-blind, phase 3 trial. The Lancet Oncology, 15(7), 700–712. https://doi.org/10.1016/S1470-2045(14)70189-5 |
| CA184-095 | NCT01057810 | Beer, T. M., Kwon, E. D., Drake, C. G., Fizazi, K., Logothetis, C., Gravis, G., Ganju, V., Polikoff, J., Saad, F., Humanski, P., Piulats, J. M., Gonzalez Mella, P., Ng, S. S., Jaeger, D., Parnis, F. X., Franke, F. A., Puente, J., Carvajal, R., Sengeløv, L., … Gerritsen, W. (2017). Randomized, Double-Blind, Phase III Trial of Ipilimumab Versus Placebo in Asymptomatic or Minimally Symptomatic Patients With Metastatic Chemotherapy-Naive Castration-Resistant Prostate Cancer. Journal of Clinical Oncology, 35(1), 40–47. https://doi.org/10.1200/JCO.2016.69.1584 |
| CA184-104 | NCT01285609 | Govindan, R., Szczesna, A., Ahn, M.-J., Schneider, C.-P., Gonzalez Mella, P. F., Barlesi, F., Han, B., Ganea, D. E., Von Pawel, J., Vladimirov, V., Fadeeva, N., Lee, K. H., Kurata, T., Zhang, L., Tamura, T., Postmus, P. E., Jassem, J., O’Byrne, K., Kopit, J., … Reck, M. (2017). Phase III Trial of Ipilimumab Combined With Paclitaxel and Carboplatin in Advanced Squamous Non–Small-Cell Lung Cancer. Journal of Clinical Oncology, 35(30), 3449–3457. https://doi.org/10.1200/JCO.2016.71.7629 |
| CA184-153 | NCT02279732 | A Randomized, Multicenter, Double-Blind, Multinational, Phase 3 Trial Comparing the Efficacy of Ipilimumab in Addition to Paclitaxel and Carboplatin Versus Placebo in Addition to Paclitaxel and Carboplatin in Subjects With Stage IV/Recurrent Non-Small Cell Lung Cancer (NSCLC) With Squamous Histology (Clinical Trial Registration No. NCT02279732). clinicaltrials.gov. Retrieved 13 December 2021, from https://clinicaltrials.gov/ct2/show/NCT02279732 |
| CA184-162 | NCT01585987 | Bang, Y.-J., Cho, J. Y., Kim, Y. H., Kim, J. W., Di Bartolomeo, M., Ajani, J. A., Yamaguchi, K., Balogh, A., Sanchez, T., & Moehler, M. (2017). Efficacy of Sequential Ipilimumab Monotherapy versus Best Supportive Care for Unresectable Locally Advanced/Metastatic Gastric or Gastroesophageal Junction Cancer. Clinical Cancer Research, 23(19), 5671–5678. https://doi.org/10.1158/1078-0432.CCR-17-0025 |
| CA184-243 | NCT01709162 | A Randomized, Open-Label, Multicenter Phase II Study of Ipilimumab Retreatment Versus Chemotherapy for Subjects With Advanced Melanoma Who Progressed After Initially Achieving Disease Control With Ipilimumab Therapy (Clinical Trial Registration No. NCT01709162). clinicaltrials.gov. Retrieved 13 December 2021, from https://clinicaltrials.gov/ct2/show/NCT01709162 |
| CASPIAN | NCT03043872 | Paz-Ares, L., Dvorkin, M., Chen, Y., Reinmuth, N., Hotta, K., Trukhin, D., Statsenko, G., Hochmair, M. J., Özgüroğlu, M., Ji, J. H., Voitko, O., Poltoratskiy, A., Ponce, S., Verderame, F., Havel, L., Bondarenko, I., Kazarnowicz, A., Losonczy, G., Conev, N. V., … Williamson, M. (2019). Durvalumab plus platinum–etoposide versus platinum–etoposide in first-line treatment of extensive-stage small-cell lung cancer (CASPIAN): A randomised, controlled, open-label, phase 3 trial. The Lancet, 394(10212), 1929–1939. https://doi.org/10.1016/S0140-6736(19)32222-6 |
| CheckMate 017 | NCT01642004 | Brahmer, J., Reckamp, K. L., Baas, P., Crinò, L., Eberhardt, W. E. E., Poddubskaya, E., Antonia, S., Pluzanski, A., Vokes, E. E., Holgado, E., Waterhouse, D., Ready, N., Gainor, J., Arén Frontera, O., Havel, L., Steins, M., Garassino, M. C., Aerts, J. G., Domine, M., … Spigel, D. R. (2015). Nivolumab versus Docetaxel in Advanced Squamous-Cell Non–Small-Cell Lung Cancer. New England Journal of Medicine, 373(2), 123–135. https://doi.org/10.1056/NEJMoa1504627 |
| CheckMate 025 | NCT01668784 | Motzer, R. J., Escudier, B., McDermott, D. F., George, S., Hammers, H. J., Srinivas, S., Tykodi, S. S., Sosman, J. A., Procopio, G., Plimack, E. R., Castellano, D., Choueiri, T. K., Gurney, H., Donskov, F., Bono, P., Wagstaff, J., Gauler, T. C., Ueda, T., Tomita, Y., … Sharma, P. (2015). Nivolumab versus Everolimus in Advanced Renal-Cell Carcinoma. New England Journal of Medicine, 373(19), 1803–1813. https://doi.org/10.1056/NEJMoa1510665 |
| CheckMate 026 | NCT02041533 | Carbone, D. P., Reck, M., Paz-Ares, L., Creelan, B., Horn, L., Steins, M., Felip, E., van den Heuvel, M. M., Ciuleanu, T.-E., Badin, F., Ready, N., Hiltermann, T. J. N., Nair, S., Juergens, R., Peters, S., Minenza, E., Wrangle, J. M., Rodriguez-Abreu, D., Borghaei, H., … Socinski, M. A. (2017). First-Line Nivolumab in Stage IV or Recurrent Non–Small-Cell Lung Cancer. New England Journal of Medicine, 376(25), 2415–2426. https://doi.org/10.1056/NEJMoa1613493 |
| CheckMate 037 | NCT01721746 | Weber, J. S., D’Angelo, S. P., Minor, D., Hodi, F. S., Gutzmer, R., Neyns, B., Hoeller, C., Khushalani, N. I., Miller, W. H., Lao, C. D., Linette, G. P., Thomas, L., Lorigan, P., Grossmann, K. F., Hassel, J. C., Maio, M., Sznol, M., Ascierto, P. A., Mohr, P., … Larkin, J. (2015). Nivolumab versus chemotherapy in patients with advanced melanoma who progressed after anti-CTLA-4 treatment (CheckMate 037): A randomised, controlled, open-label, phase 3 trial. The Lancet Oncology, 16(4), 375–384. https://doi.org/10.1016/S1470-2045(15)70076-8 |
| CheckMate 057 | NCT01673867 | Borghaei, H., Paz-Ares, L., Horn, L., Spigel, D. R., Steins, M., Ready, N. E., Chow, L. Q., Vokes, E. E., Felip, E., Holgado, E., Barlesi, F., Kohlhäufl, M., Arrieta, O., Burgio, M. A., Fayette, J., Lena, H., Poddubskaya, E., Gerber, D. E., Gettinger, S. N., … Brahmer, J. R. (2015). Nivolumab versus Docetaxel in Advanced Nonsquamous Non–Small-Cell Lung Cancer. New England Journal of Medicine, 373(17), 1627–1639. https://doi.org/10.1056/NEJMoa1507643 |
| CheckMate 066 | NCT01721772 | Robert, C., Long, G. V., Brady, B., Dutriaux, C., Maio, M., Mortier, L., Hassel, J. C., Rutkowski, P., McNeil, C., Kalinka-Warzocha, E., Savage, K. J., Hernberg, M. M., Lebbé, C., Charles, J., Mihalcioiu, C., Chiarion-Sileni, V., Mauch, C., Cognetti, F., Arance, A., … Ascierto, P. A. (2015). Nivolumab in Previously Untreated Melanoma without BRAF Mutation. New England Journal of Medicine, 372(4), 320–330. https://doi.org/10.1056/NEJMoa1412082 |
| CheckMate 067 | NCT01844505 | Larkin, J., Chiarion-Sileni, V., Gonzalez, R., Grob, J. J., Cowey, C. L., Lao, C. D., Schadendorf, D., Dummer, R., Smylie, M., Rutkowski, P., Ferrucci, P. F., Hill, A., Wagstaff, J., Carlino, M. S., Haanen, J. B., Maio, M., Marquez-Rodas, I., McArthur, G. A., Ascierto, P. A., … Wolchok, J. D. (2015). Combined Nivolumab and Ipilimumab or Monotherapy in Untreated Melanoma. New England Journal of Medicine, 373(1), 23–34. https://doi.org/10.1056/NEJMoa1504030 |
| CheckMate 069 | NCT01927419 | Postow, M. A., Chesney, J., Pavlick, A. C., Robert, C., Grossmann, K., McDermott, D., Linette, G. P., Meyer, N., Giguere, J. K., Agarwala, S. S., Shaheen, M., Ernstoff, M. S., Minor, D., Salama, A. K., Taylor, M., Ott, P. A., Rollin, L. M., Horak, C., Gagnier, P., … Hodi, F. S. (2015). Nivolumab and Ipilimumab versus Ipilimumab in Untreated Melanoma. New England Journal of Medicine, 372(21), 2006–2017. https://doi.org/10.1056/NEJMoa1414428 |
| CheckMate 078 | NCT02613507 | Wu, Y.-L., Lu, S., Cheng, Y., Zhou, C., Wang, J., Mok, T., Zhang, L., Tu, H.-Y., Wu, L., Feng, J., Zhang, Y., Luft, A. V., Zhou, J., Ma, Z., Lu, Y., Hu, C., Shi, Y., Baudelet, C., Cai, J., & Chang, J. (2019). Nivolumab Versus Docetaxel in a Predominantly Chinese Patient Population With Previously Treated Advanced NSCLC: CheckMate 078 Randomized Phase III Clinical Trial. Journal of Thoracic Oncology, 14(5), 867–875. https://doi.org/10.1016/j.jtho.2019.01.006 |
| CheckMate 141 | NCT02105636 | Ferris, R. L., Blumenschein, G., Fayette, J., Guigay, J., Colevas, A. D., Licitra, L., Harrington, K., Kasper, S., Vokes, E. E., Even, C., Worden, F., Saba, N. F., Iglesias Docampo, L. C., Haddad, R., Rordorf, T., Kiyota, N., Tahara, M., Monga, M., Lynch, M., … Gillison, M. L. (2016). Nivolumab for Recurrent Squamous-Cell Carcinoma of the Head and Neck. New England Journal of Medicine, 375(19), 1856–1867. https://doi.org/10.1056/NEJMoa1602252 |
| CheckMate 214 | NCT02231749 | Motzer, R. J., Tannir, N. M., McDermott, D. F., Arén Frontera, O., Melichar, B., Choueiri, T. K., Plimack, E. R., Barthélémy, P., Porta, C., George, S., Powles, T., Donskov, F., Neiman, V., Kollmannsberger, C. K., Salman, P., Gurney, H., Hawkins, R., Ravaud, A., Grimm, M.-O., … Escudier, B. (2018). Nivolumab plus Ipilimumab versus Sunitinib in Advanced Renal-Cell Carcinoma. New England Journal of Medicine, 378(14), 1277–1290. https://doi.org/10.1056/NEJMoa1712126 |
| CheckMate 238 | NCT02388906 | Weber, J., Mandala, M., Del Vecchio, M., Gogas, H. J., Arance, A. M., Cowey, C. L., Dalle, S., Schenker, M., Chiarion-Sileni, V., Marquez-Rodas, I., Grob, J.-J., Butler, M. O., Middleton, M. R., Maio, M., Atkinson, V., Queirolo, P., Gonzalez, R., Kudchadkar, R. R., Smylie, M., … Ascierto, P. A. (2017). Adjuvant Nivolumab versus Ipilimumab in Resected Stage III or IV Melanoma. New England Journal of Medicine, 377(19), 1824–1835. https://doi.org/10.1056/NEJMoa1709030 |
| CheckMate 331 | NCT02481830 | Spigel, D. R., Vicente, D., Ciuleanu, T. E., Gettinger, S., Peters, S., Horn, L., Audigier-Valette, C., Pardo Aranda, N., Juan-Vidal, O., Cheng, Y., Zhang, H., Shi, M., Luft, A., Wolf, J., Antonia, S., Nakagawa, K., Fairchild, J., Baudelet, C., Pandya, D., … Reck, M. (2021). Second-line nivolumab in relapsed small-cell lung cancer: CheckMate 331☆. Annals of Oncology, 32(5), 631–641. https://doi.org/10.1016/j.annonc.2021.01.071 |
| CheckMate 451 | NCT02538666 | A Randomized, Multicenter, Double-Blind, Phase 3 Study of Nivolumab, Nivolumab in Combination With Ipilimumab, or Placebo as Maintenance Therapy in Subjects With Extensive-Stage Disease Small Cell Lung Cancer (ED-SCLC) After Completion of Platinum-based First Line Chemotherapy (CheckMate 451: CHECKpoint Pathway and nivoluMAb Clinical Trial Evaluation 451) (Clinical Trial Registration No. NCT02538666). clinicaltrials.gov. Retrieved 13 December 2021, from https://clinicaltrials.gov/ct2/show/NCT02538666 |
| CheckMate 743 | NCT02899299 | Baas, P., Scherpereel, A., Nowak, A. K., Fujimoto, N., Peters, S., Tsao, A. S., Mansfield, A. S., Popat, S., Jahan, T., Antonia, S., Oulkhouir, Y., Bautista, Y., Cornelissen, R., Greillier, L., Grossi, F., Kowalski, D., Rodríguez-Cid, J., Aanur, P., Oukessou, A., … Zalcman, G. (2021). First-line nivolumab plus ipilimumab in unresectable malignant pleural mesothelioma (CheckMate 743): A multicentre, randomised, open-label, phase 3 trial. The Lancet, 397(10272), 375–386. https://doi.org/10.1016/S0140-6736(20)32714-8 |
| CheckMate 9LA | NCT03215706 | Paz-Ares, L., Ciuleanu, T.-E., Cobo, M., Schenker, M., Zurawski, B., Menezes, J., Richardet, E., Bennouna, J., Felip, E., Juan-Vidal, O., Alexandru, A., Sakai, H., Lingua, A., Salman, P., Souquet, P.-J., De Marchi, P., Martin, C., Pérol, M., Scherpereel, A., … Reck, M. (2021). First-line nivolumab plus ipilimumab combined with two cycles of chemotherapy in patients with non-small-cell lung cancer (CheckMate 9LA): An international, randomised, open-label, phase 3 trial. The Lancet Oncology, 22(2), 198–211. https://doi.org/10.1016/S1470-2045(20)30641-0 |
| CheckMate 143 | NCT02017717 | Omuro, A., Vlahovic, G., Lim, M., Sahebjam, S., Baehring, J., Cloughesy, T., Voloschin, A., Ramkissoon, S. H., Ligon, K. L., Latek, R., Zwirtes, R., Strauss, L., Paliwal, P., Harbison, C. T., Reardon, D. A., & Sampson, J. H. (2018). Nivolumab with or without ipilimumab in patients with recurrent glioblastoma: Results from exploratory phase I cohorts of CheckMate 143. Neuro-Oncology, 20(5), 674–686. https://doi.org/10.1093/neuonc/nox208 |
| CheckMate 143 Cohort 2 | NCT02017717 | Reardon, D. A., Brandes, A. A., Omuro, A., Mulholland, P., Lim, M., Wick, A., Baehring, J., Ahluwalia, M. S., Roth, P., Bähr, O., Phuphanich, S., Sepulveda, J. M., De Souza, P., Sahebjam, S., Carleton, M., Tatsuoka, K., Taitt, C., Zwirtes, R., Sampson, J., & Weller, M. (2020). Effect of Nivolumab vs Bevacizumab in Patients With Recurrent Glioblastoma: The CheckMate 143 Phase 3 Randomized Clinical Trial. JAMA Oncology, 6(7), 1003. https://doi.org/10.1001/jamaoncol.2020.1024 |
| CONDOR | NCT02319044 | Siu, L. L., Even, C., Mesía, R., Remenar, E., Daste, A., Delord, J.-P., Krauss, J., Saba, N. F., Nabell, L., Ready, N. E., Braña, I., Kotecki, N., Zandberg, D. P., Gilbert, J., Mehanna, H., Bonomi, M., Jarkowski, A., Melillo, G., Armstrong, J. M., … Fayette, J. (2019). Safety and Efficacy of Durvalumab With or Without Tremelimumab in Patients With PD-L1–Low/Negative Recurrent or Metastatic HNSCC: The Phase 2 CONDOR Randomized Clinical Trial. JAMA Oncology, 5(2), 195. https://doi.org/10.1001/jamaoncol.2018.4628 |
| DANUBE | NCT02516241 | Powles, T., van der Heijden, M. S., Castellano, D., Galsky, M. D., Loriot, Y., Petrylak, D. P., Ogawa, O., Park, S. H., Lee, J.-L., De Giorgi, U., Bögemann, M., Bamias, A., Eigl, B. J., Gurney, H., Mukherjee, S. D., Fradet, Y., Skoneczna, I., Tsiatas, M., Novikov, A., … Lesniewski-Kmak, K. (2020). Durvalumab alone and durvalumab plus tremelimumab versus chemotherapy in previously untreated patients with unresectable, locally advanced or metastatic urothelial carcinoma (DANUBE): A randomised, open-label, multicentre, phase 3 trial. The Lancet Oncology, 21(12), 1574–1588. https://doi.org/10.1016/S1470-2045(20)30541-6 |
| EAGLE | NCT02369874 | Ferris, R. L., Haddad, R., Even, C., Tahara, M., Dvorkin, M., Ciuleanu, T. E., Clement, P. M., Mesia, R., Kutukova, S., Zholudeva, L., Daste, A., Caballero-Daroqui, J., Keam, B., Vynnychenko, I., Lafond, C., Shetty, J., Mann, H., Fan, J., Wildsmith, S., … Licitra, L. (2020). Durvalumab with or without tremelimumab in patients with recurrent or metastatic head and neck squamous cell carcinoma: EAGLE, a randomized, open-label phase III study. Annals of Oncology, 31(7), 942–950. https://doi.org/10.1016/j.annonc.2020.04.001 |
| EORTC 18071 | NCT00636168 | Eggermont, A. M. M., Chiarion-Sileni, V., Grob, J.-J., Dummer, R., Wolchok, J. D., Schmidt, H., Hamid, O., Robert, C., Ascierto, P. A., Richards, J. M., Lebbé, C., Ferraresi, V., Smylie, M., Weber, J. S., Maio, M., Bastholt, L., Mortier, L., Thomas, L., Tahir, S., … Testori, A. (2016). Prolonged Survival in Stage III Melanoma with Ipilimumab Adjuvant Therapy. New England Journal of Medicine, 375(19), 1845–1855. https://doi.org/10.1056/NEJMoa1611299 |
| GeparNuevo | NCT02685059 | Loibl, S., Untch, M., Burchardi, N., Huober, J., Sinn, B. V., Blohmer, J.-U., Grischke, E.-M., Furlanetto, J., Tesch, H., Hanusch, C., Engels, K., Rezai, M., Jackisch, C., Schmitt, W. D., von Minckwitz, G., Thomalla, J., Kümmel, S., Rautenberg, B., Fasching, P. A., … Schneeweiss, A. (2019). A randomised phase II study investigating durvalumab in addition to an anthracycline taxane-based neoadjuvant therapy in early triple-negative breast cancer: Clinical results and biomarker analysis of GeparNuevo study. Annals of Oncology, 30(8), 1279–1288. https://doi.org/10.1093/annonc/mdz158 |
| IMblaze370 | NCT02788279 | Eng, C., Kim, T. W., Bendell, J., Argilés, G., Tebbutt, N. C., Di Bartolomeo, M., Falcone, A., Fakih, M., Kozloff, M., Segal, N. H., Sobrero, A., Yan, Y., Chang, I., Uyei, A., Roberts, L., Ciardiello, F., Ahn, J., Asselah, J., Badarinath, S., … Young, R. (2019). Atezolizumab with or without cobimetinib versus regorafenib in previously treated metastatic colorectal cancer (IMblaze370): A multicentre, open-label, phase 3, randomised, controlled trial. The Lancet Oncology, 20(6), 849–861. https://doi.org/10.1016/S1470-2045(19)30027-0 |
| IMmotion150 | NCT01984242 | McDermott, D. F., Huseni, M. A., Atkins, M. B., Motzer, R. J., Rini, B. I., Escudier, B., Fong, L., Joseph, R. W., Pal, S. K., Reeves, J. A., Sznol, M., Hainsworth, J., Rathmell, W. K., Stadler, W. M., Hutson, T., Gore, M. E., Ravaud, A., Bracarda, S., Suárez, C., … Powles, T. (2018). Clinical activity and molecular correlates of response to atezolizumab alone or in combination with bevacizumab versus sunitinib in renal cell carcinoma. Nature Medicine, 24(6), 749–757. https://doi.org/10.1038/s41591-018-0053-3 |
| IMMUNED | NCT02523313 | Zimmer, L., Livingstone, E., Hassel, J. C., Fluck, M., Eigentler, T., Loquai, C., Haferkamp, S., Gutzmer, R., Meier, F., Mohr, P., Hauschild, A., Schilling, B., Menzer, C., Kieker, F., Dippel, E., Rösch, A., Simon, J.-C., Conrad, B., Körner, S., … Utikal, J. (2020). Adjuvant nivolumab plus ipilimumab or nivolumab monotherapy versus placebo in patients with resected stage IV melanoma with no evidence of disease (IMMUNED): A randomised, double-blind, placebo-controlled, phase 2 trial. The Lancet, 395(10236), 1558–1568. https://doi.org/10.1016/S0140-6736(20)30417-7 |
| IMpassion031 | NCT03197935 | Mittendorf, E. A., Zhang, H., Barrios, C. H., Saji, S., Jung, K. H., Hegg, R., Koehler, A., Sohn, J., Iwata, H., Telli, M. L., Ferrario, C., Punie, K., Penault-Llorca, F., Patel, S., Duc, A. N., Liste-Hermoso, M., Maiya, V., Molinero, L., Chui, S. Y., & Harbeck, N. (2020). Neoadjuvant atezolizumab in combination with sequential nab-paclitaxel and anthracycline-based chemotherapy versus placebo and chemotherapy in patients with early-stage triple-negative breast cancer (IMpassion031): A randomised, double-blind, phase 3 trial. The Lancet, 396(10257), 1090–1100. https://doi.org/10.1016/S0140-6736(20)31953-X |
| IMpassion130 | NCT02425891 | Schmid, P., Adams, S., Rugo, H. S., Schneeweiss, A., Barrios, C. H., Iwata, H., Diéras, V., Hegg, R., Im, S.-A., Shaw Wright, G., Henschel, V., Molinero, L., Chui, S. Y., Funke, R., Husain, A., Winer, E. P., Loi, S., & Emens, L. A. (2018). Atezolizumab and Nab-Paclitaxel in Advanced Triple-Negative Breast Cancer. New England Journal of Medicine, 379(22), 2108–2121. https://doi.org/10.1056/NEJMoa1809615 |
| IMpower110 | NCT02409342 | Herbst, R. S., Giaccone, G., de Marinis, F., Reinmuth, N., Vergnenegre, A., Barrios, C. H., Morise, M., Felip, E., Andric, Z., Geater, S., Özgüroğlu, M., Zou, W., Sandler, A., Enquist, I., Komatsubara, K., Deng, Y., Kuriki, H., Wen, X., McCleland, M., … Spigel, D. R. (2020). Atezolizumab for First-Line Treatment of PD-L1–Selected Patients with NSCLC. New England Journal of Medicine, 383(14), 1328–1339. https://doi.org/10.1056/NEJMoa1917346 |
| IMpower130 | NCT02367781 | West, H., McCleod, M., Hussein, M., Morabito, A., Rittmeyer, A., Conter, H. J., Kopp, H.-G., Daniel, D., McCune, S., Mekhail, T., Zer, A., Reinmuth, N., Sadiq, A., Sandler, A., Lin, W., Ochi Lohmann, T., Archer, V., Wang, L., Kowanetz, M., & Cappuzzo, F. (2019). Atezolizumab in combination with carboplatin plus nab-paclitaxel chemotherapy compared with chemotherapy alone as first-line treatment for metastatic non-squamous non-small-cell lung cancer (IMpower130): A multicentre, randomised, open-label, phase 3 trial. The Lancet Oncology, 20(7), 924–937. https://doi.org/10.1016/S1470-2045(19)30167-6 |
| IMpower132 | NCT02657434 | Nishio, M., Barlesi, F., West, H., Ball, S., Bordoni, R., Cobo, M., Longeras, P. D., Goldschmidt, J., Novello, S., Orlandi, F., Sanborn, R. E., Szalai, Z., Ursol, G., Mendus, D., Wang, L., Wen, X., McCleland, M., Hoang, T., Phan, S., & Socinski, M. A. (2021). Atezolizumab Plus Chemotherapy for First-Line Treatment of Nonsquamous NSCLC: Results From the Randomized Phase 3 IMpower132 Trial. Journal of Thoracic Oncology, 16(4), 653–664. https://doi.org/10.1016/j.jtho.2020.11.025 |
| IMpower133 | NCT02763579 | Horn, L., Mansfield, A. S., Szczęsna, A., Havel, L., Krzakowski, M., Hochmair, M. J., Huemer, F., Losonczy, G., Johnson, M. L., Nishio, M., Reck, M., Mok, T., Lam, S., Shames, D. S., Liu, J., Ding, B., Lopez-Chavez, A., Kabbinavar, F., Lin, W., … Liu, S. V. (2018). First-Line Atezolizumab plus Chemotherapy in Extensive-Stage Small-Cell Lung Cancer. New England Journal of Medicine, 379(23), 2220–2229. https://doi.org/10.1056/NEJMoa1809064 |
| IMpower150 | NCT02366143 | Socinski, M. A., Jotte, R. M., Cappuzzo, F., Orlandi, F., Stroyakovskiy, D., Nogami, N., Rodríguez-Abreu, D., Moro-Sibilot, D., Thomas, C. A., Barlesi, F., Finley, G., Kelsch, C., Lee, A., Coleman, S., Deng, Y., Shen, Y., Kowanetz, M., Lopez-Chavez, A., Sandler, A., & Reck, M. (2018). Atezolizumab for First-Line Treatment of Metastatic Nonsquamous NSCLC. New England Journal of Medicine, 378(24), 2288–2301. https://doi.org/10.1056/NEJMoa1716948 |
| IMspire150 | NCT02908672 | Gutzmer, R., Stroyakovskiy, D., Gogas, H., Robert, C., Lewis, K., Protsenko, S., Pereira, R. P., Eigentler, T., Rutkowski, P., Demidov, L., Manikhas, G. M., Yan, Y., Huang, K.-C., Uyei, A., McNally, V., McArthur, G. A., & Ascierto, P. A. (2020). Atezolizumab, vemurafenib, and cobimetinib as first-line treatment for unresectable advanced BRAFV600 mutation-positive melanoma (IMspire150): Primary analysis of the randomised, double-blind, placebo-controlled, phase 3 trial. The Lancet, 395(10240), 1835–1844. https://doi.org/10.1016/S0140-6736(20)30934-X |
| IMspire170 | NCT03273153 | Gogas, H., Dréno, B., Larkin, J., Demidov, L., Stroyakovskiy, D., Eroglu, Z., Francesco Ferrucci, P., Pigozzo, J., Rutkowski, P., Mackiewicz, J., Rooney, I., Voulgari, A., Troutman, S., Pitcher, B., Guo, Y., Yan, Y., Castro, M., Mulla, S., Flaherty, K., & Arance, A. (2021). Cobimetinib plus atezolizumab in BRAFV600 wild-type melanoma: Primary results from the randomized phase III IMspire170 study. Annals of Oncology, 32(3), 384–394. https://doi.org/10.1016/j.annonc.2020.12.004 |
| IMvigor130 | NCT02807636 | Galsky, M. D., Arija, J. Á. A., Bamias, A., Davis, I. D., De Santis, M., Kikuchi, E., Garcia-del-Muro, X., De Giorgi, U., Mencinger, M., Izumi, K., Panni, S., Gumus, M., Özgüroğlu, M., Kalebasty, A. R., Park, S. H., Alekseev, B., Schutz, F. A., Li, J.-R., Ye, D., … Grande, E. (2020). Atezolizumab with or without chemotherapy in metastatic urothelial cancer (IMvigor130): A multicentre, randomised, placebo-controlled phase 3 trial. The Lancet, 395(10236), 1547–1557. https://doi.org/10.1016/S0140-6736(20)30230-0 |
| IMvigor211 | NCT02302807 | Powles, T., Durán, I., van der Heijden, M. S., Loriot, Y., Vogelzang, N. J., De Giorgi, U., Oudard, S., Retz, M. M., Castellano, D., Bamias, A., Fléchon, A., Gravis, G., Hussain, S., Takano, T., Leng, N., Kadel, E. E., Banchereau, R., Hegde, P. S., Mariathasan, S., … Ravaud, A. (2018). Atezolizumab versus chemotherapy in patients with platinum-treated locally advanced or metastatic urothelial carcinoma (IMvigor211): A multicentre, open-label, phase 3 randomised controlled trial. The Lancet, 391(10122), 748–757. https://doi.org/10.1016/S0140-6736(17)33297-X |
| I-SPY | NCT01042379 | Nanda, R., Liu, M. C., Yau, C., Shatsky, R., Pusztai, L., Wallace, A., Chien, A. J., Forero-Torres, A., Ellis, E., Han, H., Clark, A., Albain, K., Boughey, J. C., Jaskowiak, N. T., Elias, A., Isaacs, C., Kemmer, K., Helsten, T., Majure, M., … Esserman, L. J. (2020). Effect of Pembrolizumab Plus Neoadjuvant Chemotherapy on Pathologic Complete Response in Women With Early-Stage Breast Cancer: An Analysis of the Ongoing Phase 2 Adaptively Randomized I-SPY2 Trial. JAMA Oncology, 6(5), 676. https://doi.org/10.1001/jamaoncol.2019.6650 |
| JAVELIN Bladder 100 | NCT02603432 | Powles, T., Park, S. H., Voog, E., Caserta, C., Valderrama, B. P., Gurney, H., Kalofonos, H., Radulović, S., Demey, W., Ullén, A., Loriot, Y., Sridhar, S. S., Tsuchiya, N., Kopyltsov, E., Sternberg, C. N., Bellmunt, J., Aragon-Ching, J. B., Petrylak, D. P., Laliberte, R., … Grivas, P. (2020). Avelumab Maintenance Therapy for Advanced or Metastatic Urothelial Carcinoma. New England Journal of Medicine, 383(13), 1218–1230. https://doi.org/10.1056/NEJMoa2002788 |
| JAVELIN Gastric 100 | NCT02625610 | Moehler, M., Dvorkin, M., Boku, N., Özgüroğlu, M., Ryu, M.-H., Muntean, A. S., Lonardi, S., Nechaeva, M., Bragagnoli, A. C., Coşkun, H. S., Cubillo Gracian, A., Takano, T., Wong, R., Safran, H., Vaccaro, G. M., Wainberg, Z. A., Silver, M. R., Xiong, H., Hong, J., … Bang, Y.-J. (2021). Phase III Trial of Avelumab Maintenance After First-Line Induction Chemotherapy Versus Continuation of Chemotherapy in Patients With Gastric Cancers: Results From JAVELIN Gastric 100. Journal of Clinical Oncology, 39(9), 966–977. https://doi.org/10.1200/JCO.20.00892 |
| JAVELIN Gastric 300 | NCT02625623 | Bang, Y.-J., Ruiz, E. Y., Van Cutsem, E., Lee, K.-W., Wyrwicz, L., Schenker, M., Alsina, M., Ryu, M.-H., Chung, H.-C., Evesque, L., Al-Batran, S.-E., Park, S. H., Lichinitser, M., Boku, N., Moehler, M. H., Hong, J., Xiong, H., Hallwachs, R., Conti, I., & Taieb, J. (2018). Phase III, randomised trial of avelumab versus physician’s choice of chemotherapy as third-line treatment of patients with advanced gastric or gastro-oesophageal junction cancer: Primary analysis of JAVELIN Gastric 300. Annals of Oncology, 29(10), 2052–2060. https://doi.org/10.1093/annonc/mdy264 |
| JAVELIN Lung 200 | NCT02395172 | Barlesi, F., Vansteenkiste, J., Spigel, D., Ishii, H., Garassino, M., de Marinis, F., Özgüroğlu, M., Szczesna, A., Polychronis, A., Uslu, R., Krzakowski, M., Lee, J.-S., Calabrò, L., Arén Frontera, O., Ellers-Lenz, B., Bajars, M., Ruisi, M., & Park, K. (2018). Avelumab versus docetaxel in patients with platinum-treated advanced non-small-cell lung cancer (JAVELIN Lung 200): An open-label, randomised, phase 3 study. The Lancet Oncology, 19(11), 1468–1479. https://doi.org/10.1016/S1470-2045(18)30673-9 |
| JAVELIN Ovarian 100 | NCT02718417 | A randomized, open-label, multicenter, phase 3 study to evaluate the efficacy and safety of avelumab (MSB0010718C) in combination with and/or following chemotherapy in patients with previously untreated epithelial ovarian cancer JAVELIN Ovarian 100 (Clinical Trial Registration No. NCT02718417). clinicaltrials.gov. Retrieved 13 December 2021, from https://clinicaltrials.gov/ct2/show/NCT02718417 |
| JAVELIN Ovarian 200 | NCT02580058 | A Study Of Avelumab Alone Or In Combination With Pegylated Liposomal Doxorubicin Versus Pegylated Liposomal Doxorubicin Alone In Patients With Platinum Resistant/Refractory Ovarian Cancer (JAVELIN Ovarian 200) (Clinical Trial Registration No. NCT02580058). clinicaltrials.gov. Retrieved 13 December 2021, from https://clinicaltrials.gov/ct2/show/NCT02580058 |
| KATE2 | NCT02924883 | Emens, L. A., Esteva, F. J., Beresford, M., Saura, C., De Laurentiis, M., Kim, S.-B., Im, S.-A., Wang, Y., Salgado, R., Mani, A., Shah, J., Lambertini, C., Liu, H., de Haas, S. L., Patre, M., & Loi, S. (2020). Trastuzumab emtansine plus atezolizumab versus trastuzumab emtansine plus placebo in previously treated, HER2-positive advanced breast cancer (KATE2): A phase 2, multicentre, randomised, double-blind trial. The Lancet Oncology, 21(10), 1283–1295. https://doi.org/10.1016/S1470-2045(20)30465-4 |
| KEYNOTE-006 | NCT01866319 | Schachter, J., Ribas, A., Long, G. V., Arance, A., Grob, J.-J., Mortier, L., Daud, A., Carlino, M. S., McNeil, C., Lotem, M., Larkin, J., Lorigan, P., Neyns, B., Blank, C., Petrella, T. M., Hamid, O., Zhou, H., Ebbinghaus, S., Ibrahim, N., & Robert, C. (2017). Pembrolizumab versus ipilimumab for advanced melanoma: Final overall survival results of a multicentre, randomised, open-label phase 3 study (KEYNOTE-006). The Lancet, 390(10105), 1853–1862. https://doi.org/10.1016/S0140-6736(17)31601-X |
| KEYNOTE-010 | NCT01905657 | Herbst, R. S., Baas, P., Kim, D.-W., Felip, E., Pérez-Gracia, J. L., Han, J.-Y., Molina, J., Kim, J.-H., Arvis, C. D., Ahn, M.-J., Majem, M., Fidler, M. J., de Castro, G., Garrido, M., Lubiniecki, G. M., Shentu, Y., Im, E., Dolled-Filhart, M., & Garon, E. B. (2016). Pembrolizumab versus docetaxel for previously treated, PD-L1-positive, advanced non-small-cell lung cancer (KEYNOTE-010): A randomised controlled trial. The Lancet, 387(10027), 1540–1550. https://doi.org/10.1016/S0140-6736(15)01281-7 |
| KEYNOTE-021 | NCT02039674 | Langer, C. J., Gadgeel, S. M., Borghaei, H., Papadimitrakopoulou, V. A., Patnaik, A., Powell, S. F., Gentzler, R. D., Martins, R. G., Stevenson, J. P., Jalal, S. I., Panwalkar, A., Yang, J. C.-H., Gubens, M., Sequist, L. V., Awad, M. M., Fiore, J., Ge, Y., Raftopoulos, H., & Gandhi, L. (2016). Carboplatin and pemetrexed with or without pembrolizumab for advanced, non-squamous non-small-cell lung cancer: A randomised, phase 2 cohort of the open-label KEYNOTE-021 study. The Lancet Oncology, 17(11), 1497–1508. https://doi.org/10.1016/S1470-2045(16)30498-3 |
| KEYNOTE-024 | NCT02142738 | Reck, M., Rodríguez-Abreu, D., Robinson, A. G., Hui, R., Csőszi, T., Fülöp, A., Gottfried, M., Peled, N., Tafreshi, A., Cuffe, S., O’Brien, M., Rao, S., Hotta, K., Leiby, M. A., Lubiniecki, G. M., Shentu, Y., Rangwala, R., & Brahmer, J. R. (2016). Pembrolizumab versus Chemotherapy for PD-L1–Positive Non–Small-Cell Lung Cancer. New England Journal of Medicine, 375(19), 1823–1833. https://doi.org/10.1056/NEJMoa1606774 |
| KEYNOTE-040 | NCT02252042 | Cohen, E. E. W., Soulières, D., Le Tourneau, C., Dinis, J., Licitra, L., Ahn, M.-J., Soria, A., Machiels, J.-P., Mach, N., Mehra, R., Burtness, B., Zhang, P., Cheng, J., Swaby, R. F., Harrington, K. J., Acosta-Rivera, M., Adkins, D. R., Aghmesheh, M., Ahn, M.-J., … Zandberg, D. (2019). Pembrolizumab versus methotrexate, docetaxel, or cetuximab for recurrent or metastatic head-and-neck squamous cell carcinoma (KEYNOTE-040): A randomised, open-label, phase 3 study. The Lancet, 393(10167), 156–167. https://doi.org/10.1016/S0140-6736(18)31999-8 |
| KEYNOTE-042 | NCT02220894 | Mok, T. S. K., Wu, Y.-L., Kudaba, I., Kowalski, D. M., Cho, B. C., Turna, H. Z., Castro, G., Srimuninnimit, V., Laktionov, K. K., Bondarenko, I., Kubota, K., Lubiniecki, G. M., Zhang, J., Kush, D., Lopes, G., Adamchuk, G., Ahn, M.-J., Alexandru, A., Altundag, O., … Zippelius, A. (2019). Pembrolizumab versus chemotherapy for previously untreated, PD-L1-expressing, locally advanced or metastatic non-small-cell lung cancer (KEYNOTE-042): A randomised, open-label, controlled, phase 3 trial. The Lancet, 393(10183), 1819–1830. https://doi.org/10.1016/S0140-6736(18)32409-7 |
| KEYNOTE-045 | NCT02256436 | Bellmunt, J., de Wit, R., Vaughn, D. J., Fradet, Y., Lee, J.-L., Fong, L., Vogelzang, N. J., Climent, M. A., Petrylak, D. P., Choueiri, T. K., Necchi, A., Gerritsen, W., Gurney, H., Quinn, D. I., Culine, S., Sternberg, C. N., Mai, Y., Poehlein, C. H., Perini, R. F., & Bajorin, D. F. (2017). Pembrolizumab as Second-Line Therapy for Advanced Urothelial Carcinoma. New England Journal of Medicine, 376(11), 1015–1026. https://doi.org/10.1056/NEJMoa1613683 |
| KEYNOTE-048 | NCT02358031 | Burtness, B., Harrington, K. J., Greil, R., Soulières, D., Tahara, M., de Castro, G., Psyrri, A., Basté, N., Neupane, P., Bratland, Å., Fuereder, T., Hughes, B. G. M., Mesía, R., Ngamphaiboon, N., Rordorf, T., Wan Ishak, W. Z., Hong, R.-L., González Mendoza, R., Roy, A., … Yorio, J. (2019). Pembrolizumab alone or with chemotherapy versus cetuximab with chemotherapy for recurrent or metastatic squamous cell carcinoma of the head and neck (KEYNOTE-048): A randomised, open-label, phase 3 study. The Lancet, 394(10212), 1915–1928. https://doi.org/10.1016/S0140-6736(19)32591-7 |
| KEYNOTE-054 | NCT02362594 | Eggermont, A. M. M., Blank, C. U., Mandala, M., Long, G. V., Atkinson, V., Dalle, S., Haydon, A., Lichinitser, M., Khattak, A., Carlino, M. S., Sandhu, S., Larkin, J., Puig, S., Ascierto, P. A., Rutkowski, P., Schadendorf, D., Koornstra, R., Hernandez-Aya, L., Maio, M., … Robert, C. (2018). Adjuvant Pembrolizumab versus Placebo in Resected Stage III Melanoma. New England Journal of Medicine, 378(19), 1789–1801. https://doi.org/10.1056/NEJMoa1802357 |
| KEYNOTE-061 | NCT02370498 | Shitara, K., Özgüroğlu, M., Bang, Y.-J., Di Bartolomeo, M., Mandalà, M., Ryu, M.-H., Fornaro, L., Olesiński, T., Caglevic, C., Chung, H. C., Muro, K., Goekkurt, E., Mansoor, W., McDermott, R. S., Shacham-Shmueli, E., Chen, X., Mayo, C., Kang, S. P., Ohtsu, A., … Riche, S. L. (2018). Pembrolizumab versus paclitaxel for previously treated, advanced gastric or gastro-oesophageal junction cancer (KEYNOTE-061): A randomised, open-label, controlled, phase 3 trial. The Lancet, 392(10142), 123–133. https://doi.org/10.1016/S0140-6736(18)31257-1 |
| KEYNOTE-062 | NCT02494583 | Shitara, K., Van Cutsem, E., Bang, Y.-J., Fuchs, C., Wyrwicz, L., Lee, K.-W., Kudaba, I., Garrido, M., Chung, H. C., Lee, J., Castro, H. R., Mansoor, W., Braghiroli, M. I., Karaseva, N., Caglevic, C., Villanueva, L., Goekkurt, E., Satake, H., Enzinger, P., … Tabernero, J. (2020). Efficacy and Safety of Pembrolizumab or Pembrolizumab Plus Chemotherapy vs Chemotherapy Alone for Patients With First-line, Advanced Gastric Cancer: The KEYNOTE-062 Phase 3 Randomized Clinical Trial. JAMA Oncology, 6(10), 1571. https://doi.org/10.1001/jamaoncol.2020.3370 |
| KEYNOTE-119 | NCT02555657 | A Randomized Open-Label Phase III Study of Single Agent Pembrolizumab Versus Single Agent Chemotherapy Per Physician’s Choice for Metastatic Triple Negative Breast Cancer (mTNBC)—(KEYNOTE-119) (Clinical Trial Registration No. NCT02555657). clinicaltrials.gov. Retrieved 13 December 2021, from https://clinicaltrials.gov/ct2/show/NCT02555657 |
| KEYNOTE-144 | NCT02362048 | Overman, M., Javle, M., Davis, R. E., Vats, P., Kumar-Sinha, C., Xiao, L., Mettu, N. B., Parra, E. R., Benson, A. B., Lopez, C. D., Munugalavadla, V., Patel, P., Tao, L., Neelapu, S., & Maitra, A. (2020). Randomized phase II study of the Bruton tyrosine kinase inhibitor acalabrutinib, alone or with pembrolizumab in patients with advanced pancreatic cancer. Journal for ImmunoTherapy of Cancer, 8(1), e000587. https://doi.org/10.1136/jitc-2020-000587 |
| KEYNOTE-177 | NCT02563002 | André, T., Shiu, K.-K., Kim, T. W., Jensen, B. V., Jensen, L. H., Punt, C., Smith, D., Garcia-Carbonero, R., Benavides, M., Gibbs, P., de la Fouchardiere, C., Rivera, F., Elez, E., Bendell, J., Le, D. T., Yoshino, T., Van Cutsem, E., Yang, P., Farooqui, M. Z. H., … Diaz, L. A. (2020). Pembrolizumab in Microsatellite-Instability–High Advanced Colorectal Cancer. New England Journal of Medicine, 383(23), 2207–2218. https://doi.org/10.1056/NEJMoa2017699 |
| KEYNOTE-181 | NCT02564263 | Kojima, T., Shah, M. A., Muro, K., Francois, E., Adenis, A., Hsu, C.-H., Doi, T., Moriwaki, T., Kim, S.-B., Lee, S.-H., Bennouna, J., Kato, K., Shen, L., Enzinger, P., Qin, S.-K., Ferreira, P., Chen, J., Girotto, G., de la Fouchardiere, C., … on behalf of the KEYNOTE-181 Investigators. (2020). Randomized Phase III KEYNOTE-181 Study of Pembrolizumab Versus Chemotherapy in Advanced Esophageal Cancer. Journal of Clinical Oncology, 38(35), 4138–4148. https://doi.org/10.1200/JCO.20.01888 |
| KEYNOTE-183 | NCT02576977 | A Phase III Study of Pomalidomide and Low Dose Dexamethasone With or Without Pembrolizumab (MK3475) in Refractory or Relapsed and Refractory Multiple Myeloma (rrMM) (KEYNOTE 183) (Clinical Trial Registration No. NCT02576977). clinicaltrials.gov. Retrieved 13 December 2021, from https://clinicaltrials.gov/ct2/show/NCT02576977 |
| KEYNOTE-185 | NCT02579863 | Usmani, S. Z., Schjesvold, F., Oriol, A., Karlin, L., Cavo, M., Rifkin, R. M., Yimer, H. A., LeBlanc, R., Takezako, N., McCroskey, R. D., Lim, A. B. M., Suzuki, K., Kosugi, H., Grigoriadis, G., Avivi, I., Facon, T., Jagannath, S., Lonial, S., Ghori, R. U., … KEYNOTE-185 Investigators. (2019). Pembrolizumab plus lenalidomide and dexamethasone for patients with treatment-naive multiple myeloma (KEYNOTE-185): A randomised, open-label, phase 3 trial. The Lancet. Haematology, 6(9), e448–e458. https://doi.org/10.1016/S2352-3026(19)30109-7 |
| KEYNOTE-189 | NCT02578680 | Gandhi, L., Rodríguez-Abreu, D., Gadgeel, S., Esteban, E., Felip, E., De Angelis, F., Domine, M., Clingan, P., Hochmair, M. J., Powell, S. F., Cheng, S. Y.-S., Bischoff, H. G., Peled, N., Grossi, F., Jennens, R. R., Reck, M., Hui, R., Garon, E. B., Boyer, M., … Garassino, M. C. (2018). Pembrolizumab plus Chemotherapy in Metastatic Non–Small-Cell Lung Cancer. New England Journal of Medicine, 378(22), 2078–2092. https://doi.org/10.1056/NEJMoa1801005 |
| KEYNOTE-240 | NCT02702401 | Finn, R. S., Ryoo, B.-Y., Merle, P., Kudo, M., Bouattour, M., Lim, H. Y., Breder, V., Edeline, J., Chao, Y., Ogasawara, S., Yau, T., Garrido, M., Chan, S. L., Knox, J., Daniele, B., Ebbinghaus, S. W., Chen, E., Siegel, A. B., Zhu, A. X., … on behalf of the KEYNOTE-240 investigators. (2020). Pembrolizumab As Second-Line Therapy in Patients With Advanced Hepatocellular Carcinoma in KEYNOTE-240: A Randomized, Double-Blind, Phase III Trial. Journal of Clinical Oncology, 38(3), 193–202. https://doi.org/10.1200/JCO.19.01307 |
| KEYNOTE-355 | NCT02819518 | Cortes, J., Cescon, D. W., Rugo, H. S., Nowecki, Z., Im, S.-A., Yusof, M. M., Gallardo, C., Lipatov, O., Barrios, C. H., Holgado, E., Iwata, H., Masuda, N., Otero, M. T., Gokmen, E., Loi, S., Guo, Z., Zhao, J., Aktan, G., Karantza, V., … Frances, V.-A. (2020). Pembrolizumab plus chemotherapy versus placebo plus chemotherapy for previously untreated locally recurrent inoperable or metastatic triple-negative breast cancer (KEYNOTE-355): A randomised, placebo-controlled, double-blind, phase 3 clinical trial. The Lancet, 396(10265), 1817–1828. https://doi.org/10.1016/S0140-6736(20)32531-9 |
| KEYNOTE-407 | NCT02775435 | Paz-Ares, L., Luft, A., Vicente, D., Tafreshi, A., Gümüş, M., Mazières, J., Hermes, B., Çay Şenler, F., Csőszi, T., Fülöp, A., Rodríguez-Cid, J., Wilson, J., Sugawara, S., Kato, T., Lee, K. H., Cheng, Y., Novello, S., Halmos, B., Li, X., … Kowalski, D. M. (2018). Pembrolizumab plus Chemotherapy for Squamous Non–Small-Cell Lung Cancer. New England Journal of Medicine, 379(21), 2040–2051. https://doi.org/10.1056/NEJMoa1810865 |
| KEYNOTE-590 | NCT03189719 | A Randomized, Double-Blind, Placebo-Controlled Phase III Clinical Trial of Pembrolizumab (MK-3475) in Combination With Cisplatin and 5-Fluorouracil Versus Placebo in Combination With Cisplatin and 5-Fluorouracil as First-Line Treatment in Subjects With Advanced/Metastatic Esophageal Carcinoma (KEYNOTE-590) (Clinical Trial Registration No. NCT03189719). clinicaltrials.gov. Retrieved 13 December 2021, from https://clinicaltrials.gov/ct2/show/NCT03189719 |
| KEYNOTE-598 | NCT03302234 | Boyer, M., Şendur, M. A. N., Rodríguez-Abreu, D., Park, K., Lee, D. H., Çiçin, I., Yumuk, P. F., Orlandi, F. J., Leal, T. A., Molinier, O., Soparattanapaisarn, N., Langleben, A., Califano, R., Medgyasszay, B., Hsia, T.-C., Otterson, G. A., Xu, L., Piperdi, B., Samkari, A., … for the KEYNOTE-598 Investigators. (2021). Pembrolizumab Plus Ipilimumab or Placebo for Metastatic Non–Small-Cell Lung Cancer With PD-L1 Tumor Proportion Score ≥ 50%: Randomized, Double-Blind Phase III KEYNOTE-598 Study. Journal of Clinical Oncology, 39(21), 2327–2338. https://doi.org/10.1200/JCO.20.03579 |
| KEYNOTE-604 | NCT03066778 | Rudin, C. M., Awad, M. M., Navarro, A., Gottfried, M., Peters, S., Csőszi, T., Cheema, P. K., Rodriguez-Abreu, D., Wollner, M., Yang, J. C.-H., Mazieres, J., Orlandi, F. J., Luft, A., Gümüş, M., Kato, T., Kalemkerian, G. P., Luo, Y., Ebiana, V., Pietanza, M. C., … on behalf of the KEYNOTE-604 Investigators. (2020). Pembrolizumab or Placebo Plus Etoposide and Platinum as First-Line Therapy for Extensive-Stage Small-Cell Lung Cancer: Randomized, Double-Blind, Phase III KEYNOTE-604 Study. Journal of Clinical Oncology, 38(21), 2369–2379. https://doi.org/10.1200/JCO.20.00793 |
| MDX010-20 | NCT00094653 | Hodi, F. S., O’Day, S. J., McDermott, D. F., Weber, R. W., Sosman, J. A., Haanen, J. B., Gonzalez, R., Robert, C., Schadendorf, D., Hassel, J. C., Akerley, W., van den Eertwegh, A. J. M., Lutzky, J., Lorigan, P., Vaubel, J. M., Linette, G. P., Hogg, D., Ottensmeier, C. H., Lebbé, C., … Urba, W. J. (2010). Improved Survival with Ipilimumab in Patients with Metastatic Melanoma. New England Journal of Medicine, 363(8), 711–723. https://doi.org/10.1056/NEJMoa1003466 |
| MYSTIC | NCT02453282 | Rizvi, N. A., Cho, B. C., Reinmuth, N., Lee, K. H., Luft, A., Ahn, M.-J., van den Heuvel, M. M., Cobo, M., Vicente, D., Smolin, A., Moiseyenko, V., Antonia, S. J., Le Moulec, S., Robinet, G., Natale, R., Schneider, J., Shepherd, F. A., Geater, S. L., Garon, E. B., … for the MYSTIC Investigators. (2020). Durvalumab With or Without Tremelimumab vs Standard Chemotherapy in First-line Treatment of Metastatic Non–Small Cell Lung Cancer: The MYSTIC Phase 3 Randomized Clinical Trial. JAMA Oncology, 6(5), 661. https://doi.org/10.1001/jamaoncol.2020.0237 |
| NCT00324155 | NCT00324155 | Robert, C., Thomas, L., Bondarenko, I., O’Day, S., Weber, J., Garbe, C., Lebbe, C., Baurain, J.-F., Testori, A., Grob, J.-J., Davidson, N., Richards, J., Maio, M., Hauschild, A., Miller, W. H., Gascon, P., Lotem, M., Harmankaya, K., Ibrahim, R., … Wolchok, J. D. (2011). Ipilimumab plus Dacarbazine for Previously Untreated Metastatic Melanoma. New England Journal of Medicine, 364(26), 2517–2526. https://doi.org/10.1056/NEJMoa1104621 |
| NCT01450761 | NCT01450761 | Reck, M., Luft, A., Szczesna, A., Havel, L., Kim, S.-W., Akerley, W., Pietanza, M. C., Wu, Y., Zielinski, C., Thomas, M., Felip, E., Gold, K., Horn, L., Aerts, J., Nakagawa, K., Lorigan, P., Pieters, A., Kong Sanchez, T., Fairchild, J., & Spigel, D. (2016). Phase III Randomized Trial of Ipilimumab Plus Etoposide and Platinum Versus Placebo Plus Etoposide and Platinum in Extensive-Stage Small-Cell Lung Cancer. Journal of Clinical Oncology, 34(31), 3740–3748. https://doi.org/10.1200/JCO.2016.67.6601 |
| NCT01471197 | NCT01471197 | A Randomized, Open-Label, Phase 2 Safety and Efficacy Trial of Ipilimumab Versus Pemetrexed in Subjects With Recurrent/Stage IV Non-Squamous, Non-Small Cell Lung Cancer Who Have Not Progressed After Four Cycles of a Platinum-Based First Line Chemotherapy (Clinical Trial Registration No. NCT01471197). clinicaltrials.gov. Retrieved 13 December 2021, from https://clinicaltrials.gov/ct2/show/NCT01471197 |
| NCT01843374 | NCT01843374 | Maio, M., Scherpereel, A., Calabrò, L., Aerts, J., Perez, S. C., Bearz, A., Nackaerts, K., Fennell, D. A., Kowalski, D., Tsao, A. S., Taylor, P., Grosso, F., Antonia, S. J., Nowak, A. K., Taboada, M., Puglisi, M., Stockman, P. K., & Kindler, H. L. (2017). Tremelimumab as second-line or third-line treatment in relapsed malignant mesothelioma (DETERMINE): A multicentre, international, randomised, double-blind, placebo-controlled phase 2b trial. The Lancet. Oncology, 18(9), 1261–1273. https://doi.org/10.1016/S1470-2045(17)30446-1 |
| NCT02243371 | NCT02243371 | Tsujikawa, T., Crocenzi, T., Durham, J. N., Sugar, E. A., Wu, A. A., Onners, B., Nauroth, J. M., Anders, R. A., Fertig, E. J., Laheru, D. A., Reiss, K., Vonderheide, R. H., Ko, A. H., Tempero, M. A., Fisher, G. A., Considine, M., Danilova, L., Brockstedt, D. G., Coussens, L. M., … Le, D. T. (2020). Evaluation of Cyclophosphamide/GVAX Pancreas Followed by Listeria-Mesothelin (CRS-207) with or without Nivolumab in Patients with Pancreatic Cancer. Clinical Cancer Research, 26(14), 3578–3588. https://doi.org/10.1158/1078-0432.CCR-19-3978 |
| NCT02340975 | NCT02340975 | Kelly, R. J., Lee, J., Bang, Y.-J., Almhanna, K., Blum Murphy, M., Catenacci, D. V. T., Chung, H. C., Wainberg, Z. A., Gibson, M. K., Lee, K.-W., Bendell, J. C., Denlinger, C. S., Chee, C. E., Omori, T., Leidner, R., Lenz, H.-J., Chao, Y., Rebelatto, M. C., Brohawn, P., … Ku, G. Y. (2019). Safety and Efficacy of Durvalumab and Tremelimumab Alone or in Combination in Patients with Advanced Gastric and Gastroesophageal Junction Adenocarcinoma. Clinical Cancer Research, clincanres.2443.2019. https://doi.org/10.1158/1078-0432.CCR-19-2443 |
| NCT02498600 | NCT02498600 | Zamarin, D., Burger, R. A., Sill, M. W., Powell, D. J., Lankes, H. A., Feldman, M. D., Zivanovic, O., Gunderson, C., Ko, E., Mathews, C., Sharma, S., Hagemann, A. R., Khleif, S., & Aghajanian, C. (2020). Randomized Phase II Trial of Nivolumab Versus Nivolumab and Ipilimumab for Recurrent or Persistent Ovarian Cancer: An NRG Oncology Study. Journal of Clinical Oncology, 38(16), 1814–1823. https://doi.org/10.1200/JCO.19.02059 |
| NCT02558894 | NCT02558894 | O’Reilly, E. M., Oh, D.-Y., Dhani, N., Renouf, D. J., Lee, M. A., Sun, W., Fisher, G., Hezel, A., Chang, S.-C., Vlahovic, G., Takahashi, O., Yang, Y., Fitts, D., & Philip, P. A. (2019). Durvalumab With or Without Tremelimumab for Patients With Metastatic Pancreatic Ductal Adenocarcinoma: A Phase 2 Randomized Clinical Trial. JAMA Oncology, 5(10), 1431–1438. https://doi.org/10.1001/jamaoncol.2019.1588 |
| NCT02775903 | NCT02775903 | A Randomized, Multicenter, Open-label, Phase 2 Study Evaluating the Efficacy and Safety of Azacitidine Subcutaneous in Combination With Durvalumab (MEDI4736) in Previously Untreated Subjects With Higher-Risk Myelodysplastic Syndromes (MDS) or in Elderly (>= 65 Years) Acute Myeloid Leukemia (AML) Subjects Not Eligible for Hematopoietic Stem Cell Transplantation (HSCT) (Clinical Trial Registration No. NCT02775903). clinicaltrials.gov. Retrieved 13 December 2021, from https://clinicaltrials.gov/ct2/show/NCT02775903 |
| OAK | NCT02008227 | Rittmeyer, A., Barlesi, F., Waterkamp, D., Park, K., Ciardiello, F., von Pawel, J., Gadgeel, S. M., Hida, T., Kowalski, D. M., Dols, M. C., Cortinovis, D. L., Leach, J., Polikoff, J., Barrios, C., Kabbinavar, F., Frontera, O. A., De Marinis, F., Turna, H., Lee, J.-S., … Gandara, D. R. (2017). Atezolizumab versus docetaxel in patients with previously treated non-small-cell lung cancer (OAK): A phase 3, open-label, multicentre randomised controlled trial. The Lancet, 389(10066), 255–265. https://doi.org/10.1016/S0140-6736(16)32517-X |
| PACIFIC | NCT02125461 | Antonia, S. J., Villegas, A., Daniel, D., Vicente, D., Murakami, S., Hui, R., Yokoi, T., Chiappori, A., Lee, K. H., de Wit, M., Cho, B. C., Bourhaba, M., Quantin, X., Tokito, T., Mekhail, T., Planchard, D., Kim, Y.-C., Karapetis, C. S., Hiret, S., … Özgüroğlu, M. (2017). Durvalumab after Chemoradiotherapy in Stage III Non–Small-Cell Lung Cancer. New England Journal of Medicine, 377(20), 1919–1929. https://doi.org/10.1056/NEJMoa1709937 |
| POPLAR | NCT01903993 | Fehrenbacher, L., Spira, A., Ballinger, M., Kowanetz, M., Vansteenkiste, J., Mazieres, J., Park, K., Smith, D., Artal-Cortes, A., Lewanski, C., Braiteh, F., Waterkamp, D., He, P., Zou, W., Chen, D. S., Yi, J., Sandler, A., & Rittmeyer, A. (2016). Atezolizumab versus docetaxel for patients with previously treated non-small-cell lung cancer (POPLAR): A multicentre, open-label, phase 2 randomised controlled trial. The Lancet, 387(10030), 1837–1846. https://doi.org/10.1016/S0140-6736(16)00587-0 |
| PROLUNG | NCT02574598 | Arrieta, O., Barrón, F., Ramírez-Tirado, L. A., Zatarain-Barrón, Z. L., Cardona, A. F., Díaz-García, D., Yamamoto Ramos, M., Mota-Vega, B., Carmona, A., Peralta Álvarez, M. P., Bautista, Y., Aldaco, F., Gerson, R., Rolfo, C., & Rosell, R. (2020). Efficacy and Safety of Pembrolizumab Plus Docetaxel vs Docetaxel Alone in Patients With Previously Treated Advanced Non–Small Cell Lung Cancer: The PROLUNG Phase 2 Randomized Clinical Trial. JAMA Oncology, 6(6), 856. https://doi.org/10.1001/jamaoncol.2020.0409 |

## Table B.2. Characteristics of included trials

### Table B.2.1. Clinical and methodological characteristics of included trials.

| Trial Title | Date of publication | Masking | Mean age | Age standard deviation | N Female | N Total | Site of primary tumor | Stage |
| --- | --- | --- | --- | --- | --- | --- | --- | --- |
| ABC | 2018 | Open-label | - | - | 12 | 60 | Melanoma Brain Metastasis | Stage IV |
| ARTIC-Substudy-A | 2019 | Open-label | - | - | 197 | 595 | Non-Small Cell Lung Cancer | Locally advanced; Metastatic |
| ARTIC-Substudy-B | 2019 | Open-label | - | - | 197 | 595 | Non-Small Cell Lung Cancer | Locally advanced; Metastatic |
| ATTRACTION‑2 | 2019 | Double-blind | - | - | 145 | 493 | Gastric or Gastroesophageal Junction | unresectable advanced or recurrent |
| CA184-041 | 2012 | Double-blind | - | - | 85 | 334 | Non-Small Cell Lung Cancer | Stage IIIb or IV |
| CA184-043 | 2016 | Double-blind | 67.6 | 7.56 | 0 | 799 | Prostate | Metastatic |
| CA184-095 | 2016 | Double-blind | 69 | 0 | 0 | 602 | Prostate | Metastatic |
| CA184-104 | 2016 | Double-blind | 63.7 | 8.47 | 114 | 749 | Non-Small Cell Lung Cancer | Stage IV; Recurrent |
| CA184-153 | 2019 | Double-blind | 60.3 | 7.61 | 24 | 204 | Squamous Non-Small Cell Lung Cancer | Stage IV; Recurrent |
| CA184-162 | 2015 | Open-label | - | - | 37 | 114 | Gastric or Gastroesophageal Junction | Locally advanced; Metastatic |
| CA184-243 | 2015 | Open-label | 62.7 | 10.2 | 7 | 23 | Melanoma | Stage III or IV |
| CASPIAN | 2021 | Open-label | 62.6 | 8.35 | 229 | 805 | Small Cell Lung Cancer | Stage III or IV |
| CheckMate 017 | 2016 | Open-label | 63.3 | 8.36 | 64 | 272 | Squamous Non-Small Cell Lung Cancer | Stage IIIb or IV |
| CheckMate 025 | 2016 | Open-label | 61.3 | 10.66 | 202 | 821 | Renal Cell Carcinoma | Advanced; Metastatic |
| CheckMate 026 | 2017 | Open-label | 63.1 | 9.94 | 209 | 541 | Non-Small Cell Lung Cancer | Stage IV; Recurrent |
| CheckMate 037 | 2017 | Open-label | 59.2 | 13.59 | 144 | 405 | Melanoma | Advanced |
| CheckMate 057 | 2016 | Open-label | 61.6 | 9.53 | 263 | 582 | Non-Small Cell Lung Cancer | Advanced |
| CheckMate 066 | 2016 | Double-blind | 62.7 | 12.83 | 172 | 418 | Melanoma | Stage III or IV |
| CheckMate 067 | 2017 | Double-blind | 59.6 | 13.69 | 335 | 945 | Melanoma | Stage III or IV |
| CheckMate 069 | 2016 | Double-blind | 63.7 | 10.74 | 47 | 142 | Melanoma | Stage III or IV |
| CheckMate 078 | 2019 | Open-label | 59.1 | 8.77 | 107 | 504 | Non-Small Cell Lung Cancer | Advanced; Metastatic |
| CheckMate 141 | 2017 | Open-label | 59.1 | 10.43 | 61 | 361 | Head and Neck | Stage III or IV |
| CheckMate 143 | 2018 | Open-label | - | - | 9 | 20 | Glioblastoma | Stage IV; Recurrent |
| CheckMate 143 Cohort 2 | 2020 | Open-label | - | - | 134 | 369 | Glioblastoma | Stage IV; Recurrent |
| CheckMate 214 | 2018 | Open-label | 60.9 | 9.93 | 288 | 1096 | Renal Cell Carcinoma | Metastatic; Advanced |
| CheckMate 238 | 2021 | Double-blind | 54 | 13.42 | 379 | 906 | Melanoma | Stage IIIb–c or IV |
| CheckMate 331 | 2020 | Open-label | 61.6 | 8.8 | 218 | 569 | Small Cell Lung Cancer | Relapsed |
| CheckMate 451 | 2019 | Double-blind | 63.9 | 8.5 | 302 | 834 | Small Cell Lung Cancer | Extensive |
| CheckMate 743 | 2021 | Open-label | 68.2 | 9.1 | 138 | 605 | Mesothelioma | Unresectable |
| CheckMate 9LA | 2020 | Open-label | - | 9.4 | 215 | 719 | Non-Small Cell Lung Cancer | Metastatic |
| CONDOR | 2018 | Open-label | - | - | 47 | 267 | Head and Neck | Recurrent; Metastatic |
| DANUBE | 2021 | Open-label | 66.5 | 9.6 | 253 | 1032 | Urothelial | Unresectable locally advanced; Metastatic |
| EAGLE | 2019 | Open-label | 59.4 | 9.86 | 118 | 736 | Head and Neck | Recurrent; Metastatic |
| EORTC 18071 | 2014 | Double-blind | 51.1 | 12.86 | 362 | 951 | Melanoma | Stage III |
| GeparNuevo | 2019 | Double-blind | - | - | 174 | 174 | Triple Negative Breast Cancer | Nonmetastatic invasive |
| I-SPY | 2020 | Open-label | - | - | 270 | 270 | Breast | Early-Stage (II or III) |
| IMblaze370 | 2019 | Open-label | 57.8 | 11.1 | 145 | 363 | Colorectal | Unresectable locally advanced; Metastatic |
| IMmotion150 | 2017 | Open-label | 60.3 | 10.6 | 75 | 305 | Renal Cell Carcinoma | Metastatic |
| IMMUNED | 2020 | Double-blind | - | - | 72 | 167 | Melanoma | Stage IV; Resected |
| IMpassion031 | 2021 | Double-blind | 50.2 | 12.4 | 333 | 333 | Triple Negative Breast Cancer | Primary invasive |
| IMpassion130 | 2021 | Double-blind | 54.9 | 12.2 | 899 | 902 | Triple Negative Breast Cancer | Unresectable locally advanced; Metastatic |
| IMpower110 | 2021 | Open-label | 63.4 | 8.8 | 176 | 572 | Non-Small Cell Lung Cancer | Stage IV |
| IMpower130 | 2019 | Open-label | 64 | 9.3 | 308 | 723 | Non-Small Cell Lung Cancer | Stage IV |
| IMpower132 | 2020 | Open-label | 62.6 | 9.4 | 194 | 578 | Non-Small Cell Lung Cancer | Metastatic |
| IMpower133 | 2019 | Double-blind | 63.7 | 8.9 | 142 | 403 | Small Cell Lung Cancer | Extensive |
| IMpower150 | 2020 | Open-label | 62.8 | 9.3 | 482 | 1202 | Non-Small Cell Lung Cancer | Stage IV |
| IMspire150 | 2020 | Double-blind | 53.6 | 14.1 | 215 | 514 | Melanoma | Stage IV; Unresectable IIIc |
| IMspire170 | 2020 | Open-label | 63.6 | 13 | 176 | 446 | Melanoma | Locally advanced; Unresectable; Metastatic |
| IMvigor130 | 2020 | Double-blind | - | - | 297 | 1213 | Urothelial | Metastatic |
| IMvigor211 | 2018 | Open-label | 66 | 9.4 | 213 | 931 | Urothelial | Advanced; Metastatic |
| JAVELIN Bladder 100 | 2020 | Open-label | 67.44 | 9.4 | 159 | 700 | Urothelial | Unresectable locally advanced; Metastatic |
| JAVELIN Gastric 100 | 2021 | Open-label | 60.7 | 11.36 | 168 | 499 | Gastric or Gastroesophageal Junction | Unresectable locally advanced; Metastatic |
| JAVELIN Gastric 300 | 2018 | Open-label | 59.5 | 12.31 | 104 | 371 | Gastric or Gastroesophageal Junction | Locally advanced; Metastatic |
| JAVELIN Lung 200 | 2018 | Open-label | 62.7 | 9.81 | 250 | 792 | Non-Small Cell Lung Cancer | Stage IIIb or IV |
| JAVELIN Ovarian 100 | 2019 | Open-label | 57.86 | 11.05 | 998 | 998 | Ovarian Cancer | Stage III or IV |
| JAVELIN Ovarian 200 | 2019 | Open-label | 60.3 | 10.32 | 566 | 566 | Ovarian Cancer | Refractory |
| KATE2 | 2019 | Double-blind | 53.9 | 10.3 | 200 | 202 | HER2-Positive Breast Cancer | Locally advanced; Metastatic |
| KEYNOTE-006 | 2016 | Open-label | 60.3 | 14.1 | 337 | 834 | Melanoma | Advanced |
| KEYNOTE-010 | 2017 | Open-label | 62 | 9.7 | 399 | 1034 | Non-Small Cell Lung Cancer | Advanced |
| KEYNOTE-021 | 2017 | Open-label | 61.8 | 9.5 | 147 | 267 | Non-Small Cell Lung Cancer | Stage IIIb or IV |
| KEYNOTE-024 | 2017 | Open-label | 64.2 | 9.8 | 118 | 305 | Non-Small Cell Lung Cancer | Stage IV |
| KEYNOTE-040 | 2018 | Open-label | 60.2 | 9.2 | 83 | 495 | Head and Neck | Recurrent; Metastatic |
| KEYNOTE-042 | 2019 | Open-label | 62.8 | 9.7 | 372 | 1274 | Non-Small Cell Lung Cancer | Locally advanced; Metastatic |
| KEYNOTE-045 | 2017 | Open-label | 65.5 | 9.7 | 140 | 542 | Urothelial | Metastatic; Locally advanced; Unresectable |
| KEYNOTE-048 | 2020 | Open-label | 61 | 9.7 | 147 | 882 | Head and Neck | Recurrent; Metastatic |
| KEYNOTE-054 | 2019 | Double-blind | 53.8 | 13.9 | 391 | 1019 | Melanoma | Stage III |
| KEYNOTE-061 | 2018 | Open-label | 60.2 | 11.9 | 182 | 592 | Gastric or Gastroesophageal Junction | Metastatic; Locally advanced; Unresectable |
| KEYNOTE-062 | 2020 | Double-blind | 60.5 | 12 | 209 | 763 | Gastric or Gastroesophageal Junction | Advanced |
| KEYNOTE-119 | 2020 | Open-label | 52 | 11.3 | 620 | 622 | Triple Negative Breast Cancer | Metastatic |
| KEYNOTE-144 | 2019 | Open-label | 62.1 | 9.69 | 37 | 73 | Pancreas | Metastatic |
| KEYNOTE-177 | 2020 | Open-label | - | - | 154 | 307 | Colorectal | Stage IV |
| KEYNOTE-181 | 2019 | Open-label | 62.3 | 9.5 | 84 | 628 | Esophageal or Esophagogastric Junction | Metastatic; Locally advanced; Unresectable |
| KEYNOTE-183 | 2019 | Open-label | 65.9 | 9.6 | 94 | 251 | Multiple Myeloma | Relapsed; Refractory |
| KEYNOTE-185 | 2019 | Open-label | 74.3 | 6 | 166 | 310 | Multiple Myeloma | Stage I, II or III |
| KEYNOTE-189 | 2018 | Double-blind | 63.1 | 9.3 | 253 | 616 | Non-Small Cell Lung Cancer | Metastatic |
| KEYNOTE-240 | 2020 | Double-blind | 65.2 | 10.8 | 75 | 413 | Hepatocellular Carcinoma | Stage C; Stage B |
| KEYNOTE-355 | 2020 | Double-blind | - | - | 847 | 847 | Breast (triple negative) | locally recurrent inoperable or metastatic |
| KEYNOTE-407 | 2019 | Double-blind | 64.9 | 8.7 | 104 | 559 | Squamous Non-small Cell Lung Cancer | Metastatic |
| KEYNOTE-590 | 2021 | Double-blind | 62.4 | 9.5 | 124 | 749 | Esophageal | Locally advanced; Metastatic |
| KEYNOTE-598 | 2021 | Double-blind | 64.1 | 9.1 | 174 | 568 | Non-Small Cell Lung Cancer | Metastatic |
| KEYNOTE-604 | 2020 | Double-blind | 64.7 | 8.3 | 159 | 453 | Small Cell Lung Cancer | Stage IV |
| MDX010-20 | 2011 | Double-blind | 56.2 | - | 275 | 676 | Melanoma | Unresectable; Stage III or IV |
| MYSTIC | 2019 | Open-label | 63.7 | 9.74 | 346 | 1118 | Non-Small Cell Lung Cancer | Stage IV |
| NCT00324155 | 2014 | Double-blind | 57 | 13.61 | 201 | 502 | Melanoma | Metastatic |
| NCT01450761 | 2016 | Double-blind | 61.9 | 8.78 | 311 | 954 | Small Cell Lung Cancer | Extensive |
| NCT01471197 | 2014 | Open-label | 62.3 | 7.54 | 3 | 8 | Non-Small Cell Lung Cancer | Recurrent; Metastatic |
| NCT01843374 | 2017 | Double-blind | 65.6 | 9.1 | 137 | 571 | Mesothelioma | Unresectable |
| NCT02243371 | 2019 | Open-label | 63.4 | 8.6 | 32 | 93 | Pancreas | Metastatic |
| NCT02340975 | 2020 | Open-label | 59.4 | 12.9 | 30 | 113 | Gastric or Gastroesophageal Junction | Recurrent; Metastatic |
| NCT02498600 | 2020 | Open-label | NR | NR | 100 | 100 | Ovarian Cancer | Recurrent; Persistent |
| NCT02558894 | 2018 | Open-label | 61.5 | 9.49 | 31 | 65 | Pancreas | Metastatic |
| NCT02775903 | 2020 | Open-label | 74.6 | 6.7 | 84 | 213 | Acute Myeloid Leukemia or Acute Myelodysplastic Syndromes | - |
| OAK | 2017 | Open-label | 62.8 | 9.5 | 467 | 1225 | Non-Small Cell Lung Cancer | Stage IIIb/IV; Recurrent |
| PACIFIC | 2019 | Double-blind | 62.9 | 8.99 | 213 | 713 | Non-Small Cell Lung Cancer | Stage III; Unresectable |
| POPLAR | 2017 | Open-label | 61.6 | 9.3 | 118 | 287 | Non-Small Cell Lung Cancer | Metastatic; Advanced |
| PROLUNG | 2020 | Open-label | 60.01 | 12.5 | 46 | 78 | Non-Small Cell Lung Cancer | Locally advanced |

### Table B.2.2. Characterization of drug interventions.

| Trial Title | Arm 1 | Arm 1 n | Arm 2 | Arm 2 n | Arm 3 | Arm 3 n | Arm 4 | Arm 4 n |
| --- | --- | --- | --- | --- | --- | --- | --- | --- |
| ABC | nivolumab + ipilimumab 1 mg/kg + 3 mg/kg Q3W 4 cycles then nivolumab 3 mg/kg Q2W | 35 | nivolumab 3 mg/kg Q2W | 25 | - | - | - | - |
| ARTIC-Substudy-A | durvalumab 10 mg/kg Q2W | 62 | chemotherapy | 64 | - | - | - | - |
| ARTIC-Substudy-B | durvalumab + tremelimumab 20 mg/kg + 1 mg/kg Q4W 4 cycles then durvalumab 10 mg/kg Q2W | 174 | chemotherapy | 118 | durvalumab 10 mg/kg Q2W | 117 | tremelimumab 10 mg/kg Q4W for 24 weeks then Q12W | 60 |
| ATTRACTION‑2 | nivolumab 3 mg/kg Q2W | 330 | placebo | 163 | - | - | - | - |
| CA184-041 | ipilimumab + chemotherapy 10 mg/kg Q3W 4 cycles then Q12W | 113 | ipilimumab + chemotherapy (phased) 10 mg/kg Q3W 4 cycles then Q12W | 109 | placebo + chemotherapy | 109 | - | - |
| CA184-043 | ipilimumab + radiation therapy 10 mg/kg Q3W 4 cycles then Q12W | 393 | placebo + radiation therapy | 396 | - | - | - | - |
| CA184-095 | ipilimumab 10 mg/kg Q3W 4 cycles then Q12W | 400 | placebo | 202 | - | - | - | - |
| CA184-104 | ipilimumab + chemotherapy 10 mg/kg Q3W 4 cycles then Q12W | 479 | placebo + chemotherapy | 477 | - | - | - | - |
| CA184-153 | ipilimumab + chemotherapy 10 mg/kg Q3W during induction then Q12W | 148 | placebo + chemotherapy | 147 | - | - | - | - |
| CA184-162 | chemotherapy (lead-in) + ipilimumab 10 mg/kg Q3W 4 cycles then Q12W | 57 | chemotherapy (lead-in) + chemotherapy | 45 | - | - | - | - |
| CA184-243 | ipilimumab 3 mg/kg Q3W 4 cycles | 18 | chemotherapy | 5 | - | - | - | - |
| CASPIAN | durvalumab + chemotherapy 1500 mg Q3W 4 cycles | 268 | chemotherapy | 269 | - | - | - | - |
| CheckMate 017 | nivolumab 3 mg/kg Q2W | 135 | chemotherapy | 137 | - | - | - | - |
| CheckMate 025 | nivolumab 3 mg/kg Q2W | 410 | chemotherapy | 411 | - | - | - | - |
| CheckMate 026 | nivolumab 3 mg/kg Q2W | 271 | chemotherapy | 270 | - | - | - | - |
| CheckMate 037 | nivolumab 3 mg/kg Q2W | 272 | chemotherapy | 133 | - | - | - | - |
| CheckMate 057 | chemotherapy NA | 290 | nivolumab 3 mg/kg Q2W | 292 | - | - | - | - |
| CheckMate 066 | nivolumab 3 mg/kg Q2W | 210 | chemotherapy | 208 | - | - | - | - |
| CheckMate 067 | nivolumab + placebo 3 mg/kg Q2W | 316 | nivolumab + ipilimumab 1 mg/kg + 3 mg/kg Q3W 4 cycles then nivolumab 3 mg/kg Q2W | 314 | ipilimumab + placebo 3 mg/kg Q3W 4 cycles | 315 | - | - |
| CheckMate 069 | nivolumab + ipilimumab 1 mg/kg + 3 mg/kg Q3W 4 cycles then nivolumab 3 mg/kg Q2W | 95 | placebo + ipilimumab 3 mg/kg Q3W 4 cycles | 47 | - | - | - | - |
| CheckMate 078 | nivolumab 3 mg/kg Q2W | 338 | chemotherapy | 166 | - | - | - | - |
| CheckMate 141 | nivolumab 3 mg/kg Q2W | 240 | chemotherapy | 121 | - | - | - | - |
| CheckMate 143 | nivolumab 3 mg/kg Q2W | 10 | nivolumab + ipilimumab 1 mg/kg + 3 mg/kg Q3W 4 cycles then nivolumab 3 mg/kg Q2W | 10 | - | - | - | - |
| CheckMate 143 Cohort 2 | nivolumab 3 mg/kg Q2W | 184 | bevacizumab | 185 | - | - | - | - |
| CheckMate 214 | nivolumab + ipilimumab 3 mg/kg + 1 mg/kg Q3W 4 cycles then nivolumab 3 mg/kg Q2W | 550 | sunitinib | 546 | - | - | - | - |
| CheckMate 238 | nivolumab 3 mg/kg Q2W | 453 | ipilimumab 10 mg/kg Q3W 4 cycles then Q12W | 453 | - | - | - | - |
| CheckMate 331 | nivolumab 240mg Q2W | 284 | chemotherapy | 285 | - | - | - | - |
| CheckMate 451 | placebo NA | 275 | nivolumab 240 mg Q2W | 280 | nivolumab + ipilimumab 1 mg/kg + 3 mg/kg Q3W 4 cycles then nivolumab 240 mg Q2W | 279 | - | - |
| CheckMate 743 | nivolumab + ipilimumab 3 mg/kg Q2W + 1 mg/kg Q6W | 303 | chemotherapy | 302 | - | - | - | - |
| CheckMate 9LA | nivolumab + ipilimumab + chemotherapy 360 mg Q3W + 1 mg/kg Q6W | 358 | chemotherapy | 349 | - | - | - | - |
| CONDOR | durvalumab 10 mg/kg Q2W 12 months | 67 | tremelimumab 10 mg/kg Q4W 7 cycles then Q12W | 67 | durvalumab + tremelimumab 20 mg/kg + 1 mg/kg Q4W 4 cycles then durvalumab 10 mg/kg Q2W | 133 | - | - |
| DANUBE | durvalumab 1.5 g Q4W | 346 | durvalumab + tremelimumab 1.5 g + 75 mg Q4W 4 cycles then durvalumab 1.5 g Q4W | 342 | chemotherapy | 344 | - | - |
| EAGLE | durvalumab + tremelimumab 20 mg/kg + 1 mg/kg Q4W 4 cycles then durvalumab 10 mg/kg Q2W | 247 | durvalumab 10 mg/kg Q2W | 240 | chemotherapy | 249 | - | - |
| EORTC 18071 | ipilimumab 10 mg/kg Q3W 4 cycles then Q12W | 475 | placebo | 476 | - | - | - | - |
| GeparNuevo | durvalumab + chemotherapy 1500 mg Q4W | 88 | placebo + chemotherapy | 86 | - | - | - | - |
| I-SPY | pembrolizumab + chemotherapy 200 mg Q3W | 69 | chemotherapy | 201 | - | - | - | - |
| IMblaze370 | atezolizumab 1200 mg Q3W | 183 | atezolizumab + cobimetinib 1200 mg Q3W | 90 | regorafenib | 90 | - | - |
| IMmotion150 | atezolizumab + bevacizumab 1200 mg Q3W | 101 | atezolizumab 1200 mg Q3W | 103 | sunitinib | 101 | - | - |
| IMMUNED | nivolumab + ipilimumab 1 mg/kg + 3 mg/kg Q3W 4 cycles then nivolumab 3 mg/kg Q2W | 56 | nivolumab + placebo 3 mg/kg Q2W | 59 | placebo | 52 | - | - |
| IMpassion031 | atezolizumab + chemotherapy 840 mg Q2W then 1200 mg Q3W 11 cycles | 165 | placebo + chemotherapy | 168 | - | - | - | - |
| IMpassion130 | atezolizumab + chemotherapy 840 mg Q2W | 451 | placebo + chemotherapy | 451 | - | - | - | - |
| IMpower110 | chemotherapy NA | 287 | atezolizumab 1200 mg Q3W | 285 | - | - | - | - |
| IMpower130 | atezolizumab + chemotherapy 1200 mg Q3W | 483 | chemotherapy | 240 | - | - | - | - |
| IMpower132 | chemotherapy NA | 286 | atezolizumab + chemotherapy 1200 mg Q3W | 292 | - | - | - | - |
| IMpower133 | atezolizumab + chemotherapy 1200 mg Q3W | 201 | placebo + chemotherapy | 202 | - | - | - | - |
| IMpower150 | atezolizumab + chemotherapy 1200 mg Q3W | 400 | chemotherapy | 400 | - | - | - | - |
| IMspire150 | atezolizumab + vemurafenib + cobimetinib 840 mg Q2W | 256 | placebo + vemurafenib + cobimetinib | 255 | - | - | - | - |
| IMspire170 | pembrolizumab 200 mg Q3W | 224 | atezolizumab + cobimetinib 840 mg Q2W | 222 | - | - | - | - |
| IMvigor130 | atezolizumab + chemotherapy 1200 mg Q3W | 451 | atezolizumab 1200 mg Q3W | 362 | placebo + chemotherapy | 400 | - | - |
| IMvigor211 | atezolizumab 1200 mg Q3W | 467 | chemotherapy | 464 | - | - | - | - |
| JAVELIN Bladder 100 | avelumab 10 mg/kg Q2W | 350 | no intervention | 350 | - | - | - | - |
| JAVELIN Gastric 100 | avelumab 10 mg/kg Q2W | 249 | chemotherapy | 250 | - | - | - | - |
| JAVELIN Gastric 300 | avelumab 10 mg/kg Q2W | 186 | chemotherapy | 185 | - | - | - | - |
| JAVELIN Lung 200 | avelumab 10 mg/kg Q2W | 396 | chemotherapy | 265 | - | - | - | - |
| JAVELIN Ovarian 100 | avelumab + chemotherapy (phased) 10 mg/kg Q2W 24 months | 332 | avelumab + chemotherapy 10 mg/kg Q3W 24 months | 331 | chemotherapy | 335 | - | - |
| JAVELIN Ovarian 200 | avelumab 10 mg/kg Q2W | 188 | avelumab + chemotherapy 10 mg/kg Q2W | 188 | chemotherapy | 190 | - | - |
| KATE2 | atezolizumab + trastuzumab emtansine 1200 mg Q3W | 133 | placebo + trastuzumab emtansine | 69 | - | - | - | - |
| KEYNOTE-006 | ipilimumab 3 mg/kg Q3W 4 cycles | 278 | pembrolizumab 10 mg/kg Q2W 24 months | 279 | pembrolizumab 10 mg/kg Q3W 24 months | 277 | - | - |
| KEYNOTE-010 | pembrolizumab 2 mg/kg Q3W 24 months | 345 | pembrolizumab 10 mg/kg Q3W 24 months | 343 | chemotherapy | 343 | - | - |
| KEYNOTE-021 | pembrolizumab + chemotherapy 200 mg Q3W | 60 | chemotherapy | 63 | - | - | - | - |
| KEYNOTE-024 | pembrolizumab 200 mg Q3W | 154 | chemotherapy | 151 | - | - | - | - |
| KEYNOTE-040 | pembrolizumab 200 mg Q3W 35 cycles | 247 | chemotherapy | 248 | - | - | - | - |
| KEYNOTE-042 | pembrolizumab 200 mg Q3W | 637 | chemotherapy | 637 | - | - | - | - |
| KEYNOTE-045 | pembrolizumab 200 mg Q3W 35 cycles | 270 | chemotherapy | 272 | - | - | - | - |
| KEYNOTE-048 | pembrolizumab 200 mg Q3W | 301 | pembrolizumab + chemotherapy 200 mg Q3W | 281 | chemotherapy + cetuximab | 300 | - | - |
| KEYNOTE-054 | pembrolizumab 200 mg Q3W | 514 | placebo | 505 | - | - | - | - |
| KEYNOTE-061 | pembrolizumab 200 mg Q3W 35 cycles | 296 | chemotherapy | 296 | - | - | - | - |
| KEYNOTE-062 | pembrolizumab 200 mg Q3W | 256 | pembrolizumab + chemotherapy 200 mg Q3W | 257 | placebo + chemotherapy | 250 | - | - |
| KEYNOTE-119 | pembrolizumab 200 mg Q3W | 212 | chemotherapy | 310 | - | - | - | - |
| KEYNOTE-144 | acalabrutinib NA | 37 | pembrolizumab + acalabrutinib 200 mg Q3W | 40 | - | - | - | - |
| KEYNOTE-177 | pembrolizumab 200 mg Q3W | 153 | chemotherapy | 154 | - | - | - | - |
| KEYNOTE-181 | pembrolizumab 200 mg Q3W | 314 | chemotherapy | 314 | - | - | - | - |
| KEYNOTE-183 | pembrolizumab + pomalidomide + dexametasone 200 mg Q3W | 122 | placebo + pomalidomide + dexametasone | 123 | - | - | - | - |
| KEYNOTE-185 | pembrolizumab + lenalidomide + dexamethasone 200 mg Q3W 18 cycles | 156 | lenalidomide + dexamethasone | 154 | - | - | - | - |
| KEYNOTE-189 | pembrolizumab + chemotherapy 200 mg Q3W | 405 | placebo + chemotherapy | 202 | - | - | - | - |
| KEYNOTE-240 | pembrolizumab 200 mg Q3W | 278 | placebo | 135 | - | - | - | - |
| KEYNOTE-355 | pembrolizumab + chemotherapy 200 mg Q3W | 566 | placebo + chemotherapy | 281 | - | - | - | - |
| KEYNOTE-407 | pembrolizumab + chemotherapy 200 mg Q3W 35 cycles | 278 | placebo + chemotherapy | 280 | - | - | - | - |
| KEYNOTE-590 | pembrolizumab + chemotherapy 200 mg Q3W | 373 | placebo + chemotherapy | 376 | - | - | - | - |
| KEYNOTE-598 | pembrolizumab + ipilimumab 200 mg Q3W + 1 mg/kg Q6W | 284 | pembrolizumab + placebo 200 mg Q3W | 284 | - | - | - | - |
| KEYNOTE-604 | pembrolizumab + chemotherapy 200 mg Q3W | 228 | placebo + chemotherapy | 225 | - | - | - | - |
| MDX010-20 | ipilimumab + gp100 Melanoma Peptide Vaccine 3 mg/kg Q3W | 403 | gp100 Melanoma Peptide Vaccine | 136 | - | - | - | - |
| MYSTIC | durvalumab 20 mg/kg Q4W | 374 | durvalumab + tremelimumab 20 mg/kg + 1 mg/kg Q4W 4 cycles then durvalumab 20 mg/kg Q4W | 372 | chemotherapy | 372 | - | - |
| NCT00324155 | ipilimumab + chemotherapy 10 mg/kg Q3W 4 cycles then Q12W | 250 | placebo + chemotherapy | 252 | - | - | - | - |
| NCT01450761 | ipilimumab + chemotherapy 10 mg/kg Q3W 4 cycles then Q12W | 478 | placebo + chemotherapy | 476 | - | - | - | - |
| NCT01471197 | ipilimumab 10 mg/kg Q3W 4 cycles then Q12W | 6 | chemotherapy | 2 | - | - | - | - |
| NCT01843374 | placebo NA | 189 | tremelimumab 10 mg/kg | 382 | - | - | - | - |
| NCT02243371 | nivolumab + chemotherapy + GVAX Pancreas Vaccine + CRS-207 3 mg/kg Q3W | 51 | chemotherapy + GVAX Pancreas Vaccine + CRS-207 | 42 | - | - | - | - |
| NCT02340975 | durvalumab + tremelimumab 20 mg/kg + 1 mg/kg Q4W 4 cycles then durvalumab 10 mg/kg Q2W | 27 | durvalumab 10 mg/kg Q2W | 24 | tremelimumab 10mg/kg Q4W | 12 | - | - |
| NCT02498600 | nivolumab 3 mg/kg Q2W | 49 | nivolumab + ipilimumab 3 mg/kg + 1 mg/kg Q3W 4 cycles then nivolumab 3 mg/kg Q2W | 51 | - | - | - | - |
| NCT02558894 | durvalumab + tremelimumab 1.5 g + 1 mg/kg Q4W 4 cycles then durvalumab 1.5 g Q4W | 33 | durvalumab 1.5 g Q4W | 32 | - | - | - | - |
| NCT02775903 | durvalumab + chemotherapy 1500 mg Q3W | 42 | chemotherapy | 42 | durvalumab + chemotherapy 1500 mg Q3W | 64 | chemotherapy | 65 |
| OAK | atezolizumab 1200 mg Q3W | 612 | chemotherapy | 613 | - | - | - | - |
| PACIFIC | durvalumab 10 mg/kg Q2W 12 months | 476 | placebo | 237 | - | - | - | - |
| POPLAR | chemotherapy NA | 144 | atezolizumab 1200 mg Q3W | 143 | - | - | - | - |
| PROLUNG | pembrolizumab + chemotherapy 200 mg Q3W | 40 | chemotherapy | 38 | - | - | - | - |

### Table B.2.3. Patients per cancer type, trial characteristics.

| Type of cancer | Number of trials | Number pf Participants |
| --- | --- | --- |
| Acute myeloid leucemia/Acute Myelodysplastic Syndromes | 1 | 213 |
| Breast | 7 | 3,350 |
| Colorectal | 2 | 670 |
| Esophageal/Gastric/GE junction | 9 | 4,322 |
| Glioblastoma | 2 | 389 |
| Head and neck | 5 | 2,741 |
| Hepatocellular | 1 | 413 |
| Melanoma | 15 | 8,008 |
| Mesothelioma | 2 | 1,176 |
| Multiple Myeloma | 2 | 561 |
| Non-Small Cell Lung Cancer | 28 | 17,014 |
| Ovarian cancer | 3 | 1664 |
| Pancreas | 3 | 231 |
| Prostate | 2 | 1,401 |
| Renal cell carcinoma | 3 | 2,222 |
| Small Cell Lung Cancer | 6 | 4,018 |
| Urothelial | 5 | 4,418 |

## Table B.3. Risk of bias assessment

| Trial | Random sequence generation | Allocation concealment | Performance bias | Detection bias | Attrition bias | Selective reporting bias | Overall risk of bias |
| --- | --- | --- | --- | --- | --- | --- | --- |
| ABC | Low | Low | High | High | Unclear | High | High |
| ARTIC-Substudy-A | Low | Low | High | Low | High | High | High |
| ARTIC-Substudy-B | Low | Low | High | Low | High | Low | High |
| ATTRACTION‑2 | Low | Low | Low | Low | Unclear | Low | Low |
| CA184-041 | Low | Unclear | Low | Low | Low | Low | High |
| CA184-043 | Low | Low | Low | Low | High | Low | Low |
| CA184-095 | Low | Unclear | High | Unclear | Low | Low | High |
| CA184-104 | Low | Unclear | Low | Low | Low | Low | High |
| CA184-153 | Unclear | Unclear | Low | Unclear | Unclear | Low | High |
| CA184-162 | Low | Unclear | High | Unclear | Low | Low | High |
| CA184-243 | Unclear | Unclear | High | High | Unclear | High | Low |
| CASPIAN | Low | Low | High | Unclear | Unclear | Low | High |
| Checkmate 017 | Low | Low | High | Unclear | Unclear | Low | High |
| Checkmate 025 | Low | Unclear | High | Unclear | Low | Low | High |
| Checkmate 026 | Low | Unclear | High | Unclear | Low | Low | High |
| Checkmate 037 | Low | Low | High | Low | Low | High | Low |
| Checkmate 057 | Low | Low | High | Unclear | Low | Low | Low |
| Checkmate 066 | Low | Unclear | Low | Low | Low | Low | High |
| Checkmate 067 | Low | Low | Low | Low | Low | Low | High |
| Checkmate 069 | Low | Low | Low | Low | Low | Low | High |
| Checkmate 078 | Low | Low | High | Unclear | Unclear | Low | High |
| Checkmate 141 | Low | Unclear | High | Unclear | High | Low | High |
| Checkmate 143 | Low | Unclear | High | Low | Low | High | Low |
| Checkmate 143 Cohort 2 | Low | Low | High | High | Unclear | High | High |
| Checkmate 214 | Low | Unclear | High | Low | High | High | High |
| Checkmate 238 | Low | Low | Low | Low | Low | High | High |
| Checkmate 331 | Low | Low | High | Low | High | Low | High |
| Checkmate 451 | Low | Low | Low | Low | Unclear | Low | High |
| Checkmate 743 | Low | Low | High | Unclear | High | Low | High |
| Checkmate 9LA | Low | Low | High | Low | High | High | High |
| CONDOR | Low | Low | High | Unclear | Low | Low | High |
| DANUBE | Low | Low | High | Low | Low | High | High |
| EAGLE | Low | Low | High | Unclear | Low | Low | High |
| EORTC 18071 | Low | Low | Low | Low | High | Low | High |
| Geparnuevo | Low | Unclear | Low | Low | High | High | Low |
| I-SPY | Low | High | High | Unclear | Unclear | High | High |
| Imblaze370 | Low | Low | High | Low | Low | High | High |
| Imblaze370 | Low | Low | High | Low | Low | High | High |
| Immotion150 | Low | Unclear | High | Unclear | Low | High | High |
| IMMUNED | Low | Low | Low | Low | Unclear | High | High |
| Impassion031 | Low | Low | Low | Low | Low | Low | Low |
| Impassion130 | Low | Low | Low | Low | Low | Low | Low |
| Impower110 | Low | Low | High | Unclear | Unclear | Low | High |
| Impower130 | Low | Low | High | Unclear | Low | Low | High |
| Impower132 | Low | Low | High | Unclear | Low | Low | High |
| Impower133 | Low | Unclear | Low | Low | Unclear | Low | High |
| Impower150 | Low | Unclear | High | Unclear | Unclear | Low | High |
| Imspire150 | Low | Low | Low | Low | Low | High | High |
| Imspire170 | Low | Low | High | Unclear | High | High | Low |
| Imvigor130 | Low | Low | Low | Low | High | High | High |
| Imvigor211 | Low | High | High | Unclear | High | Low | High |
| JAVELIN Bladder 100 | Low | Unclear | High | Low | Unclear | High | High |
| JAVELIN Gastric 100 | Low | High | High | Unclear | Low | Low | High |
| JAVELIN Lung 200 | Low | Unclear | High | Unclear | Low | Low | High |
| JAVELIN Ovarian 100 | Low | Low | High | Unclear | Unclear | Low | High |
| KATE2 | Low | Low | High | Unclear | High | Low | Low |
| KEYNOTE-006 | Low | Low | High | Unclear | High | Low | High |
| KEYNOTE-010 | Low | Low | High | Unclear | Low | Low | High |
| KEYNOTE-021 | Low | Low | High | Low | Low | High | High |
| KEYNOTE-024 | Low | Unclear | High | Low | Unclear | Low | High |
| KEYNOTE-040 | Low | Low | High | Unclear | Low | Low | High |
| KEYNOTE-042 | Low | Low | High | Unclear | Low | Low | High |
| KEYNOTE-045 | Low | Unclear | High | Unclear | Low | Low | High |
| KEYNOTE-048 | Low | Low | High | Unclear | Low | High | High |
| KEYNOTE-054 | Low | Unclear | Low | Low | Low | Low | High |
| KEYNOTE-061 | Low | Low | High | Unclear | Low | Low | High |
| KEYNOTE-062 | Low | Low | High | Low | Low | Low | High |
| KEYNOTE-119 | Unclear | Unclear | High | High | Unclear | Unclear | High |
| KEYNOTE-144 | Low | Low | High | High | Low | Unclear | High |
| KEYNOTE-177 | Low | High | High | Low | Low | High | High |
| KEYNOTE-181 | Low | Low | High | Unclear | Unclear | Low | High |
| KEYNOTE-183 | Low | Low | High | Low | Low | Unclear | High |
| KEYNOTE-185 | Low | High | High | Low | Low | High | High |
| KEYNOTE-189 | Low | Low | Low | Low | High | Low | Low |
| KEYNOTE-240 | Low | Low | High | Low | Unclear | Low | High |
| KEYNOTE-355 | Low | Low | Low | Low | Unclear | Low | Low |
| KEYNOTE-407 | Low | Low | Low | Low | High | Low | Low |
| KEYNOTE-590 | Low | Low | Low | Unclear | Low | Low | Low |
| KEYNOTE-598 | Low | Low | Low | Low | Low | High | High |
| KEYNOTE-604 | Low | Low | Low | Low | Unclear | High | High |
| MDX010-20 | Unclear | Unclear | Low | Low | High | Low | High |
| MYSTIC | Low | Low | High | Low | Low | Low | High |
| NCT00324155 | Low | Low | Low | Low | Low | Low | Low |
| NCT01450761 | Low | Low | Low | Low | Low | Low | Low |
| NCT01471197 | Low | High | High | Low | Low | Low | High |
| NCT01843374 | Low | Low | Low | Low | High | Low | High |
| NCT02243371 | Low | Unclear | High | High | Unclear | Unclear | High |
| NCT02340975 | Unclear | Unclear | High | Unclear | Low | Low | High |
| NCT02498600 | Low | Low | High | Unclear | High | Low | High |
| NCT02558894 | Low | Low | High | High | Unclear | Unclear | High |
| NCT02775903 | Unclear | Low | High | High | Unclear | Unclear | High |
| OAK | Low | High | High | Unclear | Low | Low | High |
| PACIFIC | Low | Low | Low | Unclear | Low | High | High |
| PACIFIC | Low | Low | Low | Unclear | Low | High | High |
| POPLAR | Low | Low | High | Unclear | Low | Low | High |
| PROLUNG | Low | Unclear | High | Unclear | Unclear | Low | High |

##

## Figure B.2. Network plots based on individual treatments

### Figure B.2.1. Network plot for individual treatments, pneumonitis


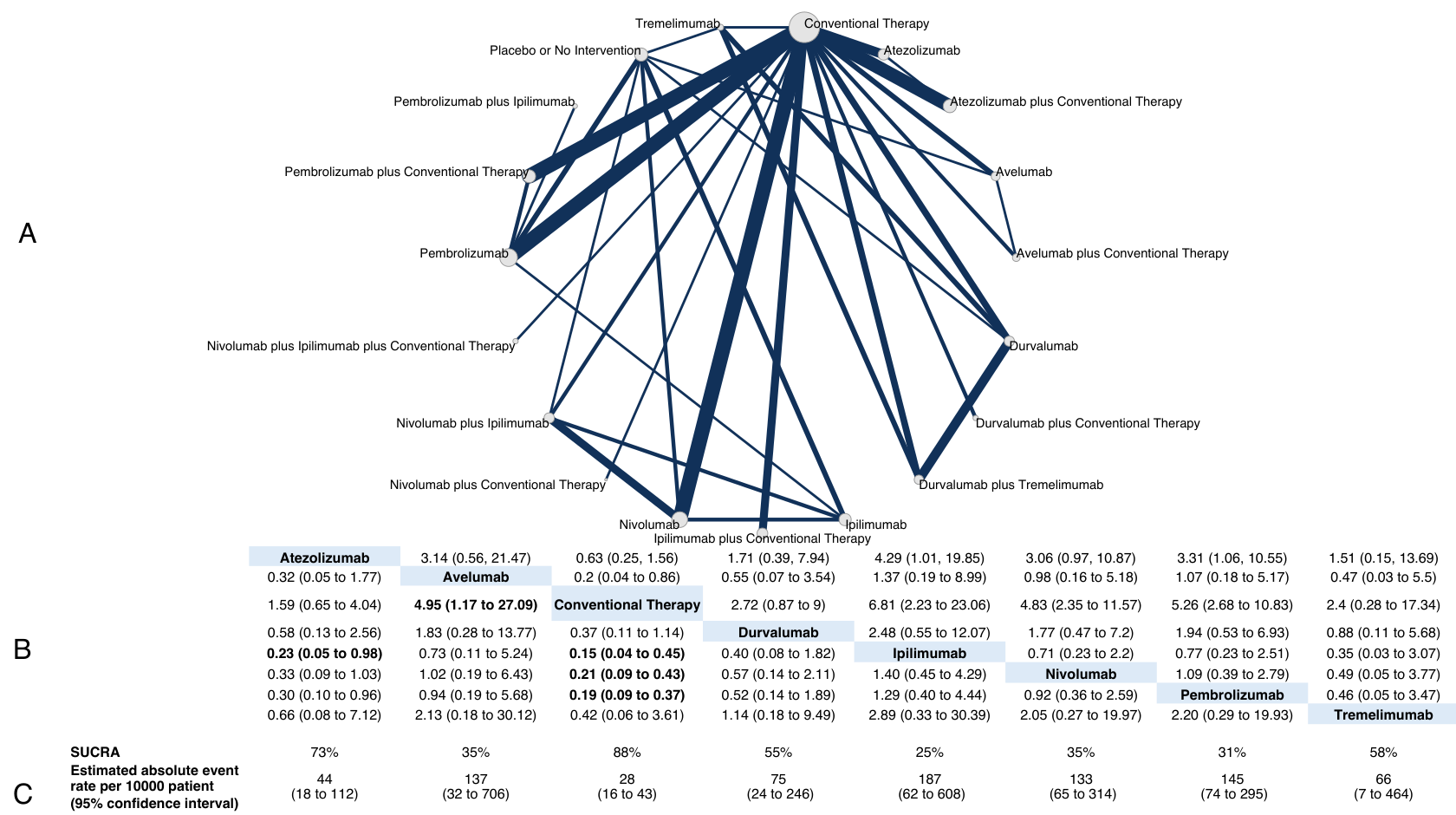


(A) Network plot showing comparisons in serious pneumonitis between nodes (grey circles), each representing an intervention. The size of each node is proportional to the total number of participants assigned to the intervention. The width of each connecting line is proportional to the number of studies performing head-to-head comparisons between the two nodes. (B) League table showing the comparative safety profile of each intervention in terms of this outcome. Values in each cell refer to odds ratios and corresponding 95% credible intervals. The interventions are ordered alphabetically. Significant results are in bold. (C) Estimated absolute event rates for each intervention, expressed as rate per 10,000 patients, with corresponding 95% confidence intervals and SUCRA, expressed as a percentage, with higher values indicating a higher certainty that an intervention is superior in terms of the risk of this outcome.

### Figure B.2.2. Network plot for individual treatments, colitis


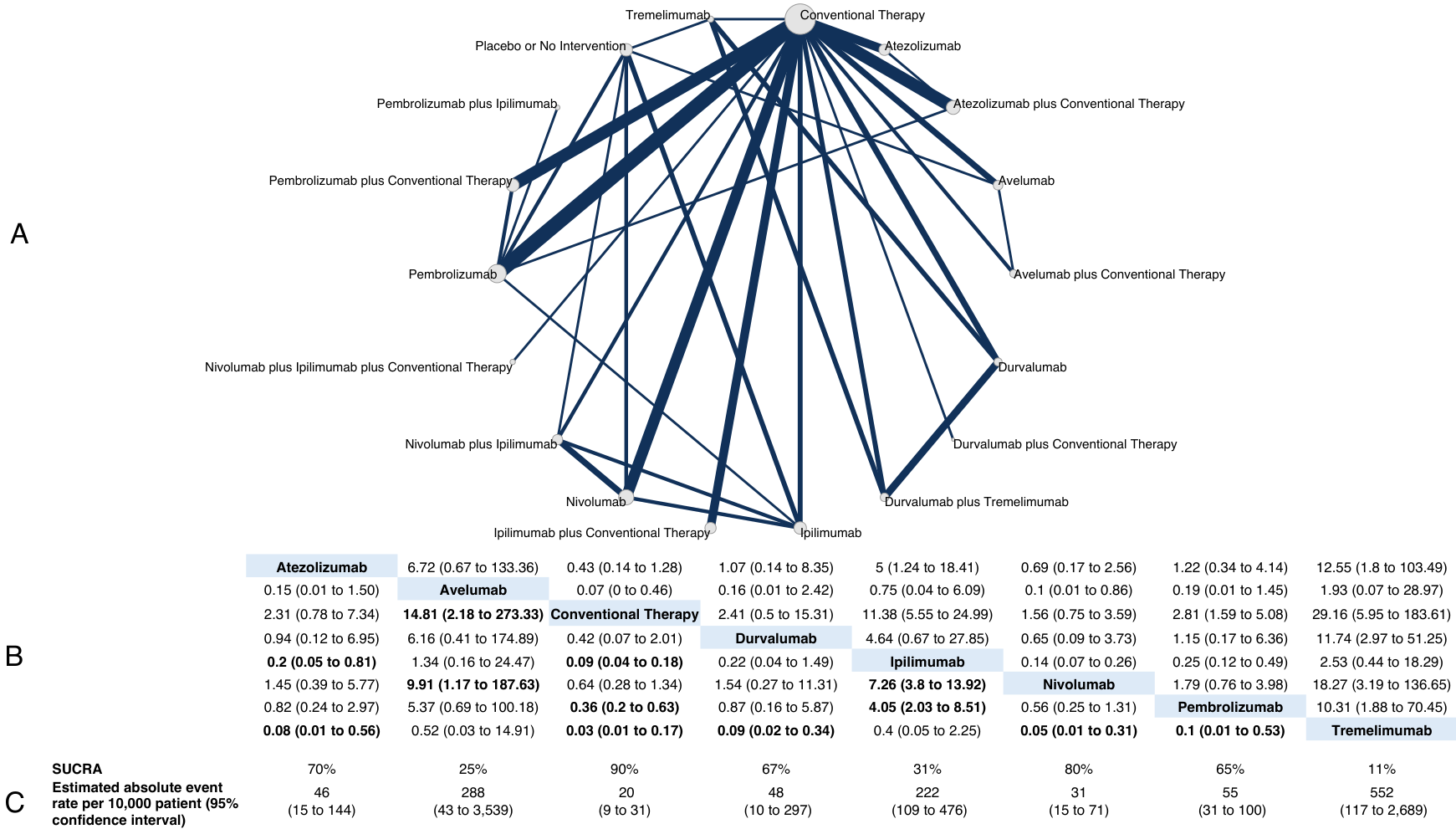


(A) Network plot showing comparisons in serious colitis between nodes (grey circles), each representing an intervention. The size of each node is proportional to the total number of participants assigned to the intervention. The width of each connecting line is proportional to the number of studies performing head-to-head comparisons between the two nodes. (B) League table showing the comparative safety profile of each intervention in terms of this outcome. Values in each cell refer to odds ratios and corresponding 95% credible intervals. The interventions are ordered alphabetically. Significant results are in bold. (C) Estimated absolute event rates for each intervention, expressed as rate per 10,000 patients, with corresponding 95% confidence intervals and SUCRA, expressed as a percentage, with higher values indicating a higher certainty that an intervention is superior in terms of the risk of this outcome.

### Figure B.2.3. Network plot for individual treatments, hepatitis


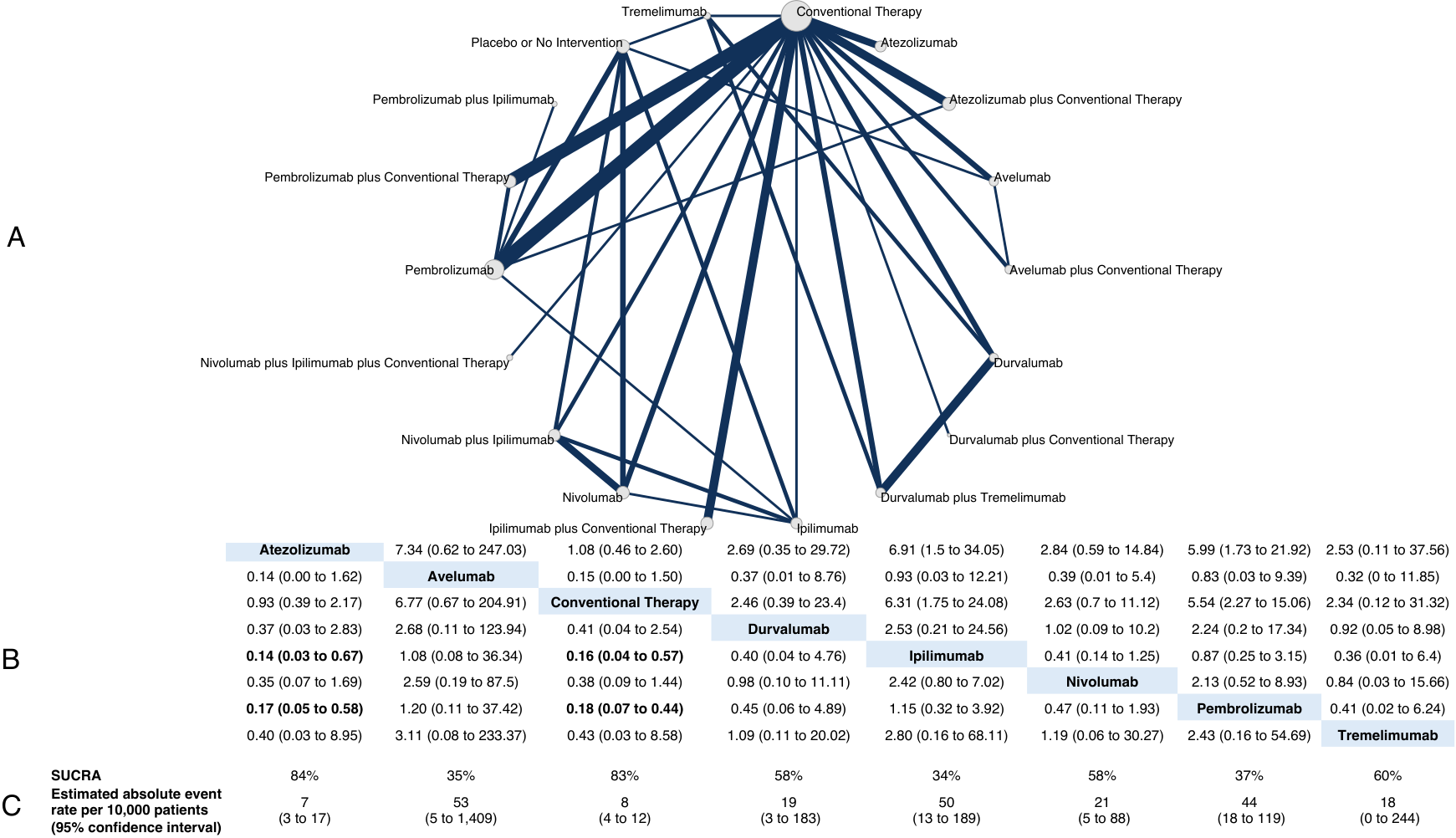


(A) Network plot showing comparisons in serious hepatitis between nodes (grey circles), each representing an intervention. The size of each node is proportional to the total number of participants assigned to the intervention. The width of each connecting line is proportional to the number of studies performing head-to-head comparisons between the two nodes. (B) League table showing the comparative safety profile of each intervention in terms of this outcome. Values in each cell refer to odds ratios and corresponding 95% credible intervals. The interventions are ordered alphabetically. Significant results are in bold. (C) Estimated absolute event rates for each intervention, expressed as rate per 10,000 patients, with corresponding 95% confidence intervals and SUCRA, expressed as a percentage, with higher values indicating a higher certainty that an intervention is superior in terms of the risk of this outcome.

### Figure B.2.4. Network plot for individual treatments, hypophysitis


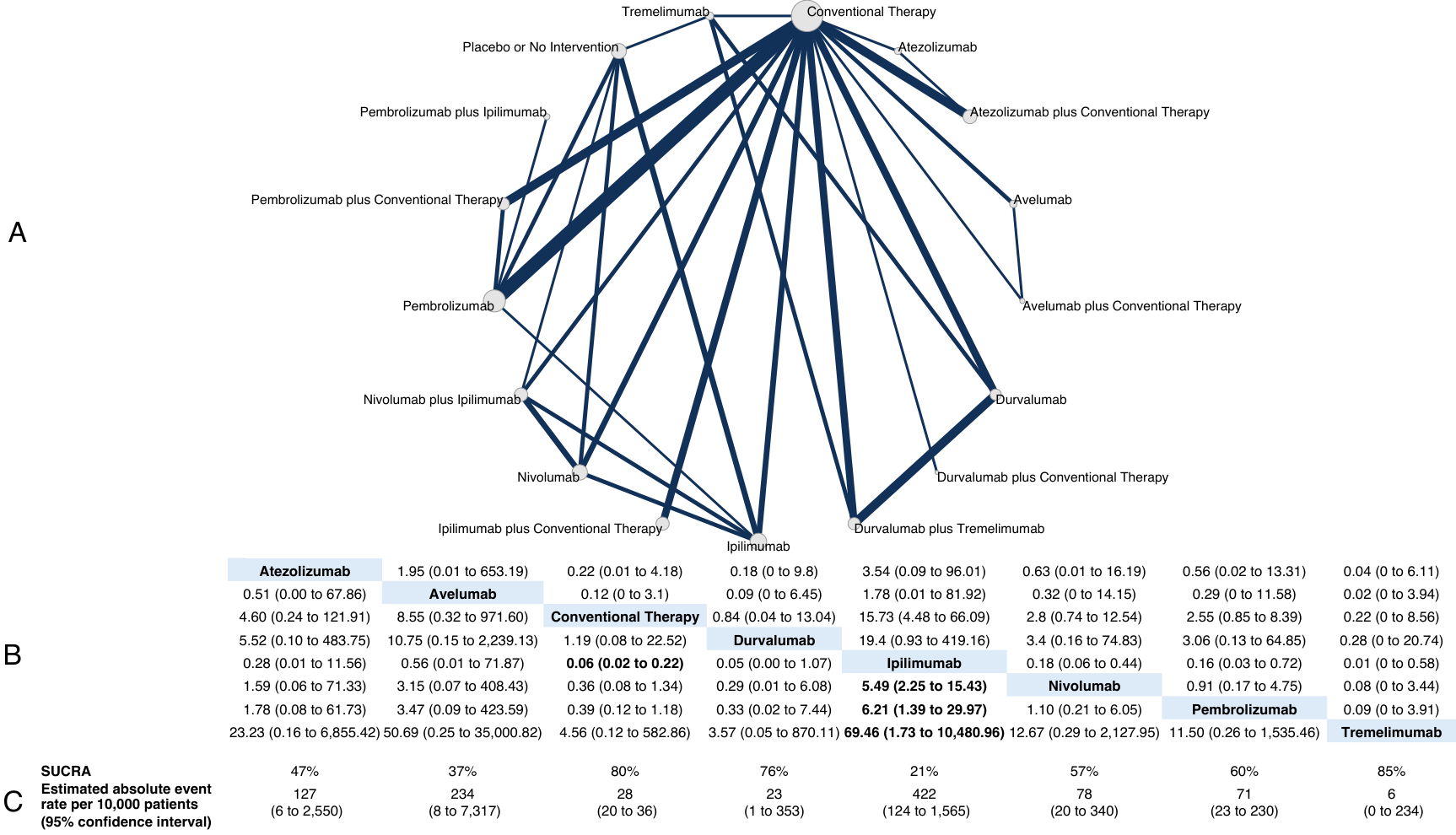


(A) Network plot showing comparisons in serious hypophysitis between nodes (grey circles), each representing an intervention. The size of each node is proportional to the total number of participants assigned to the intervention. The width of each connecting line is proportional to the number of studies performing head-to-head comparisons between the two nodes. (B) League table showing the comparative safety profile of each intervention in terms of this outcome. Values in each cell refer to odds ratios and corresponding 95% credible intervals. The interventions are ordered alphabetically. Significant results are in bold. (C) Estimated absolute event rates for each intervention, expressed as rate per 10,000 patients, with corresponding 95% confidence intervals and SUCRA, expressed as a percentage, with higher values indicating a higher certainty that an intervention is superior in terms of the risk of this outcome.

## Figure B.3. Network plots based on treatment modalities

### Figure B.3.1. Network plot for treatment modalities, overall immune-related serious adverse events.

### Figure B.3.2. Network plot for treatment modalities, pneumonitis.


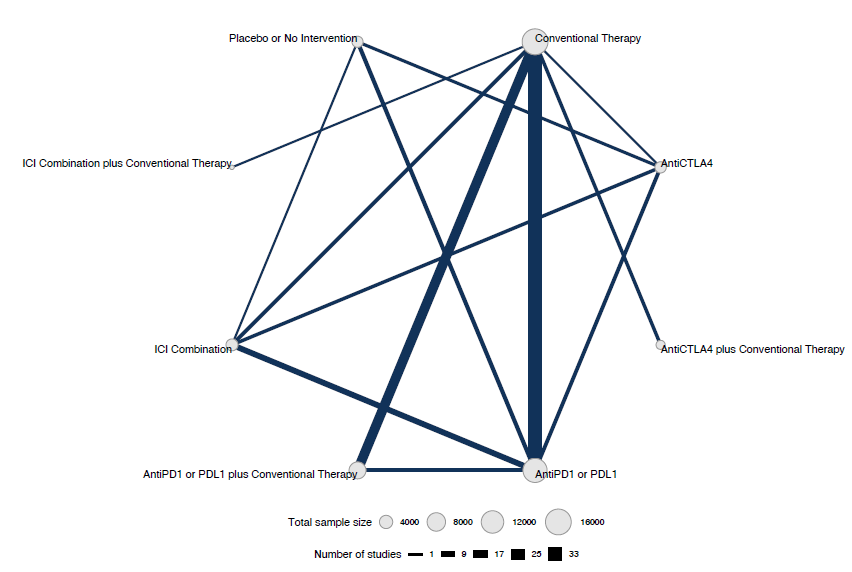


### Figure B.3.3. Network plot, treatment modalities, myocarditis.


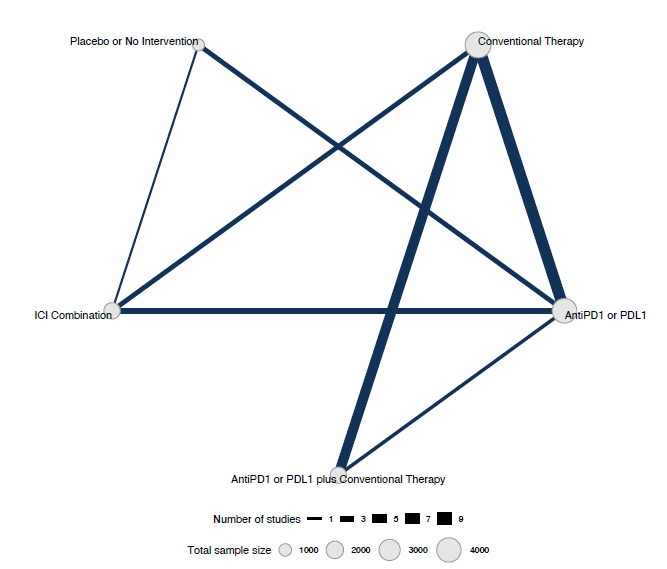


### Figure B.3.4. Network plot, treatment modalities, colitis.


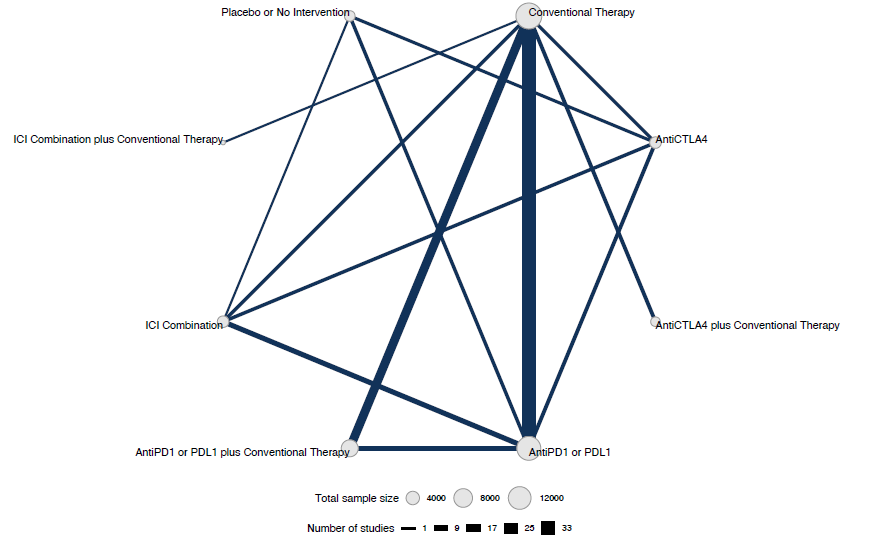


### Figure B.3.5. Network plot, treatment modalities, nephritis


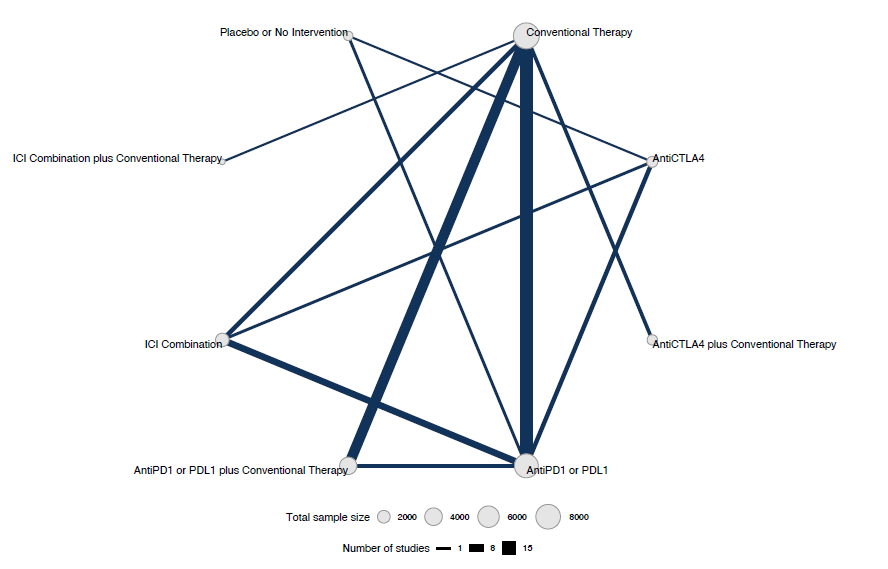


### Figure B.3.6. Network plot, treatment modalities, pancreatitis


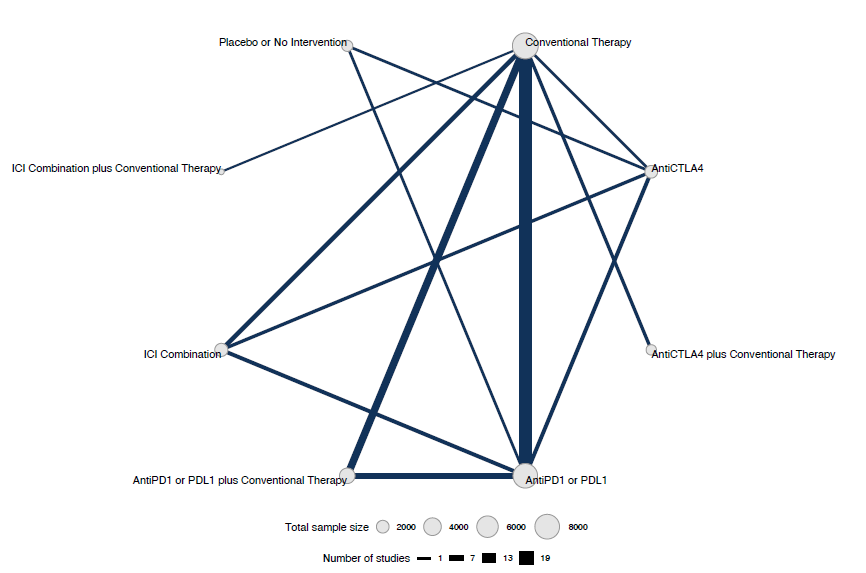


### Figure B.3.7. Network plot, treatment modalities, hepatitis.


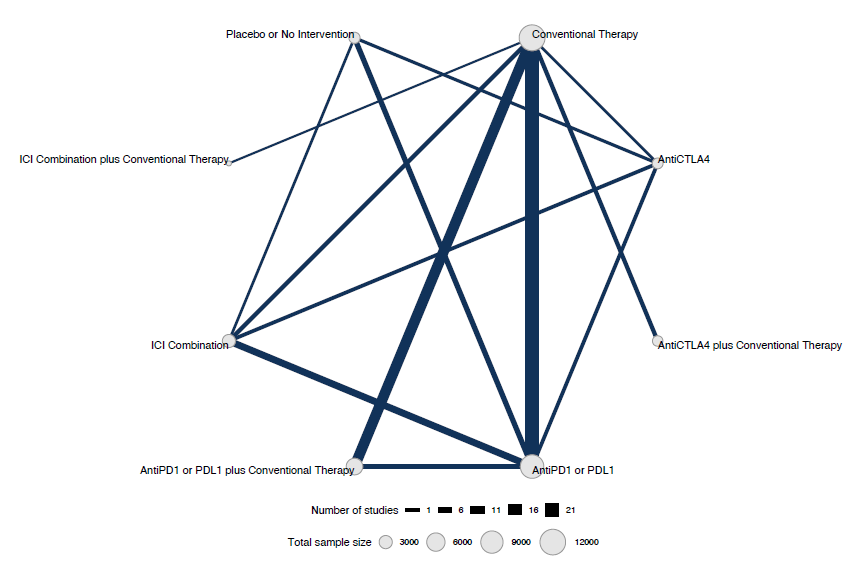


### Figure B.3.8. Network plot, treatment modalities, hypophysitis.


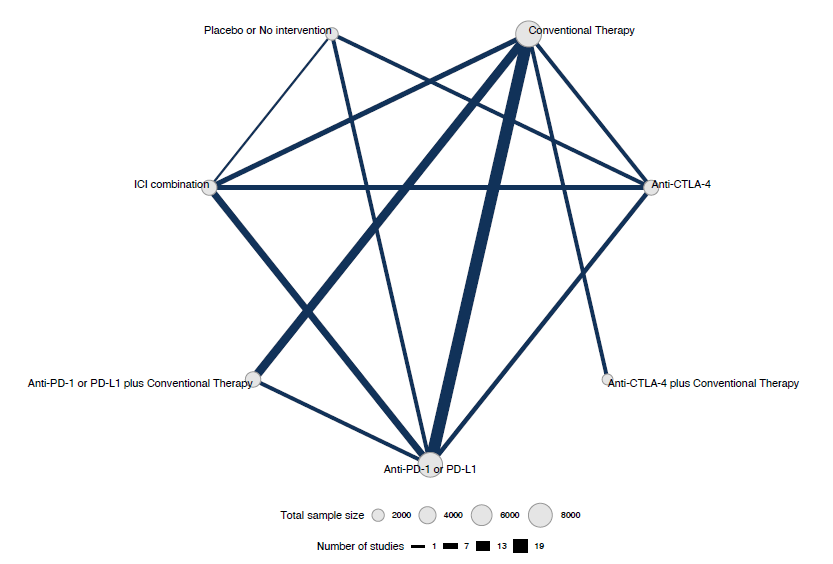


## Table B.4. Model fit for each outcome

### Table B.4.1. Network meta-analysis models, individual interventions, overall immune-related serious adverse events.

|  | Fixed effects model | Random effects model | Unrelated mean effect model |
| --- | --- | --- | --- |
| Data points | 206 | 206 | 206 |
| Posterior mean residual deviance | 330.01 | 218,86 | 213,2 |
| pD | 116.3 | 143,42 | 149,34 |
| DIC | 446.31 | 362,28 | 362,54 |
| Tau | - | 0,81 | 0,89 |
| SD | - | 0,8 | 0,88 |
| SD 95% CrI lower bound | - | 0,57 | 0,61 |
| SD 95% CrI upper bound | - | 1,09 | 1,22 |

CrI, credible interval. DIC, deviance information criterion. SD, standard deviation.

### Table B.4.2. Network meta-analysis models, individual interventions, pneumonitis.

|  | Fixed effects model | Random effects model | Unrelated mean effect model |
| --- | --- | --- | --- |
| Data points | 177 | 177 | 177 |
| Posterior mean residual deviance | 239.7 | 192.09 | 181.71 |
| pD | 103.31 | 109.64 | 116.69 |
| DIC | 343.01 | 301.73 | 298.41 |
| Tau | - | 0.68 | 0.86 |
| SD | - | 0.69 | 0.85 |
| SD 95% CrI lower bound | - | 0.26 | 0.46 |
| SD 95% CrI upper bound | - | 1.08 | 1.31 |

CrI, credible interval. DIC, deviance information criterion. SD, standard deviation.

### Table B.4.3. Network meta-analysis models, individual interventions, myocarditis.

|  | Fixed effects model | Random effects model | Unrelated mean effect model |
| --- | --- | --- | --- |
| Data points | 45 | 45 | 45 |
| Posterior mean residual deviance | 40.97 | 42.59 | 38.93 |
| pD | 28.3 | 23.33 | 23.62 |
| DIC | 69.28 | 65.92 | 62.55 |
| Tau | - | 0.29 | 1.39 |
| SD | - | 0.21 | 1.2 |
| SD 95% CrI lower bound | - | 0.04 | 0.07 |
| SD 95% CrI upper bound | - | 1.01 | 3.79 |

CrI, credible interval. DIC, deviance information criterion. SD, standard deviation.

### Table B.4.4. Network meta-analysis models, individual interventions, colitis.

|  | Fixed effects model | Random effects model | Unrelated mean effect model |
| --- | --- | --- | --- |
| Data points | 174 | 174 | 174 |
| Posterior mean residual deviance | 183.15 | 176.04 | 163.9 |
| pD | 104.26 | 93.75 | 99.02 |
| DIC | 287.4 | 269.8 | 262.93 |
| Tau | - | 0.22 | 0.31 |
| SD | - | 0.18 | 0.28 |
| SD 95% CrI lower bound | - | 0.04 | 0.02 |
| SD 95% CrI upper bound | - | 0.59 | 0.81 |

CrI, credible interval. DIC, deviance information criterion. SD, standard deviation.

### Table B.4.5. Network meta-analysis models, individual interventions, nephritis.

|  | Fixed effects model | Random effects model | Unrelated mean effect model |
| --- | --- | --- | --- |
| Data points | 84 | 84 | 84 |
| Posterior mean residual deviance | 72.47 | 71.64 | 71 |
| pD | 52.35 | 42.34 | 44.88 |
| DIC | 124.82 | 113.99 | 115.88 |
| Tau | - | 0.22 | 0.48 |
| SD | - | 0.17 | 0.38 |
| SD 95% CrI lower bound | - | 0.03 | 0.01 |
| SD 95% CrI upper bound | - | 0.66 | 1.43 |

CrI, credible interval. DIC, deviance information criterion. SD, standard deviation.

### Table B.4.6. Network meta-analysis models, individual interventions, pancreatitis.

|  | Fixed effects model | Random effects model | Unrelated mean effect model |
| --- | --- | --- | --- |
| Data points | 89 | 89 | 89 |
| Posterior mean residual deviance | 96.42 | 90.04 | 86.1 |
| pD | 56.01 | 46.78 | 50.71 |
| DIC | 152.43 | 136.82 | 136.81 |
| Tau | - | 0.25 | 0.61 |
| SD | - | 0.19 | 0.54 |
| SD 95% CrI lower bound | - | 0.04 | 0.02 |
| SD 95% CrI upper bound | - | 0.74 | 1.62 |

CrI, credible interval. DIC, deviance information criterion. SD, standard deviation.

### Table B.4.7. Network meta-analysis models, individual interventions, hepatitis.

|  | Fixed effects model | Random effects model | Unrelated mean effect model |
| --- | --- | --- | --- |
| Data points | 140 | 140 | 140 |
| Posterior mean residual deviance | 132 | 133.96 | 129.95 |
| pD | 83.06 | 69.96 | 74.64 |
| DIC | 215.05 | 203.92 | 204.6 |
| Tau | - | 0.21 | 0.37 |
| SD | - | 0.17 | 0.31 |
| SD 95% CrI lower bound | - | 0.03 | 0.02 |
| SD 95% CrI upper bound | - | 0.59 | 1.03 |

CrI, credible interval. DIC, deviance information criterion. SD, standard deviation.

### Table B.4.8. Network meta-analysis models, individual interventions, hypophysitis.

|  | Fixed effects model | Random effects model | Unrelated mean effect model |
| --- | --- | --- | --- |
| Data points | 112 | 112 | 112 |
| Posterior mean residual deviance | 87.68 | 91.08 | 93.38 |
| pD | 65.14 | 53.56 | 56.69 |
| DIC | 152.82 | 144.65 | 150.06 |
| Tau | - | 0.2 | 0.36 |
| SD | - | 0.16 | 0.29 |
| SD 95% CrI lower bound | - | 0.03 | 0.01 |
| SD 95% CrI upper bound | - | 0.55 | 1.08 |

CrI, credible interval. DIC, deviance information criterion. SD, standard deviation.

## Figure B.4. Pairwise meta-analyses

### Figure B.4.1. Pairwise meta-analysis, overall immune-related serious adverse events


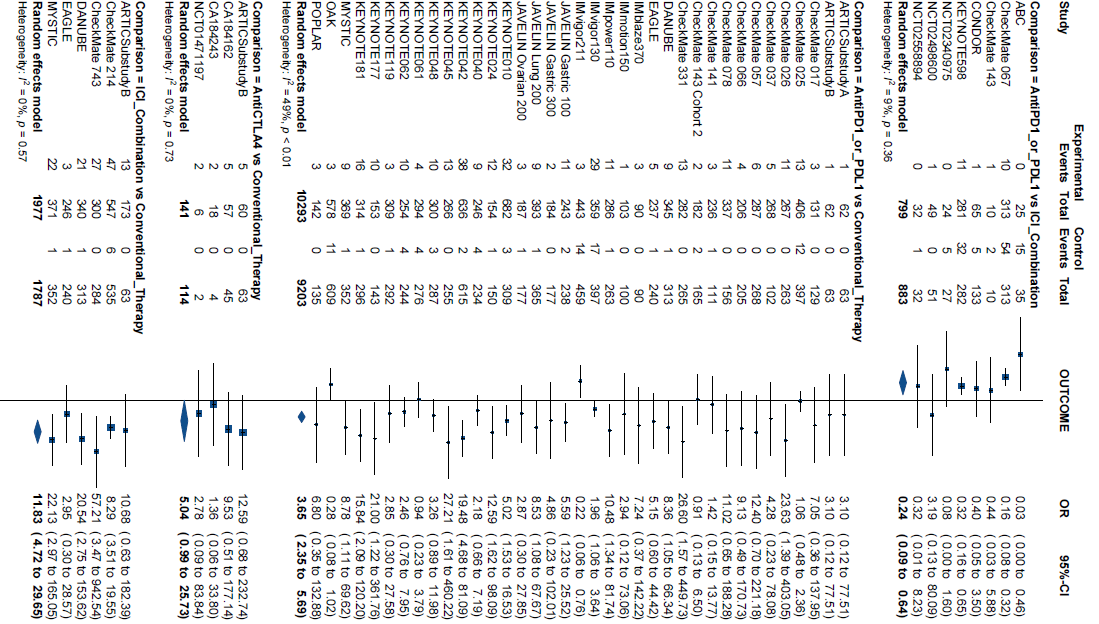


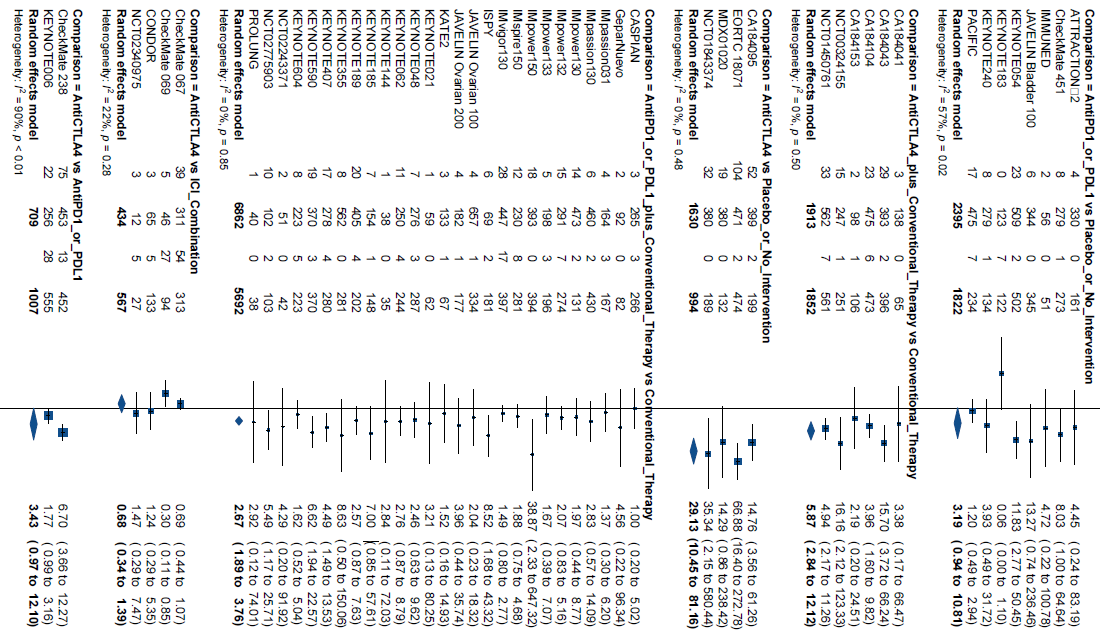


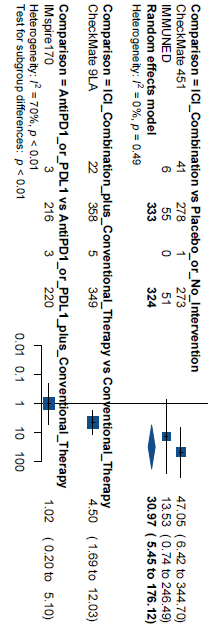


### Figure B.4.2. Pairwise meta-analysis, pneumonitis.


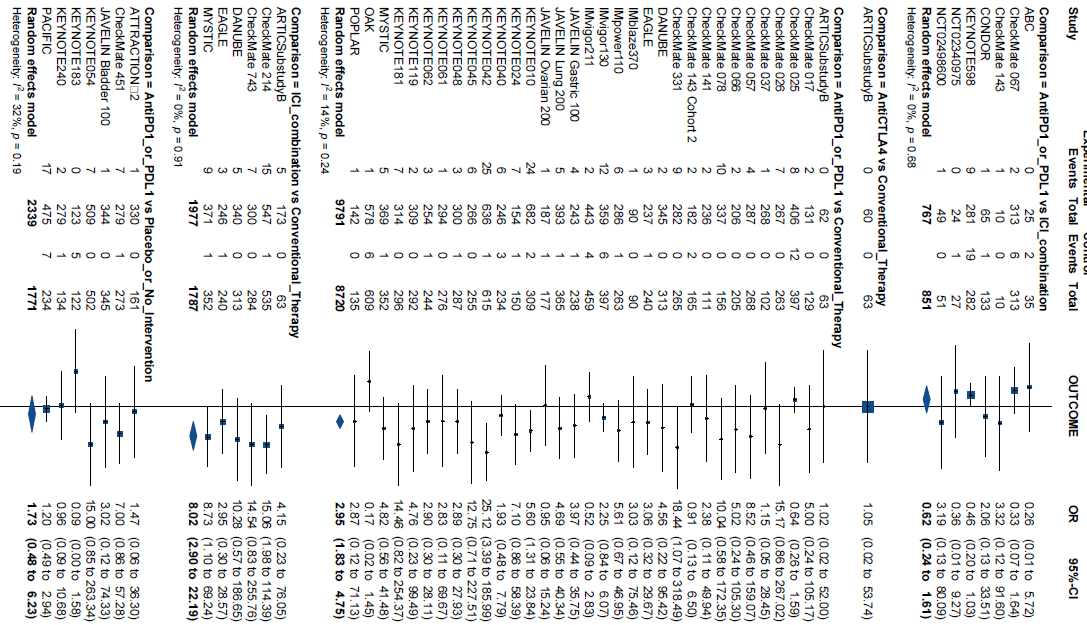


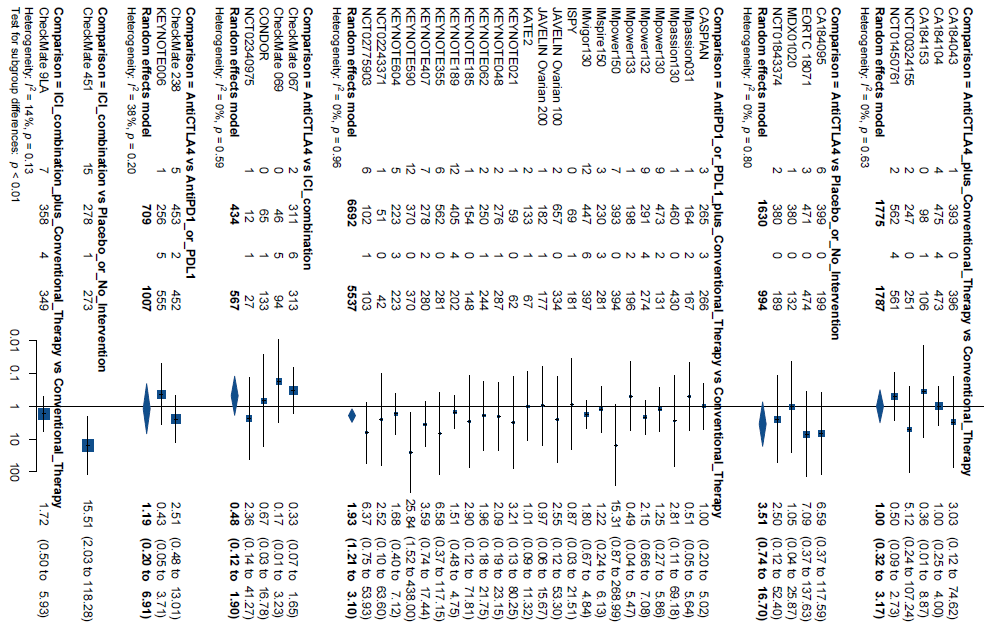


### Figure B.4.3. Pairwise meta-analyses, myocarditis.


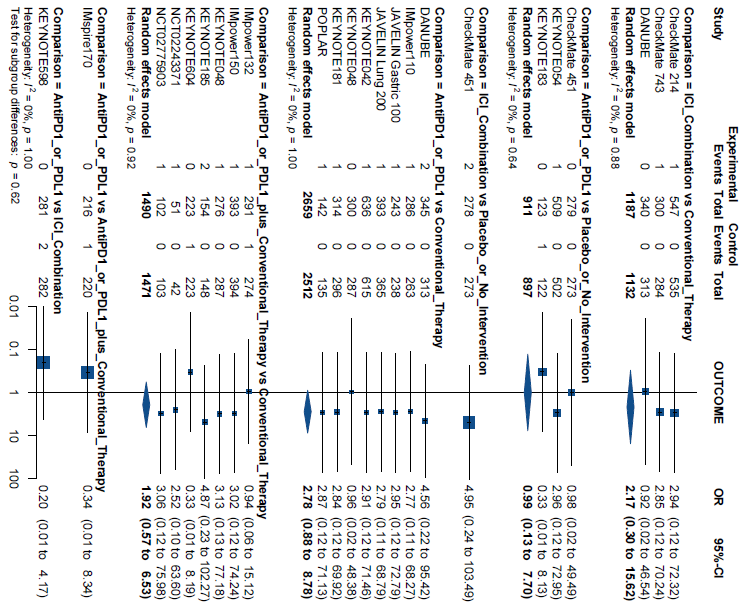


### Figure B.4.4. Pairwise meta-analyses, colitis.


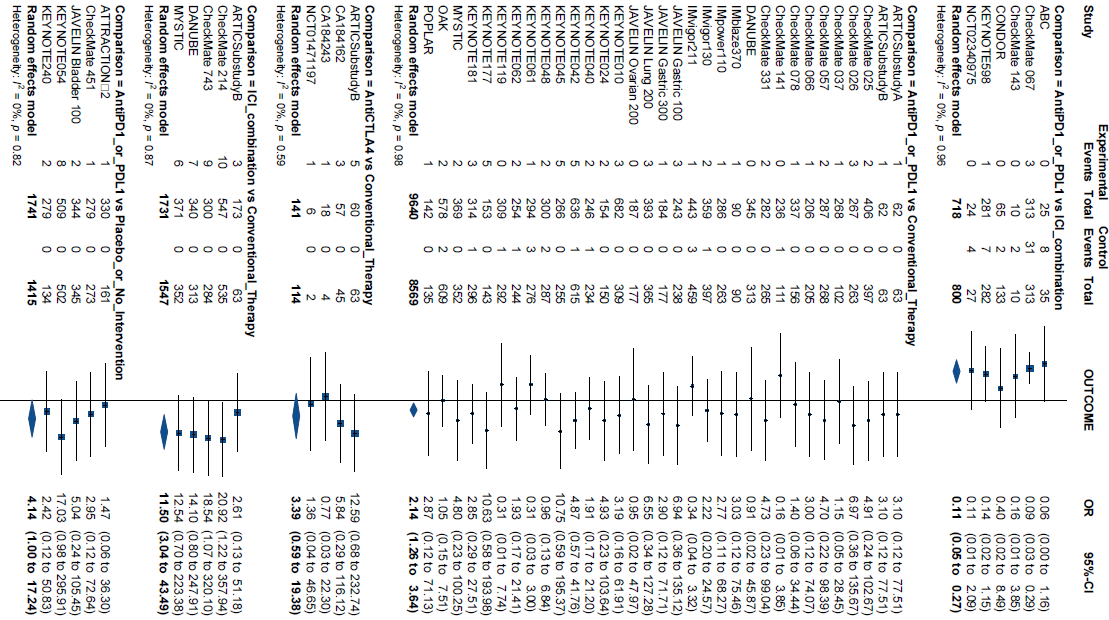

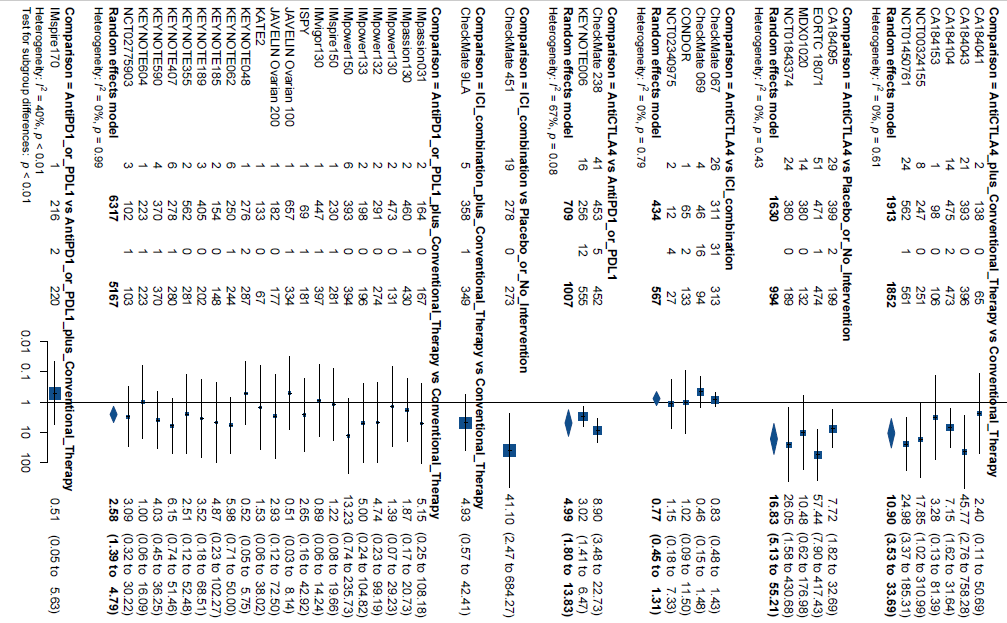


### Figure B.4.5. Pairwise meta-analyses, nephritis.


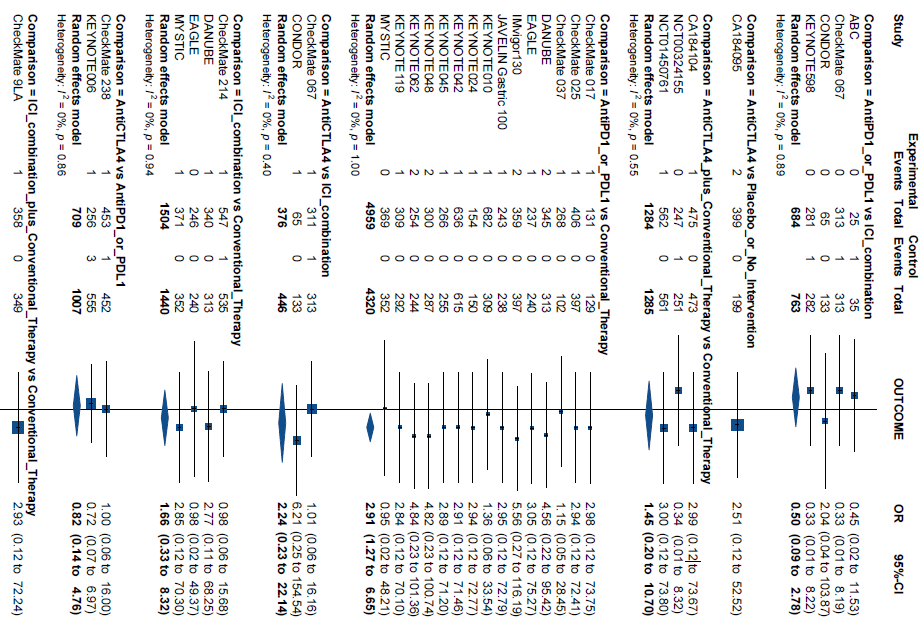


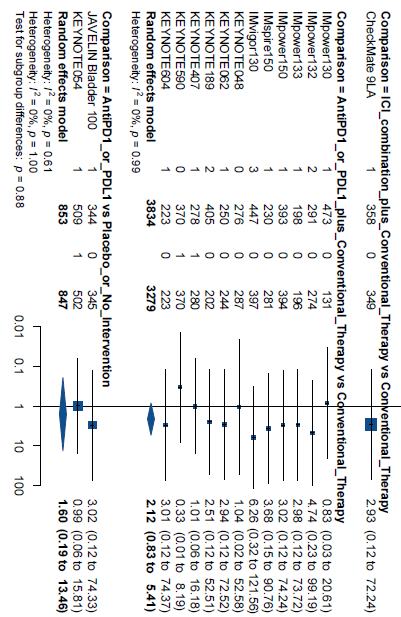


### Figure B.4.6. Pairwise meta-analyses, pancreatitis.


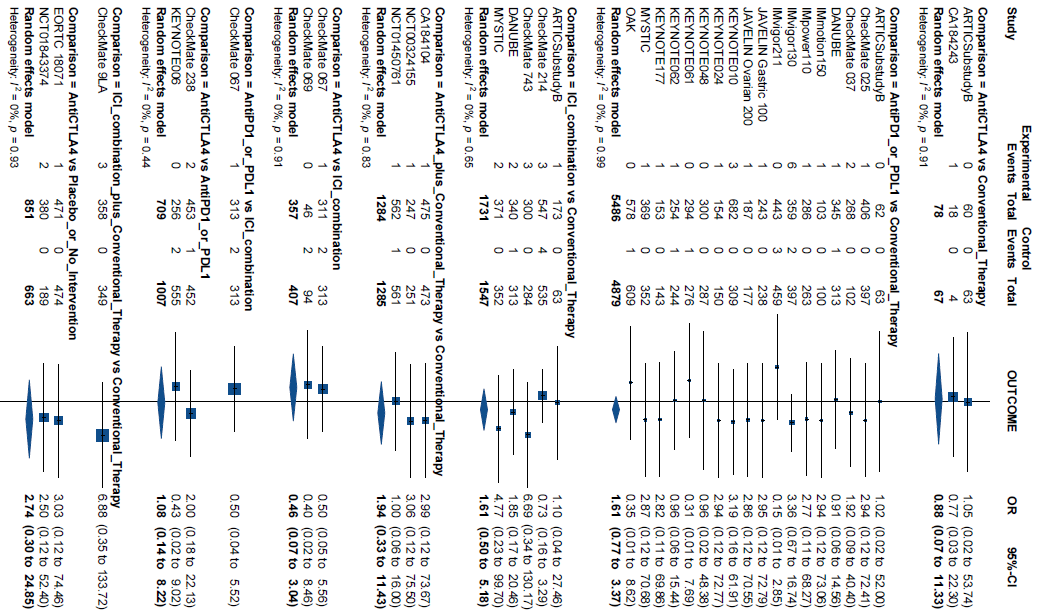


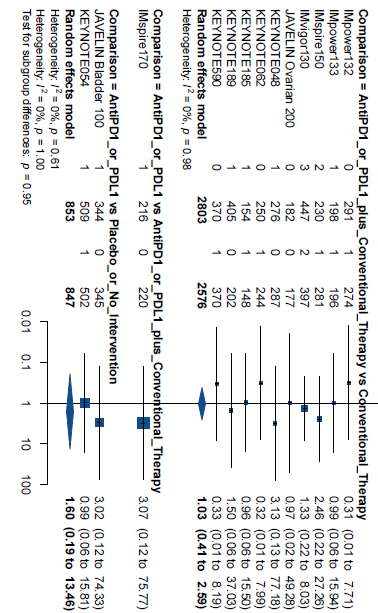


### Figure B.4.7. Pairwise meta-analyses, hepatitis.


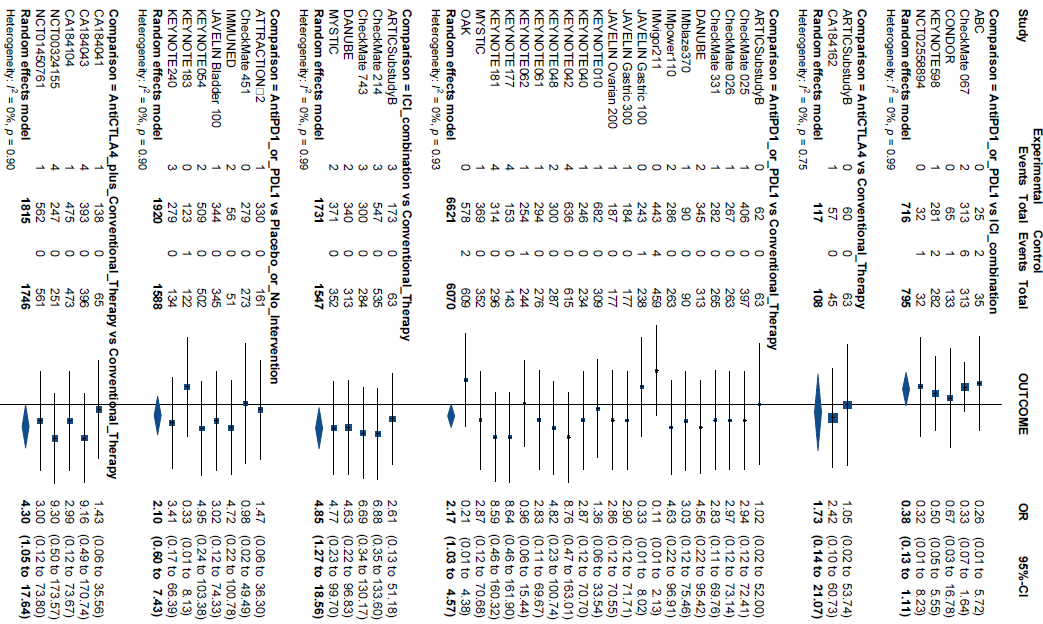


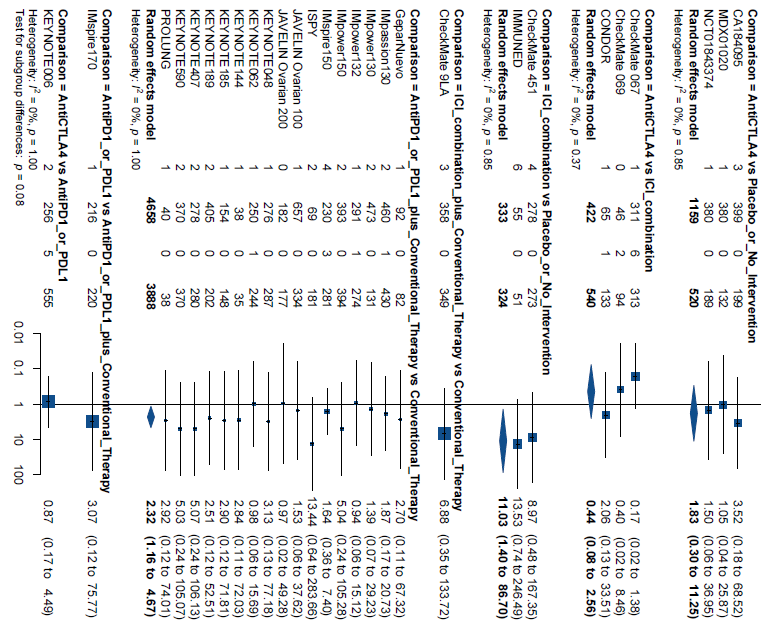


### Figure B.4.8. Pairwise meta-analyses, hypophysitis.


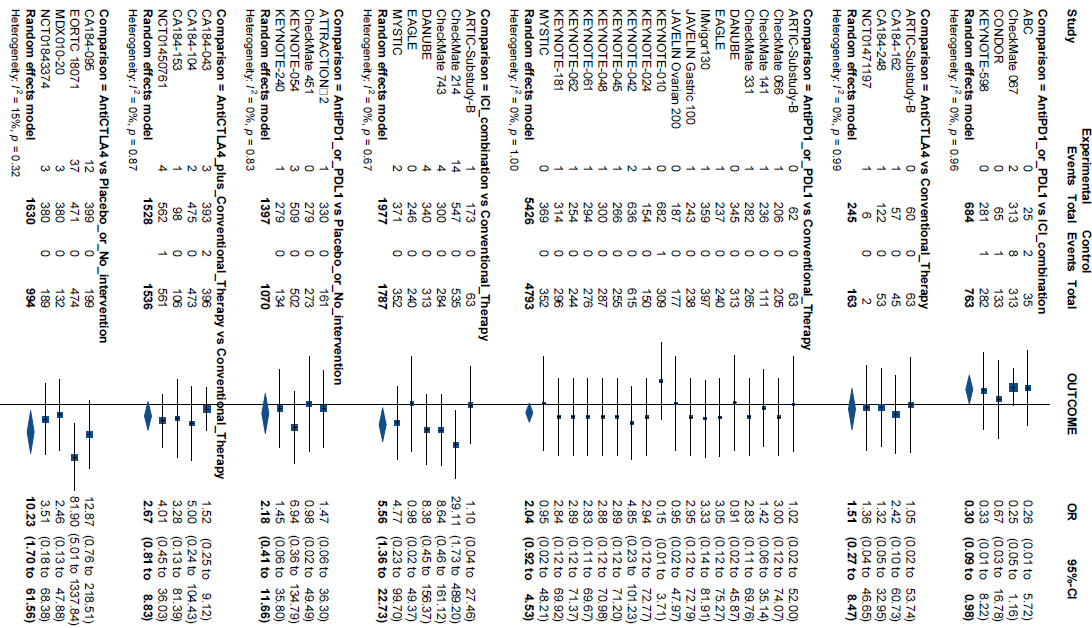


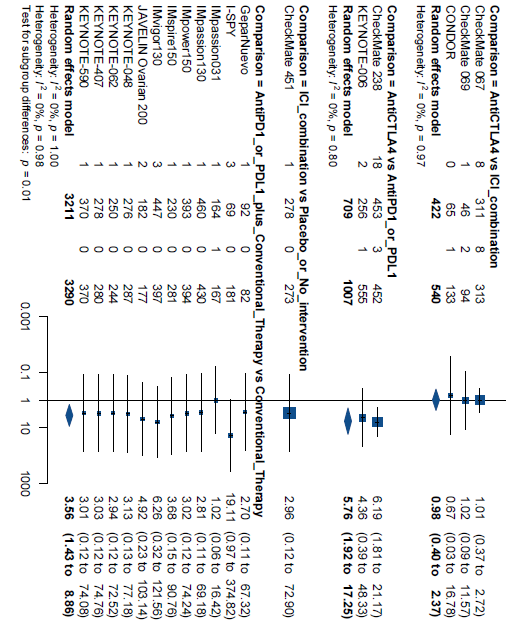


## Figure B.5. Nodesplit figures

### Figure B.5.1. Nodesplit figure, overall immune-related serious adverse events


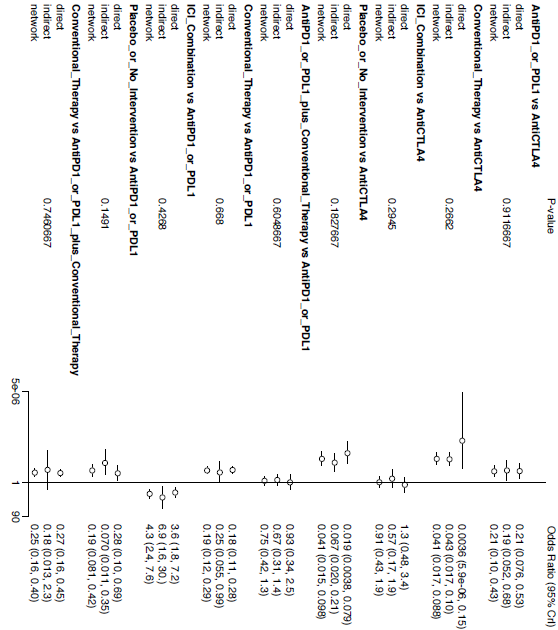


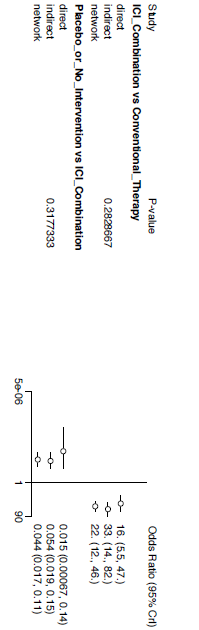


### Figure B.5.2. Nodesplit figure, pneumonitis.

### Figure B.5.3. Nodesplit figure, myocarditis.

### Figure B.5.4. Nodesplit figure, colitis.

### Figure B.5.5. Nodesplit figure, nephritis.


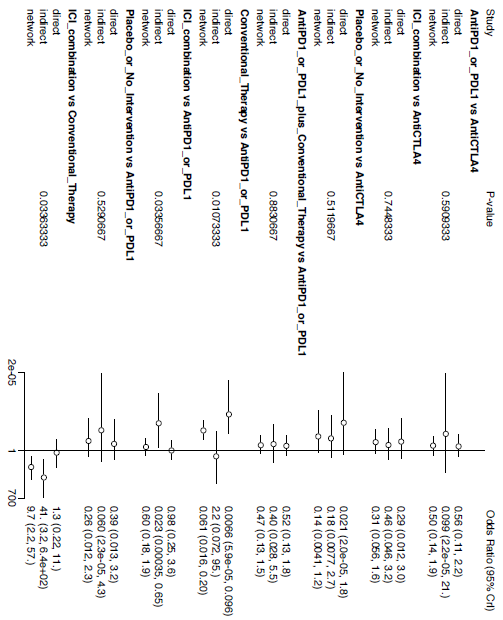


### Figure B.5.6. Nodesplit figure, pancreatitis.

### Figure B.5.7. Nodesplit figure, hepatitis.

### Figure B.5.8. Nodesplit figure, hypophysitis.

##

## Table B.5. Meta regression models and model fit according to treatment modalities

The following analyses were based on the treatment modalities, though only for networks with at least 40 trials, as smaller networks are not adequately powered to detect interaction effects. However, we present model fit statistics for outcomes where the meta-regression analyses were not conducted.

### Table B.5.1. Network meta-analysis regression models, treatment modalities, overall immune-related serious adverse events.

|  | Fixed effects model | Random effects model | Unrelated mean effect model | OS regression model | PFS regression model | Bias regression model | Female regression model |
| --- | --- | --- | --- | --- | --- | --- | --- |
| Data points | 206 | 206 | 206 | 145 | 160 | 206 | 206 |
| Posterior mean residual deviance | 361.88 | 208.95 | 206.26 | 146.6 | 164.36 | 206.36 | 209.15 |
| pD | 105.77 | 143.83 | 147.1 | 107.55 | 119.95 | 161.34 | 151.16 |
| DIC | 467.66 | 352.78 | 353.36 | 254.15 | 284.31 | 365.82 | 360.31 |
| Tau | - | 0.91 | 0.98 | 0.96 | 0.96 | 1.16 | 1.00 |
| SD | - | 0.91 | 0.97 | 0.95 | 0.94 | 1.13 | 0.99 |
| SD 95% CrI lower bound | - | 0.68 | 0.72 | 0.66 | 0.66 | 0.98 | 0.74 |
| SD 95% CrI upper bound | - | 1.19 | 1.3 | 1.32 | 1.32 | 1.48 | 1.31 |
| Beta | - | - | - | 0.16 | 0.16 | -0.5 | 0.23 |
| Beta 95% CrI lower bound | - | - | - | -0.7 | -0.7 | -1.72 | -0.49 |
| Beta 95% CrI upper bound | - | - | - | 1.06 | 1.06 | 0.64 | 0.96 |

CrI, credible interval. DIC, deviance information criterion. OS, overall survival. PFS, progression-free survival. SD, standard deviation.

### Table B.5.2. Network meta-analysis regression models, treatment modalities, pneumonitis.

|  | Fixed effects model | Random effects model | Unrelated mean effect model | OS regression model | PFS regression model | Bias regression model | Female regression model |
| --- | --- | --- | --- | --- | --- | --- | --- |
| Data points | 177 | 177 | 177 | 177 | 144 | 177 | 177 |
| Posterior mean residual deviance | 245.01 | 184.86 | 175.9 | 182.49 | 147.69 | 182.57 | 183.05 |
| pD | 93.32 | 107.78 | 112.07 | 121.2 | 100.8 | 120.9 | 121.14 |
| DIC | 338.33 | 292.64 | 287.97 | 303.69 | 248.49 | 303.46 | 304.2 |
| Tau | - | 0.78 | 0.93 | 1.00 | 1.04 | 0.99 | 0.99 |
| SD | - | 0.78 | 0.91 | 0.99 | 1.03 | 0.97 | 0.98 |
| SD 95% CrI lower bound | - | 0.41 | 0.55 | 0.6 | 0.62 | 0.59 | 0.59 |
| SD 95% CrI upper bound | - | 1.18 | 1.37 | 1.48 | 1.57 | 1.47 | 1.47 |
| Beta | - | - | - | -0.03 | 0.07 | -0.47 | -0.12 |
| Beta 95% CrI lower bound | - | - | - | -0.94 | -0.97 | -1.69 | -1.06 |
| Beta 95% CrI upper bound | - | - | - | 0.9 | 1.12 | 0.68 | 0.88 |

CrI, credible interval. DIC, deviance information criterion. OS, overall survival. PFS, progression-free survival. SD, standard deviation.

### Table B.5.3. Network meta-analysis models, treatment modalities, myocarditis.

|  | Fixed effects model | Random effects model | Unrelated mean effect model |
| --- | --- | --- | --- |
| Data points | 45 | 45 | 45 |
| Posterior mean residual deviance | 51.22 | 45.59 | 36.65 |
| pD | 27.87 | 21.97 | 22.75 |
| DIC | 79.09 | 67.56 | 59.4 |
| Tau | - | 0.34 | 1.78 |
| SD | - | 0.24 | 1.65 |
| SD 95% CrI lower bound | - | 0.04 | 0.09 |
| SD 95% CrI upper bound | - | 1.25 | 4.27 |

CrI, credible interval. DIC, deviance information criterion. SD, standard deviation.

### Table B.5.4. Network meta-analysis regression models, treatment modalities, colitis.

|  | Fixed effects model | Random effects model | Unrelated mean effect model | OS regression model | PFS regression model | Bias regression model | Female regression model |
| --- | --- | --- | --- | --- | --- | --- | --- |
| Data points | 174 | 174 | 174 | 174 | 174 | 174 | 174 |
| Posterior mean residual deviance | 185.25 | 170.52 | 160.7 | 168.63 | 168.48 | 167.68 | 168.74 |
| pD | 93.27 | 89.93 | 96.54 | 106.84 | 106.91 | 107.21 | 107.86 |
| DIC | 278.51 | 260.44 | 257.24 | 275.47 | 275.39 | 274.89 | 276.6 |
| Tau | - | 0.32 | 0.56 | 0.61 | 0.62 | 0.65 | 0.66 |
| SD | - | 0.27 | 0.56 | 0.61 | 0.61 | 0.63 | 0.64 |
| SD 95% CrI lower bound | - | 0.04 | 0.08 | 0.04 | 0.08 | 0.11 | 0.11 |
| SD 95% CrI upper bound | - | 0.81 | 1.09 | 1.23 | 1.22 | 1.25 | 1.30 |
| Beta | - | - | - | -0.87 | -0.87 | -0.88 | -0.05 |
| Beta 95% CrI lower bound | - | - | - | -2.38 | -2.41 | -2.4 | -1.04 |
| Beta 95% CrI upper bound | - | - | - | 0.45 | 0.45 | 0.46 | 1.03 |

CrI, credible interval. DIC, deviance information criterion. OS, overall survival. PFS, progression-free survival. SD, standard deviation.

### Table B.5.5. Network meta-analysis models, treatment modalities, nephritis.

|  | Fixed effects model | Random effects model | Unrelated mean effect model |
| --- | --- | --- | --- |
| Data points | 84 | 84 | 84 |
| Posterior mean residual deviance | 79.4 | 72.17 | 66.59 |
| pD | 49.02 | 40.62 | 44.07 |
| DIC | 128.42 | 112.79 | 110.66 |
| Tau | - | 0.29 | 0.87 |
| SD | - | 0.21 | 0.72 |
| SD 95% CrI lower bound | - | 0.03 | 0.03 |
| SD 95% CrI upper bound | - | 0.99 | 2.57 |

CrI, credible interval. DIC, deviance information criterion. SD, standard deviation.

### Table B.5.6. Network meta-analysis models, treatment modalities, pancreatitis.

|  | Fixed effects model | Random effects model | Unrelated mean effect model |
| --- | --- | --- | --- |
| Data points | 89 | 89 | 89 |
| Posterior mean residual deviance | 93.62 | 87.1 | 85.02 |
| pD | 50.12 | 43.03 | 47.57 |
| DIC | 143.74 | 130.13 | 132.59 |
| Tau | - | 0.24 | 0.54 |
| SD | - | 0.19 | 0.46 |
| SD 95% CrI lower bound | - | 0.03 | 0.02 |
| SD 95% CrI upper bound | - | 0.75 | 1.52 |

CrI, credible interval. DIC, deviance information criterion. SD, standard deviation.

### Table B.5.7. Network meta-analysis regression models, treatment modalities, hepatitis.

|  | Fixed effects model | Random effects model | Unrelated mean effect model | OS regression model | PFS regression model | Bias regression model | Female regression model |
| --- | --- | --- | --- | --- | --- | --- | --- |
| Data points | 135 | 135 | 135 | 102 | 112 | 135 | 135 |
| Posterior mean residual deviance | 137.61 | 126.57 | 116.27 | 88.39 | 99.19 | 117.06 | 125.09 |
| pD | 72.91 | 64.42 | 71.24 | 62.62 | 70.09 | 83.72 | 88.91 |
| DIC | 210.52 | 190.99 | 187.51 | 151.01 | 169.27 | 200.78 | 214 |
| Tau | - | 0.32 | 0.99 | 1.3 | 1.41 | 1.39 | 1.32 |
| SD | - | 0.24 | 0.97 | 1.33 | 1.46 | 1.39 | 1.29 |
| SD 95% CrI lower bound | - | 0.04 | 0.06 | 0.23 | 0.3 | 0.22 | 0.33 |
| SD 95% CrI upper bound | - | 1.01 | 2.1 | 2.17 | 2.18 | 2.49 | 2.37 |
| Beta | - | - | - | 0.08 | -0.05 | -0.9 | 0.84 |
| Beta 95% CrI lower bound | - | - | - | -1.87 | -1.6 | -4.07 | -0.75 |
| Beta 95% CrI upper bound | - | - | - | 2.05 | 1.5 | 1.5 | 2.74 |

CrI, credible interval. DIC, deviance information criterion. OS, overall survival. PFS, progression-free survival. SD, standard deviation.

### Table B.5.8. Network meta-analysis regression models, treatment modalities, hypophysitis.

|  | Fixed effects model | Random effects model | Unrelated mean effect model | OS regression model | PFS regression model | Bias regression model | Female regression model |
| --- | --- | --- | --- | --- | --- | --- | --- |
| Data points | 112 | 112 | 112 | 78 | 85 | 112 | 112 |
| Posterior mean residual deviance | 87.67 | 80.43 | 83.75 | 58.89 | 64.54 | 82.44 | 82.72 |
| pD | 60.39 | 50.44 | 53.80 | 46.39 | 50.51 | 63.87 | 53.90 |
| DIC | 148.06 | 130.87 | 137.54 | 105.28 | 115.05 | 146.31 | 136.62 |
| Tau | - | 0.19 | 0.40 | 1.35 | 1.18 | 0.67 | 0.87 |
| SD | - | 0.16 | 0.32 | 1.40 | 0.94 | 0.66 | 1.02 |
| SD 95% CrI lower bound | - | 0.03 | 0.02 | 0.07 | 0.04 | 0.22 | 0.08 |
| SD 95% CrI upper bound | - | 0.54 | 1.20 | 2.50 | 3.18 | 1.10 | 2.63 |
| Beta | - | - | - | 1.30 | 1.36 | -0.88 | 0.84 |
| Beta 95% CrI lower bound | - | - | - | -2.11 | -2.15 | -4.09 | -0.75 |
| Beta 95% CrI upper bound | - | - | - | 9.23 | 8.2 | 1.48 | 2.72 |

CrI, credible interval. DIC, deviance information criterion. OS, overall survival. PFS, progression-free survival. SD, standard deviation.

## Table B.6. Assessment of publication bias

| Outcome | Degrees of freedom | P value |
| --- | --- | --- |
| Overall immune-related serious adverse events | 108 | 0.08 |
| Pneumonitis | 91 | 0.23 |
| Myocarditis | 19 | 0.99 |
| Colitis | **89** | **0.02** |
| Nephritis | 40 | 0.36 |
| Pancreatitis | 43 | 0.59 |
| Hepatitis | 67 | 0.88 |
| Hypophysitis | 53 | 0.31 |

Bold indicates statistical significance.

##

## Table B.7. Network meta-analysis results

Below are presented the league tables for each outcome assessed, according to individual intervention, as well as the estimated absolute rate for each outcome, extrapolated according to the GRADE methodology.

### Table B.7.1. League table, overall immune-related serious adverse events.

| Atezolizumab | 1.43 (0.53 to 3.72) | 4.59 (1.04 to 21.75) | 3.1 (0.44 to 24.35) | 0.55 (0.24 to 1.23) | 2.05 (0.54 to 7.62) | 1.6 (0.31 to 8.51) | 6.29 (1.62 to 24.92) | 7.64 (1.63 to 35.18) | 2.82 (0.98 to 8.31) | 5.41 (0.13 to 894.65) | 14.37 (4.79 to 42.98) | 3.36 (1.03 to 10.66) | 2.82 (1.02 to 7.47) | 2.37 (0.81 to 6.62) | 8.72 (1.21 to 62.6) | 0.57 (0.17 to 1.81) | 10.64 (2.21 to 53.75) |
| --- | --- | --- | --- | --- | --- | --- | --- | --- | --- | --- | --- | --- | --- | --- | --- | --- | --- |
| 0.7 (0.27 to 1.88) | Atezolizumab plus Conventional Therapy | 3.19 (0.8 to 14.03) | 2.17 (0.34 to 16.01) | 0.39 (0.2 to 0.73) | 1.44 (0.42 to 4.94) | 1.12 (0.24 to 5.59) | 4.4 (1.28 to 15.93) | 5.36 (1.26 to 23.06) | 1.98 (0.79 to 5.18) | 3.76 (0.1 to 625.04) | 10.1 (3.85 to 26.53) | 2.36 (0.82 to 6.7) | 1.98 (0.85 to 4.44) | 1.65 (0.65 to 4.1) | 6.13 (0.9 to 40.64) | 0.4 (0.14 to 1.12) | 7.48 (1.73 to 34.69) |
| **0.22 (0.05 to 0.96)** | 0.31 (0.07 to 1.25) | Avelumab | 0.68 (0.11 to 4.27) | 0.12 (0.03 to 0.4) | 0.45 (0.09 to 2.19) | 0.35 (0.05 to 2.36) | 1.38 (0.26 to 6.97) | 1.68 (0.27 to 9.78) | 0.62 (0.14 to 2.5) | 1.18 (0.03 to 209.47) | 3.16 (0.71 to 12.71) | 0.74 (0.15 to 3.16) | 0.62 (0.15 to 2.27) | 0.52 (0.12 to 2.02) | 1.91 (0.21 to 16.43) | 0.12 (0.03 to 0.5) | 2.33 (0.37 to 14.2) |
| 0.32 (0.04 to 2.29) | 0.46 (0.06 to 2.97) | 1.46 (0.23 to 8.98) | Avelumab plus Conventional Therapy | 0.18 (0.03 to 1.04) | 0.66 (0.08 to 5.1) | 0.51 (0.05 to 5.23) | 2.04 (0.24 to 16.13) | 2.46 (0.25 to 22.82) | 0.92 (0.12 to 6.22) | 1.78 (0.03 to 355.81) | 4.66 (0.62 to 32.19) | 1.09 (0.14 to 7.63) | 0.9 (0.13 to 5.7) | 0.75 (0.1 to 4.97) | 2.78 (0.21 to 35.16) | 0.18 (0.02 to 1.25) | 3.42 (0.36 to 32.13) |
| 1.8 (0.81 to 4.26) | **2.57 (1.36 to 5.08)** | **8.23 (2.49 to 31.87)** | 5.59 (0.96 to 38.09) | Conventional Therapy | 3.69 (1.36 to 10.67) | 2.88 (0.71 to 12.59) | 11.37 (3.96 to 34.7) | 13.74 (3.84 to 52.37) | 5.09 (2.65 to 10.7) | 9.77 (0.28 to 1558.52) | 26 (12.74 to 55.12) | 6.1 (2.74 to 13.88) | 5.09 (2.94 to 8.87) | 4.26 (2.24 to 8.25) | 15.66 (2.66 to 95.23) | 1.03 (0.44 to 2.36) | 19.17 (5.21 to 78.77) |
| 0.49 (0.13 to 1.85) | 0.7 (0.2 to 2.36) | 2.23 (0.46 to 11.66) | 1.51 (0.2 to 12.72) | **0.27 (0.09 to 0.74)** | Durvalumab | 0.78 (0.13 to 4.66) | 3.07 (1.35 to 7.29) | 3.74 (0.74 to 18.56) | 1.39 (0.42 to 4.59) | 2.66 (0.06 to 469.61) | 7.05 (2.14 to 22.89) | 1.65 (0.44 to 6.04) | 1.38 (0.44 to 4.2) | 1.15 (0.34 to 3.86) | 4.24 (0.55 to 31.96) | 0.28 (0.09 to 0.83) | 5.24 (1.57 to 17.81) |
| 0.63 (0.12 to 3.24) | 0.9 (0.18 to 4.18) | 2.87 (0.42 to 19.74) | 1.95 (0.19 to 20.92) | 0.35 (0.08 to 1.41) | 1.28 (0.21 to 7.53) | Durvalumab plus Conventional Therapy | 3.94 (0.65 to 24) | 4.76 (0.69 to 32.29) | 1.77 (0.36 to 8.74) | 3.41 (0.07 to 623.4) | 9.05 (1.75 to 44.51) | 2.11 (0.39 to 10.79) | 1.77 (0.36 to 7.95) | 1.49 (0.3 to 6.89) | 5.48 (0.53 to 52.55) | 0.36 (0.07 to 1.85) | 6.7 (0.93 to 50.09) |
| **0.16 (0.04 to 0.62)** | **0.23 (0.06 to 0.78)** | 0.73 (0.14 to 3.9) | 0.49 (0.06 to 4.21) | **0.09 (0.03 to 0.25)** | **0.33 (0.14 to 0.74)** | 0.25 (0.04 to 1.55) | Durvalumab plus Tremelimumab | 1.21 (0.23 to 6.27) | 0.45 (0.13 to 1.57) | 0.86 (0.02 to 150.11) | 2.28 (0.65 to 7.75) | 0.53 (0.14 to 2.02) | 0.45 (0.13 to 1.42) | 0.37 (0.1 to 1.29) | 1.38 (0.17 to 10.75) | 0.09 (0.03 to 0.29) | 1.7 (0.55 to 5.17) |
| **0.13 (0.03 to 0.61)** | **0.19 (0.04 to 0.79)** | 0.59 (0.1 to 3.76) | 0.41 (0.04 to 4.03) | **0.07 (0.02 to 0.26)** | 0.27 (0.05 to 1.34) | 0.21 (0.03 to 1.45) | 0.82 (0.16 to 4.33) | Ipilimumab | 0.37 (0.11 to 1.28) | 0.72 (0.02 to 122.66) | 1.88 (0.6 to 5.98) | 0.44 (0.09 to 2.01) | 0.37 (0.09 to 1.4) | 0.31 (0.07 to 1.29) | 1.14 (0.13 to 9.96) | 0.07 (0.02 to 0.29) | 1.4 (0.23 to 8.94) |
| 0.35 (0.12 to 1.02) | 0.5 (0.19 to 1.26) | 1.61 (0.4 to 7.04) | 1.09 (0.16 to 8.11) | **0.2 (0.09 to 0.38)** | 0.72 (0.22 to 2.39) | 0.56 (0.11 to 2.8) | 2.23 (0.64 to 7.77) | 2.71 (0.78 to 9.09) | Nivolumab | 1.9 (0.05 to 308.83) | 5.09 (2.55 to 9.84) | 1.19 (0.4 to 3.38) | 1 (0.42 to 2.22) | 0.83 (0.31 to 2.09) | 3.09 (0.45 to 19.62) | 0.2 (0.08 to 0.49) | 3.74 (0.87 to 17.19) |
| 0.18 (0 to 7.42) | 0.27 (0 to 10.03) | 0.84 (0 to 38.79) | 0.56 (0 to 34.21) | 0.1 (0 to 3.58) | 0.38 (0 to 15.77) | 0.29 (0 to 14.39) | 1.16 (0.01 to 49.01) | 1.39 (0.01 to 65) | 0.53 (0 to 20.1) | Nivolumab plus Conventional Therapy | 2.65 (0.02 to 104.38) | 0.61 (0 to 24.37) | 0.52 (0 to 19.65) | 0.44 (0 to 16.04) | 1.56 (0.01 to 89.22) | 0.1 (0 to 4.11) | 1.96 (0.01 to 94.6) |
| **0.07 (0.02 to 0.21)** | **0.1 (0.04 to 0.26)** | 0.32 (0.08 to 1.42) | 0.21 (0.03 to 1.62) | **0.04 (0.02 to 0.08)** | **0.14 (0.04 to 0.47)** | **0.11 (0.02 to 0.57)** | 0.44 (0.13 to 1.53) | 0.53 (0.17 to 1.67) | 0.2 (0.1 to 0.39) | 0.38 (0.01 to 59.22) | Nivolumab plus Ipilimumab | 0.23 (0.08 to 0.69) | 0.2 (0.09 to 0.43) | 0.16 (0.06 to 0.42) | 0.61 (0.09 to 3.92) | 0.04 (0.02 to 0.09) | 0.74 (0.18 to 3.33) |
| **0.3 (0.09 to 0.97)** | 0.42 (0.15 to 1.21) | 1.36 (0.32 to 6.54) | 0.91 (0.13 to 7.27) | **0.16 (0.07 to 0.36)** | 0.61 (0.17 to 2.27) | 0.47 (0.09 to 2.55) | 1.88 (0.49 to 7.35) | 2.26 (0.5 to 10.9) | 0.84 (0.3 to 2.52) | 1.63 (0.04 to 267.74) | **4.26 (1.45 to 12.88)** | Nivolumab plus Ipilimumab plus Conventional Therapy | 0.83 (0.31 to 2.23) | 0.7 (0.25 to 1.99) | 2.59 (0.37 to 18.64) | 0.17 (0.05 to 0.53) | 3.16 (0.68 to 16.06) |
| **0.35 (0.13 to 0.98)** | 0.5 (0.23 to 1.18) | 1.62 (0.44 to 6.79) | 1.1 (0.18 to 7.82) | **0.2 (0.11 to 0.34)** | 0.72 (0.24 to 2.3) | 0.57 (0.13 to 2.75) | 2.24 (0.7 to 7.54) | 2.71 (0.72 to 10.7) | 1 (0.45 to 2.37) | 1.93 (0.05 to 316.16) | **5.09 (2.3 to 11.73)** | 1.2 (0.45 to 3.22) | Pembrolizumab | 0.83 (0.38 to 1.86) | 3.09 (0.57 to 17.03) | 0.2 (0.08 to 0.47) | 3.77 (0.96 to 16.56) |
| 0.42 (0.15 to 1.23) | 0.61 (0.24 to 1.54) | 1.93 (0.5 to 8.57) | 1.33 (0.2 to 9.95) | **0.23 (0.12 to 0.45)** | 0.87 (0.26 to 2.96) | 0.67 (0.15 to 3.38) | 2.68 (0.78 to 9.55) | 3.25 (0.77 to 14.32) | 1.2 (0.48 to 3.19) | 2.29 (0.06 to 379.44) | **6.12 (2.36 to 16.2)** | 1.43 (0.5 to 4.06) | 1.2 (0.54 to 2.6) | Pembrolizumab plus Conventional Therapy | 3.71 (0.57 to 24.14) | 0.24 (0.08 to 0.67) | 4.52 (1.05 to 21.42) |
| **0.11 (0.02 to 0.83)** | 0.16 (0.02 to 1.11) | 0.52 (0.06 to 4.85) | 0.36 (0.03 to 4.71) | **0.06 (0.01 to 0.38)** | 0.24 (0.03 to 1.8) | 0.18 (0.02 to 1.87) | 0.72 (0.09 to 5.81) | 0.88 (0.1 to 7.88) | 0.32 (0.05 to 2.24) | 0.64 (0.01 to 123.31) | 1.65 (0.25 to 11) | 0.39 (0.05 to 2.74) | 0.32 (0.06 to 1.77) | 0.27 (0.04 to 1.76) | Pembrolizumab plus Ipilimumab | 0.07 (0.01 to 0.43) | 1.23 (0.14 to 11.29) |
| 1.76 (0.55 to 5.9) | 2.5 (0.89 to 7.37) | **8.03 (2 to 36.81)** | 5.46 (0.8 to 42.95) | 0.97 (0.42 to 2.27) | **3.58 (1.2 to 11.28)** | 2.8 (0.54 to 15.35) | **11.11 (3.4 to 39.32)** | **13.42 (3.44 to 55.48)** | **4.96 (2.03 to 13.31)** | 9.57 (0.24 to 1619.62) | **25.21 (10.99 to 62.13)** | **5.93 (1.87 to 19.36)** | **4.95 (2.13 to 11.81)** | **4.13 (1.48 to 11.9)** | **15.36 (2.32 to 105.64)** | Placebo or No Intervention | 18.67 (4.81 to 81.92) |
| **0.09 (0.02 to 0.45)** | **0.13 (0.03 to 0.58)** | 0.43 (0.07 to 2.7) | 0.29 (0.03 to 2.8) | **0.05 (0.01 to 0.19)** | **0.19 (0.06 to 0.64)** | 0.15 (0.02 to 1.07) | 0.59 (0.19 to 1.8) | 0.72 (0.11 to 4.37) | 0.27 (0.06 to 1.15) | 0.51 (0.01 to 93.53) | 1.35 (0.3 to 5.69) | 0.32 (0.06 to 1.47) | 0.27 (0.06 to 1.04) | **0.22 (0.05 to 0.95)** | 0.81 (0.09 to 7.27) | **0.05 (0.01 to 0.21)** | Tremelimumab |

The league table show the relative effects of each intervention (the treatment on the column to the treatment of the row). The relative effects are measured as an odds ratio, with corresponding 95% credible intervals in parentheses. Bold indicates statistical significance.

### Table B.7.2. Estimated absolute event rate, overall immune-related serious adverse events.

| Intervention | Proportion per 10,000  (95% confidence interval) |
| --- | --- |
| Atezolizumab | 119 (54 to 279) |
| Atezolizumab plus Conventional Therapy | 170 (90 to 331) |
| Avelumab | 525 (165 to 1,769) |
| Avelumab plus Conventional Therapy | 363 (64 to 2,044) |
| Durvalumab | 242 (90 to 671) |
| Durvalumab plus Conventional Therapy | 190 (47 to 782) |
| Durvalumab plus Tremelimumab | 712 (260 to 1,896) |
| Ipilimumab | 848 (252 to 2,610) |
| Nivolumab | 331 (175 to 673) |
| Nivolumab plus Conventional Therapy | 618 (18 to 9,131) |
| Nivolumab plus Ipilimumab | 1,492 (791 to 2,710) |
| Nivolumab plus Ipilimumab plus Conventional Therapy | 395 (181 to 856) |
| Pembrolizumab | 331 (194 to 564) |
| Pembrolizumab plus Conventional Therapy | 279 (148 to 527) |
| Pembrolizumab plus Ipilimumab | 955 (176 to 3,911) |
| Ipilimumab plus conventional therapy | 421 (174 to 1,007) |
| Tremelimumab | 1,144 (339 to 3,469) |

### Table B.7.3. League table, pneumonitis.

| Atezolizumab | 1.13 (0.39, 3.38) | 3.14 (0.56, 21.47) | 2.5 (0.2, 39.72) | 0.63 (0.25, 1.56) | 1.71 (0.39, 7.94) | 1.41 (0.24, 8.98) | 3.71 (0.84, 18.2) | 4.29 (1.01, 19.85) | 0.58 (0.13, 2.7) | 3.06 (0.97, 10.87) | 2.84 (0.04, 566.99) | 8.65 (2.26, 36.44) | 1.06 (0.13, 8.54) | 3.31 (1.06, 10.55) | 1.99 (0.6, 6.7) | 7.21 (0.99, 54.4) | 0.89 (0.21, 3.5) | 1.51 (0.15, 13.69) |
| --- | --- | --- | --- | --- | --- | --- | --- | --- | --- | --- | --- | --- | --- | --- | --- | --- | --- | --- |
| 0.88 (0.30 to 2.60) | Atezolizumab plus Conventional Therapy | 2.77 (0.53 to 17.45) | 2.26 (0.19 to 31.45) | 0.56 (0.26 to 1.15) | 1.52 (0.39 to 6.15) | 1.22 (0.22 to 6.94) | 3.27 (0.81 to 14.21) | 3.79 (0.99 to 15.7) | 0.51 (0.12 to 2.14) | 2.68 (0.96 to 8.44) | 2.4 (0.03 to 438.15) | 7.58 (2.2 to 28.22) | 0.95 (0.13 to 7.27) | 2.94 (1.05 to 8.15) | 1.77 (0.59 to 5.19) | 6.36 (0.92 to 42.98) | 0.8 (0.21 to 2.75) | 1.33 (0.14 to 10.81) |
| 0.32 (0.05 to 1.77) | 0.36 (0.06 to 1.88) | Avelumab | 0.82 (0.07 to 9.09) | 0.2 (0.04 to 0.86) | 0.55 (0.07 to 3.54) | 0.44 (0.05 to 3.8) | 1.17 (0.15 to 8.07) | 1.37 (0.19 to 8.99) | 0.18 (0.02 to 1.27) | 0.98 (0.16 to 5.18) | 0.87 (0.01 to 185.66) | 2.75 (0.4 to 16.49) | 0.34 (0.03 to 3.82) | 1.07 (0.18 to 5.17) | 0.63 (0.1 to 3.26) | 2.28 (0.2 to 22.4) | 0.29 (0.04 to 1.56) | 0.47 (0.03 to 5.5) |
| 0.39 (0.03 to 4.93) | 0.44 (0.03 to 5.18) | 1.22 (0.11 to 14.65) | Avelumab plus Conventional Therapy | 0.25 (0.02 to 2.57) | 0.68 (0.04 to 9.65) | 0.55 (0.03 to 9.43) | 1.45 (0.09 to 21.33) | 1.68 (0.11 to 23.49) | 0.23 (0.01 to 3.18) | 1.2 (0.09 to 14.42) | 1.08 (0.01 to 305.58) | 3.41 (0.23 to 44.35) | 0.42 (0.02 to 8.31) | 1.3 (0.09 to 15.18) | 0.78 (0.05 to 9.03) | 2.82 (0.13 to 51.88) | 0.35 (0.02 to 4.42) | 0.58 (0.02 to 12.98) |
| 1.59 (0.65 to 4.04) | 1.79 (0.87 to 3.88) | **4.95 (1.17 to 27.09)** | 4.05 (0.39 to 52.23) | Conventional Therapy | 2.72 (0.87 to 9) | 2.19 (0.48 to 10.84) | 5.86 (1.84 to 21.24) | 6.81 (2.23 to 23.06) | 0.93 (0.28 to 3.13) | 4.83 (2.35 to 11.57) | 4.31 (0.07 to 737.54) | 13.64 (5.19 to 41) | 1.71 (0.26 to 11.37) | 5.26 (2.68 to 10.83) | 3.15 (1.48 to 7.01) | 11.4 (1.95 to 67.36) | 1.43 (0.49 to 3.86) | 2.4 (0.28 to 17.34) |
| 0.58 (0.13 to 2.56) | 0.66 (0.16 to 2.59) | 1.83 (0.28 to 13.77) | 1.48 (0.10 to 23.67) | 0.37 (0.11 to 1.14) | Durvalumab | 0.8 (0.12 to 5.62) | 2.15 (0.82 to 5.93) | 2.48 (0.55 to 12.07) | 0.34 (0.06 to 1.78) | 1.77 (0.47 to 7.2) | 1.59 (0.02 to 293.9) | 5.01 (1.16 to 22.46) | 0.63 (0.07 to 5.69) | 1.94 (0.53 to 6.93) | 1.16 (0.28 to 4.61) | 4.16 (0.5 to 32.64) | 0.52 (0.14 to 1.72) | 0.88 (0.11 to 5.68) |
| 0.72 (0.12 to 4.33) | 0.82 (0.14 to 4.46) | 2.28 (0.26 to 21.53) | 1.81 (0.11 to 34.59) | 0.46 (0.09 to 2.09) | 1.25 (0.18 to 8.52) | Durvalumab plus Conventional Therapy | 2.67 (0.36 to 19.6) | 3.13 (0.45 to 21.94) | 0.42 (0.06 to 2.94) | 2.2 (0.39 to 13.13) | 1.95 (0.02 to 409.78) | 6.22 (0.98 to 40.44) | 0.77 (0.07 to 9.3) | 2.4 (0.43 to 12.7) | 1.44 (0.25 to 7.95) | 5.09 (0.49 to 54) | 0.65 (0.09 to 3.93) | 1.08 (0.08 to 13.49) |
| 0.27 (0.06 to 1.20) | 0.31 (0.07 to 1.23) | 0.85 (0.12 to 6.5) | 0.69 (0.05 to 10.91) | **0.17 (0.05 to 0.54)** | 0.47 (0.17 to 1.22) | 0.37 (0.05 to 2.75) | Durvalumab plus Tremelimumab | 1.15 (0.24 to 5.88) | 0.16 (0.03 to 0.83) | 0.82 (0.2 to 3.38) | 0.73 (0.01 to 141.27) | 2.33 (0.51 to 10.74) | 0.29 (0.03 to 2.68) | 0.9 (0.22 to 3.32) | 0.54 (0.12 to 2.16) | 1.93 (0.22 to 15.49) | 0.24 (0.06 to 0.9) | 0.41 (0.05 to 2.38) |
| **0.23 (0.05 to 0.98)** | 0.26 (0.06 to 1.01) | 0.73 (0.11 to 5.24) | 0.60 (0.04 to 9.29) | **0.15 (0.04 to 0.45)** | 0.40 (0.08 to 1.82) | 0.32 (0.05 to 2.24) | 0.87 (0.17 to 4.24) | Ipilimumab | 0.14 (0.02 to 0.7) | 0.71 (0.23 to 2.2) | 0.64 (0.01 to 124.38) | 2 (0.63 to 6.63) | 0.25 (0.03 to 2.3) | 0.77 (0.23 to 2.51) | 0.46 (0.11 to 1.77) | 1.68 (0.21 to 12.18) | 0.21 (0.05 to 0.7) | 0.35 (0.03 to 3.07) |
| 1.73 (0.38 to 7.73) | 1.95 (0.47 to 8.10) | 5.41 (0.79 to 42.96) | 4.39 (0.31 to 71.70) | 1.08 (0.32 to 3.59) | 2.96 (0.56 to 16.16) | 2.37 (0.34 to 17.52) | **6.37 (1.21 to 36.85)** | **7.37 (1.44 to 40.19)** | Ipilimumab plus Conventional Therapy | 5.22 (1.31 to 23.29) | 4.68 (0.06 to 926.74) | 14.84 (3.14 to 74.25) | 1.85 (0.2 to 17.25) | 5.72 (1.43 to 23.23) | 3.41 (0.81 to 14.17) | 12.3 (1.44 to 104.34) | 1.55 (0.31 to 7.29) | 2.58 (0.23 to 26.17) |
| 0.33 (0.09 to 1.03) | 0.37 (0.12 to 1.04) | 1.02 (0.19 to 6.43) | 0.83 (0.07 to 11.31) | **0.21 (0.09 to 0.43)** | 0.57 (0.14 to 2.11) | 0.45 (0.08 to 2.58) | 1.21 (0.30 to 4.95) | 1.40 (0.45 to 4.29) | **0.19 (0.04 to 0.76)** | Nivolumab | 0.89 (0.01 to 155.85) | 2.81 (1.09 to 7.37) | 0.35 (0.04 to 2.62) | 1.09 (0.39 to 2.79) | 0.65 (0.21 to 1.87) | 2.35 (0.33 to 14.97) | 0.3 (0.08 to 0.87) | 0.49 (0.05 to 3.77) |
| 0.37 (0.00 to 26.45) | 0.42 (0.00 to 29.54) | 1.16 (0.01 to 99.27) | 0.92 (0.00 to 125.2) | 0.23 (0.00 to 15.32) | 0.63 (0.00 to 47.28) | 0.51 (0.00 to 44.56) | 1.37 (0.01 to 107.95) | 1.57 (0.01 to 124.15) | 0.21 (0.00 to 16.83) | 1.12 (0.01 to 82.92) | Nivolumab plus Conventional Therapy | 3.19 (0.02 to 238.19) | 0.39 (0 to 38.89) | 1.23 (0.01 to 85.81) | 0.73 (0 to 51.04) | 2.6 (0.01 to 245.74) | 0.33 (0 to 24.42) | 0.53 (0 to 59.99) |
| 0.12 (0.03 to 0.44) | **0.13 (0.04 to 0.45)** | 0.36 (0.06 to 2.50) | 0.29 (0.02 to 4.34) | **0.07 (0.02 to 0.19)** | **0.20 (0.04 to 0.86)** | 0.16 (0.02 to 1.02) | 0.43 (0.09 to 1.98) | 0.50 (0.15 to 1.59) | **0.07 (0.01 to 0.32)** | **0.36 (0.14 to 0.91)** | 0.31 (0.00 to 56.89) | Nivolumab plus Ipilimumab | 0.12 (0.01 to 1.05) | 0.39 (0.12 to 1.17) | 0.23 (0.06 to 0.79) | 0.84 (0.11 to 5.84) | 0.1 (0.03 to 0.34) | 0.17 (0.02 to 1.48) |
| 0.94 (0.11 to 7.44) | 1.06 (0.14 to 7.94) | 2.91 (0.26 to 35.53) | 2.38 (0.12 to 54.55) | 0.58 (0.09 to 3.80) | 1.60 (0.18 to 14.65) | 1.30 (0.11 to 14.97) | 3.46 (0.37 to 33.94) | 4.01 (0.43 to 37.37) | 0.54 (0.06 to 5.10) | 2.84 (0.38 to 23.10) | 2.56 (0.03 to 616.51) | 8.06 (0.96 to 69.34) | Nivolumab plus Ipilimumab plus Conventional Therapy | 3.09 (0.41 to 22.72) | 1.86 (0.24 to 14.12) | 6.68 (0.5 to 87.29) | 0.84 (0.09 to 6.79) | 1.39 (0.08 to 21.39) |
| 0.30 (0.10 to 0.96) | **0.34 (0.12 to 0.95)** | 0.94 (0.19 to 5.68) | 0.77 (0.07 to 10.57) | **0.19 (0.09 to 0.37)** | 0.52 (0.14 to 1.89) | 0.42 (0.08 to 2.33) | 1.11 (0.30 to 4.48) | 1.29 (0.40 to 4.44) | **0.17 (0.04 to 0.70)** | 0.92 (0.36 to 2.59) | 0.81 (0.01 to 147.21) | 2.59 (0.85 to 8.65) | 0.32 (0.04 to 2.44) | Pembrolizumab | 0.6 (0.23 to 1.56) | 2.15 (0.42 to 11.07) | 0.27 (0.09 to 0.74) | 0.46 (0.05 to 3.47) |
| 0.50 (0.15 to 1.67) | 0.57 (0.19 to 1.69) | 1.58 (0.31 to 9.96) | 1.28 (0.11 to 18.49) | **0.32 (0.14 to 0.68)** | 0.86 (0.22 to 3.57) | 0.70 (0.13 to 4.03) | 1.86 (0.46 to 8.20) | 2.16 (0.57 to 8.80) | 0.29 (0.07 to 1.24) | 1.53 (0.54 to 4.85) | 1.37 (0.02 to 248.49) | **4.32 (1.26 to 16.06)** | 0.54 (0.07 to 4.25) | 1.67 (0.64 to 4.39) | Pembrolizumab plus Conventional Therapy | 3.6 (0.56 to 23.66) | 0.45 (0.13 to 1.55) | 0.76 (0.08 to 6.26) |
| 0.14 (0.02 to 1.03) | 0.16 (0.02 to 1.09) | 0.44 (0.04 to 4.95) | 0.35 (0.02 to 7.82) | **0.09 (0.01 to 0.51)** | 0.24 (0.03 to 1.99) | 0.20 (0.02 to 2.02) | 0.52 (0.06 to 4.55) | 0.6 (0.08 to 4.87) | **0.08 (0.01 to 0.69)** | 0.43 (0.07 to 3.06) | 0.38 (0.00 to 84.17) | 1.19 (0.17 to 9.37) | 0.15 (0.01 to 1.99) | 0.47 (0.09 to 2.39) | 0.28 (0.04 to 1.80) | Pembrolizumab plus Ipilimumab | 0.13 (0.02 to 0.83) | 0.21 (0.01 to 2.85) |
| 1.11 (0.30 to 4.62) | 1.26 (0.36 to 4.72) | 3.50 (0.64 to 24.39) | 2.86 (0.23 to 44.69) | 0.7 (0.26 to 2.04) | 1.92 (0.58 to 6.92) | 1.54 (0.25 to 10.73) | **4.14 (1.12 to 17.15)** | **4.79 (1.42 to 19.07)** | 0.65 (0.14 to 3.25) | **3.38 (1.15 to 12.06)** | 3.05 (0.04 to 563.37) | **9.56 (2.91 to 37.47)** | 1.19 (0.15 to 10.73) | **3.69 (1.35 to 11.01)** | 2.22 (0.65 to 7.99) | **7.96 (1.21 to 57.48)** | Placebo or No Intervention | 1.67 (0.21 to 12.51) |
| 0.66 (0.08 to 7.12) | 0.75 (0.09 to 7.37) | 2.13 (0.18 to 30.12) | 1.72 (0.08 to 47.09) | 0.42 (0.06 to 3.61) | 1.14 (0.18 to 9.49) | 0.93 (0.07 to 13.3) | 2.41 (0.42 to 19.97) | 2.89 (0.33 to 30.39) | 0.39 (0.04 to 4.42) | 2.05 (0.27 to 19.97) | 1.89 (0.02 to 453.86) | 5.79 (0.68 to 58.63) | 0.72 (0.05 to 12.33) | 2.2 (0.29 to 19.93) | 1.32 (0.16 to 12.97) | 4.8 (0.35 to 74.13) | 0.6 (0.08 to 4.87) | Tremelimumab |

The league table show the relative effects of each intervention (the treatment on the column to the treatment of the row). The relative effects are measured as an odds ratio, with corresponding 95% credible intervals in parentheses. Bold indicates statistical significance.

### Table B.7.4. Estimated absolute event rate, pneumonitis.

| Intervention | Proportion per 10,000  (95% confidence interval) |
| --- | --- |
| Atezolizumab | 44 (18 to 112) |
| Atezolizumab plus Conventional Therapy | 50 (24 to 107) |
| Avelumab | 137 (32 to 706) |
| Avelumab plus Conventional Therapy | 112 (10 to 1,278) |
| Durvalumab | 75 (24 to 246) |
| Durvalumab plus Conventional Therapy | 61 (13 to 295) |
| Durvalumab plus Tremelimumab | 161 (51 to 562) |
| Ipilimumab | 187 (62 to 608) |
| Ipilimumab plus Conventional Therapy | 26 (7 to 87) |
| Nivolumab | 133 (65 to 314) |
| Nivolumab plus Conventional Therapy | 119 (1 to 6,743) |
| Nivolumab plus Ipilimumab | 368 (143 to 1,032) |
| Nivolumab plus Ipilimumab plus Conventional Therapy | 47 (7 to 309) |
| Pembrolizumab | 145 (74 to 295) |
| Pembrolizumab plus Conventional Therapy | 87 (41 to 193) |
| Pembrolizumab plus Ipilimumab | 310 (54 to 1,590) |
| Tremelimumab | 66 (7 to 464) |

### Table B.7.5. League table, myocarditis.

| Atezolizumab | 0.08 (0 to 14.48) | 0.94 (0 to 4532.66) | 0.02 (0 to 1) | 0.17 (0 to 113.47) | 0.07 (0 to 44.72) | 0 (0 to 1.61) | 0.01 (0 to 5.38) | 0.07 (0 to 56.07) | 0.07 (0 to 17.04) | 0.01 (0 to 1.45) | 0.03 (0 to 3.47) | 0.11 (0 to 72.64) | 0.01 (0 to 1.01) |
| --- | --- | --- | --- | --- | --- | --- | --- | --- | --- | --- | --- | --- | --- |
| 13.31 (0.07 to 16991.40) | Atezolizumab plus Conventional Therapy | 11.37 (0.07 to 27980.68) | 0.22 (0.01 to 1.91) | 2.46 (0.02 to 377.44) | 1 (0.01 to 192.26) | 0.01 (0 to 6.43) | 0.13 (0 to 18.33) | 1 (0.01 to 214.33) | 1.04 (0.02 to 41.37) | 0.19 (0.01 to 2.7) | 0.44 (0.01 to 9.09) | 1.48 (0.01 to 269.45) | 0.07 (0 to 2.6) |
| 1.06 (0.00 to 2985.66) | 0.09 (0.00 to 13.95) | Avelumab | 0.02 (0 to 0.97) | 0.2 (0 to 98.85) | 0.08 (0 to 42.64) | 0 (0 to 1.28) | 0.01 (0 to 5.09) | 0.08 (0 to 58.01) | 0.09 (0 to 12.66) | 0.01 (0 to 1.58) | 0.04 (0 to 3.91) | 0.12 (0 to 62.14) | 0.01 (0 to 1.14) |
| **63.39 (1.00 to 60238.08)** | 4.58 (0.52 to 110.27) | 52.38 (1.03 to 105566.77) | Conventional Therapy | 11.38 (0.4 to 1114.91) | 4.73 (0.08 to 542.91) | 0.07 (0 to 17.97) | 0.67 (0 to 53.79) | 4.85 (0.08 to 724.32) | 4.98 (0.37 to 100.22) | 0.9 (0.09 to 6.43) | 2.15 (0.3 to 17.38) | 7.43 (0.15 to 939.49) | 0.37 (0.01 to 6.98) |
| 5.81 (0.01 to 15106.60) | 0.41 (0.00 to 45.40) | 5.11 (0.01 to 15962.24) | 0.09 (0.00 to 2.48) | Durvalumab | 0.4 (0 to 149.69) | 0.01 (0 to 0.98) | 0.05 (0 to 16.63) | 0.4 (0 to 163.65) | 0.42 (0 to 42.61) | 0.07 (0 to 3.78) | 0.18 (0 to 10.3) | 0.61 (0 to 223.66) | 0.03 (0 to 3.19) |
| 14.56 (0.02 to 34341.93) | 1.00 (0.01 to 158.93) | 13.02 (0.02 to 47732.35) | 0.21 (0.00 to 12.43) | 2.50 (0.01 to 968.11) | Durvalumab plus Conventional Therapy | 0.01 (0 to 15.99) | 0.13 (0 to 51.75) | 1.04 (0 to 493.97) | 1.04 (0.01 to 151.06) | 0.18 (0 to 16.49) | 0.45 (0 to 36.34) | 1.6 (0 to 726.85) | 0.07 (0 to 10.91) |
| 1513.00 (0.62 to 36138287.58) | 83.8' (0.16 to 455250.68) | 1341.64 (0.78 to 71529202.75) | 15.03 (0.06 to 46180.66) | **184.92 (1.02 to 837464.07)** | 92.95 (0.06 to 797563.53) | Durvalumab plus Tremelimumab | 9.78 (0 to 154090.11) | 82.7 (0.06 to 983757.81) | 84.84 (0.13 to 384135.58) | 13.73 (0.03 to 52071.44) | 34.66 (0.09 to 132117.91) | 132.94 (0.1 to 1322779.52) | 5.91 (0.01 to 28661.51) |
| 123.79 (0.19 to 373009.90) | 7.81 (0.05 to 2430.67) | 105.09 (0.20 to 501754.13) | 1.50 (0.02 to 244.97) | 19.44 (0.06 to 15888.38) | 7.80 (0.02 to 8004.94) | 0.10 (0.00 to 211.04) | Nivolumab | 7.74 (0.02 to 9525.55) | 6.96 (0.26 to 856.64) | 1.3 (0.01 to 225.54) | 3.32 (0.03 to 683.04) | 12.57 (0.04 to 7018.27) | 0.58 (0 to 97.75) |
| 14.55 (0.02 to 38593.27) | 1.00 (0.00 to 178.01) | 12.83 (0.02 to 61280.80) | 0.21 (0.00 to 11.83) | 2.49 (0.01 to 971.82) | 0.96 (0.00 to 535.20) | 0.01 (0.00 to 15.41) | 0.13 (0.00 to 51.96) | Nivolumab plus Conventional Therapy | 1.03 (0 to 150.17) | 0.17 (0 to 18.36) | 0.43 (0 to 42.41) | 1.5 (0 to 866.15) | 0.07 (0 to 11.12) |
| 14.06 (0.06 to 19237.13) | 0.96 (0.02 to 48.6) | 11.11 (0.08 to 31835.59) | 0.20 (0.01 to 2.67) | 2.36 (0.02 to 396.61) | 0.96 (0.01 to 189.46) | 0.01 (0.00 to 7.65) | 0.14 (0.00 to 3.88) | 0.97 (0.01 to 238.53) | Nivolumab plus Ipilimumab | 0.18 (0.01 to 3.37) | 0.43 (0.01 to 10.36) | 1.5 (0.01 to 244.13) | 0.08 (0 to 1.4) |
| 76.09 (0.69 to 107696.53) | 5.36 (0.37 to 188.34) | 67.98 (0.63 to 170000.59) | 1.11 (0.16 to 11.13) | 13.5 (0.26 to 1908.13) | 5.61 (0.06 to 981.14) | 0.07 (0.00 to 34.98) | 0.77 (0.00 to 79.98) | 5.76 (0.05 to 1083.25) | 5.7 (0.30 to 185.16) | Pembrolizumab | 2.4 (0.19 to 43.85) | 8.29 (0.35 to 608.81) | 0.44 (0.02 to 5.37) |
| 29.74 (0.29 to 39611.14) | 2.27 (0.11 to 77.36) | 26.99 (0.26 to 59845.00) | 0.46 (0.06 to 3.33) | 5.60 (0.10 to 786.27) | 2.22 (0.03 to 390.57) | 0.03 (0.00 to 11.44) | 0.30 (0.00 to 38.13) | 2.30 (0.02 to 459.02) | 2.32 (0.10 to 85.79) | 0.42 (0.02 to 5.21) | Pembrolizumab plus Conventional Therapy | 3.62 (0.05 to 516.21) | 0.17 (0 to 5.06) |
| 9.06 (0.01 to 23327.67) | 0.67 (0.00 to 72.96) | 8.31 (0.02 to 31799.89) | 0.13 (0.00 to 6.77) | 1.63 (0.00 to 626.32) | 0.62 (0.00 to 284.9) | 0.01 (0.00 to 9.54) | 0.08 (0.00 to 23.98) | 0.67 (0.00 to 286.52) | 0.67 (0.00 to 70.03) | 0.12 (0.00 to 2.89) | 0.28 (0.00 to 20.16) | Pembrolizumab plus Ipilimumab | 0.05 (0 to 2.88) |
| 195.4 (0.99 to 365605.47) | 13.66 (0.39 to 946.08) | 176.27 (0.88 to 521075.49) | 2.68 (0.14 to 73.97) | 34.34 (0.31 to 7892.49) | 13.93 (0.09 to 3508.60) | 0.17 (0.00 to 130.43) | 1.73 (0.01 to 225.50) | 13.8 (0.09 to 4698.06) | 12.95 (0.72 to 518.92) | 2.26 (0.19 to 52.33) | 5.88 (0.20 to 227.71) | 20.56 (0.35 to 3578.22) | Placebo or No Intervention |

The league table show the relative effects of each intervention (the treatment on the column to the treatment of the row). The relative effects are measured as an odds ratio, with corresponding 95% credible intervals in parentheses. Bold indicates statistical significance.

### Table B.7.6. Anticipated absolute event rate, myocarditis.

| Intervention | Proportion per 10,000  (95% confidence interval) |
| --- | --- |
| Atezolizumab | 1,127 (20 to 9,917) |
| Atezolizumab plus Conventional Therapy | 90 (10 to 1,809) |
| Avelumab | 949 (20 to 9,952) |
| Durvalumab | 222 (8 to 6,908) |
| Durvalumab plus Conventional Therapy | 93 (1 to 5,210) |
| Durvalumab plus Tremelimumab | 1 (0 to 347) |
| Nivolumab | 13 (0 to 973) |
| Nivolumab plus Conventional Therapy | 96 (1 to 5,920) |
| Nivolumab plus Ipilimumab | 98 (7 to 1,672) |
| Pembrolizumab | 18 (1 to 127) |
| Pembrolizumab plus Conventional Therapy | 42 (6 to 336) |
| Pembrolizumab plus Ipilimumab | 146 (3 to 6,531) |

### Table B.7.8. League table, colitis.

| Atezolizumab | 2.15 (0.55 to 8.53) | 6.72 (0.67 to 133.36) | 1.75 (0.09 to 59.17) | 0.43 (0.14 to 1.28) | 1.07 (0.14 to 8.35) | 1.2 (0.1 to 20.48) | 6.12 (1.05 to 45.68) | 5 (1.24 to 18.41) | 7.3 (1.53 to 34.08) | 0.69 (0.17 to 2.56) | 8.38 (2 to 33.39) | 1.98 (0.21 to 27.62) | 1.22 (0.34 to 4.14) | 1.51 (0.39 to 5.72) | 8.32 (0.89 to 127.94) | 0.1 (0.02 to 0.54) | 12.55 (1.8 to 103.49) |
| --- | --- | --- | --- | --- | --- | --- | --- | --- | --- | --- | --- | --- | --- | --- | --- | --- | --- |
| 0.47 (0.12 to 1.82) | Atezolizumab plus Conventional Therapy | 3.03 (0.36 to 62.73) | 0.81 (0.04 to 27.01) | 0.21 (0.07 to 0.51) | 0.5 (0.07 to 3.84) | 0.55 (0.05 to 8.73) | 2.89 (0.49 to 20.32) | 2.32 (0.67 to 7.23) | 3.29 (0.85 to 14.08) | 0.32 (0.09 to 1.07) | 3.88 (1.07 to 13.27) | 0.91 (0.11 to 12.62) | 0.57 (0.18 to 1.63) | 0.71 (0.2 to 2.31) | 3.94 (0.45 to 52.6) | 0.05 (0.01 to 0.22) | 5.82 (0.86 to 46.19) |
| 0.15 (0.01 to 1.50) | 0.33 (0.02 to 2.75) | Avelumab | 0.27 (0.01 to 7.21) | 0.07 (0 to 0.46) | 0.16 (0.01 to 2.42) | 0.17 (0.01 to 5.19) | 0.95 (0.03 to 13.18) | 0.75 (0.04 to 6.09) | 1.08 (0.06 to 9.52) | 0.1 (0.01 to 0.86) | 1.23 (0.06 to 10.84) | 0.3 (0.01 to 6.26) | 0.19 (0.01 to 1.45) | 0.23 (0.01 to 1.88) | 1.28 (0.04 to 28.27) | 0.02 (0 to 0.14) | 1.93 (0.07 to 28.97) |
| 0.57 (0.02 to 11.02) | 1.24 (0.04 to 22.63) | 3.7 (0.14 to 147.06) | Avelumab plus Conventional Therapy | 0.25 (0.01 to 3.71) | 0.6 (0.01 to 17.14) | 0.68 (0.01 to 29.46) | 3.55 (0.08 to 97.45) | 2.81 (0.09 to 49.9) | 4.16 (0.12 to 75.16) | 0.39 (0.01 to 6.97) | 4.7 (0.15 to 84.29) | 1.14 (0.03 to 38.44) | 0.69 (0.02 to 11.5) | 0.87 (0.03 to 14.69) | 4.92 (0.1 to 169.64) | 0.06 (0 to 1.18) | 7.01 (0.17 to 218.3) |
| 2.31 (0.78 to 7.34) | **4.88 (1.97 to 14.18)** | **14.81 (2.18 to 273.33)** | 4.02 (0.27 to 118.02) | Conventional Therapy | 2.41 (0.5 to 15.31) | 2.73 (0.32 to 37.26) | 13.92 (3.59 to 83.06) | 11.38 (5.55 to 24.99) | 16.25 (6.81 to 48.73) | 1.56 (0.75 to 3.59) | 18.87 (8.81 to 47.34) | 4.49 (0.68 to 50.46) | 2.81 (1.59 to 5.08) | 3.47 (1.69 to 7.46) | 19.36 (2.83 to 228.46) | 0.24 (0.06 to 0.8) | 29.16 (5.95 to 183.61) |
| 0.94 (0.12 to 6.95) | 2.01 (0.26 to 14.57) | 6.16 (0.41 to 174.89) | 1.67 (0.06 to 76.02) | 0.42 (0.07 to 2.01) | Durvalumab | 1.13 (0.07 to 23.12) | 5.65 (1.95 to 20.92) | 4.64 (0.67 to 27.85) | 6.74 (0.85 to 46.44) | 0.65 (0.09 to 3.73) | 7.74 (1.07 to 50.69) | 1.84 (0.13 to 35.61) | 1.15 (0.17 to 6.36) | 1.41 (0.2 to 8.3) | 7.94 (0.56 to 141.46) | 0.1 (0.01 to 0.7) | 11.74 (2.97 to 51.25) |
| 0.84 (0.05 to 9.92) | 1.81 (0.11 to 18.92) | 5.78 (0.19 to 188.73) | 1.48 (0.03 to 75.86) | 0.37 (0.03 to 3.13) | 0.88 (0.04 to 15.29) | Durvalumab plus Conventional Therapy | 4.99 (0.3 to 88.86) | 4.19 (0.27 to 41.28) | 6.02 (0.38 to 68.34) | 0.57 (0.04 to 5.75) | 6.94 (0.46 to 73.01) | 1.65 (0.07 to 41.96) | 1.03 (0.07 to 9.54) | 1.27 (0.08 to 12.52) | 7.11 (0.28 to 178.36) | 0.09 (0 to 1.06) | 10.24 (0.55 to 192.48) |
| **0.16 (0.02 to 0.95)** | 0.35 (0.05 to 2.04) | 1.05 (0.08 to 29.61) | 0.28 (0.01 to 11.87) | **0.07 (0.01 to 0.28)** | **0.18 (0.05 to 0.51)** | 0.2 (0.01 to 3.34) | Durvalumab plus Tremelimumab | 0.82 (0.12 to 3.82) | 1.17 (0.16 to 6.85) | 0.11 (0.02 to 0.52) | 1.37 (0.2 to 6.92) | 0.32 (0.02 to 5.3) | 0.2 (0.03 to 0.89) | 0.24 (0.04 to 1.2) | 1.35 (0.1 to 22.21) | 0.02 (0 to 0.1) | 2.04 (0.73 to 5.58) |
| **0.2 (0.05 to 0.81)** | 0.43 (0.14 to 1.50) | 1.34 (0.16 to 24.47) | 0.36 (0.02 to 11.4) | **0.09 (0.04 to 0.18)** | 0.22 (0.04 to 1.49) | 0.24 (0.02 to 3.69) | 1.22 (0.26 to 8.3) | Ipilimumab | 1.44 (0.44 to 5.29) | 0.14 (0.07 to 0.26) | 1.66 (0.96 to 3.17) | 0.4 (0.05 to 4.78) | 0.25 (0.12 to 0.49) | 0.31 (0.11 to 0.82) | 1.69 (0.24 to 21.16) | 0.02 (0.01 to 0.06) | 2.53 (0.44 to 18.29) |
| **0.14 (0.03 to 0.65)** | 0.30 (0.07 to 1.18) | 0.92 (0.11 to 17.45) | 0.24 (0.01 to 8.01) | **0.06 (0.02 to 0.15)** | 0.15 (0.02 to 1.18) | 0.17 (0.01 to 2.62) | 0.85 (0.15 to 6.33) | 0.7 (0.19 to 2.25) | Ipilimumab plus Conventional Therapy | 0.1 (0.03 to 0.32) | 1.16 (0.3 to 4.07) | 0.27 (0.03 to 3.5) | 0.17 (0.05 to 0.49) | 0.21 (0.06 to 0.66) | 1.17 (0.13 to 16.6) | 0.01 (0 to 0.07) | 1.77 (0.25 to 13.9) |
| 1.45 (0.39 to 5.77) | 3.15 (0.93 to 10.9) | **9.91 (1.17 to 187.63)** | 2.59 (0.14 to 82.81) | 0.64 (0.28 to 1.34) | 1.54 (0.27 to 11.31) | 1.74 (0.17 to 26.08) | **8.79 (1.92 to 62.04)** | **7.26 (3.8 to 13.92)** | **10.37 (3.14 to 38.51)** | Nivolumab | 12.07 (6.26 to 25.82) | 2.91 (0.36 to 35.02) | 1.79 (0.76 to 3.98) | 2.22 (0.77 to 6.12) | 12.26 (1.62 to 155.73) | 0.15 (0.04 to 0.52) | 18.27 (3.19 to 136.65) |
| **0.12 (0.03 to 0.50)** | **0.26 (0.08 to 0.94)** | 0.81 (0.09 to 15.49) | 0.21 (0.01 to 6.89) | **0.05 (0.02 to 0.11)** | **0.13 (0.02 to 0.93)** | 0.14 (0.01 to 2.18) | 0.73 (0.14 to 4.9) | 0.6 (0.32 to 1.04) | 0.86 (0.25 to 3.28) | **0.08 (0.04 to 0.16)** | Nivolumab plus Ipilimumab | 0.24 (0.03 to 2.93) | 0.15 (0.06 to 0.33) | 0.18 (0.06 to 0.51) | 1.02 (0.13 to 12.8) | 0.01 (0 to 0.04) | 1.52 (0.25 to 11.06) |
| 0.51 (0.04 to 4.86) | 1.1 (0.08 to 9.39) | 3.38 (0.16 to 105.29) | 0.88 (0.03 to 39.33) | 0.22 (0.02 to 1.48) | 0.54 (0.03 to 7.49) | 0.61 (0.02 to 15.17) | 3.16 (0.19 to 40.13) | 2.52 (0.21 to 19.72) | 3.69 (0.29 to 32.13) | 0.34 (0.03 to 2.74) | 4.21 (0.34 to 34.55) | Nivolumab plus Ipilimumab plus Conventional Therapy | 0.62 (0.05 to 4.56) | 0.77 (0.06 to 6.05) | 4.3 (0.2 to 88.54) | 0.05 (0 to 0.5) | 6.46 (0.35 to 86.85) |
| 0.82 (0.24 to 2.97) | 1.76 (0.61 to 5.47) | 5.37 (0.69 to 100.18) | 1.44 (0.09 to 44.75) | **0.36 (0.2 to 0.63)** | 0.87 (0.16 to 5.87) | 0.97 (0.1 to 14.53) | **4.98 (1.12 to 32.4)** | **4.05 (2.03 to 8.51)** | **5.81 (2.02 to 19.86)** | 0.56 (0.25 to 1.31) | **6.71 (3.05 to 17.29)** | 1.6 (0.22 to 19.1) | Pembrolizumab | 1.25 (0.53 to 2.83) | 6.76 (1.12 to 76.53) | 0.09 (0.02 to 0.27) | 10.31 (1.88 to 70.45) |
| 0.66 (0.17 to 2.59) | 1.41 (0.43 to 5.02) | 4.35 (0.53 to 86.12) | 1.16 (0.07 to 37.47) | **0.29 (0.13 to 0.59)** | 0.71 (0.12 to 4.94) | 0.79 (0.08 to 12.21) | 4.09 (0.83 to 25.39) | **3.26 (1.23 to 8.95)** | **4.68 (1.5 to 17.59)** | 0.45 (0.16 to 1.29) | **5.45 (1.95 to 17.2)** | 1.3 (0.17 to 15.9) | 0.8 (0.35 to 1.87) | Pembrolizumab plus Conventional Therapy | 5.54 (0.75 to 71.89) | 0.07 (0.01 to 0.26) | 8.5 (1.41 to 58.99) |
| 0.12 (0.01 to 1.13) | 0.25 (0.02 to 2.23) | 0.78 (0.04 to 25.55) | 0.2 (0.01 to 9.82) | **0.05 (0 to 0.35)** | 0.13 (0.01 to 1.80) | 0.14 (0.01 to 3.54) | 0.74 (0.05 to 9.57) | 0.59 (0.05 to 4.23) | 0.85 (0.06 to 7.92) | **0.08 (0.01 to 0.62)** | 0.98 (0.08 to 7.68) | 0.23 (0.01 to 4.9) | **0.15 (0.01 to 0.89)** | 0.18 (0.01 to 1.33) | Pembrolizumab plus Ipilimumab | 0.01 (0 to 0.11) | 1.49 (0.09 to 20.44) |
| **9.66 (1.85 to 58.58)** | **20.4 (4.57 to 116.4)** | **64.29 (6.96 to 1475.64)** | 17.66 (0.84 to 638.39) | **4.1 (1.26 to 16.82)** | **10.24 (1.43 to 98.38)** | 11.68 (0.94 to 218.33) | **59.64 (9.93 to 523.73)** | **47.06 (16.36 to 187.7)** | **69.75 (15.22 to 398.99)** | **6.47 (1.92 to 28.03)** | **78.66 (24.55 to 349.65)** | **19.19 (2.00 to 294.02)** | **11.54 (3.74 to 47.35)** | **14.28 (3.8 to 69)** | **82.02 (8.9 to 1295.65)** | Placebo or No Intervention | 122.53 (18.58 to 1100.58) |
| **0.08 (0.01 to 0.56)** | 0.17 (0.02 to 1.16) | 0.52 (0.03 to 14.91) | 0.14 (0 to 5.94) | **0.03 (0.01 to 0.17)** | **0.09 (0.02 to 0.34)** | 0.10 (0.01 to 1.80) | 0.49 (0.18 to 1.37) | 0.4 (0.05 to 2.25) | 0.57 (0.07 to 3.97) | **0.05 (0.01 to 0.31)** | 0.66 (0.09 to 3.96) | 0.15 (0.01 to 2.84) | **0.1 (0.01 to 0.53)** | **0.12 (0.02 to 0.71)** | 0.67 (0.05 to 11.52) | **0.01 (0 to 0.05)** | Tremelimumab |

The league table show the relative effects of each intervention (the treatment on the column to the treatment of the row). The relative effects are measured as an odds ratio, with corresponding 95% credible intervals in parentheses. Bold indicates statistical significance.

### Table B.7.9. Anticipated absolute event rate, colitis.

| Intervention | Proportion per 10,000  (95% confidence interval) |
| --- | --- |
| Atezolizumab | 46 (15 to 144) |
| Atezolizumab plus Conventional Therapy | 96 (39 to 276) |
| Avelumab | 288 (43 to 3,539) |
| Avelumab plus Conventional Therapy | 79 (5 to 1,912) |
| Durvalumab | 48 (10 to 297) |
| Durvalumab plus Conventional Therapy | 54 (6 to 694) |
| Durvalumab plus Tremelimumab | 271 (71 to 1,427) |
| Ipilimumab | 222 (109 to 476) |
| Ipilimumab plus Conventional Therapy | 315 (134 to 889) |
| Nivolumab | 31 (15 to 71) |
| Nivolumab plus Ipilimumab | 364 (173 to 866) |
| Nivolumab plus Ipilimumab plus Conventional Therapy | 89 (13 to 918) |
| Pembrolizumab | 55 (31 to 100) |
| Pembrolizumab plus Conventional Therapy | 69 (33 to 147) |
| Pembrolizumab plus Ipilimumab | 373 (56 to 3,140) |
| Tremelimumab | 552 (117 to 2,689) |

### Table B.7.10. League table, nephritis.

| Atezolizumab | 1.28 (0.19 to 10.41) | 0.42 (0 to 204.58) | 0.02 (0 to 0.45) | 0.07 (0 to 4.37) | 0.04 (0 to 2.27) | 0.08 (0 to 3.39) | 0.02 (0 to 1.04) | 0.03 (0 to 1.26) | 0.04 (0 to 2.49) | 0.06 (0 to 22.09) | 0.07 (0 to 2.22) | 0.02 (0 to 0.64) | 0.28 (0 to 120.92) | 0.02 (0 to 1.37) | 0.63 (0 to 419.35) |
| --- | --- | --- | --- | --- | --- | --- | --- | --- | --- | --- | --- | --- | --- | --- | --- |
| 0.78 (0.10 to 5.18) | Atezolizumab plus Conventional Therapy | 0.33 (0 to 115.71) | 0.01 (0 to 0.24) | 0.06 (0 to 2.54) | 0.03 (0 to 1.27) | 0.06 (0 to 1.85) | 0.01 (0 to 0.54) | 0.02 (0 to 0.73) | 0.03 (0 to 1.4) | 0.05 (0 to 15.73) | 0.06 (0 to 1.27) | 0.01 (0 to 0.39) | 0.22 (0 to 89.13) | 0.01 (0 to 0.8) | 0.46 (0 to 270.25) |
| 2.39 (0.00 to 990.66) | 3.03 (0.01 to 1255.10) | Avelumab | 0.04 (0 to 1.36) | 0.15 (0 to 12.6) | 0.07 (0 to 7) | 0.17 (0 to 8.48) | 0.04 (0 to 2.99) | 0.07 (0 to 3.49) | 0.1 (0 to 6.15) | 0.15 (0 to 66.07) | 0.17 (0 to 6.55) | 0.04 (0 to 1.87) | 0.68 (0 to 276.89) | 0.04 (0 to 1.48) | 1.43 (0 to 810.61) |
| 54.87 (2.24 to 6101.96) | **68.47 (4.15 to 7323.23)** | 24.19 (0.74 to 3906.28) | Conventional Therapy | 3.82 (0.49 to 57.19) | 2 (0.18 to 28.28) | 4.37 (0.7 to 26.62) | 1.08 (0.11 to 10.68) | 1.72 (0.31 to 9.56) | 2.47 (0.3 to 19.9) | 3.65 (0.09 to 501.72) | 3.93 (1.21 to 14.73) | 1.08 (0.29 to 3.95) | 16.22 (0.27 to 3331.08) | 1.12 (0.04 to 14.41) | 35.51 (0.41 to 9224.82) |
| 14.17 (0.23 to 2543.67) | 17.99 (0.39 to 3351.62) | 6.79 (0.08 to 1218.26) | 0.26 (0.02 to 2.05) | Durvalumab | 0.51 (0.06 to 3.06) | 1.09 (0.05 to 19.58) | 0.27 (0.01 to 6.51) | 0.43 (0.02 to 7.34) | 0.63 (0.02 to 13.36) | 0.94 (0.01 to 178.81) | 1.03 (0.06 to 13.55) | 0.27 (0.01 to 3.41) | 4.1 (0.03 to 1337.76) | 0.27 (0 to 8.2) | 8.5 (0.16 to 1539.8) |
| 27.63 (0.44 to 5279.87) | 35.25 (0.79 to 6264.29) | 13.81 (0.14 to 2545.70) | 0.50 (0.04 to 5.55) | 1.97 (0.33 to 15.56) | Durvalumab plus Tremelimumab | 2.11 (0.1 to 46.1) | 0.53 (0.02 to 16.25) | 0.82 (0.04 to 17.63) | 1.2 (0.04 to 30.59) | 1.92 (0.02 to 393.3) | 1.94 (0.11 to 32.31) | 0.52 (0.03 to 8.06) | 8.14 (0.06 to 2508.34) | 0.53 (0.01 to 19.38) | 16.9 (0.34 to 3204.7) |
| 13.30 (0.30 to 1773.25) | 16.18 (0.54 to 2378.70) | 5.82 (0.12 to 1069.05) | 0.23 (0.04 to 1.42) | 0.92 (0.05 to 21.04) | 0.47 (0.02 to 10.33) | Ipilimumab | 0.24 (0.01 to 4.55) | 0.42 (0.05 to 2.37) | 0.58 (0.05 to 5.05) | 0.86 (0.01 to 141.95) | 0.9 (0.17 to 5.41) | 0.25 (0.03 to 2.09) | 3.86 (0.05 to 830.5) | 0.27 (0.01 to 2.96) | 8.26 (0.06 to 2970.72) |
| 52.43 (0.97 to 8347.66) | **68.83 (1.84 to 9989.27)** | 22.64 (0.33 to 5376.88) | 0.93 (0.09 to 8.70) | 3.66 (0.15 to 115.67) | 1.90 (0.06 to 61.01) | 4.11 (0.22 to 67.23) | Ipilimumab plus Conventional Therapy | 1.59 (0.1 to 28.12) | 2.26 (0.11 to 48.51) | 3.72 (0.04 to 616.44) | 3.74 (0.28 to 49.45) | 0.99 (0.07 to 12.99) | 15.17 (0.16 to 4466.51) | 0.99 (0.02 to 28.89) | 34.05 (0.23 to 12491.48) |
| 32.20 (0.79 to 4637.58) | **41.43 (1.37 to 5964.64)** | 15.32 (0.29 to 2977.93) | 0.58 (0.10 to 3.25) | 2.35 (0.14 to 46.38) | 1.22 (0.06 to 25.40) | 2.41 (0.42 to 19.11) | 0.63 (0.04 to 10.36) | Nivolumab | 1.41 (0.16 to 13.08) | 2.13 (0.04 to 389.38) | 2.35 (0.33 to 17.03) | 0.59 (0.08 to 5.64) | 9.53 (0.13 to 2190.76) | 0.64 (0.02 to 11.07) | 20.9 (0.16 to 6963.41) |
| 23.99 (0.40 to 3643.84) | 30.20 (0.71 to 4753.35) | 10.20 (0.16 to 2088.82) | 0.41 (0.05 to 3.35) | 1.59 (0.07 to 42.34) | 0.83 (0.03 to 22.45) | 1.72 (0.20 to 18.69) | 0.44 (0.02 to 9.45) | 0.71 (0.08 to 6.23) | Nivolumab plus Ipilimumab | 1.54 (0.02 to 274.59) | 1.6 (0.17 to 16.99) | 0.43 (0.04 to 5.17) | 6.79 (0.07 to 2103.45) | 0.44 (0.01 to 10.01) | 15.72 (0.1 to 4537.09) |
| 16.12 (0.05 to 5054.17) | 20.18 (0.06 to 6542.29) | 6.72 (0.02 to 3018.31) | 0.27 (0.00 to 11.20) | 1.06 (0.01 to 93.54) | 0.52 (0.00 to 46.9) | 1.16 (0.01 to 70.87) | 0.27 (0.00 to 23.74) | 0.47 (0.00 to 26.55) | 0.65 (0 to 43.35) | Nivolumab plus Ipilimumab plus Conventional Therapy | 1.08 (0.01 to 55.73) | 0.29 (0 to 16.05) | 4.11 (0.01 to 2742.81) | 0.27 (0 to 27.16) | 8.76 (0.02 to 5455.73) |
| 14.42 (0.45 to 1752.45) | 17.77 (0.79 to 2167.83) | 6.04 (0.15 to 970.16) | **0.25 (0.07 to 0.83)** | 0.97 (0.07 to 17.22) | 0.52 (0.03 to 9.19) | 1.11 (0.18 to 5.91) | 0.27 (0.02 to 3.52) | 0.43 (0.06 to 3.01) | 0.63 (0.06 to 5.88) | 0.93 (0.02 to 141.83) | Pembrolizumab | 0.27 (0.06 to 1.26) | 3.97 (0.08 to 728.7) | 0.29 (0.01 to 2.98) | 8.95 (0.09 to 2852.57) |
| **53.20 (1.55 to 5935.34)** | **67.14 (2.54 to 7548.77)** | 23.32 (0.53 to 4167.99) | 0.93 (0.25 to 3.45) | 3.66 (0.29 to 72.00) | 1.91 (0.12 to 34.78) | 4.07 (0.48 to 33.27) | 1.01 (0.08 to 13.81) | 1.68 (0.18 to 12.87) | 2.31 (0.19 to 27.1) | 3.50 (0.06 to 549.34) | 3.73 (0.79 to 18.11) | Pembrolizumab plus Conventional Therapy | 15.29 (0.24 to 3198.81) | 1.07 (0.03 to 16.74) | 33.54 (0.34 to 9191.12) |
| 3.58 (0.01 to 1197.06) | 4.54 (0.01 to 1395.11) | 1.46 (0.00 to 921.4) | 0.06 (0.00 to 3.66) | 0.24 (0.00 to 32.67) | 0.12 (0.00 to 15.64) | 0.26 (0.00 to 19.20) | 0.07 (0.00 to 6.30) | 0.10 (0.00 to 7.79) | 0.15 (0 to 13.7) | 0.24 (0.00 to 143.43) | 0.25 (0.00 to 12.7) | 0.07 (0.00 to 4.18) | Pembrolizumab plus Ipilimumab | 0.06 (0 to 6.52) | 2.07 (0 to 1848.94) |
| 59.19 (0.73 to 12747.91) | **75.68 (1.25 to 14943.04)** | 23.11 (0.67 to 5147.35) | 0.89 (0.07 to 27.65) | 3.66 (0.12 to 247.36) | 1.90 (0.05 to 134.40) | 3.70 (0.34 to 130.66) | 1.01 (0.03 to 57.18) | 1.57 (0.09 to 58.85) | 2.27 (0.1 to 109.25) | 3.68 (0.04 to 1235.81) | 3.40 (0.34 to 111.61) | 0.93 (0.06 to 34.92) | 16.22 (0.15 to 5780.56) | Placebo or No Intervention | 35.65 (0.19 to 17721.4) |
| 1.58 (0.00 to 969.20) | 2.16 (0.00 to 1311.44) | 0.70 (0.00 to 401.54) | 0.03 (0.00 to 2.46) | 0.12 (0.00 to 6.28) | 0.06 (0.00 to 2.97) | 0.12 (0.00 to 16.82) | 0.03 (0.00 to 4.42) | 0.05 (0.00 to 6.21) | 0.06 (0 to 10.45) | 0.11 (0.00 to 66.17) | 0.11 (0.00 to 11.62) | 0.03 (0.00 to 2.90) | 0.48 (0.00 to 334.49) | 0.03 (0.00 to 5.26) | Tremelimumab |

The league table show the relative effects of each intervention (the treatment on the column to the treatment of the row). The relative effects are measured as an odds ratio, with corresponding 95% credible intervals in parentheses. Bold indicates statistical significance.

### Table B.7.11. Estimated absolute event rate, nephritis.

| Intervention | Proportion per 10,000  (95% confidence interval) |
| --- | --- |
| Atezolizumab | 267 (11 to 7,532) |
| Atezolizumab plus Conventional Therapy | 331 (20 to 7,855) |
| Avelumab | 119 (3 to 6,614) |
| Durvalumab | 19 (2 to 278) |
| Durvalumab plus Tremelimumab | 9 (0 to 139) |
| Ipilimumab | 21 (3 to 131) |
| Ipilimumab plus Conventional Therapy | 5 (0 to 53) |
| Nivolumab | 8 (1 to 47) |
| Nivolumab plus Ipilimumab | 12 (1 to 98) |
| Nivolumab plus Ipilimumab plus Conventional Therapy | 18 (0 to 2,006) |
| Pembrolizumab | 19 (6 to 73) |
| Pembrolizumab plus Conventional Therapy | 5 (1 to 19) |
| Pembrolizumab plus Ipilimumab | 80 (1 to 6,249) |
| Tremelimumab | 174 (2 to 8,218) |

### Table B.7.12. League table, pancreatitis.

| Atezolizumab | 0.55 (0.15 to 1.94) | 12.51 (0.47 to 1868.63) | 0.34 (0 to 314.77) | 0.58 (0.2 to 1.7) | 0.63 (0.05 to 8.31) | 1.63 (0.23 to 18.65) | 0.75 (0.12 to 4.45) | 1 (0.12 to 10.06) | 0.68 (0.1 to 4.73) | 1.08 (0.22 to 5.75) | 7.18 (0.33 to 567.99) | 0.84 (0.18 to 3.89) | 0.41 (0.05 to 2.68) | 0.21 (0.01 to 2.57) | 0.58 (0.01 to 17.56) |
| --- | --- | --- | --- | --- | --- | --- | --- | --- | --- | --- | --- | --- | --- | --- | --- |
| 1.82 (0.52 to 6.61) | Atezolizumab plus Conventional Therapy | 21.94 (0.83 to 3703.27) | 0.62 (0 to 595.74) | 1.06 (0.37 to 3.12) | 1.14 (0.09 to 15.14) | 3.01 (0.41 to 31.37) | 1.33 (0.21 to 8.21) | 1.82 (0.21 to 19.72) | 1.24 (0.18 to 8.84) | 1.95 (0.41 to 10.28) | 13.19 (0.6 to 967.08) | 1.53 (0.33 to 6.88) | 0.74 (0.1 to 4.93) | 0.37 (0.02 to 4.6) | 1.07 (0.02 to 31.41) |
| 0.08 (0.00 to 2.13) | 0.05 (0.00 to 1.21) | Avelumab | 0.03 (0 to 3.04) | 0.05 (0 to 1.04) | 0.05 (0 to 3.02) | 0.14 (0 to 6.01) | 0.06 (0 to 1.94) | 0.08 (0 to 3.31) | 0.05 (0 to 1.9) | 0.09 (0 to 2.57) | 0.57 (0 to 112.01) | 0.07 (0 to 1.82) | 0.03 (0 to 1.05) | 0.01 (0 to 0.59) | 0.04 (0 to 4.3) |
| 2.94 (0.00 to 15212.55) | 1.61 (0.00 to 7669.65) | 37.83 (0.33 to 133514.90) | Avelumab plus Conventional Therapy | 1.69 (0 to 8693.52) | 1.9 (0 to 14434.28) | 5.22 (0 to 38720.29) | 2.09 (0 to 12749.88) | 3.13 (0 to 18787.73) | 1.95 (0 to 11260.84) | 3.07 (0 to 16542.79) | 24.86 (0.01 to 297322.59) | 2.49 (0 to 13837.69) | 1.23 (0 to 6951.41) | 0.59 (0 to 2995.65) | 1.76 (0 to 13299.62) |
| 1.73 (0.59 to 5.03) | 0.95 (0.32 to 2.73) | 20.85 (0.97 to 3114.55) | 0.59 (0.00 to 527.32) | Conventional Therapy | 1.1 (0.11 to 11.14) | 2.85 (0.54 to 24.71) | 1.28 (0.28 to 5.46) | 1.72 (0.27 to 13.79) | 1.16 (0.24 to 5.96) | 1.84 (0.59 to 6.56) | 12.16 (0.7 to 840.1) | 1.42 (0.47 to 4.85) | 0.72 (0.13 to 3.17) | 0.36 (0.02 to 3.41) | 1.04 (0.02 to 25.11) |
| 1.59 (0.12 to 18.99) | 0.87 (0.07 to 11.18) | 20.53 (0.33 to 4572.08) | 0.53 (0.00 to 711.55) | 0.91 (0.09 to 8.79) | Durvalumab | 2.61 (0.48 to 20.75) | 1.2 (0.07 to 15.89) | 1.6 (0.08 to 34.78) | 1.09 (0.06 to 16.94) | 1.69 (0.12 to 21.5) | 11.56 (0.29 to 1229.47) | 1.29 (0.11 to 17.21) | 0.64 (0.04 to 9.74) | 0.32 (0.01 to 7.36) | 0.93 (0.02 to 33.24) |
| 0.61 (0.05 to 4.26) | 0.33 (0.03 to 2.44) | 7.28 (0.17 to 1232.65) | 0.19 (0.00 to 210.99) | 0.35 (0.04 to 1.84) | 0.38 (0.05 to 2.10) | Durvalumab plus Tremelimumab | 0.44 (0.03 to 4.33) | 0.59 (0.04 to 8.16) | 0.41 (0.03 to 4.24) | 0.64 (0.06 to 5.23) | 4.29 (0.12 to 338.54) | 0.49 (0.05 to 4) | 0.24 (0.02 to 2.45) | 0.12 (0 to 1.92) | 0.35 (0.01 to 8.85) |
| 1.34 (0.22 to 8.61) | 0.75 (0.12 to 4.68) | 16.94 (0.52 to 2772.69) | 0.48 (0.00 to 465.58) | 0.78 (0.18 to 3.52) | 0.84 (0.06 to 15.17) | 2.27 (0.23 to 29.25) | Ipilimumab | 1.35 (0.12 to 17.7) | 0.94 (0.19 to 4.35) | 1.46 (0.35 to 6.85) | 9.96 (0.38 to 776.21) | 1.13 (0.21 to 6.28) | 0.55 (0.06 to 4.41) | 0.29 (0.01 to 3.39) | 0.78 (0.02 to 26.98) |
| 1.00 (0.10 to 8.33) | 0.55 (0.05 to 4.67) | 12.44 (0.30 to 2165.01) | 0.32 (0.00 to 346.30) | 0.58 (0.07 to 3.70) | 0.63 (0.03 to 12.22) | 1.68 (0.12 to 26.40) | 0.74 (0.06 to 8.07) | Ipilimumab plus Conventional Therapy | 0.68 (0.05 to 7.84) | 1.08 (0.1 to 9.89) | 7.25 (0.2 to 675.18) | 0.83 (0.08 to 7.27) | 0.4 (0.03 to 4.52) | 0.2 (0.01 to 3.74) | 0.57 (0.01 to 24.68) |
| 1.47 (0.21 to 9.85) | 0.81 (0.11 to 5.44) | 18.66 (0.53 to 2677.12) | 0.51 (0.00 to 516.42) | 0.86 (0.17 to 4.13) | 0.92 (0.06 to 16.25) | 2.46 (0.24 to 33.92) | 1.06 (0.23 to 5.34) | 1.48 (0.13 to 19.90) | Nivolumab | 1.57 (0.31 to 8.7) | 10.51 (0.36 to 833.61) | 1.23 (0.19 to 8.05) | 0.6 (0.06 to 5.63) | 0.31 (0.01 to 4.11) | 0.85 (0.01 to 31.24) |
| 0.93 (0.17 to 4.63) | 0.51 (0.10 to 2.47) | 11.42 (0.39 to 1729.22) | 0.33 (0.00 to 311.76) | 0.54 (0.15 to 1.70) | 0.59 (0.05 to 8.31) | 1.56 (0.19 to 17.51) | 0.69 (0.15 to 2.90) | 0.93 (0.10 to 10.09) | 0.64 (0.11 to 3.19) | Nivolumab plus Ipilimumab | 6.61 (0.29 to 491.36) | 0.77 (0.16 to 3.74) | 0.38 (0.05 to 2.51) | 0.19 (0.01 to 2.19) | 0.56 (0.01 to 17.16) |
| 0.14 (0.00 to 3.03) | 0.08 (0.00 to 1.66) | 1.75 (0.01 to 443.21) | 0.04 (0.00 to 77.10) | 0.08 (0.00 to 1.42) | 0.09 (0.00 to 3.46) | 0.23 (0.00 to 8.03) | 0.10 (0.00 to 2.62) | 0.14 (0.00 to 5.07) | 0.10 (0.00 to 2.75) | 0.15 (0.00 to 3.49) | Nivolumab plus Ipilimumab plus Conventional Therapy | 0.11 (0 to 2.75) | 0.06 (0 to 1.45) | 0.03 (0 to 1.27) | 0.07 (0 to 6.07) |
| 1.19 (0.26 to 5.61) | 0.66 (0.15 to 3.04) | 15.08 (0.55 to 2273.98) | 0.40 (0.00 to 390.77) | 0.70 (0.21 to 2.14) | 0.77 (0.06 to 9.32) | 2.05 (0.25 to 19.29) | 0.89 (0.16 to 4.66) | 1.21 (0.14 to 12.31) | 0.81 (0.12 to 5.39) | 1.29 (0.27 to 6.39) | 8.73 (0.36 to 638.82) | Pembrolizumab | 0.5 (0.07 to 2.65) | 0.25 (0.01 to 2.35) | 0.71 (0.02 to 20.15) |
| 2.44 (0.37 to 18.92) | 1.35 (0.20 to 9.81) | 30.22 (0.95 to 6732.8) | 0.81 (0.00 to 1028.49) | 1.39 (0.32 to 7.60) | 1.56 (0.10 to 26.11) | 4.12 (0.41 to 59.81) | 1.81 (0.23 to 16.33) | 2.49 (0.22 to 34.44) | 1.65 (0.18 to 17.11) | 2.62 (0.40 to 21.27) | 17.91 (0.69 to 1629.37) | 1.99 (0.38 to 13.88) | Pembrolizumab plus Conventional Therapy | 0.51 (0.02 to 7.82) | 1.46 (0.03 to 54.13) |
| 4.86 (0.39 to 106.76) | 2.69 (0.22 to 56.09) | **67.63 (1.71 to 12527.61)** | 1.70 (0.00 to 2232.42) | 2.77 (0.29 to 54.81) | 3.17 (0.14 to 119.76) | 8.56 (0.52 to 243.61) | 3.49 (0.30 to 76.66) | 4.94 (0.27 to 184.12) | 3.28 (0.24 to 84.90) | 5.29 (0.46 to 120.47) | 37.05 (0.79 to 6401.00) | 3.98 (0.43 to 79.63) | 1.95 (0.13 to 51.24) | Placebo or No Intervention | 2.67 (0.17 to 82.46) |
| 1.72 (0.06 to 89.47) | 0.94 (0.03 to 47.80) | 24.82 (0.23 to 6738.58) | 0.57 (0.00 to 1003.32) | 0.96 (0.04 to 43.81) | 1.07 (0.03 to 63.06) | 2.82 (0.11 to 141.83) | 1.28 (0.04 to 59.31) | 1.76 (0.04 to 121.12) | 1.17 (0.03 to 67.51) | 1.80 (0.06 to 95.05) | 13.87 (0.16 to 3240.93) | 1.41 (0.05 to 61.88) | 0.68 (0.02 to 35.13) | 0.37 (0.01 to 6.05) | Tremelimumab |

The league table show the relative effects of each intervention (the treatment on the column to the treatment of the row). The relative effects are measured as an odds ratio, with corresponding 95% credible intervals in parentheses. Bold indicates statistical significance.

### Table B.7.13. Estimated absolute event rate, pancreatitis.

| Intervention | Proportion per 10,000  (95% confidence interval) |
| --- | --- |
| Atezolizumab | 17 (5 to 50) |
| Atezolizumab plus Conventional Therapy | 9 (3 to 27) |
| Avelumab | 204 (9 to 7,571) |
| Avelumab plus Conventional Therapy | 5 (0 to 3,454) |
| Durvalumab | 10 (1 to 110) |
| Durvalumab plus Tremelimumab | 28 (5 to 241) |
| Ipilimumab | 12 (2 to 54) |
| Ipilimumab plus Conventional Therapy | 17 (2 to 136) |
| Nivolumab | 11 (2 to 59) |
| Nivolumab plus Ipilimumab | 18 (5 to 65) |
| Nivolumab plus Ipilimumab plus Conventional Therapy | 120 (7 to 4,567) |
| Pembrolizumab | 14 (4 to 48) |
| Pembrolizumab plus Conventional Therapy | 7 (1 to 31) |
| Tremelimumab | 10 (0 to 245) |

### Table B.7.14. League table, hepatitis.

| Atezolizumab | 1.35 (0.52 to 3.62) | 7.34 (0.62 to 247.03) | 2.17 (0.08 to 100.87) | 1.08 (0.46 to 2.60) | 2.69 (0.35 to 29.72) | 4.8 (0.08 to 808.63) | 5.93 (0.92 to 59.87) | 6.91 (1.5 to 34.05) | 8.23 (1.59 to 70.78) | 2.84 (0.59 to 14.84) | 18.22 (3.79 to 102.58) | 13.29 (0.74 to 1066.43) | 5.99 (1.73 to 21.92) | 5.23 (1.29 to 23.14) | 9.84 (0.67 to 176.19) | 0.43 (0.03 to 3.05) | 2.53 (0.11 to 37.56) |
| --- | --- | --- | --- | --- | --- | --- | --- | --- | --- | --- | --- | --- | --- | --- | --- | --- | --- |
| 0.74 (0.28 to 1.93) | Atezolizumab plus Conventional Therapy | 5.44 (0.49 to 168.12) | 1.61 (0.06 to 70.48) | 0.80 (0.37 to 1.65) | 1.97 (0.27 to 21.5) | 3.40 (0.06 to 646.60) | 4.43 (0.68 to 42.44) | 5.07 (1.21 to 22.63) | 6.12 (1.21 to 51.14) | 2.1 (0.47 to 9.92) | 13.53 (2.97 to 69.28) | 9.91 (0.53 to 713.65) | 4.46 (1.43 to 14.53) | 3.89 (1.02 to 15.42) | 7.29 (0.52 to 123.29) | 0.33 (0.02 to 2.13) | 1.85 (0.08 to 26.91) |
| 0.14 (0.00 to 1.62) | 0.18 (0.01 to 2.05) | Avelumab | 0.30 (0.01 to 12.08) | 0.15 (0.00 to 1.50) | 0.37 (0.01 to 8.76) | 0.66 (0.00 to 174.73) | 0.83 (0.02 to 17.54) | 0.93 (0.03 to 12.21) | 1.16 (0.03 to 23.3) | 0.39 (0.01 to 5.4) | 2.52 (0.07 to 36.15) | 1.91 (0.02 to 224.2) | 0.83 (0.03 to 9.39) | 0.72 (0.02 to 9.37) | 1.31 (0.03 to 44.28) | 0.05 (0 to 0.89) | 0.32 (0 to 11.85) |
| 0.46 (0.01 to 12.11) | 0.62 (0.01 to 17.83) | 3.28 (0.08 to 196.99) | Avelumab plus Conventional Therapy | 0.50 (0.01 to 13.27) | 1.27 (0.02 to 70.5) | 2.20 (0.01 to 805.98) | 2.86 (0.04 to 153.05) | 3.21 (0.07 to 100.81) | 4.02 (0.07 to 180.04) | 1.31 (0.03 to 40.69) | 8.46 (0.17 to 286.47) | 6.25 (0.05 to 1294.8) | 2.75 (0.07 to 77.01) | 2.52 (0.05 to 74.66) | 4.46 (0.06 to 315.46) | 0.18 (0 to 8.32) | 1.13 (0.01 to 80.68) |
| 0.93 (0.39 to 2.17) | 1.25 (0.61 to 2.68) | 6.77 (0.67 to 204.91) | 2.01 (0.08 to 83.27) | Conventional Therapy | 2.46 (0.39 to 23.4) | 4.31 (0.08 to 782.05) | 5.43 (1.02 to 47.35) | 6.31 (1.75 to 24.08) | 7.67 (1.84 to 55.16) | 2.63 (0.7 to 11.12) | 16.77 (4.51 to 75.58) | 12.11 (0.78 to 901.03) | 5.54 (2.27 to 15.06) | 4.83 (1.62 to 15.84) | 9.09 (0.73 to 145.94) | 0.41 (0.03 to 2.3) | 2.34 (0.12 to 31.32) |
| 0.37 (0.03 to 2.83) | 0.51 (0.05 to 3.73) | 2.68 (0.11 to 123.94) | 0.79 (0.01 to 55.28) | 0.41 (0.04 to 2.54) | Durvalumab | 1.68 (0.02 to 456.73) | 2.18 (0.63 to 8.93) | 2.53 (0.21 to 24.56) | 3.15 (0.23 to 43.41) | 1.02 (0.09 to 10.2) | 6.6 (0.59 to 75.47) | 4.94 (0.14 to 546.57) | 2.24 (0.2 to 17.34) | 1.94 (0.17 to 16.87) | 3.61 (0.12 to 103.32) | 0.15 (0.01 to 2.07) | 0.92 (0.05 to 8.98) |
| 0.21 (0.00 to 12.44) | 0.29 (0.00 to 16.62) | 1.52 (0.01 to 338.14) | 0.46 (0.00 to 108.82) | 0.23 (0.00 to 12.75) | 0.59 (0.00 to 49.98) | Durvalumab plus Conventional Therapy | 1.33 (0.01 to 105.92) | 1.42 (0.01 to 108.1) | 1.84 (0.01 to 135.54) | 0.58 (0 to 43.19) | 3.78 (0.02 to 308.16) | 2.98 (0.01 to 989.67) | 1.3 (0.01 to 83.21) | 1.13 (0.01 to 68.16) | 1.96 (0.01 to 303.69) | 0.08 (0 to 8.4) | 0.45 (0 to 65.1) |
| 0.17 (0.02 to 1.08) | 0.23 (0.02 to 1.46) | 1.20 (0.06 to 57.63) | 0.35 (0.01 to 25.43) | **0.18 (0.02 to 0.98)** | 0.46 (0.11 to 1.59) | 0.75 (0.01 to 199.25) | Durvalumab plus Tremelimumab | 1.15 (0.1 to 9.8) | 1.4 (0.11 to 17.04) | 0.47 (0.04 to 4.11) | 3.03 (0.28 to 29.87) | 2.27 (0.07 to 219.35) | 1.01 (0.11 to 7.07) | 0.88 (0.09 to 6.76) | 1.63 (0.06 to 43.77) | 0.07 (0 to 0.82) | 0.42 (0.02 to 3.33) |
| **0.14 (0.03 to 0.67)** | **0.20 (0.04 to 0.83)** | 1.08 (0.08 to 36.34) | 0.31 (0.01 to 14.71) | **0.16 (0.04 to 0.57)** | 0.40 (0.04 to 4.76) | 0.7 (0.01 to 144.21) | 0.87 (0.10 to 9.57) | Ipilimumab | 1.24 (0.16 to 11.47) | 0.41 (0.14 to 1.25) | 2.67 (0.92 to 8.56) | 1.94 (0.09 to 171.65) | 0.87 (0.25 to 3.15) | 0.77 (0.14 to 3.92) | 1.42 (0.1 to 24.31) | 0.06 (0 to 0.39) | 0.36 (0.01 to 6.4) |
| **0.12 (0.01 to 0.63)** | **0.16 (0.02 to 0.83)** | 0.86 (0.04 to 33.65) | 0.25 (0.01 to 13.92) | **0.13 (0.02 to 0.54)** | 0.32 (0.02 to 4.43) | 0.54 (0.01 to 105.7) | 0.71 (0.06 to 9.09) | 0.81 (0.09 to 6.08) | Ipilimumab plus Conventional Therapy | 0.33 (0.03 to 2.72) | 2.17 (0.21 to 18.24) | 1.61 (0.05 to 147.61) | 0.7 (0.09 to 4.09) | 0.62 (0.07 to 4.03) | 1.14 (0.05 to 25.54) | 0.05 (0 to 0.55) | 0.28 (0.01 to 4.97) |
| 0.35 (0.07 to 1.69) | 0.48 (0.10 to 2.12) | 2.59 (0.19 to 87.5) | 0.76 (0.02 to 35.64) | 0.38 (0.09 to 1.44) | 0.98 (0.10 to 11.11) | 1.73 (0.02 to 315.14) | 2.11 (0.24 to 22.94) | 2.42 (0.80 to 7.02) | 3.02 (0.37 to 29.01) | Nivolumab | 6.38 (2.37 to 19.7) | 4.71 (0.2 to 446.51) | 2.13 (0.52 to 8.93) | 1.87 (0.32 to 9.9) | 3.38 (0.22 to 64.11) | 0.16 (0.01 to 1.03) | 0.84 (0.03 to 15.66) |
| **0.05 (0.01 to 0.26)** | **0.07 (0.01 to 0.34)** | 0.40 (0.03 to 14.11) | 0.12 (0.00 to 5.85) | **0.06 (0.01 to 0.22)** | 0.15 (0.01 to 1.71) | 0.26 (0.00 to 54.37) | 0.33 (0.03 to 3.63) | 0.37 (0.12 to 1.08) | 0.46 (0.05 to 4.69) | **0.16 (0.05 to 0.42)** | Nivolumab plus Ipilimumab | 0.74 (0.03 to 60.54) | 0.33 (0.08 to 1.36) | 0.29 (0.05 to 1.52) | 0.54 (0.03 to 9.29) | 0.02 (0 to 0.14) | 0.13 (0.01 to 2.24) |
| 0.08 (0 to 1.35) | 0.10 (0.00 to 1.87) | 0.52 (0.00 to 42.33) | 0.16 (0.00 to 18.48) | 0.08 (0.00 to 1.28) | 0.20 (0.00 to 7.22) | 0.34 (0.00 to 111.11) | 0.44 (0.00 to 14.64) | 0.52 (0.01 to 10.86) | 0.62 (0.01 to 22.09) | 0.21 (0.00 to 5.07) | 1.35 (0.02 to 31.18) | Nivolumab plus Ipilimumab plus Conventional Therapy | 0.46 (0.01 to 7.96) | 0.39 (0 to 8) | 0.71 (0.01 to 34.13) | 0.03 (0 to 1.01) | 0.17 (0 to 8.66) |
| **0.17 (0.05 to 0.58)** | **0.22 (0.07 to 0.70)** | 1.20 (0.11 to 37.42) | 0.36 (0.01 to 14.67) | **0.18 (0.07 to 0.44)** | 0.45 (0.06 to 4.89) | 0.77 (0.01 to 146.30) | 0.99 (0.14 to 9.42) | 1.15 (0.32 to 3.92) | 1.42 (0.24 to 11.25) | 0.47 (0.11 to 1.93) | 3.05 (0.73 to 13.17) | 2.17 (0.13 to 164.97) | Pembrolizumab | 0.87 (0.25 to 3.08) | 1.62 (0.16 to 21.15) | 0.07 (0.01 to 0.39) | 0.41 (0.02 to 6.24) |
| **0.19 (0.04 to 0.78)** | **0.26 (0.06 to 0.98)** | 1.38 (0.11 to 48.11) | 0.4 (0.01 to 21.15) | **0.21 (0.06 to 0.62)** | 0.52 (0.06 to 5.94) | 0.89 (0.01 to 193.53) | 1.14 (0.15 to 11.36) | 1.30 (0.26 to 7.03) | 1.63 (0.25 to 14.05) | 0.54 (0.10 to 3.13) | 3.44 (0.66 to 21.19) | 2.55 (0.12 to 211.00) | 1.15 (0.32 to 4.08) | Pembrolizumab plus Conventional Therapy | 1.85 (0.13 to 33.63) | 0.08 (0.01 to 0.61) | 0.48 (0.02 to 7.68) |
| 0.10 (0.01 to 1.49) | 0.14 (0.01 to 1.92) | 0.76 (0.02 to 37.04) | 0.22 (0.00 to 17.61) | 0.11 (0.01 to 1.38) | 0.28 (0.01 to 8.50) | 0.51 (0.00 to 132.45) | 0.61 (0.02 to 17.90) | 0.71 (0.04 to 9.64) | 0.88 (0.04 to 19.51) | 0.3 (0.02 to 4.47) | 1.87 (0.11 to 29.73) | 1.42 (0.03 to 179.07) | 0.62 (0.05 to 6.39) | 0.54 (0.03 to 7.64) | Pembrolizumab plus Ipilimumab | 0.04 (0 to 0.82) | 0.25 (0 to 9.83) |
| 2.3 (0.33 to 37.87) | 3.08 (0.47 to 50.35) | **18.40 (1.12 to 896.79)** | 5.62 (0.12 to 369.53) | 2.44 (0.44 to 35.51) | 6.88 (0.48 to 151.42) | 12.96 (0.12 to 3324.78) | **14.88 (1.22 to 330.14)** | **15.47 (2.59 to 233.75)** | **20.32 (1.80 to 447.14)** | 6.43 (0.97 to 90.36) | **41.43 (7.10 to 610.31)** | 34.13 (0.99 to 4613.46) | 13.78 (2.53 to 180.08) | **11.99 (1.65 to 183.68)** | **24.02 (1.21 to 821.23)** | Placebo or No Intervention | 5.73 (0.27 to 161.11) |
| 0.40 (0.03 to 8.95) | 0.54 (0.04 to 12.33) | 3.11 (0.08 to 233.37) | 0.89 (0.01 to 108.66) | 0.43 (0.03 to 8.58) | 1.09 (0.11 to 20.02) | 2.20 (0.02 to 583.75) | 2.37 (0.3 to 41.38) | 2.80 (0.16 to 68.11) | 3.57 (0.2 to 107.82) | 1.19 (0.06 to 30.27) | 7.69 (0.45 to 191.96) | 6.05 (0.12 to 904.03) | 2.43 (0.16 to 54.69) | 2.08 (0.13 to 54.14) | 3.98 (0.10 to 221.64) | 0.17 (0.01 to 3.77) | Tremelimumab |

The league table show the relative effects of each intervention (the treatment on the column to the treatment of the row). The relative effects are measured as an odds ratio, with corresponding 95% credible intervals in parentheses. Bold indicates statistical significance.

### Table B.7.15. Estimated absolute event rate, hepatitis.

| Intervention | Proportion per 10,000  (95% confidence interval) |
| --- | --- |
| Atezolizumab | 7 (3 to 17) |
| Atezolizumab plus Conventional Therapy | 9 (4 to 21) |
| Avelumab | 53 (5 to 1,409) |
| Avelumab plus Conventional Therapy | 16 (0 to 625) |
| Durvalumab | 19 (3 to 183) |
| Durvalumab plus Conventional Therapy | 34 (0 to 3,850) |
| Durvalumab plus Tremelimumab | 43 (8 to 365) |
| Ipilimumab | 50 (13 to 189) |
| Ipilimumab plus Conventional Therapy | 61 (14 to 422) |
| Nivolumab | 21 (5 to 88) |
| Nivolumab plus Ipilimumab | 132 (35 to 570) |
| Nivolumab plus Ipilimumab plus Conventional Therapy | 96 (6 to 4,190) |
| Pembrolizumab | 44 (18 to 119) |
| Pembrolizumab plus Conventional Therapy | 38 (12 to 125) |
| Pembrolizumab plus Ipilimumab | 72 (5 to 1,046) |
| Tremelimumab | 18 (0 to 244) |

### Table B.7.16. League table, hypophysitis.

| Atezolizumab | 2.01 (0.24 to 28.23) | 1.95 (0.01 to 653.19) | 15.99 (0.1 to 8272.59) | 0.22 (0.01 to 4.18) | 0.18 (0 to 9.8) | 0.96 (0.01 to 221.43) | 1.39 (0.03 to 47.87) | 3.54 (0.09 to 96.01) | 0.61 (0.02 to 13.38) | 0.63 (0.01 to 16.19) | 3.7 (0.1 to 100.03) | 0.56 (0.02 to 13.31) | 1.23 (0.03 to 31.67) | 2.2 (0.01 to 633.57) | 0 (0 to 0.24) | 0.04 (0 to 6.11) |
| --- | --- | --- | --- | --- | --- | --- | --- | --- | --- | --- | --- | --- | --- | --- | --- | --- |
| 0.50 (0.04 to 4.09) | Atezolizumab plus Conventional Therapy | 0.91 (0.01 to 180.62) | 7.57 (0.07 to 2805.49) | 0.11 (0.01 to 0.68) | 0.09 (0 to 2.53) | 0.44 (0 to 77.5) | 0.68 (0.02 to 11.8) | 1.71 (0.07 to 19.52) | 0.3 (0.01 to 2.72) | 0.31 (0.01 to 3.31) | 1.81 (0.08 to 19.5) | 0.28 (0.01 to 2.67) | 0.61 (0.03 to 6.51) | 0.98 (0.01 to 212.81) | 0 (0 to 0.07) | 0.02 (0 to 1.56) |
| 0.51 (0.00 to 67.86) | 1.10 (0.01 to 97.74) | Avelumab | 7.25 (0.33 to 631.82) | 0.12 (0 to 3.1) | 0.09 (0 to 6.45) | 0.46 (0 to 130.62) | 0.74 (0 to 38.17) | 1.78 (0.01 to 81.92) | 0.3 (0 to 10.37) | 0.32 (0 to 14.15) | 1.89 (0.01 to 81.84) | 0.29 (0 to 11.58) | 0.61 (0.01 to 27.22) | 0.97 (0 to 537.85) | 0 (0 to 0.19) | 0.02 (0 to 3.94) |
| 0.06 (0.00 to 10.09) | 0.13 (0.00 to 15.01) | 0.14 (0.00 to 3.01) | Avelumab plus Conventional Therapy | 0.01 (0 to 0.64) | 0.01 (0 to 1.23) | 0.05 (0 to 28.25) | 0.08 (0 to 6.52) | 0.21 (0 to 15.55) | 0.03 (0 to 2.18) | 0.04 (0 to 2.77) | 0.22 (0 to 16.19) | 0.03 (0 to 2.12) | 0.07 (0 to 5.17) | 0.12 (0 to 93.64) | 0 (0 to 0.03) | 0 (0 to 0.64) |
| 4.60 (0.24 to 121.91) | **8.89 (1.46 to 143.25)** | 8.55 (0.32 to 971.60) | **77.06 (1.56 to 17354.89)** | Conventional Therapy | 0.84 (0.04 to 13.04) | 4.3 (0.07 to 537.5) | 5.92 (1.09 to 68.48) | 15.73 (4.48 to 66.09) | 2.63 (0.85 to 9.81) | 2.8 (0.74 to 12.54) | 16.55 (4.88 to 67.19) | 2.55 (0.85 to 8.39) | 5.46 (1.5 to 25.45) | 9.79 (0.17 to 1356.71) | 0.02 (0 to 0.33) | 0.22 (0 to 8.56) |
| 5.52 (0.10 to 483.75) | 11.21 (0.40 to 657.03) | 10.75 (0.15 to 2239.13) | 95.85 (0.82 to 34873.46) | 1.19 (0.08 to 22.52) | Durvalumab | 5.18 (0.03 to 1374.58) | 6.86 (1.16 to 96.79) | 19.4 (0.93 to 419.16) | 3.13 (0.17 to 75.07) | 3.4 (0.16 to 74.83) | 20.26 (1.03 to 458.55) | 3.06 (0.13 to 64.85) | 6.51 (0.31 to 166.84) | 12.48 (0.09 to 2613.18) | 0.02 (0 to 1.43) | 0.28 (0 to 20.74) |
| 1.04 (0.00 to 181.16) | 2.27 (0.01 to 338.17) | 2.16 (0.01 to 987.42) | 19.18 (0.04 to 15404.02) | 0.23 (0.00 to 13.48) | 0.19 (0.00 to 30.91) | Durvalumab plus Conventional Therapy | 1.42 (0.01 to 202.08) | 3.63 (0.03 to 258.29) | 0.63 (0 to 42.36) | 0.65 (0 to 53.52) | 3.85 (0.03 to 282.01) | 0.58 (0 to 43.26) | 1.29 (0.01 to 105.31) | 2.21 (0 to 1329.44) | 0 (0 to 0.55) | 0.04 (0 to 12.68) |
| 0.72 (0.02 to 36.92) | 1.48 (0.08 to 42.70) | 1.36 (0.03 to 229.34) | 12.00 (0.15 to 3868.09) | **0.17 (0.01 to 0.92)** | 0.15 (0.01 to 0.86) | 0.71 (0.00 to 111.05) | Durvalumab plus Tremelimumab | 2.66 (0.17 to 22.01) | 0.44 (0.03 to 3.8) | 0.47 (0.03 to 4.05) | 2.8 (0.18 to 23.04) | 0.43 (0.03 to 3.29) | 0.91 (0.06 to 8.64) | 1.61 (0.01 to 262.78) | 0 (0 to 0.09) | 0.04 (0 to 1.37) |
| 0.28 (0.01 to 11.56) | 0.59 (0.05 to 13.69) | 0.56 (0.01 to 71.87) | 4.84 (0.06 to 1544.72) | **0.06 (0.02 to 0.22)** | 0.05 (0.00 to 1.07) | 0.28 (0.00 to 38.18) | 0.38 (0.05 to 5.95) | Ipilimumab | 0.17 (0.03 to 1.01) | 0.18 (0.06 to 0.44) | 1.06 (0.45 to 2.64) | 0.16 (0.03 to 0.72) | 0.35 (0.05 to 2.42) | 0.59 (0.01 to 82.96) | 0 (0 to 0.02) | 0.01 (0 to 0.58) |
| 1.64 (0.07 to 61.29) | 3.33 (0.37 to 70.00) | 3.31 (0.10 to 450.00) | 28.69 (0.46 to 7239.72) | 0.38 (0.10 to 1.17) | 0.32 (0.01 to 5.90) | 1.59 (0.02 to 248.49) | 2.27 (0.26 to 30.97) | 5.90 (0.99 to 36.67) | Ipilimumab plus Conventional Therapy | 1.06 (0.17 to 6.66) | 6.31 (1.03 to 38.07) | 0.95 (0.18 to 5.41) | 2.09 (0.33 to 13.62) | 3.69 (0.05 to 555.23) | 0.01 (0 to 0.16) | 0.08 (0 to 3.9) |
| 1.59 (0.06 to 71.33) | 3.22 (0.30 to 78.49) | 3.15 (0.07 to 408.43) | 27.42 (0.36 to 8035.94) | 0.36 (0.08 to 1.34) | 0.29 (0.01 to 6.08) | 1.54 (0.02 to 214.15) | 2.14 (0.25 to 32.41) | **5.49 (2.25 to 15.43)** | 0.94 (0.15 to 6.05) | Nivolumab | 5.88 (2.05 to 18.76) | 0.91 (0.17 to 4.75) | 1.98 (0.27 to 13.66) | 3.32 (0.05 to 483.59) | 0.01 (0 to 0.12) | 0.08 (0 to 3.44) |
| 0.27 (0.01 to 10.06) | 0.55 (0.05 to 12.30) | 0.53 (0.01 to 68.74) | 4.56 (0.06 to 1299.81) | **0.06 (0.01 to 0.20)** | **0.05 (0.00 to 0.97)** | 0.26 (0.00 to 35.51) | 0.36 (0.04 to 5.51) | 0.94 (0.38 to 2.23) | **0.16 (0.03 to 0.97)** | **0.17 (0.05 to 0.49)** | Nivolumab plus Ipilimumab | 0.15 (0.03 to 0.73) | 0.33 (0.05 to 2.41) | 0.57 (0.01 to 84.04) | 0 (0 to 0.02) | 0.01 (0 to 0.56) |
| 1.78 (0.08 to 61.73) | 3.59 (0.37 to 67.10) | 3.47 (0.09 to 423.59) | 30.12 (0.47 to 7826.46) | 0.39 (0.12 to 1.18) | 0.33 (0.02 to 7.44) | 1.71 (0.02 to 215.91) | 2.33 (0.30 to 35.16) | **6.21 (1.39 to 29.97)** | 1.05 (0.18 to 5.55) | 1.10 (0.21 to 6.05) | **6.51 (1.37 to 33.45)** | Pembrolizumab | 2.19 (0.48 to 10.62) | 3.75 (0.08 to 456.76) | 0.01 (0 to 0.12) | 0.09 (0 to 3.91) |
| 0.81 (0.03 to 29.46) | 1.65 (0.15 to 36.40) | 1.65 (0.04 to 190.49) | 14.29 (0.19 to 3649.21) | **0.18 (0.04 to 0.67)** | 0.15 (0.01 to 3.26) | 0.77 (0.01 to 117.09) | 1.10 (0.12 to 16.55) | 2.87 (0.41 to 19.29) | 0.48 (0.07 to 3.01) | 0.51 (0.07 to 3.67) | 3.05 (0.41 to 21.16) | 0.46 (0.09 to 2.09) | Pembrolizumab plus Conventional Therapy | 1.75 (0.03 to 242.57) | 0 (0 to 0.08) | 0.04 (0 to 1.85) |
| 0.46 (0.00 to 113.01) | 1.02 (0.00 to 152.61) | 1.03 (0.00 to 422.01) | 8.45 (0.01 to 6618.93) | 0.10 (0.00 to 5.97) | 0.08 (0.00 to 11.28) | 0.45 (0.00 to 209.96) | 0.62 (0.00 to 71.47) | 1.68 (0.01 to 108.16) | 0.27 (0.00 to 20.64) | 0.3 (0.00 to 20.86) | 1.77 (0.01 to 113.06) | 0.27 (0.00 to 12.68) | 0.57 (0.00 to 39.01) | Pembrolizumab plus Ipilimumab | 0 (0 to 0.27) | 0.02 (0 to 7.99) |
| **348.93 (4.21 to 99746.52)** | **696.86 (14.73 to 164574.93)** | **736.39 (5.39 to 521428.99)** | **6505.03 (29.77 to 5339993.83)** | **65.00 (3.04 to 8233.79)** | 63.67 (0.70 to 17852.57) | **348.4 (1.83 to 175346.88)** | **446.26 (11.30 to 108031.70)** | **1015.61 (50.19 to 145262.45)** | **186.81 (6.14 to 23338.27)** | **183.81 (8.11 to 26849.78)** | **1103.77 (50.88 to 153494.05)** | **168.47 (8.24 to 18638.85)** | **370.97 (12.89 to 55854.72)** | **738.97 (3.67 to 530727.32)** | Placebo or No Intervention | 12.19 (0.65 to 1357) |
| 23.23 (0.16 to 6855.42) | 45.71 (0.64 to 11060.62) | 50.69 (0.25 to 35000.82) | 467.70 (1.56 to 291947.30) | 4.56 (0.12 to 582.86) | 3.57 (0.05 to 870.11) | 23.74 (0.08 to 13995.08) | 26.42 (0.73 to 5613.99) | 69.46 (1.73 to 10480.96) | 13.05 (0.26 to 1583.68) | 12.67 (0.29 to 2127.95) | **76.59 (1.80 to 11335.49)** | 11.50 (0.26 to 1535.46) | 26.31 (0.54 to 3583.37) | 49.88 (0.13 to 40343.28) | 0.08 (0.00 to 1.54) | Tremelimumab |

The league table show the relative effects of each intervention (the treatment on the column to the treatment of the row). The relative effects are measured as an odds ratio, with corresponding 95% credible intervals in parentheses. Bold indicates statistical significance.

### Table B.7.17. Estimated absolute event rate, hypophysitis.

| Intervention | Proportion per 10,000  (95% confidence interval) |
| --- | --- |
| Atezolizumab | 127 (6 to 2,550) |
| Atezolizumab plus Conventional Therapy | 243 (40 to 2,868) |
| Avelumab | 234 (8 to 7,317) |
| Avelumab plus Conventional Therapy | 1,778 (43 to 9,798) |
| Durvalumab | 23 (1 to 353) |
| Durvalumab plus Conventional Therapy | 119 (1 to 6,014) |
| Durvalumab plus Tremelimumab | 163 (30 to 1,612) |
| Ipilimumab | 422 (124 to 1,565) |
| Ipilimumab plus Conventional Therapy | 73 (23 to 268) |
| Nivolumab | 78 (20 to 340) |
| Nivolumab plus Ipilimumab | 444 (135 to 1,587) |
| Pembrolizumab | 71 (23 to 230) |
| Pembrolizumab plus Conventional Therapy | 150 (41 to 666) |
| Pembrolizumab plus Ipilimumab | 267 (4 to 7,920) |
| Tremelimumab | 6 (0 to 234) |

## Table B.8. Individual interventions ranks (SUCRA)

### Table B.8.1. Ranks, individual interventions, overall immune-related serious adverse events.

|  | SUCRA |
| --- | --- |
| Conventional Therapy | 95% |
| Placebo or No Intervention | 95% |
| Atezolizumab | 82% |
| Atezolizumab plus Conventional Therapy | 74% |
| Durvalumab plus Conventional Therapy | 68% |
| Durvalumab | 63% |
| Pembrolizumab plus Conventional Therapy | 58% |
| Nivolumab plus Ipilimumab plus Conventional Therapy | 55% |
| Pembrolizumab | 53% |
| Nivolumab | 49% |
| Avelumab plus Conventional Therapy | 49% |
| Ipilimumab plus Conventional Therapy | 43% |
| Nivolumab plus Conventional Therapy | 39% |
| Avelumab | 36% |
| Durvalumab plus Tremelimumab | 28% |
| Pembrolizumab plus Ipilimumab | 23% |
| Tremelimumab | 16% |
| Ipilimumab | 14% |

### Table B.8.2. Ranks, individual interventions, pneumonitis.

|  | SUCRA |
| --- | --- |
| Conventional Therapy | 88% |
| Ipilimumab plus Conventional Therapy | 86% |
| Placebo or No Intervention | 77% |
| Atezolizumab | 73% |
| Atezolizumab plus Conventional Therapy | 69% |
| Nivolumab plus Ipilimumab plus Conventional Therapy | 67% |
| Durvalumab plus Conventional Therapy | 61% |
| Tremelimumab | 58% |
| Durvalumab | 55% |
| Pembrolizumab plus Conventional Therapy | 50% |
| Nivolumab plus Conventional Therapy | 45% |
| Avelumab plus Conventional Therapy | 43% |
| Avelumab | 35% |
| Nivolumab | 35% |
| Pembrolizumab | 31% |
| Durvalumab plus Tremelimumab | 28% |
| Ipilimumab | 25% |
| Pembrolizumab plus Ipilimumab | 16% |
| Nivolumab plus Ipilimumab | 8% |

### Table B.8.3. Ranks, individual interventions, myocarditis.

|  | SUCRA |
| --- | --- |
| Durvalumab plus Tremelimumab | 86% |
| Placebo or No Intervention | 81% |
| Pembrolizumab | 71% |
| Conventional Therapy | 71% |
| Nivolumab | 70% |
| Pembrolizumab plus Conventional Therapy | 54% |
| Durvalumab plus Conventional Therapy | 43% |
| Nivolumab plus Conventional Therapy | 43% |
| Atezolizumab plus Conventional Therapy | 41% |
| Nivolumab plus Ipilimumab | 39% |
| Pembrolizumab plus Ipilimumab | 36% |
| Durvalumab | 30% |
| Avelumab | 17% |
| Atezolizumab | 17% |

### Table B.8.4. Ranks, individual interventions, colitis.

|  | SUCRA |
| --- | --- |
| Placebo or No Intervention | 99% |
| Conventional Therapy | 90% |
| Nivolumab | 80% |
| Atezolizumab | 70% |
| Durvalumab | 67% |
| Pembrolizumab | 65% |
| Durvalumab plus Conventional Therapy | 63% |
| Pembrolizumab plus Conventional Therapy | 59% |
| Avelumab plus Conventional Therapy | 54% |
| Nivolumab plus Ipilimumab plus Conventional Therapy | 52% |
| Atezolizumab plus Conventional Therapy | 50% |
| Ipilimumab | 31% |
| Durvalumab plus Tremelimumab | 26% |
| Avelumab | 25% |
| Ipilimumab plus Conventional Therapy | 21% |
| Pembrolizumab plus Ipilimumab | 21% |
| Nivolumab plus Ipilimumab | 17% |
| Tremelimumab | 11% |

### Table B.8.5. Ranks, individual interventions, nephritis.

|  | SUCRA |
| --- | --- |
| Conventional Therapy | 82% |
| Pembrolizumab plus Conventional Therapy | 79% |
| Ipilimumab plus Conventional Therapy | 75% |
| Placebo or No Intervention | 75% |
| Nivolumab | 67% |
| Durvalumab plus Tremelimumab | 64% |
| Nivolumab plus Ipilimumab | 59% |
| Nivolumab plus Ipilimumab plus Conventional Therapy | 51% |
| Durvalumab | 49% |
| Pembrolizumab | 47% |
| Ipilimumab | 46% |
| Pembrolizumab plus Ipilimumab | 31% |
| Avelumab | 25% |
| Tremelimumab | 23% |
| Atezolizumab | 16% |
| Atezolizumab plus Conventional Therapy | 13% |

###

### Table B.8.6. Ranks, individual interventions, pancreatitis.

|  | SUCRA |
| --- | --- |
| Placebo or No Intervention | 82% |
| Pembrolizumab plus Conventional Therapy | 71% |
| Atezolizumab plus Conventional Therapy | 65% |
| Conventional Therapy | 64% |
| Avelumab plus Conventional Therapy | 61% |
| Durvalumab | 58% |
| Tremelimumab | 58% |
| Nivolumab | 56% |
| Ipilimumab | 54% |
| Pembrolizumab | 49% |
| Ipilimumab plus Conventional Therapy | 45% |
| Atezolizumab | 43% |
| Nivolumab plus Ipilimumab | 40% |
| Durvalumab plus Tremelimumab | 30% |
| Nivolumab plus Ipilimumab plus Conventional Therapy | 14% |
| Avelumab | 9% |

###

### Table B.8.7. Ranks, individual interventions, hepatitis.

|  | SUCRA |
| --- | --- |
| Placebo or No Intervention | 93% |
| Atezolizumab | 84% |
| Conventional Therapy | 83% |
| Atezolizumab plus Conventional Therapy | 76% |
| Avelumab plus Conventional Therapy | 60% |
| Tremelimumab | 60% |
| Durvalumab | 58% |
| Nivolumab | 58% |
| Durvalumab plus Conventional Therapy | 46% |
| Pembrolizumab plus Conventional Therapy | 41% |
| Pembrolizumab | 37% |
| Durvalumab plus Tremelimumab | 37% |
| Avelumab | 35% |
| Ipilimumab | 34% |
| Pembrolizumab plus Ipilimumab | 30% |
| Ipilimumab plus Conventional Therapy | 30% |
| Nivolumab plus Ipilimumab plus Conventional Therapy | 26% |
| Nivolumab plus Ipilimumab | 13% |

###

### Table B.8.8. Ranks, individual interventions, hypophysitis.

|  | SUCRA |
| --- | --- |
| Placebo or No Intervention | 99% |
| Tremelimumab | 85% |
| Conventional Therapy | 80% |
| Durvalumab | 76% |
| Pembrolizumab | 60% |
| Ipilimumab plus Conventional Therapy | 58% |
| Nivolumab | 57% |
| Durvalumab plus Conventional Therapy | 47% |
| Atezolizumab | 47% |
| Pembrolizumab plus Conventional Therapy | 42% |
| Durvalumab plus Tremelimumab | 39% |
| Avelumab | 37% |
| Pembrolizumab plus Ipilimumab | 36% |
| Atezolizumab plus Conventional Therapy | 32% |
| Ipilimumab | 21% |
| Nivolumab plus Ipilimumab | 20% |
| Avelumab plus Conventional Therapy | 12% |

**References for Data Supplement**

1. van Valkenhoef G, Dias S, Ades AE, Welton NJ. Automated generation of node-splitting models for assessment of inconsistency in network meta-analysis. Research Synthesis Methods. 2016 Mar 1;7(1):80–93.

2. Dias S, Sutton AJ, Ades AE, Welton NJ. Evidence synthesis for decision making 2: A generalized linear modeling framework for pairwise and network meta-analysis of randomized controlled trials. Medical Decision Making. 2013 Jul;33(5):607–17.

3. Rhodes KM, Turner RM, Higgins JPT. Predictive distributions were developed for the extent of heterogeneity in meta-analyses of continuous outcome data. Journal of Clinical Epidemiology. 2015 Jan 1;68(1):52–60.

4. Turner RM, Davey J, Clarke MJ, Thompson SG, Higgins JP. Predicting the extent of heterogeneity in meta-analysis, using empirical data from the Cochrane Database of Systematic Reviews. International Journal of Epidemiology. 2012 Jun;41(3):818–27.

5. Li T, Higgins JP, Deeks JJ, Jpt H. Chapter 5: Collecting data [Internet]. 2022. Available from: [www.training.cochrane.org/handbook](http://www.training.cochrane.org/handbook).

6. Higgins JPT, Thompson SG, Deeks JJ, Altman DG. Measuring inconsistency in meta-analyses Testing for heterogeneity.

7. Dias S, Welton NJ, Sutton AJ, Caldwell DM, Lu G, Ades AE. Evidence synthesis for decision making 4: Inconsistency in networks of evidence based on randomized controlled trials. Medical Decision Making. 2013 Jul;33(5):641–56.

8. Page MJ, Higgins JP, Sterne JA, Jpt H. Chapter 13: Assessing risk of bias due to missing results in a synthesis [Internet]. 2022. Available from: [www.training.cochrane.org/handbook](http://www.training.cochrane.org/handbook).

9. Inthout J, Ioannidis JP, Borm GF. The Hartung-Knapp-Sidik-Jonkman method for random effects meta-analysis is straightforward and considerably outperforms the standard DerSimonian-Laird method. BMC Medical Research Methodology. 2014 Feb 18;14(1).

10. Röver C, Knapp G, Friede T. Hartung-Knapp-Sidik-Jonkman approach and its modification for random-effects meta-analysis with few studies. BMC Medical Research Methodology. 2015;15(1).

11. Brooks SP, Gelman A. General methods for monitoring convergence of iterative simulations)? Journal of Computational and Graphical Statistics. 1998;7(4):434–55.

12. Salanti G, Ades AE, Ioannidis JPA. Graphical methods and numerical summaries for presenting results from multiple-treatment meta-analysis: An overview and tutorial. Journal of Clinical Epidemiology. 2011 Feb;64(2):163–71.

13. Guyatt GH, Oxman AD, Schünemann HJ, Tugwell P, Knottnerus A. GRADE guidelines: A new series of articles in the Journal of Clinical Epidemiology. Journal of Clinical Epidemiology. 2011 Apr;64(4):380–2.
